# Characterization of Metagenomes in Urban Aquatic Compartments Reveals High Prevalence of Clinically Relevant Antibiotic Resistance Genes in Wastewaters

**DOI:** 10.3389/fmicb.2017.02200

**Published:** 2017-11-16

**Authors:** Charmaine Ng, Martin Tay, Boonfei Tan, Thai-Hoang Le, Laurence Haller, Hongjie Chen, Tse H. Koh, Timothy M. S. Barkham, Janelle R. Thompson, Karina Y.-H. Gin

**Affiliations:** ^1^Department of Civil and Environmental Engineering, National University of Singapore, Singapore, Singapore; ^2^Centre for Environmental Sensing and Modeling, Singapore-MIT Alliance for Research and Technology Centre, Singapore, Singapore; ^3^Department of Environmental Engineering, Ho Chi Minh City International University, Ho Chi Minh City, Vietnam; ^4^Department of Pathology, Singapore General Hospital, Singapore, Singapore; ^5^Department of Laboratory Medicine, Tan Tock Seng Hospital, Singapore, Singapore; ^6^NUS Environmental Research Institute, National University of Singapore, Singapore, Singapore

**Keywords:** comparative metagenomics, antibiotic resistant genes, wastewaters, hospital, municipal, water body, tributary, beta-lactamase resistant genes

## Abstract

The dissemination of antimicrobial resistance (AMR) is an escalating problem and a threat to public health. Comparative metagenomics was used to investigate the occurrence of antibiotic resistant genes (ARGs) in wastewater and urban surface water environments in Singapore. Hospital and municipal wastewater (*n* = 6) were found to have higher diversity and average abundance of ARGs (303 ARG subtypes, 197,816 x/Gb) compared to treated wastewater effluent (*n* = 2, 58 ARG subtypes, 2,692 x/Gb) and surface water (*n* = 5, 35 subtypes, 7,985 x/Gb). A cluster analysis showed that the taxonomic composition of wastewaters was highly similar and had a bacterial community composition enriched in gut bacteria (*Bacteroides, Faecalibacterium, Bifidobacterium, Blautia, Roseburia, Ruminococcus*), the *Enterobacteriaceae* group (*Klebsiella, Aeromonas, Enterobacter*) and opportunistic pathogens (*Prevotella, Comamonas, Neisseria*). Wastewater, treated effluents and surface waters had a shared resistome of 21 ARGs encoding multidrug resistant efflux pumps or resistance to aminoglycoside, macrolide-lincosamide-streptogramins (MLS), quinolones, sulfonamide, and tetracycline resistance which suggests that these genes are wide spread across different environments. Wastewater had a distinctively higher average abundance of clinically relevant, class A beta-lactamase resistant genes (i.e., *bla*_KPC_, *bla*_CTX-M_, *bla*_SHV_, *bla*_TEM_). The wastewaters from clinical isolation wards, in particular, had a exceedingly high levels of *bla*_KPC-2_ genes (142,200 x/Gb), encoding for carbapenem resistance. Assembled scaffolds (16 and 30 kbp) from isolation ward wastewater samples indicated this gene was located on a Tn3-based transposon (Tn*4401*), a mobilization element found in *Klebsiella pneumonia* plasmids. In the longer scaffold, transposable elements were flanked by a toxin–antitoxin (TA) system and other metal resistant genes that likely increase the persistence, fitness and propagation of the plasmid in the bacterial host under conditions of stress. A few bacterial species (*Enterobacter cloacae, Klebsiella pneumoniae, Citrobacter freundii, Pseudomonas aeruginosa*) that were cultured from the isolation ward wastewaters on CHROMagar media harbored the *bla*_KPC-2_ gene. This suggests that hospital wastewaters derived from clinical specialty wards are hotspots for the spread of AMR. Assembled scaffolds of other mobile genetic elements such as IncQ and IncF plasmids bearing quinolone resistance genes (*qnrS1, qnrS2*) and the class A beta-lactamase gene (*bla*_TEM-1_) were recovered in wastewater samples which may aid the transfer of AMR.

## Introduction

Antimicrobial resistance (AMR) is a growing global health threat due to concerns over the reduced clinically efficacy of current antibiotics in the treatment of bacterial infections, especially for hospital-acquired infections. The excessive use of last-resort antibiotics, such as extended-spectrum-beta-lactams (ESBLs) and carbapenems has resulted in an increased prevalence of carbapenem-resistant *Enterobacteriaceae* (CRE) which have spread globally due to poor infection control and a highly mobile and connected world (Queenan and Bush, 2007; Nordmann et al., 2011; Papp-Wallace et al., 2011). Gram-negative bacteria, specifically *Enterobacteriaceae* are common causes of community- and hospital acquired infections and frequently harbor multiple antibiotic resistance mechanisms (Vasoo et al., 2015). The drug resistance problem extends beyond the hospital setting. Extensive studies have demonstrated that wastewater, specifically hospital discharges, and wastewater treatment plants (WWTPs) are important reservoirs of antibiotic resistant bacteria (ARB) and antibiotic resistance genes (ARGs) (Volkmann et al., 2004; Zhang et al., 2009; Berglund et al., 2014; Al-Jassim et al., 2015; Li J. et al., 2015; Xu J. et al., 2015; Mao et al., 2015; Chagas et al., 2016). Wastewater contains an amalgam of human and animal excrement, commensal and pathogenic bacteria, high loads of nutrients, detergents, antimicrobial agents, and heavy metals that may harbor and preferentially select for ARB (Novo et al., 2013). Studies on removal rates of opportunistic pathogens in conventional wastewater treatment processes have shown the persistence of *Pseudomonas* spp. and *Aeromonas hydrophila* in chlorinated effluent and an increased abundance of *Mycobacterium* spp. (Al-Jassim et al., 2015). Documented evidence points to wastewater treatment processes inducing the propagation of selected ARG subtypes (Alexander et al., 2015; Li J. et al., 2015; Mao et al., 2015; Xu G. et al., 2015) and this has prompted investigations into assessing the microbial risk associated with reusing treated wastewater effluent in agricultural irrigation (Al-Jassim et al., 2015).

The globalization of medical healthcare, influx of travelers and a transient workforce has spawned the emergence of broader population consequences and the importation of pathogens harboring antibiotic resistance genes through carriage in the human microbiome. Singapore is a travel hub, receiving an average almost 15 million foreign tourists per year over the last 2 years (Singapore Tourism Board, 2014), with 70% of overseas visitors seeking medical treatment in Singapore hospitals coming from the South East Asian region (Horowitz and Rosensweig, 2007; Smith et al., 2009). Visitors engaging in medical tourism abroad may exacerbate the carriage and spread of pathogens and antibiotic resistant determinants upon returning home (Kennedy and Collignon, 2010; Kumarasamy et al., 2010; Lopez et al., 2010; Struelens et al., 2010; Rogers et al., 2011; Van der Bij and Pitout, 2012; Wilson and Chen, 2012; Chen and Wilson, 2013). Understanding the dynamics and occurrence of AR from hotspots (wastewater), through the wastewater treatment process and to the urban environment (surface waters) provides bearing on the extent of the spread of AMR in densely populated cities that are reliant on an urban water cycle.

The over-reliance of culture-based methods of single strains which is consider the standard in investigating clinical resistance has vastly underestimated and narrowed insights into the composition of resistomes in different environments (Dantas, 2017). Recently, there has been increased interest in utilizing metagenomics as a tool to evaluate antibiotic resistance in the human microbiota and different environments to assess the risk to human health (Bengtsson-Palme et al., 2017). The objective of this study was to characterize ARG profiles, and potential vectors of transfer such as mobile genetic elements (MGE) in wastewater; treated effluent and urban environmental surface waters using assembled metagenomes. Assembled metagenomes were interrogated against the comprehensive antibiotic resistant database (CARD^1^) and Resfam database^2^ for the broad identification of ARGs in each ecological niche. The phylogenic composition of bacteria was compared between samples and scaffolds containing specific plasmid replicons were identified using PlasmidFinder^3^. Hospital wastewater is a high source of opportunistic pathogens, ARGs, antimicrobials and chemical agents. This work focuses on exploring the diversity of β-lactamase resistant genes and inspected the gene neighborhoods of scaffolds containing emergent carbapenem resistant genes (i.e., *bla*_KPC_) to identify genetic elements that promote proliferation and persistence of these ARGs in the hospital setting.

## Materials and Methods

### Samples and Sequencing

Five clinical wastewater samples were collected from two hospitals in Singapore. Two hospital blocks (i.e., block A of hospital 1 and block B of hospital 1) (1,597 beds) were sampled once a week over a period of 2 weeks from a manhole receiving direct sewage from each block. These two blocks were differentiated based on their ward types, with block A (H1, H2) consisting of clinical isolation wards and block B (H3, H4) consisting of general wards. For hospital 2 (H5; 1,500 beds), one sample was collected from the main manhole discharging mixed wastewater from the entire hospital. Three samples were collected at different treatment stages of a municipal wastewater treatment plant, an influent (WW) and effluent samples (TW1, TW2) from the Modified Ludzack-Ettinger (MLE) process whereby wastewaters undergo anoxic and aerobic treatment. Surface waters were collected from three urban tributaries (BH, BI, BB) and an urban water body (RA) located southeast of the island within the commercial district. As a comparison, surface waters were sampled from a forested water body in central part of Singapore (MA). More details of sampling sites are found in **Table 1**. A volume of 1 L of wastewater and 10 – 20 L of surface water was collected using sterile plastic bottles and transported to the laboratory for immediate processing. For DNA extraction, water samples were filtered on 0.45 μm cellulose nitrate membranes (Sartorius stedim, Goettingen, Germany) until the membrane was saturated for maximum biomass yield. DNA was extracted using a PowerWater DNA isolation kit (Mo Bio Laboratories, Inc., Carlsbad, CA, United States) according to the manufacturer’s instructions. The quantity and quality of DNA was measured using a Qubit 3.0 Fluorometer (Thermo Fisher Scientific, Waltham, MA, United States).

**Table 1:** Water sample sources for antibiotic resistome profile analysis.

Source of sample	Sample description	Sample ID	IMG ID	SRA ID
Hospital wastewater discharge	Clinical isolation ward (Hospital 1, week 1)	H1	3300008488	SRR5997548
	Clinical isolation ward (Hospital 1, week 2)	H2	3300008070	SRR5997541
	General ward (Hospital 1, week 1)	H3	3300008069	SRR5997540
	General ward (Hospital 1, week 2)	H4	3300008487	SRR5997552
	Entire hospital (Hospital 2)	H5	3300008067	SRR5997551
Wastewater treatment plant	Municipal wastewater influent	WW	3300008071	SRR5997546
	Post anaerobic/aerobic treated effluent (Train 1)	TW1	3300008507	SRR5997542
	Post anaerobic/aerobic treated effluent (Train 2)	TW2	3300008065	SRR5997545
Surface waters	Urban tributary (Site 1)	BH	3300008066	SRR5997544
	Urban tributary (Site 2)	BI	3300008508	SRR5997549
	Urban tributary (Site 3)	BB	3300008509	SRR5997550
	Forested water body (Site 4)	MA	3300008072	SRR5997543
	Urban water body (Site 5)	RA	3300008510	SRR5997547

Sequencing was performed at the Singapore Centre of Environmental Life Sciences and Engineering (SCELSE). Library preparation was performed according to Illumina’s TruSeq Nano DNA Sample Preparation protocol. DNA samples were sheared on a Covaris S220 (Covaris, United States) to ∼450 bp following manufacturer’s recommendation. Each library was uniquely tagged with one of Illumina’s TruSeq DNA HT dual barcode combination to enable library pooling for sequencing. The finished libraries were quantitated using Invitrogen’s Picogreen assay and the average library size was determined on a Bioanalyzer 2100, using a DNA 7500 chip (Agilent Technologies, United States). Library concentrations were normalized to 4 nM and validated by qPCR on a ViiA-7 real-time thermocycler (Applied Biosystems, United States), using qPCR primers recommended in Illumina’s qPCR protocol, and Illumina’s PhiX control library as standard. These libraries were pooled at equimolar concentrations and sequenced in a lane on Illumina HiSeq2500 sequencer in rapid mode at a final concentration of 10 pM and a read length of 250 bp paired-end.

### Metagenomic Assembly and ORF Prediction

The quality of the sequenced library was assessed using BBDuk (BBTools package) where reads were trimmed to remove adaptor sequences and base calls with Phred scores above Q20 were accepted. Only paired reads that were greater than 75 bp in length were retained. Subsequently BBDuk was also used for removal of reads homologous to PhiX phage, which is commonly used as a control on the Illumina sequencing platform. A total of 13 datasets were generated and the number of paired reads which passed the quality filtering ranged between 1,928,883 and 3,202,560 (Supplementary Table S1). Quality filtered paired reads were assembled using CLC workbench (Version 6.0.2, CLC Bio, Aarhaus, Denmark) using the default settings, and sequence assemblies were submitted to the Integrated Microbial Genomes and Microbiome Samples (IMG^4^) for ORF prediction and automated annotation. Metagenomic datasets were deposited under the IMG Genome IDs and raw sequence reads can be downloaded via the NCBI short read archive (SRA) under accession numbers SRR5997540 – SRR5997552 (**Table 1**).

### Plasmid Identification

To detect and characterize plasmids, which may function as vectors for the transfer of ARGs, assembled contigs were analyzed using PlasmidFinder 1.3^5^ at a threshold of a 95% nucleotide sequence identity match. This database consists of 116 replicon sequences derived from 559 fully sequenced plasmids of multidrug resistant *Enterobacteriaceae*.

### Identification of ARG-Like ORFs

Predicted ORFs were interrogated against an “Antimicrobial Resistance Protein Database” (AMRPD) using BLASTP (*E*-value ≤ 10^-5^). We created the AMRPD by combining protein sequences in the CARD database^6^ and the Resfam AR Proteins database, v1.2^7^, which yielded a total of 5,331 ARG protein sequences. A queried ORF was regarded as an ARG-like sequence under the criteria of >70% similarity with a coverage of >70%. All identified ARG-like ORFs were assigned to ARG subtypes (e.g., *sul1, sul2, sul3*) and subsequently organized into ARG types (antibiotic class, e.g., sulfonamides) using the CARD database as a reference. The number of ARG-like ORFs identified in each sample is presented in Supplementary Table S1. All ARG annotations and IMG gene IDs are found in Supplementary Table S8.

### ARG Abundance Analysis

To estimate the abundance of ARG-like ORFs in the different water samples, coverage was calculated using the following formula as described in Ma et al. (2016).

Coverage of ARG-like ORF expressed as x/Gb:

$$\frac{(\text{Number of mapped read})\times \text{250/Length of ARG like ORF}}{\text{Size of metagenomic dataset}(\text{Gb})}$$

Each ARG-like ORF was then assigned to an ARG subtype based on the BLASTP assignment according to the threshold mentioned previously, and the abundance for each of the ARG types was calculated by summing the coverage values of each ARG subtype classified to common antibiotic classes.

### Microbial Community Structure

To characterize the microbial community structure between samples, predicted ORFs were aligned against the NCBI non-redundant (NR) protein database using DIAMOND with the default parameters. The similarity search results were analyzed through MEGAN 5^8^ by assigning BLAST results to NCBI taxonomies with the lowest common ancestor (LCA) algorithm. Identified taxa are found in Supplementary Table S7.

### Statistical Analysis

Primer version 7 (Clarke and Gorley, 2015) was used to analyze clustering patterns of the microbial community structure (at the genus level) in the various types of water samples. A log(X+1) transformation was applied to datasets and a resemblance matrix was calculated by Bray–Curtis analysis. Clustering patterns were statistically validated by an Analysis of Similarity (ANOSIM) procedure using 999 iterations to test the significance of the clustered groups. A SIMPER analysis was used to determine the similarities in microbial community composition between samples.

### Phylogenetic Identification of *bla*_KPC_ Bearing Bacteria in Wastewater Samples

CHROMagar^TM^ Orientation, CHROMagar^TM^ KPC and ESBL (CHROMagar, Paris, France) were used to isolate bacteria that were resistant to carbapenems and/or ESBLs in hospital wastewater. ESBL and KPC supplements were added to the base medium at a final concentration of 570 and 400 μg/mL according to manufacturer’s instructions. Samples were serially diluted in 1x phosphate buffered saline (PBS, Vivantis Technologies, Malaysia) and 10 mL of sample was filtered on to 0.45 μm nitrocellulose membranes (Sartorius stedim, Goettingen, Germany). Plates were then incubated at 37°C for 24 h. Bacterial isolates that grew on plates were re-streaked onto fresh media to ensure pure cultures were obtained. Colony PCR was performed and 16S rRNA genes were amplified using 27F (5′-AGA GTT TGA TYM TGG CTC AG-3′) and 1492R (5′-GGY TAC CTT GTT ACG ACT T-3′), and primer set F (5′–ATG TCA CTG TAT CGC CGT CT-3′) and R (5′-TTT TCA GAG CCT TAC TGC CC-3′) to screen for the presence of the *bla*_KPC_ gene (Bratu et al., 2005). PCR products were run on a 1% agarose gel and products purified using the Expin cleanup kit (GeneAll Biotechnology, Seoul, South Korea). Purified products were sent for capillary sequencing at AIT biotech (Singapore), and sequences were manually assessed using the Bioedit software and queried against the National Centre for Biotechnology Information (NCBI) 16S rRNA gene database for archaea and bacteria for taxonomic identification and the non-redundant database for *bla*_KPC_ identification using BLASTN.

## Results

### Characterization of the Antibiotic Resistome

**FIGURE 1:**
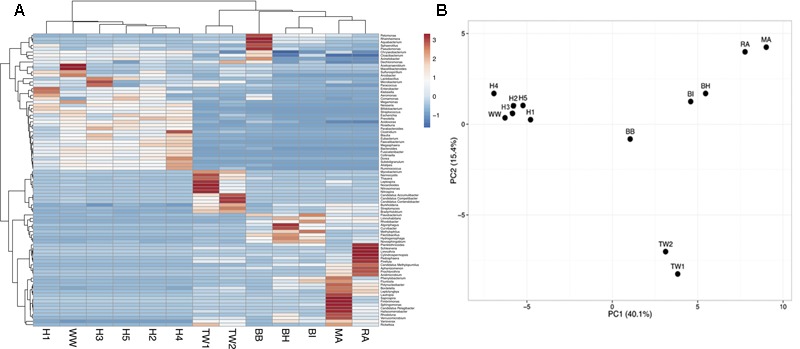
**(A)** A heat map visualizing the distribution pattern of bacterial genera based on relative abundance (*z*-score). **(B)** Principal component analysis (PCA) of the bacterial community composition (genus-level) across all sample types. Only taxa present at >1% were included in the analysis. The bacterial community composition of wastewaters (H1-5, WW) was significantly different from treated effluent and surface waters (TW1-2, BH, BI, BB, MA, RA).

A principal component analysis of microbial community structure at the genus level showed wastewater samples formed one cluster (H1, H2, H3, H4, H5, WW), which was significantly different (*p* = 0.008, ANOSIM) from treated effluent and surface water samples (TW1, TW2, BH, BI, BB, MA, RA) formed another (**Figure 1**). A SIMPER analysis showed an average dissimilarity of 62% in bacterial community structure between the two groups. Wastewaters were enriched with genera associated with gut microbiomes (*Bacteroides, Faecalibacterium, Bifidobacterium, Blautia, Roseburia*, and *Ruminococcus*), members of the *Enterobacteriaceae* group (*Klebsiella, Aeromonas, Enterobacter*) and other opportunistic pathogens (*Prevotella, Comamonas, Neisseria*) while the treated effluent and surface water group had less of these genera present, and more enriched in *Limnohabitans*, a planktonic bacteria. Metagenomes from hospitals and municipal wastewater had a higher percentage of ARG-like ORFs identified (0.09–0.16%) compared to treated effluent and surface water (0.01–0.05%, Supplementary Table S1). These ORFs were assigned to 344 subtypes, which were classified into 22 main ARG types. Multidrug resistant efflux pumps, aminoglycoside and quinolone accounted for >66% of ARG subtypes identified in each sample (Supplementary Table S2).

### The Shared Antibiotic Resistome

Wastewater had the highest diversity (303 subtypes) of ARGs followed by surface water (58 subtypes) and treated effluent (35 subtypes, Supplementary Figure S1). A core resistome of 21 ARG subtypes were shared between these three clusters, which included those encoding multidrug resistance efflux pumps (*adeJ, macB, mdtB, mexKT, msrE, pmrE*, RND antibiotic efflux pump), and resistance to aminoglycoside [*ant(3), aph(3″), aph(6), ant(3″)-Ia*], macrolide (*ereA*), macrolide-lincosamide-streptogramin [MLS (*ermF*)], quinolone (fluoroquinolone resistant DNA topoisomerase, *qnrS2*), sulfonamides (*sul1, sul2*) or tetracycline (tet efflux pump, tet ribosomal protection protein, *tetX*) (Supplementary Table S3A). Genes encoding for *aph(3″)*, RND antibiotic efflux pumps and fluoroquinolone resistance DNA topoisomerase were detected in each sample with a coverage ranging between 73-87,965 x/Gb, 26-1,097 x/Gb, and 232-11,966 x/Gb, respectively (Supplementary Table S3A).

The wastewater and surface water shared 28 ARG subtypes, which spanned from genes encoding for resistance against aminoglycosides (*aph(3″), aac(6′)-Ib9*), bacitracin (*bacA*), class A (*cfxA6*) and class D beta-lactamases (*bla*_OXA_), chloramphenicol (*cat*), MLS (*cfr*), polymyxin (*arnA*), trimethoprim (*dfrAE*) and multidrug resistance efflux pumps (*crp, phoP, emrB, mdfA, mdtH, adeC-adeK-oprM, baeRS, msbA, adeGB, mexEFB, abeM, oprN, evgS*) (Supplementary Figure S1 and Table S3B). The treated effluents showed the least similarity with 9 and 1 ARG subtype (*bla*_OXA-198_) shared between the wastewater and surface waters (Supplementary Figure S1 and Table S3C). The wastewater and treated effluents had common genes encoding for resistance to aminoglycosides [p*aph(6)-Id*], multidrug resistance efflux pumps (ABC antibiotic efflux pump), MLS (*ermB*), beta-lactamases (*bla*_V EB-1a_, *bla*_OXA-347_), vancomycin (*vanX*), tetracycline (*tetO*), streptogramin (*vatB*) and sulfonamide (*sul3*) (Supplementary Table S3D).

### Dominant ARG Subtypes across All Samples

**FIGURE 2:**
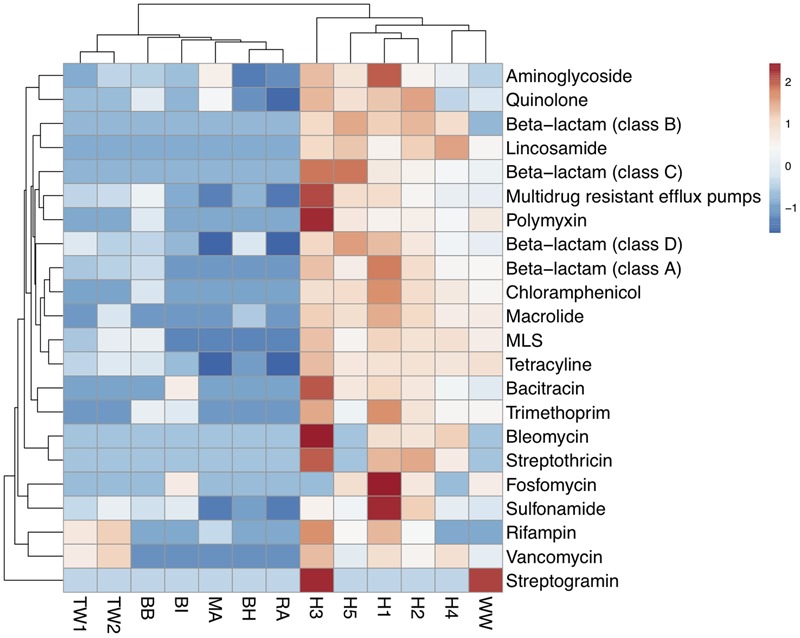
Heat map of the abundance of ARG-like ORFs assigned to ARG types across wastewaters (WW, H1-5), treated effluents (TW1, 2) and surface waters (BI, BH, BB, RA, MA). Abundance values were transformed using log (x+1). Clustering based on euclidean distances showed two distinct groupings of the antibiotic resistomes of the wastewater samples, the treated effluents, and environmental waters.

The abundance of each of the 22 ARG types detected was calculated based on summing the coverage of ARG subtypes belonging to the same ARG type (Supplementary Table S4). A cluster analysis showed that wastewater samples had a higher similarity in antibiotic resistance profiles forming one cluster, while the treated effluent and surface water formed another (**Figure 2**). The wastewater cluster had a higher average abundance of ARGs (197,816 x/Gb) compared to the treated effluent (2,692 x/Gb) and surface water (7,985 x/Gb) clusters. Multidrug resistant efflux pumps, class A beta-lactamase and aminoglycoside resistance genes were the most abundant ARG types in the wastewater cluster, accounting for an average of 77,056, 53,034 and 24,524 x/Gb, respectively, while the treated effluent and surface water cluster was more abundant in aminoglycoside (2,626 x/Gb), multidrug resistant efflux pumps (1,857 x/Gb) and quinolone resistant genes (1,523 x/Gb).

The most abundant gene, *bla*_KPC-2_ (277,258 x/Gb) was detected in hospital sample H1 and at lower levels in hospital sample H2 (7,141 x/Gb) (Supplementary Table S5). Of the top 3 most abundant ARGs detected in each sample, two of the most commonly detected ARG was a quinolone resistant gene (fluoroquinolone resistant DNA topoisomerase) which was identified across 7 samples [H2 (11,966 x/Gb), TW1 (383 x/Gb), BH (464 x/Gb), BI (836 x/Gb), BB (2,887 x/Gb), MA (5,446 x/Gb), RA (232 x/Gb)] and aminoglycoside resistant gene *aph(3″)* gene was identified across six samples [H1 (87,964 x/Gb), TW2 (637 x/Gb), BI (1,025 x/Gb), BB (1,302 x/Gb), MA (14,200 x/Gb), RA (371 x/Gb), Supplementary Figure S2 and Table S3A]. These two genes were detected in all samples but at varying abundances (Supplementary Table S3A). Among these ARGs, other subtypes belonging to multidrug efflux pumps, aminoglycosidase, quinolone and beta-lactam resistant genes were also abundant in the hospital wastewater samples (Supplementary Figure S2).

The municipal wastewater was dominated by tetracycline resistance (*tet* ribosomal protein), class A beta-lactamase (*cfxA6*) and macrolide resistance genes (*ereA*), and treated wastewater consistently with vancomycin resistant genes (*vanX*) (Supplementary Figure S2).

### Occurrence of β-Lactam Resistance Genes

Beta-lactamase genes in wastewater represented between 8 and 54% of total abundance of ARGs in wastewater samples, which was much higher compared to treated effluents (1–3%) and surface waters (0–8%). The clinically important β-lactamase gene variants identified in all samples is found in Supplementary Table S5. The *bla*_OXA_ genes were the most ubiquitous and detected in all samples except for surface waters MA and RA (Supplementary Figure S3 and Table S5). The highest abundance of *bla*_OXA_ genes was mainly in hospital wastewaters from H5 (7,094 x/Gb) and H1 (3,541 x/Gb, Supplementary Figure S3). For class C, the *AmpC* beta lactamase gene was only detected in wastewater samples at particularly high abundance in H5 (25,995 x/Gb) and H3 (23,568 x/Gb, Supplementary Figure S3). Class B beta-lactamases which consisted of *bla*_IMP_, *bla*_NDM_ and *bla*_V IM_ were present in lower abundance and only in hospital wastewater samples (Supplementary Figure S3 and Table S5).

We detected *bla*_KPC-2_ and *bla*_NDM-1_, *bla*_NDM-2_, and *bla*_NDM-3_ in hospital discharge samples only (Supplementary Table S5). The *bla*_KPC-2_ gene was the most abundant ARG in hospital discharge samples, H1 (277,258 x/Gb), and the second most abundant ARG in H2 (7,141 x/Gb, Supplementary Figure S3 and Table S5). The *bla*_NDM_ (metallo-beta-lactamase, class B) genes were less abundant (8-19 x/Gb) but detected in 4 out of the 5 hospital wastewater samples (Supplementary Figure S3 and Table S5). Other metallo-beta-lactamase class B genes, *bla*_V IM_ (6-56 x/Gb) and *bla*_IMP_ (12-142 x/Gb) were detected in all hospital wastewater samples at higher abundance than *bla*_NDM_ genes. These included *bla*_V IM-2,3_, *bla*_V IM-11,12_, *bla*_V IM-18_, *bla*_V IM-26_, *bla*_V IM-30_, and *bla*_IMP-1_, *bla*_IMP-4_ *bla*_IMP-6_, *bla*_IMP-42._

### Hospital Wastewater Bacteria Bearing Dominant *bla*_KPC_ Genes

**FIGURE 3:**
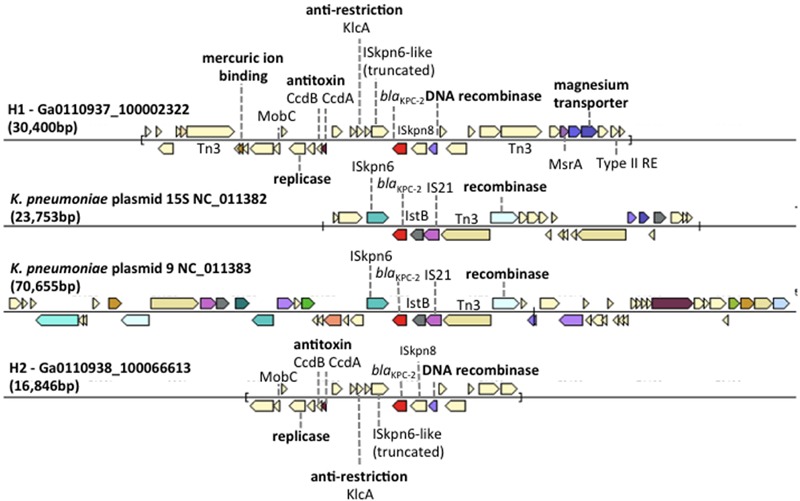
Comparison of gene neighborhood in other genomes containing the *bla*_KPC-2_ gene in H1, *Klebsiella pneumoniae* plasmid 15S (NC_011382), *K. pneumoniae* plasmid 9 (NC_011383), and H2. Genes of similar colors belong to the same orthologous groups. The plasmid borne Tn4410 elements were labeled on *K. pneumoniae* plasmid 15S (NC_011382) and *K. pneumoniae* plasmid 9 (NC_011383).

The scaffold carrying the *bla*_KPC-2_ gene in H1 (Ga0110937_ 100002322, 30,400 bp) and H2 (Ga0110938_100066613, 16,846 bp) samples were compared to *Klebsiella pneumoniae* plasmid sequences in the IMG database (**Figure 3**). The *bla*_KPC-2_ gene sequence and gene context had the best match to the *K. pneumoniae* plasmid 15S (NC_011382) and *K. pneumoniae* plasmid 9 (NC_011383) in the IMG database, both of which were isolated from *Klebsiella* strains from a hospital in New York. The genetic contexts of *bla*_KPC-2_ gene on the H1 and H2 scaffolds were identical suggesting that this scaffold is a partial plasmid sequence that may be responsible for the high genotypic resistance toward carbapenem antibiotics observed in the hospital wastewater samples. On the H1 and H2 scaffolds, genes encoding plasmid mobility (MobB), replication, antitoxin proteins (CcdA/B), anti-restriction encoded protein (KlcA) and a site-specific DNA recombinase site flanking the *bla*_KPC-2_ gene, were present. The slightly longer H1 scaffold possessed magnesium, mercuric ion binding genes. These genes likely play a role in mechanisms that allow the genetic modification of plasmids for increased fitness, metal/antibiotic resistance, and MGE acquisition for host propagation.

To verify species of bacteria carrying the *bla*_KPC_ gene, bacteria resistant to carbapenems (KPC) and extended-spectrum β-lactams (ESBL) were isolated from hospital wastewater samples. Bacterial strains belonging to *K. pneumoniae, Enterobacter cloacae, Pseudomonas aeruginosa*, and *Citrobacter freundii* that carried the *bla*_KPC-2_ gene had 100% sequence identity to the *bla*_KPC-2_ gene identified in the H1 and H2 samples (**Table 2**). These taxa of bacteria are candidates that may harbor the *bla*_KPC-2_ bearing plasmid.

**Table 2:** Cultured bacterial isolates bearing the *bla*_KPC-2_ gene.

Sample	Isolate number	Media	Bacterial species
H1	H2	ESBL	*Klebsiella pneumoniae*
H1	H6	KPC	*Enterobacter cloacae*
H1	C20	OR	*Pseudomonas aeruginosa*
H2	H42	KPC	*Citrobacter freundii*

*Abbreviations are as follows: CHROMagar orientation (OR), and CHROMagar supplemented with (ESBL) and (KPC).*

### Plasmids Associated with Antimicrobial Resistant *Enterobacteriaceae*

**FIGURE 4:**
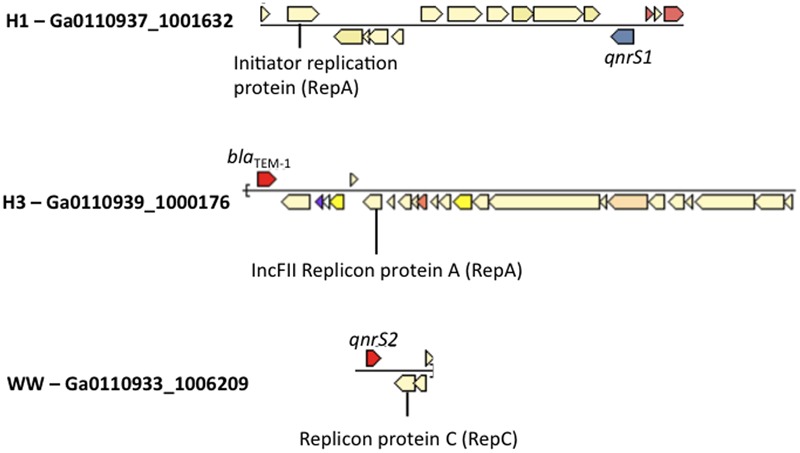
Co-occurrence of *Enterobacteriaceae* plasmid replicons and ARGs in metagenomic scaffolds in H1, H3 and WW samples.

All wastewater and surface water sample BB had at least one contig matching replicon sequences of IncQ2 plasmid in Plasmidfinder (Supplementary Table S6). Plasmidfinder identified a total of 72 plasmids, in the assembled metagenomes; however, only 3 scaffolds were found to carry ARGs. This included the class A beta lactamase gene, *bla*_TEM-1_ (Ga0110939_10001761) in H3, and *qnrS1* genes in H1 (Ga0110937_100163213), and *qnrS2* in WW (Ga0110933_12248201) within the same scaffold as plasmid replicons (**Figure 4**).

## Discussion

The main findings of this study show that wastewater, particularly hospital discharge, are primary hotspots for AMR, and carry a high diversity and abundance of ARGs compared to surface waters and treated effluents. This is consistent with a recent *in silico* survey of ARGs from different ecological niches by Fitzpatrick and Walsh (2016) whereby the highest ARG diversity was described in the human gut microbiome, followed by hospital WWTP and non-hospital WWTP and environmental waters. Pal et al. (2016) investigated the structure and diversity of human, animal, and environmental resistomes and found a similar trend. The wastewater cluster was enriched with commensal human gut microbiota (e.g., *Bacteroides, Faecalibacterium, Bifidobacterium, Blautia, Roseburia, Ruminococcus*) that is likely derived from the fecal shedding carrying traces of bacteria from the gastrointestinal tract (Lozupone et al., 2012; Pal et al., 2016; Pop et al., 2016). A study of multidrug resistant- and ESBL producing bacteria in Brazilian hospital effluent described resistant species belonging to *Klebsiella, Aeromonas, Enterobacter, Escherichia coli* (Chagas et al., 2016). The enrichment of the same *Enterobacteriaceae* (*Klebsiella, Aeromonas, Enterobacter*) taxa in the wastewaters cluster suggests that members within the community are potential carriers and a source of clinically important ARGs.

The ARG types detected in the core resistome have been reported in studies across environmental waters (sediments, soil, ocean), drinking water, and the influent and effluents of WWTP (Nesme et al., 2014; Li B. et al., 2015). The aminoglycoside resistant gene *aph(3″)* which was detected in all samples in this study, and was the top 3 most abundant ARG in 6 of the 13 samples appears to be widespread in different environments with variants of this same gene [*aph(3″)-IIa, aph(3′)-Ib*] described in other environmental datasets of similar nature to the ones in this study (Pal et al., 2016). The wastewater resistome had more ARGs in common with surface waters than treated effluent. The shared resistome between wastewater and surface waters were composed mainly of multidrug resistant efflux pumps, and genes resistant to aminoglycosides, bacitracin, beta-lactams (beta-lactamase class A and D), chloramphenicol, MLS, polymyxin and trimethoprim. In a recent study of ARGs of surface waters in Singapore, genes associated with a few these ARG types (multidrug resistant efflux pumps, beta-lactamase A–D) were detected in using the GeoChip (Low et al., 2016). Among the beta-lactamases detected in the wastewater, two genes which confer resistance to cephalosporins; class A beta-lactamase gene *cfxA6*, a gene commonly found in the human gut microbiome (Hu et al., 2013) and class D beta-lactamase gene *bla*_OXA_ which are plasmid mediated and associated with species of *Acinetobacter* (Evans and Amyes, 2014) were found in surface waters. Although the microbial community composition of surface waters have a relatively low representation of taxa (i.e., gut bacteria or opportunistic pathogens) which potentially carry these genes, traces of non-point source contamination coming from surface runoffs in the surrounding urban areas may facilitate contribute to beta-lactamases observed in the surface water resistomes.

Wastewaters had the highest diversity and abundance of clinically important β-lactam resistance genes. Some of these beta-lactamases confer resistance to ESBLs and carbapenems, which is a last resort antibiotic used in treating Gram-negative infections. The first KPC producing *K. pneumoniae* was identified in 2001 in North Carolina, which has since spread globally (Nordmann et al., 2011; Yigit et al., 2011). KPC producing *K. pneumonia* arrived in Asia in 2004 spreading from China to South Korea and Taiwan. In 2012, cases emerged in Singapore where two of four patients were found to harbor the China related strains. The detection in two other persons of non-Chinese origin with had no travel history suggesting possible community dissemination (Balm et al., 2012; Ling et al., 2015). National surveillance of AMR and antibiotic prescription has shown a dramatic increase in the occurrence of cephalosporin and carbapenem resistant *Enterobactericeae* (CRE) in local hospitals over the years (Hsu et al., 2007, 2010; Liew et al., 2011; Ng et al., 2014; Venkatachalam et al., 2014; Young et al., 2014). The CRE trend obtained from rectal screening of inpatients at local government hospitals showed a positive CRE increase from an average of 16.0 per 100,000 patient-days from 2013 to 2015, in which *bla*_KPC_ was the predominant carbapenemase gene detected (Marimuthu et al., 2017). Hence, it is not surprising that a strikingly high abundance of carbapenemase gene *bla*_KPC-2_ was detected in hospital discharge from clinical isolation wards. Furthermore, isolates of *Enterobacteriaceae* (i.e., *K. pneumonia, E. cloacae*, and *C. freundii*) and *P. aeruginosa* harboring the *bla*_KPC-2_ gene were cultured from the same hospital wastewater samples indicating that this gene was present in a variety of different species.

Two scaffolds, assembled from the clinical isolation wastewater samples, carried the abundant *bla*_KPC-2_ gene on a plasmid borne *Tn4410* like element. Other metal resistant genes, (mercuric, magnesium) and a toxin–antitoxin (TA) system encoded genes (*ccdB, ccdA*) were also detected on the scaffold. Plasmid-based TA systems are involved in post-segregation killing or growth inhibition of daughter cells that do not inherit a plasmid copy during cell division (Hayes, 2003). The TA system is a form of bacterial persistence, a phenotype of dormant cells present at a low frequency in a growing population and characterized by tolerance to the presence of a variety of antibiotics (Balaban et al., 2004; Lewis, 2010). The occurrence of the TA system on a plasmid bearing the *bla*_KPC-2_ gene suggests that these functional genes are important mechanisms for the selection and persistence of KPC resistant phenotype in the hospital wastewaters.

The first case of NDM-1 was described in 2009 when a Swedish patient of Indian origin who traveled to New Delhi contracted a urinary tract infection resulting from NDM-1 producing *K. pneumoniae* (Yong et al., 2009). The first local cases of NDM-1 was identified in 2010 in an Indian and Bangladeshi national, and since then there has been a progressive increase of locally transmitted cases (Chien et al., 2012) mediated by *bla*_NDM_ bearing plasmids in *K. pneumoniae* and *E. coli* (Khong et al., 2016). In our study, a range of other carbapenemases including class B metallo-beta lactamases (*bla*_NDM_, *bla*_IMP_, *bla*_V IM_), were detected at lower abundance than *bla*_KPC-2_. The microbial community structure of wastewater was enriched in the *Enterobacteriaceae* group (*Klebsiella, Enterobacter*) and other opportunistic pathogens (*Comamonas*), which are the main taxa found carrying *bla*- genes as described in our previous study of hospital wastewaters (Le et al., 2016). The overrepresentation of these taxa in wastewaters offers an explanation for the high prevalence of beta-lactam resistance genes in the wastewater cluster. Fecal waste coming from patients colonized with ESBL and carbapenem resistant bacteria likely contribute to the high abundance of genes encoding ESBL (*bla*_CTX_, *bla*_TEM_, *bla*_OXA_, *bla*_SHV_) and carbapenem (*bla*_KPC_, *bla*_NDM_) resistance genes observed in hospital wastewaters. Our dataset also shows that community sewage may not have as large of an impact on the spread on ESBL and carbapenem genes given that these genes were present at lower abundance in municipal wastewaters, relative to hospital wastewater.

Partial plasmids sequences were assembled from wastewater datasets, one of which carried the *bla*_TEM-1_ gene (H3), and the two others quinolone resistance genes *qnrS1* (H1) and *qnrS2* (WW). IncQ plasmids bearing *qnrS2* genes are highly mobile and have been isolated in bacterial communities of WWTP elsewhere (Bonemann et al., 2005). The presence of these MGEs carrying quinolone resistance genes in local municipal wastewaters suggests the potential genetic exchange of quinolone resistant genes between bacterial species in community sewage. Although we were unable to detected ARGs on the IncQ2 plasmid in surface waters, which was perhaps due to low sequence coverage, hence poor scaffold assemblies, the detection of an IncQ2 plasmid in one of the surface water samples (BB) supports the notion that these plasmids may play a disseminating role of quinolone resistance in surface waters.

Wastewater treatment plants represent another significant hotspot for ARG transfer facilitated by high cell densities, and the mixing of sub-inhibitory concentrations of antibiotics (Rizzo et al., 2013; Fitzpatrick and Walsh, 2016). A review of urban WWTPs concluded that a large diversity of ARGs conferring resistance to almost all mechanisms of antibiotic resistance are capable of surviving the wastewater treatment process (Rizzo et al., 2013). The treatment process has been shown to induce the abundance of tetracycline (*tet* genes), sulfonamide (*sul* genes), quinolone (*gyr, qnr, par* genes), beta-lactam (*bla*_V IM_, *ampC* genes), vancomycin (*vanA*), ARGs (Alexander et al., 2015; Li J. et al., 2015; Mao et al., 2015; Xu J. et al., 2015). We were unable establish the selective enrichment of specific ARGs in treated effluent due to insufficient coverage of low abundance ARGs in the untreated waters. However, within the context of our study the detection of a few ARGs in shared resistome of wastewater and treated effluents suggested incomplete removal of certain ARGs after the secondary treatment process. This included genes resistant to aminoglycosides [*aph(6)-ld*], multidrug efflux pumps (ABC efflux pumps), MLS (*ermB*), sulfonamide (*sul3*), tetracycline (*tetO*), beta-lactam (*bla*_V EB-1a_, *bla*_OXA-347_), vancomycin (*vanX*) and streptogramin (*vatB*).

## Conclusion

The metagenomic approach used in this study has unveiled a vast array of ARGs within different ecological niches enabling a comparative analysis of resistomes to track of AMR dissemination patterns in Singapore. Although ARGs are found almost everywhere, all environments do not pose the same risk (Martinez et al., 2015). It is proposed that environments with higher abundance and diversity of ARGs are likely involved with higher probability of transfer due to increase chances of for potential donor strains to physically interact with suitable recipients (Bengtsson-Palme and Larsson, 2015). We conclude that wastewater habitats, particularly hospital wastewaters contain high loads of opportunistic pathogens, and higher diversity and abundance clinically important ESBL and carbapenem resistant genes. The detection of plasmid-encoded *bla*_KPC_ in other bacterial species in local hospital wastewaters is evidence that it is highly transmissible to other bacteria. Countries including France, Portugal, Brazil, China are facing increased frequency of the recovery of KPC producing *Enterobacteriaceae* in environmental rivers (Woodford et al., 2014; Xu G. et al., 2015). This raises questions as to whether hospitals should develop waste management strategies and invests in pre-treatment membrane technology (ultra- or nanofiltration) prior to discharging hospital wastewaters into public sewers. This together with antibiotic stewardship programs could help reduce the propagation and dissemination of antibiotic resistance beyond the hospital setting (Hocquet et al., 2016). Efforts to monitor WWTP and surface waters should continue to gain better perspectives into dissemination within the general community.

## Author Contributions

CN wrote the manuscript and TK and TB provided clinical wastewater samples. CN, T-HL, LH, and HC conducted sampling and performed the experiments. CN, MT, and BT analyzed datasets. JT provided computational resources and supervision for data analysis. CN, MT, BT, and KG conceived and designed the experiments.

## Conflict of Interest Statement

The authors declare that the research was conducted in the absence of any commercial or financial relationships that could be construed as a potential conflict of interest.

## Footnotes

1 https://card.mcmaster.ca/

2 http://www.dantaslab.org/resfams/

3 https://cge.cbs.dtu.dk/services/PlasmidFinder/

4 https://img.jgi.doe.gov/cgi-bin/mer/main.cgi

5 https://cge.cbs.dtu.dk/services/PlasmidFinder/

6 https://card.mcmaster.ca/

7 http://www.dantaslab.org/resfams/

8 https://ab.inf.uni-tuebingen.de/software/megan5
