# Corrigendum: Characterization of Metagenomes in Urban Aquatic Compartments Reveals High Prevalence of Clinically Relevant Antibiotic Resistance Genes in Wastewaters

**DOI:** 10.3389/fmicb.2018.00175

**Published:** 2018-02-05

**Authors:** Charmaine Ng, Martin Tay, Boonfei Tan, Thai-Hoang Le, Laurence Haller, Hongjie Chen, Tse H. Koh, Timothy M. S. Barkham, Janelle R. Thompson, Karina Y.-H. Gin

**Affiliations:** ^1^Department of Civil and Environmental Engineering, National University of Singapore, Singapore, Singapore; ^2^Centre for Environmental Sensing and Modeling, Singapore-MIT Alliance for Research and Technology Centre, Singapore, Singapore; ^3^Department of Environmental Engineering, Ho Chi Minh City International University, Ho Chi Minh City, Vietnam; ^4^Department of Pathology, Singapore General Hospital, Singapore, Singapore; ^5^Department of Laboratory Medicine, Tan Tock Seng Hospital, Singapore, Singapore; ^6^NUS Environmental Research Institute, National University of Singapore, Singapore, Singapore

**Keywords:** comparative metagenomics, antibiotic resistant genes, wastewaters, hospital, municipal, water body, tributary, beta lactamase resistant genes

Janelle R. Thompson was not included as an author in the published article. The authors apologize for this error and state that this does not change the scientific conclusions of the article in any way.

The original article has been updated.

## Author contributions

CN wrote the manuscript and TK and TB provided clinical wastewater samples. CN, T-HL, LH, and HC conducted sampling and performed the experiments. CN, MT, and BT analyzed datasets. JT provided computational resources and supervision for data analysis. CN, MT, BT, and KG conceived and designed the experiments.

### Conflict of interest statement

The authors declare that the research was conducted in the absence of any commercial or financial relationships that could be construed as a potential conflict of interest.

