# Supplementary material for: Characterization of Metagenomes in Urban Aquatic Compartments Reveals High Prevalence of Clinically Relevant Antibiotic Resistance Genes in Wastewaters
Source: Front Microbiol. 2017 Nov 16;8:2200. doi: 10.3389/fmicb.2017.02200 (PMC5696577; doi:10.3389/fmicb.2017.02200)
Supplement: Supplementary file 1 [file Data_Sheet_1.DOC]

Table S1. Metagenomic datasets.

| Sample ID | Trimmed read pairs | Total no. of ORFs | No. of ARG-like ORFs | Total percentage of ARG-like ORFs (%) | Size of metagenome (MB) | No. of unique ARG subtypes |
| --- | --- | --- | --- | --- | --- | --- |
| H1 | 3,202,560 | 329,801 | 512 | 0.16 | 144 | 188 |
| H2 | 2,697,598 | 418,086 | 416 | 0.1 | 183 | 164 |
| H3 | 3,270,686 | 447,152 | 488 | 0.11 | 201 | 191 |
| H4 | 2,694,623 | 415,717 | 327 | 0.09 | 187 | 133 |
| H5 | 2,937,862 | 349,143 | 415 | 0.12 | 156 | 164 |
| WW | 2,669,915 | 436,195 | 308 | 0.07 | 172 | 121 |
| TW1 | 1,928,883 | 326,339 | 48 | 0.01 | 123 | 22 |
| TW2 | 2,190,396 | 344,840 | 51 | 0.01 | 136 | 27 |
| BH | 2,476,019 | 298,008 | 42 | 0.01 | 108 | 16 |
| BI | 2,164,405 | 261,533 | 40 | 0.02 | 101 | 15 |
| BB | 2,353,560 | 304,046 | 168 | 0.05 | 118 | 51 |
| MA | 2,400,624 | 300,778 | 40 | 0.01 | 137 | 4 |
| RA | 2,530,686 | 302,742 | 23 | 0.01 | 123 | 6 |

Table S2. No. of ARG subtypes identified across samples.

| ARG Type | H1 | H2 | H3 | H4 | H5 | WW | TW1 | TW2 | BH | BI | BB | MA | RA |
| --- | --- | --- | --- | --- | --- | --- | --- | --- | --- | --- | --- | --- | --- |
| Aminoglycoside | 40 | 36 | 43 | 37 | 35 | 26 | 9 | 9 | 7 | 12 | 17 | 11 | 8 |
| Bacitracin | 3 | 7 | 4 | 2 | 2 | 2 | 0 | 0 | 0 | 1 | 0 | 0 | 0 |
| Beta-lactam (Class A) | 18 | 17 | 18 | 16 | 13 | 11 | 1 | 1 | 0 | 0 | 2 | 0 | 0 |
| Beta-lactam (Class B) | 5 | 7 | 4 | 2 | 5 | 0 | 0 | 0 | 0 | 0 | 0 | 0 | 0 |
| Beta-lactam (Class C) | 5 | 4 | 5 | 3 | 7 | 2 | 0 | 0 | 0 | 0 | 1 | 0 | 0 |
| Beta-lactam (Class D) | 16 | 12 | 14 | 9 | 13 | 8 | 4 | 1 | 4 | 1 | 2 | 0 | 0 |
| Bleomycin | 1 | 1 | 2 | 1 | 0 | 0 | 0 | 0 | 0 | 0 | 0 | 0 | 0 |
| Chloramphenicol | 13 | 13 | 26 | 17 | 15 | 9 | 0 | 0 | 0 | 0 | 1 | 0 | 0 |
| Multidrug resistance efflux pumps | 296 | 212 | 251 | 150 | 232 | 156 | 17 | 14 | 21 | 11 | 95 | 8 | 8 |
| Fosfomycin | 4 | 1 | 0 | 0 | 1 | 2 | 0 | 0 | 0 | 1 | 0 | 0 | 0 |
| Lincosamide | 3 | 4 | 3 | 1 | 4 | 3 | 0 | 0 | 0 | 0 | 0 | 0 | 0 |
| Macrolide | 3 | 4 | 6 | 7 | 5 | 4 | 0 | 1 | 1 | 0 | 0 | 0 | 0 |
| MLSB | 8 | 6 | 4 | 7 | 7 | 5 | 1 | 2 | 0 | 0 | 5 | 0 | 0 |
| Polymyxin | 4 | 4 | 2 | 2 | 5 | 4 | 0 | 0 | 0 | 0 | 1 | 0 | 0 |
| Quinolone | 40 | 47 | 43 | 30 | 42 | 42 | 10 | 11 | 7 | 11 | 31 | 20 | 7 |
| Rifampicin | 1 | 1 | 3 | 0 | 1 | 0 | 1 | 1 | 0 | 0 | 0 | 1 | 0 |
| Streptogramin | 0 | 0 | 1 | 0 | 0 | 1 | 0 | 0 | 0 | 0 | 0 | 0 | 0 |
| Streptothricin | 2 | 1 | 2 | 1 | 0 | 0 | 0 | 0 | 0 | 0 | 0 | 0 | 0 |
| Sulfonamide | 2 | 2 | 2 | 2 | 3 | 2 | 2 | 3 | 1 | 1 | 2 | 0 | 0 |
| Tetracycline | 28 | 27 | 43 | 23 | 20 | 20 | 2 | 6 | 1 | 1 | 8 | 0 | 0 |
| Trimethoprim | 6 | 4 | 7 | 5 | 4 | 3 | 0 | 0 | 0 | 1 | 3 | 0 | 0 |
| Vancomycin | 14 | 6 | 24 | 12 | 1 | 1 | 1 | 2 | 0 | 0 | 0 | 0 | 0 |
| Total | 512 | 416 | 507 | 327 | 415 | 301 | 48 | 51 | 42 | 40 | 168 | 40 | 23 |

Table S3A. Abundance of the shared/core resistome between wastewater, treated effluent and surface waters.

| ARG Type | ARG subtype | H1 | H2 | H3 | H4 | H5 | WW | TW1 | TW2 | BH | BI | BB | MA | RA |
| --- | --- | --- | --- | --- | --- | --- | --- | --- | --- | --- | --- | --- | --- | --- |
| Aminoglycoside | *ant(3)* | 3009.31 | 162.52 | 221.58 | 0.00 | 250.47 | 61.28 | 9.68 | 17.15 | 0.00 | 27.00 | 80.31 | 0.00 | 0.00 |
|  | *ant(3")-Ia* | 351.08 | 0.00 | 342.11 | 24.10 | 354.90 | 27.94 | 0.00 | 26.66 | 14.53 | 24.32 | 13.97 | 0.00 | 0.00 |
|  | *aph(3")* | 87964.96 | 2549.26 | 23010.08 | 886.09 | 7409.38 | 380.48 | 72.51 | 637.48 | 237.69 | 1024.90 | 1302.19 | 14200.09 | 371.57 |
|  | *aph(6)* | 4335.52 | 289.01 | 75.96 | 67.96 | 602.96 | 18.09 | 0.00 | 15.38 | 0.00 | 20.70 | 46.08 | 0.00 | 0.00 |
| Multidrug resistance efflux pumps | *adeJ* | 15.78 | 9.83 | 10.98 | 0.00 | 10.61 | 613.57 | 0.00 | 21.12 | 0.00 | 0.00 | 19.54 | 0.00 | 0.00 |
|  | *mdtB* | 508.66 | 100.64 | 1660.52 | 51.27 | 104.03 | 39.68 | 65.49 | 11.55 | 110.51 | 133.01 | 17.18 | 0.00 | 22.04 |
|  | *pmrE* | 240.61 | 26.76 | 5223.28 | 43.69 | 70.43 | 179.22 | 10.52 | 0.00 | 0.00 | 58.07 | 71.61 | 0.00 | 0.00 |
|  | *msrE* | 168.30 | 192.51 | 73.18 | 127.24 | 99.67 | 33.49 | 36.56 | 0.00 | 0.00 | 0.00 | 18.82 | 0.00 | 0.00 |
|  | *mexK* | 267.89 | 0.00 | 53.14 | 90.72 | 130.93 | 0.00 | 27.25 | 0.00 | 114.64 | 217.07 | 6171.29 | 0.00 | 25.09 |
|  | *mexT* | 87.43 | 48.68 | 38.87 | 7.98 | 64.38 | 28.99 | 39.50 | 36.95 | 0.00 | 0.00 | 223.95 | 0.00 | 0.00 |
|  | *macB* | 104.75 | 181.55 | 292.18 | 174.12 | 79.20 | 123.31 | 21.87 | 21.16 | 0.00 | 0.00 | 205.40 | 0.00 | 0.00 |
|  | RND Antibiotic Efflux Pump | 411.86 | 253.98 | 678.65 | 586.90 | 1097.33 | 249.85 | 26.37 | 162.07 | 530.16 | 238.84 | 702.34 | 232.64 | 144.49 |
| MLS | *ermF* | 175.10 | 27.30 | 1068.56 | 52.30 | 21.97 | 26.45 | 5.65 | 0.00 | 0.00 | 0.00 | 43.07 | 0.00 | 0.00 |
| Macrolide | *ereA* | 45.31 | 41.94 | 148.48 | 69.69 | 163.72 | 135.84 | 0.00 | 12.12 | 9.23 | 0.00 | 0.00 | 0.00 | 0.00 |
| Quinolone | *qnrS2* | 898.22 | 268.45 | 0.00 | 0.00 | 0.00 | 61.31 | 0.00 | 12.54 | 0.00 | 0.00 | 9.44 | 0.00 | 0.00 |
|  | Resistant DNA Topoisomerase | 2485.13 | 11966.26 | 9677.69 | 613.71 | 3066.27 | 830.01 | 383.25 | 292.43 | 464.12 | 836.90 | 2887.69 | 5446.78 | 232.01 |
| Sulfonamide | *sul1* | 1757.97 | 1300.52 | 164.15 | 35.35 | 129.16 | 38.58 | 9.71 | 6.40 | 5.40 | 156.03 | 43.74 | 0.00 | 0.00 |
|  | *sul2* | 79709.58 | 894.46 | 190.29 | 42.46 | 9.59 | 5.16 | 9.76 | 52.81 | 0.00 | 0.00 | 17.18 | 0.00 | 0.00 |
| Tetracycline | Tet MFS Efflux Pump | 560.98 | 695.66 | 2710.09 | 351.55 | 604.75 | 146.00 | 0.00 | 31.98 | 0.00 | 32.47 | 53.61 | 0.00 | 0.00 |
|  | Tet Ribosomal Protection Protein | 604.32 | 536.45 | 1381.04 | 560.53 | 202.77 | 1204.78 | 20.79 | 44.53 | 0.00 | 0.00 | 82.73 | 0.00 | 0.00 |
|  | *tetX* | 132.72 | 73.76 | 56.72 | 4.69 | 15.15 | 17.53 | 6.99 | 0.00 | 10.20 | 0.00 | 0.00 | 0.00 | 0.00 |

Table S3B. Abundance of the shared resistome between wastewater and surface waters.

| ARG Type | ARG subtype | H1 | H2 | H3 | H4 | H5 | WW | BH | BI | BB | MA | RA |
| --- | --- | --- | --- | --- | --- | --- | --- | --- | --- | --- | --- | --- |
| Aminoglycoside | *aph(3")* | 60.97 | 29.36 | 212.82 | 168.39 | 21.79 | 15.64 | 0.00 | 0.00 | 46.08 | 0.00 | 0.00 |
|  | *aac(6')-Ib9* | 0.00 | 63.85 | 0.00 | 208.19 | 113.89 | 28.82 | 0.00 | 0.00 | 10.65 | 0.00 | 0.00 |
| Bacitracin | *bacA* | 83.57 | 214.62 | 13979.89 | 53.96 | 240.71 | 19.49 | 0.00 | 400.56 | 0.00 | 0.00 | 0.00 |
| Beta lactam (Class A) | *cfxA6* | 143.30 | 218.70 | 93.77 | 31.92 | 11.25 | 347.03 | 0.00 | 0.00 | 39.21 | 0.00 | 0.00 |
| Beta lactam (Class D) | *bla*_OXA_ | 0.00 | 0.00 | 13.53 | 0.00 | 115.64 |  | 70.31 | 0.00 | 0.00 | 0.00 | 0.00 |
| Beta lactam (Class D) | *Beta-lactam Class D* | 0.00 | 0.00 | 10247.85 | 498.81 | 48.85 | 57.28 | 25.07 | 0.00 | 0.00 | 0.00 | 0.00 |
| Chloramphenicol | *cat* | 3341.59 | 441.69 | 201.73 | 160.74 | 305.82 | 87.67 | 0.00 | 0.00 | 30.06 | 0.00 | 0.00 |
| MLS | *cfr* | 46.21 | 34.80 | 95.75 | 56.46 | 28.73 | 66.88 | 0.00 | 0.00 | 80.80 | 0.00 | 0.00 |
| Mutidrug resistance efflux pump | *crp* | 10738.37 | 1666.81 | 37407.68 | 71.67 | 18683.50 | 108.16 | 0.00 | 0.00 | 59.00 | 0.00 | 0.00 |
|  | *phoP* | 300.81 | 57.70 | 1803.18 | 15.21 | 112.52 | 57.37 | 0.00 | 0.00 | 40.01 | 0.00 | 0.00 |
|  | *emrB* | 508.39 | 62.58 | 2235.17 | 126.18 | 83.25 | 104.70 | 0.00 | 0.00 | 32.84 | 0.00 | 0.00 |
|  | *baeR* | 348.08 | 66.72 | 27440.15 | 39.79 | 40.59 | 52.76 | 42.91 | 0.00 | 145.45 | 0.00 | 0.00 |
|  | *mdfA* | 310.41 | 260.86 | 5563.04 | 0.00 | 83.56 | 14.79 | 0.00 | 0.00 | 15.65 | 0.00 | 0.00 |
|  | *mdtH* | 204.30 | 21.32 | 2154.64 | 39.52 | 146.01 | 114.26 | 0.00 | 0.00 | 13.01 | 0.00 | 0.00 |
|  | *adeC-adeK-oprM* | 143.71 | 145.28 | 58.76 | 27.65 | 819.89 | 31.26 | 0.00 | 0.00 | 101.88 | 0.00 | 0.00 |
|  | *baeS* | 64.30 | 58.34 | 3095.95 | 15.88 | 128.01 | 19.46 | 0.00 | 0.00 | 12.00 | 0.00 | 0.00 |
|  | *msbA* | 77.02 | 109.38 | 131.57 | 58.45 | 32.70 | 10.71 | 0.00 | 0.00 | 284.56 | 0.00 | 0.00 |
|  | *adeG* | 94.80 | 0.00 | 0.00 | 43.14 | 57.99 |  | 0.00 | 0.00 | 60.82 | 0.00 | 0.00 |
|  | *adeB* | 195.78 | 149.50 | 387.81 | 148.07 | 300.33 | 42.87 | 0.00 | 0.00 | 145.41 | 0.00 | 0.00 |
|  | *mexE* | 28.05 | 0.00 | 16.65 | 8.29 | 0.00 | 0.00 | 0.00 | 0.00 | 111.24 | 0.00 | 0.00 |
|  | *mexF* | 75.26 | 17.58 | 50.27 | 0.00 | 0.00 | 55.31 | 34.93 | 49.14 | 65.30 | 0.00 | 0.00 |
|  | *mexB* | 19.48 | 0.00 | 262.29 | 7.01 | 0.00 | 27.00 | 0.00 | 0.00 | 515.28 | 0.00 | 0.00 |
|  | *abeM* | 0.00 | 61.09 | 74.43 | 41.15 | 281.71 | 9.39 | 0.00 | 0.00 | 47.86 | 0.00 | 0.00 |
|  | *oprN* | 0.00 | 18.53 | 67.70 | 99.61 | 0.00 | 0.00 | 14.28 | 0.00 | 16.74 | 0.00 | 0.00 |
|  | *evgS* | 0.00 | 34.80 | 1702.39 | 27.12 | 69.27 | 59.61 | 0.00 | 0.00 | 25.45 | 0.00 | 0.00 |
| Polymyxin | *arnA* | 57.40 | 63.82 | 0.00 | 32.00 | 94.21 | 90.33 | 0.00 | 0.00 | 22.47 | 0.00 | 0.00 |
| Trimethoprim | *dfrA* | 3531.95 | 306.33 | 261.35 | 17.65 | 35.31 | 0.00 | 0.00 | 0.00 | 89.42 | 0.00 | 0.00 |
|  | *dfrE* | 0.00 | 0.00 | 0.00 | 17.14 | 0.00 | 0.00 | 0.00 | 48.65 | 0.00 | 0.00 | 0.00 |

Table S3C. Abundance of the shared resistome between wastewater and surface waters.

| ARG Type | ARG subtype | TW1 | TW2 | BH | BI | BB | MA | RA |
| --- | --- | --- | --- | --- | --- | --- | --- | --- |
| Beta-lactam (Class D) | *bla*_OXA-198_ | 0.00 | 24.28 | 13.36 | 25.33 | 0.00 | 0.00 | 0.00 |

Table S3D. Abundance of the shared resistome between wastewater and treated effluent.

| ARG Type | ARG subtype | H1 | H2 | H3 | H4 | H5 | WW | TW1 | TW2 |
| --- | --- | --- | --- | --- | --- | --- | --- | --- | --- |
| Aminoglycoside | *aph(6)-Id* | 394.96 | 0.00 | 0.00 | 0.00 | 0.00 | 0.00 | 0.00 | 19.47 |
| Multidrug resistance efflux pumps | ABC Antibiotic Efflux Pump | 206.46 | 143.70 | 99.43 | 371.13 | 98.13 | 53.64 | 35.01 | 11.99 |
| MLS | *ermB* | 186.36 | 305.63 | 27.61 | 162.55 | 32.52 | 46.29 | 0.00 | 45.05 |
| Beta-lactam (Class A) | *bla*_VEB-1a_ | 71.44 | 0.00 | 24.81 | 0.00 | 0.00 | 75.14 | 0.00 | 13.46 |
| Beta-lactam (Class D) | *bla*_OXA-347_ | 116.91 | 43.08 | 1079.60 | 17.77 | 5.46 | 10.90 | 12.48 | 0.00 |
| Vancomycin | *vanX* | 72.49 | 0.00 | 68.46 | 33.48 | 0.00 | 0.00 | 182.73 | 604.19 |
| Tetracycline | *tetO* | 29.00 | 98.88 | 329.73 | 74.22 | 56.62 | 37.09 | 0.00 | 12.12 |
| Streptogramin | *vatB* | 0.00 | 0.00 | 16.84 | 0.00 | 0.00 | 13.73 | 0.00 | 41.92 |
| Sulfonamide | *sul3* | 0.00 | 0.00 | 0.00 | 0.00 | 12.48 | 0.00 | 0.00 | 37.36 |

Table S4. Abundance of ARG types.

|  | Abundance of ARG types (coverage x/GB) | | | | | | | | | | | | |
| --- | --- | --- | --- | --- | --- | --- | --- | --- | --- | --- | --- | --- | --- |
|  | Wastewaters | | | | | | Treated effluent | | Surface waters | | | | |
| ARG type | H1 | H2 | H3 | H4 | H5 | WW | TW1 | TW2 | BH | BI | BB | MA | RA |
| Aminoglycoside | 104293.76 | 4758.84 | 25851.09 | 2352.15 | 9239.73 | 648.72 | 270.38 | 716.15 | 252.22 | 1069.92 | 1502.23 | 14200.09 | 371.57 |
| Bacitracin | 565.65 | 214.62 | 13979.89 | 53.96 | 240.71 | 19.49 | 0.00 | 0.00 | 0.00 | 400.56 | 0.00 | 0.00 | 0.00 |
| Beta-lactam  (class A) | 279150.17 | 7762.85 | 28285.12 | 825.23 | 1512.45 | 674.10 | 8.07 | 13.46 | 0.00 | 0.00 | 55.21 | 0.00 | 0.00 |
| Beta-lactam  (class B) | 234.61 | 396.11 | 161.76 | 135.66 | 545.63 | 0.00 | 0.00 | 0.00 | 0.00 | 0.00 | 0.00 | 0.00 | 0.00 |
| Beta-lactam  (class C) | 300.62 | 161.33 | 23602.22 | 81.74 | 26161.48 | 40.75 | 0.00 | 0.00 | 0.00 | 0.00 | 9.90 | 0.00 | 0.00 |
| Beta-lactam  (class D) | 3586.44 | 744.75 | 1796.35 | 187.46 | 7133.39 | 120.73 | 68.30 | 24.28 | 148.09 | 25.33 | 61.85 | 0.00 | 0.00 |
| Bleomycin | 10.01 | 8.21 | 107.18 | 14.59 | 0.00 | 0.00 | 0.00 | 0.00 | 0.00 | 0.00 | 0.00 | 0.00 | 0.00 |
| Chloramphenicol | 5060.78 | 550.77 | 458.15 | 227.43 | 548.71 | 100.38 | 0.00 | 0.00 | 0.00 | 0.00 | 30.06 | 0.00 | 0.00 |
| Multidrug resistant efflux pumps | 26738.65 | 7067.35 | 396018.54 | 3249.77 | 26134.55 | 3127.17 | 858.45 | 1009.16 | 847.42 | 696.12 | 9169.52 | 232.64 | 191.62 |
| Fosfomycin | 1532.62 | 28.91 | 0.00 | 0.00 | 47.76 | 20.79 | 0.00 | 0.00 | 0.00 | 59.20 | 0.00 | 0.00 | 0.00 |
| Lincosamide | 128.64 | 1037.56 | 731.01 | 4130.64 | 1532.79 | 94.48 | 0.00 | 0.00 | 0.00 | 0.00 | 0.00 | 0.00 | 0.00 |
| Macrolide | 1573.87 | 487.36 | 728.95 | 139.49 | 364.68 | 168.86 | 0.00 | 12.12 | 9.23 | 0.00 | 0.00 | 0.00 | 0.00 |
| MLSB | 795.67 | 398.87 | 1261.63 | 467.71 | 147.52 | 191.96 | 5.65 | 45.05 | 0.00 | 0.00 | 123.87 | 0.00 | 0.00 |
| Polymyxin | 57.40 | 63.82 | 7714.89 | 32.00 | 94.21 | 90.33 | 0.00 | 0.00 | 0.00 | 0.00 | 22.47 | 0.00 | 0.00 |
| Quinolone | 8984.06 | 14309.64 | 10933.03 | 613.71 | 5328.19 | 988.40 | 383.25 | 377.84 | 464.12 | 836.90 | 2920.25 | 5446.78 | 232.01 |
| Rifampin | 758.09 | 40.47 | 1857.76 | 0.00 | 47.37 | 0.00 | 120.14 | 411.61 | 0.00 | 0.00 | 0.00 | 8.99 | 0.00 |
| Streptogramin | 0.00 | 0.00 | 16.84 | 0.00 | 0.00 | 13.73 | 0.00 | 0.00 | 0.00 | 0.00 | 0.00 | 0.00 | 0.00 |
| Streptothricin | 30.95 | 38.19 | 98.55 | 8.38 | 0.00 | 0.00 | 0.00 | 0.00 | 0.00 | 0.00 | 0.00 | 0.00 | 0.00 |
| Sulfonamide | 81467.55 | 2194.98 | 354.44 | 77.82 | 151.24 | 43.74 | 19.48 | 96.57 | 5.40 | 156.03 | 60.92 | 0.00 | 0.00 |
| Tetracyline | 1420.05 | 1411.47 | 4833.01 | 1017.17 | 919.29 | 1445.46 | 27.78 | 88.63 | 10.20 | 32.47 | 136.33 | 0.00 | 0.00 |
| Trimethoprim | 3680.27 | 274.86 | 2006.12 | 90.08 | 45.45 | 95.36 | 0.00 | 0.00 | 0.00 | 48.65 | 89.42 | 0.00 | 0.00 |
| Vancomycin | 394.35 | 114.76 | 1078.57 | 351.02 | 27.29 | 29.29 | 182.73 | 646.11 | 0.00 | 0.00 | 0.00 | 0.00 | 0.00 |
| Total coverage | 520764.23 | 42065.72 | 521875.10 | 14056.03 | 80222.43 | 7913.74 | 1944.22 | 3440.97 | 1736.69 | 3325.17 | 14182.02 | 19888.50 | 795.20 |

Table S5. Abundance of clinically important beta-lactamase resistance genes.

| **ARGs** | **Abundance of ARGs** | | | | | | | | | | | | |
| --- | --- | --- | --- | --- | --- | --- | --- | --- | --- | --- | --- | --- | --- |
| ***Class A beta-lactamase*** | H1 | H2 | H3 | H4 | H5 | WW | TW1 | TW2 | BH | BI | BB | MA | RA |
| *bla*_CTX-M-3_ | 0.00 | 0.00 | 331.80 | 0.00 | 0.00 | 0.00 | 0.00 | 0.00 | 0.00 | 0.00 | 0.00 | 0.00 | 0.00 |
| *bla*_CTX-M-15_ | 49.60 | 21.84 | 0.00 | 0.00 | 0.00 | 0.00 | 0.00 | 0.00 | 0.00 | 0.00 | 0.00 | 0.00 | 0.00 |
| *bla*_CTX-M-18_ | 430.51 | 24.06 | 0.00 | 0.00 | 0.00 | 0.00 | 0.00 | 0.00 | 0.00 | 0.00 | 0.00 | 0.00 | 0.00 |
| *bla*_CTX-M-147_ | 0.00 | 0.00 | 0.00 | 0.00 | 0.00 | 0.00 | 0.00 | 0.00 | 0.00 | 0.00 | 0.00 | 0.00 | 0.00 |
| *bla*_KPC-2_ | 277258.43 | 7141.84 | 0.00 | 0.00 | 0.00 | 0.00 | 0.00 | 0.00 | 0.00 | 0.00 | 0.00 | 0.00 | 0.00 |
| *bla*_SHV-12_ | 0.00 | 0.00 | 46.38 | 0.00 | 0.00 | 0.00 | 0.00 | 0.00 | 0.00 | 0.00 | 0.00 | 0.00 | 0.00 |
| *bla*_SHV-17_ | 0.00 | 0.00 | 0.00 | 0.00 | 35.30 | 0.00 | 0.00 | 0.00 | 0.00 | 0.00 | 0.00 | 0.00 | 0.00 |
| *bla*_SHV-28_ | 0.00 | 0.00 | 26.87 | 0.00 | 0.00 | 0.00 | 0.00 | 0.00 | 0.00 | 0.00 | 0.00 | 0.00 | 0.00 |
| *bla*_SHV-36_ | 215.47 | 16.58 | 0.00 | 0.00 | 0.00 | 0.00 | 0.00 | 0.00 | 0.00 | 0.00 | 0.00 | 0.00 | 0.00 |
| *bla*_SHV-123_ | 10.75 | 14.48 | 0.00 | 27.16 | 0.00 | 0.00 | 0.00 | 0.00 | 0.00 | 0.00 | 0.00 | 0.00 | 0.00 |
| *bla*_TEM_ | 0.00 | 87.31 | 17336.71 | 0.00 | 85.47 | 62.99 | 0.00 | 0.00 | 0.00 | 0.00 | 0.00 | 0.00 | 0.00 |
| *bla*_TEM-157_ | 275.08 | 0.00 | 0.00 | 0.00 | 0.00 | 0.00 | 0.00 | 0.00 | 0.00 | 0.00 | 0.00 | 0.00 | 0.00 |
| *bla*_TEM-163_ | 0.00 | 0.00 | 0.00 | 8.85 | 0.00 | 0.00 | 0.00 | 0.00 | 0.00 | 0.00 | 0.00 | 0.00 | 0.00 |
| *bla*_TEM-211_ | 24.45 | 0.00 | 0.00 | 0.00 | 0.00 | 0.00 | 0.00 | 0.00 | 0.00 | 0.00 | 0.00 | 0.00 | 0.00 |
| ***Class B beta-lactamase*** |  |  |  |  |  |  |  |  |  |  |  |  |  |
| *bla*_IMP-1_ | 141.77 | 0.00 | 11.87 | 127.71 | 87.16 | 0.00 | 0.00 | 0.00 | 0.00 | 0.00 | 0.00 | 0.00 | 0.00 |
| *bla*_IMP-4_ | 0.00 | 130.98 | 0.00 | 0.00 | 0.00 | 0.00 | 0.00 | 0.00 | 0.00 | 0.00 | 0.00 | 0.00 | 0.00 |
| *bla*_IMP-42_ | 0.00 | 37.45 | 0.00 | 0.00 | 0.00 | 0.00 | 0.00 | 0.00 | 0.00 | 0.00 | 0.00 | 0.00 | 0.00 |
| *bla*_IMP-6_ | 0.00 | 93.79 | 0.00 | 0.00 | 0.00 | 0.00 | 0.00 | 0.00 | 0.00 | 0.00 | 0.00 | 0.00 | 0.00 |
| *bla*_NDM-1_ | 17.45 | 0.00 | 0.00 | 0.00 | 0.00 | 0.00 | 0.00 | 0.00 | 0.00 | 0.00 | 0.00 | 0.00 | 0.00 |
| *bla*_NDM-2_ | 14.17 | 19.34 | 12.66 | 0.00 | 0.00 | 0.00 | 0.00 | 0.00 | 0.00 | 0.00 | 0.00 | 0.00 | 0.00 |
| *bla*_NDM-3_ | 0.00 | 0.00 | 14.83 | 7.95 | 0.00 | 0.00 | 0.00 | 0.00 | 0.00 | 0.00 | 0.00 | 0.00 | 0.00 |
| *bla*_VIM-2_ | 47.74 | 17.73 | 0.00 | 0.00 | 0.00 | 0.00 | 0.00 | 0.00 | 0.00 | 0.00 | 0.00 | 0.00 | 0.00 |
| *bla*_VIM-3_ | 0.00 | 0.00 | 0.00 | 0.00 | 26.20 | 0.00 | 0.00 | 0.00 | 0.00 | 0.00 | 0.00 | 0.00 | 0.00 |
| *bla*_VIM-11_ | 0.00 | 10.45 | 0.00 | 0.00 | 0.00 | 0.00 | 0.00 | 0.00 | 0.00 | 0.00 | 0.00 | 0.00 | 0.00 |
| *bla*_VIM-12_ | 0.00 | 0.00 | 0.00 | 8.64 | 0.00 | 0.00 | 0.00 | 0.00 | 0.00 | 0.00 | 0.00 | 0.00 | 0.00 |
| *bla*_VIM-18_ | 0.00 | 0.00 | 8.06 | 0.00 | 0.00 | 0.00 | 0.00 | 0.00 | 0.00 | 0.00 | 0.00 | 0.00 | 0.00 |
| *bla*_VIM-26_ | 0.00 | 0.00 | 5.56 | 0.00 | 0.00 | 0.00 | 0.00 | 0.00 | 0.00 | 0.00 | 0.00 | 0.00 | 0.00 |
| *bla*_VIM-30_ | 0.00 | 0.00 | 55.76 | 0.00 | 0.00 | 0.00 | 0.00 | 0.00 | 0.00 | 0.00 | 0.00 | 0.00 | 0.00 |
| ***Class C beta-lactamase*** |  |  |  |  |  |  |  |  |  |  |  |  |  |
| *ampC* | 0.00 | 142.69 | 23568.69 | 35.85 | 25995.22 | 40.75 | 0.00 | 0.00 | 0.00 | 0.00 | 0.00 | 0.00 | 0.00 |
| *bla*_CMY-112_ | 22.64 | 0.00 | 0.00 | 0.00 | 0.00 | 0.00 | 0.00 | 0.00 | 0.00 | 0.00 | 0.00 | 0.00 | 0.00 |
| *bla*_CMY-78_ | 0.00 | 0.00 | 33.53 | 0.00 | 0.00 | 0.00 | 0.00 | 0.00 | 0.00 | 0.00 | 0.00 | 0.00 | 0.00 |
| ***Class D beta-lactamase*** |  |  |  |  |  |  |  |  |  |  |  |  |  |
| *bla*_OXA_ | 0.00 | 0.00 | 13.53 | 0.00 | 115.64 | 0.00 | 0.00 | 0.00 | 70.31 | 0.00 | 0.00 | 0.00 | 0.00 |
| *bla*_OXA-1_ | 1486.91 | 379.94 | 482.83 | 12.83 | 48.13 | 0.00 | 0.00 | 0.00 | 0.00 | 0.00 | 0.00 | 0.00 | 0.00 |
| *bla*_OXA-2_ | 5.77 | 29.05 | 19.50 | 4.97 | 15.48 | 0.00 | 0.00 | 0.00 | 0.00 | 0.00 | 0.00 | 0.00 | 0.00 |
| *bla*_OXA-5_ | 0.00 | 0.00 | 13.99 | 73.00 | 0.00 | 0.00 | 0.00 | 0.00 | 0.00 | 0.00 | 0.00 | 0.00 | 0.00 |
| *bla*_OXA-10_ | 1546.73 | 127.57 | 49.63 | 38.27 | 6349.55 | 0.00 | 0.00 | 0.00 | 0.00 | 0.00 | 0.00 | 0.00 | 0.00 |
| *bla*_OXA-12_ | 101.27 | 186.21 | 34.38 | 0.00 | 126.59 | 0.00 | 0.00 | 0.00 | 0.00 | 0.00 | 0.00 | 0.00 | 0.00 |
| *bla*_OXA-13_ | 0.00 | 0.00 | 45.25 | 0.00 | 0.00 | 0.00 | 0.00 | 0.00 | 0.00 | 0.00 | 0.00 | 0.00 | 0.00 |
| *bla*_OXA-21_ | 193.78 | 19.12 | 0.00 | 0.00 | 0.00 | 0.00 | 0.00 | 0.00 | 0.00 | 0.00 | 0.00 | 0.00 | 0.00 |
| *bla*_OXA-35_ | 0.00 | 0.00 | 0.00 | 0.00 | 0.00 | 0.00 | 0.00 | 0.00 | 0.00 | 0.00 | 30.76 | 0.00 | 0.00 |
| *bla*_OXA-58_ | 0.00 | 4.66 | 7.37 | 6.86 | 9.56 | 0.00 | 0.00 | 0.00 | 0.00 | 0.00 | 0.00 | 0.00 | 0.00 |
| *bla*_OXA-129_ | 16.92 | 15.48 | 15.19 | 0.00 | 0.00 | 0.00 | 0.00 | 0.00 | 0.00 | 0.00 | 0.00 | 0.00 | 0.00 |
| *bla*_OXA-141_ | 0.00 | 0.00 | 0.00 | 0.00 | 418.00 | 0.00 | 0.00 | 0.00 | 0.00 | 0.00 | 0.00 | 0.00 | 0.00 |
| *bla*_OXA-167_ | 0.00 | 0.00 | 5.56 | 0.00 | 0.00 | 0.00 | 0.00 | 0.00 | 0.00 | 0.00 | 0.00 | 0.00 | 0.00 |
| *bla*_OXA-198_ | 0.00 | 0.00 | 0.00 | 0.00 | 0.00 | 0.00 | 0.00 | 24.28 | 13.36 | 25.33 | 0.00 | 0.00 | 0.00 |
| *bla*_OXA-209_ | 0.00 | 0.00 | 0.00 | 0.00 | 0.00 | 0.00 | 0.00 | 0.00 | 39.36 | 0.00 | 31.10 | 0.00 | 0.00 |
| *bla*_OXA-211_ | 0.00 | 0.00 | 6.91 | 0.00 | 0.00 | 0.00 | 0.00 | 0.00 | 0.00 | 0.00 | 0.00 | 0.00 | 0.00 |
| *bla*_OXA-225_ | 0.00 | 0.00 | 0.00 | 15.07 | 0.00 | 0.00 | 0.00 | 0.00 | 0.00 | 0.00 | 0.00 | 0.00 | 0.00 |
| *bla*_OXA-226_ | 13.20 | 0.00 | 12.57 | 0.00 | 0.00 | 0.00 | 0.00 | 0.00 | 0.00 | 0.00 | 0.00 | 0.00 | 0.00 |
| *bla*_OXA-333_ | 0.00 | 0.00 | 0.00 | 0.00 | 0.00 | 36.09 | 0.00 | 0.00 | 0.00 | 0.00 | 0.00 | 0.00 | 0.00 |
| *bla*_OXA-334_ | 0.00 | 25.95 | 0.00 | 0.00 | 5.82 | 0.00 | 0.00 | 0.00 | 0.00 | 0.00 | 0.00 | 0.00 | 0.00 |
| *bla*_OXA-347_ | 116.91 | 43.08 | 1079.60 | 17.77 | 5.46 | 10.90 | 12.48 | 0.00 | 0.00 | 0.00 | 0.00 | 0.00 | 0.00 |
| *bla*_OXA-420_ | 59.79 | 0.00 | 0.00 | 0.00 | 0.00 | 0.00 | 0.00 | 0.00 | 0.00 | 0.00 | 0.00 | 0.00 | 0.00 |

Table S6. Identification of plasmids.

| **Sample** | **Plasmid** | **Percentage Identity** | **Query/HSP length** | **Contig** | **Position in contig** | **ARG present in scaffold** | **Accession number** |
| --- | --- | --- | --- | --- | --- | --- | --- |
| H1 | *IncFIB(K)* | 99.29 | 560 / 560 | Ga0110937_10208381 | 11..570 | - | [JN233704](http://www.ncbi.nlm.nih.gov/nuccore/JN233704) |
|  | *IncHI2A* | 100 | 452 / 630 | Ga0110937_10696961 | 1272..1723 | - | [BX664015](http://www.ncbi.nlm.nih.gov/nuccore/BX664015) |
|  | *IncFII(K)* | 97.3 | 148 / 148 | Ga0110937_10234541 | 1348..1495 | - | [CP000648](http://www.ncbi.nlm.nih.gov/nuccore/CP000648) |
|  | *IncFIA(HI1)* | 97.93 | 387 / 388 | Ga0110937_10250851 | 1412..1798 | - | [AF250878](http://www.ncbi.nlm.nih.gov/nuccore/AF250878) |
|  | *IncP(Beta)* | 99.14 | 583 / 582 | Ga0110937_10076841 | 1451..2032 | - | [U67194](http://www.ncbi.nlm.nih.gov/nuccore/U67194) |
|  | *IncU* | 100 | 565 / 565 | Ga0110937_10290911 | 169..733 | - | [DQ401103](http://www.ncbi.nlm.nih.gov/nuccore/DQ401103) |
|  | *IncP(6)* | 99.87 | 780 / 806 | Ga0110937_10001861 | 1725..2504 | - | [JF785550](http://www.ncbi.nlm.nih.gov/nuccore/JF785550) |
|  | *Col(MGD2)* | 100 | 103 / 136 | Ga0110937_10173991 | 1796..1898 | - | [NC_003789](http://www.ncbi.nlm.nih.gov/nuccore/NC_003789) |
|  | *IncQ2* | 97.09 | 378 / 450 | Ga0110937_10057081 | 1831..2208 | - | [FJ696404](http://www.ncbi.nlm.nih.gov/nuccore/FJ696404) |
|  | *IncR* | 99.6 | 251 / 251 | Ga0110937_11835991 | 191..441 | - | [DQ449578](http://www.ncbi.nlm.nih.gov/nuccore/DQ449578) |
|  | *Col(IMGS31)* | 100 | 156 / 198 | Ga0110937_10307861 | 1..156 | - | [NC_011406](http://www.ncbi.nlm.nih.gov/nuccore/NC_011406) |
|  | *ColKP3* | 96.17 | 183 / 280 | Ga0110937_10004771 | 1..183 | - | [JN205800](http://www.ncbi.nlm.nih.gov/nuccore/JN205800) |
|  | *IncX3* | 100 | 295 / 374 | Ga0110937_10827141 | 1..295 | - | [JN247852](http://www.ncbi.nlm.nih.gov/nuccore/JN247852) |
|  | *IncQ1* | 97.99 | 398 / 450 | Ga0110937_10181191 | 1..398 | - | [HE654726](http://www.ncbi.nlm.nih.gov/nuccore/HE654726) |
|  | *IncFIB(pQil)* | 99.86 | 740 / 740 | Ga0110937_10131031 | 2110..2849 | - | [JN233705](http://www.ncbi.nlm.nih.gov/nuccore/JN233705) |
|  | *IncFIB(Mar)* | 99.54 | 439 / 439 | Ga0110937_10903041 | 286..724 | - | [JN420336](http://www.ncbi.nlm.nih.gov/nuccore/JN420336) |
|  | *IncHI2* | 100 | 251 / 327 | Ga0110937_10201521 | 335..585 | - | [BX664015](http://www.ncbi.nlm.nih.gov/nuccore/BX664015) |
|  | *IncI2* | 97.82 | 229 / 316 | Ga0110937_10709151 | 35..263 | - | [AP002527](http://www.ncbi.nlm.nih.gov/nuccore/AP002527) |
|  | *IncHI1B* | 99.47 | 570 / 570 | Ga0110937_10416401 | 515..1084 | - | [JN420336](http://www.ncbi.nlm.nih.gov/nuccore/JN420336) |
|  | *Col3M* | 98.09 | 157 / 157 | Ga0110937_10008501 | 5329..5485 | - | [JX514065](http://www.ncbi.nlm.nih.gov/nuccore/JX514065) |
|  | *IncFII(K)* | 95.95 | 148 / 148 | Ga0110937_10022661 | 5448..5595 | - | [CP000648](http://www.ncbi.nlm.nih.gov/nuccore/CP000648) |
|  | *IncN* | 99.81 | 514 / 514 | Ga0110937_1004240 | 5630..6143 | - | [AY046276](http://www.ncbi.nlm.nih.gov/nuccore/AY046276) |
|  | *repA* | 99.71 | 693 / 693 | Ga0110937_1001632 | 792..1482 | *qnrS1* (Ga0110937_100163213) | [JX397875](http://www.ncbi.nlm.nih.gov/nuccore/JX397875) |
|  | *IncA/C2* | 99.52 | 417 / 417 | Ga0110937_1000169 | 80034..80450 | - | [JN157804](http://www.ncbi.nlm.nih.gov/nuccore/JN157804) |
| H2 | *IncX3* | 100 | 371 / 374 | Ga0110938_12076471 | 104..474 | - | [JN247852](http://www.ncbi.nlm.nih.gov/nuccore/JN247852) |
|  | *IncFIB(K)* | 99.11 | 560 / 560 | Ga0110938_10125481 | 1225..1784 | - | [JN233704](http://www.ncbi.nlm.nih.gov/nuccore/JN233704) |
|  | *IncA/C2* | 99.52 | 417 / 417 | Ga0110938_10016091 | 15388..15804 | - | [JN157804](http://www.ncbi.nlm.nih.gov/nuccore/JN157804) |
|  | *IncHI2* | 100 | 327 / 327 | Ga0110938_10231771 | 160..486 | - | [BX664015](http://www.ncbi.nlm.nih.gov/nuccore/BX664015) |
|  | *IncP(6)* | 99.87 | 780 / 806 | Ga0110938_10014351 | 1725..2504 | - | [JF785550](http://www.ncbi.nlm.nih.gov/nuccore/JF785550) |
|  | *IncN* | 99.81 | 514 / 514 | Ga0110938_10376681 | 1752..2265 | - | [AY046276](http://www.ncbi.nlm.nih.gov/nuccore/AY046276) |
|  | *IncX1* | 97.33 | 374 / 374 | Ga0110938_11682501 | 188..561 | - | [EU370913](http://www.ncbi.nlm.nih.gov/nuccore/EU370913) |
|  | *IncR* | 100 | 214 / 251 | Ga0110938_10904051 | 1..214 | - | [DQ449578](http://www.ncbi.nlm.nih.gov/nuccore/DQ449578) |
|  | *ColKP3* | 97.13 | 279 / 280 | Ga0110938_10141141 | 1..279 | - | [JN205800](http://www.ncbi.nlm.nih.gov/nuccore/JN205800) |
|  | *IncFIA(HI1)* | 97.44 | 352 / 388 | Ga0110938_10206071 | 1..352 | - | [AF250878](http://www.ncbi.nlm.nih.gov/nuccore/AF250878) |
|  | *repA* | 99.58 | 479 / 693 | Ga0110938_10533901 | 1..477 | - | [JX397875](http://www.ncbi.nlm.nih.gov/nuccore/JX397875) |
|  | *IncFIB(pQil)* | 99.7 | 669 / 740 | Ga0110938_11470041 | 1..669 | - | [JN233705](http://www.ncbi.nlm.nih.gov/nuccore/JN233705) |
|  | *IncP(Beta)* | 98.8 | 583 / 582 | Ga0110938_10371611 | 2158..2739 | - | [U67194](http://www.ncbi.nlm.nih.gov/nuccore/U67194) |
|  | *Col3M* | 98.09 | 157 / 157 | Ga0110938_10048571 | 5843..5999 | - | [JX514065](http://www.ncbi.nlm.nih.gov/nuccore/JX514065) |
|  | *IncU* | 100 | 565 / 565 | Ga0110938_10185231 | 692..1256 | - | [DQ401103](http://www.ncbi.nlm.nih.gov/nuccore/DQ401103) |
|  | *IncFII* | 98.85 | 261 / 261 | Ga0110938_10043061 | 6933..7193 | - | [AY458016](http://www.ncbi.nlm.nih.gov/nuccore/AY458016) |
|  | *Col(IMGS31)* | 98.99 | 198 / 198 | Ga0110938_10073791 | 995..1192 | - | [NC_011406](http://www.ncbi.nlm.nih.gov/nuccore/NC_011406) |
| H3 | *IncU* | 100 | 565 / 565 | Ga0110939_10041991 | 1076..1640 | - | [DQ401103](http://www.ncbi.nlm.nih.gov/nuccore/DQ401103) |
|  | *IncP(6)* | 97.92 | 816 / 806 | Ga0110939_10340091 | 1894..2709 | - | [JF785550](http://www.ncbi.nlm.nih.gov/nuccore/JF785550) |
|  | *IncHI2A* | 100 | 553 / 630 | Ga0110939_10796971 | 1..553 | - | [BX664015](http://www.ncbi.nlm.nih.gov/nuccore/BX664015) |
|  | *IncFIB(pLF82)* | 96.11 | 411 / 560 | Ga0110939_11957791 | 233..643 | - | [CU638872](http://www.ncbi.nlm.nih.gov/nuccore/CU638872) |
|  | *IncFIB(K)* | 98.9 | 363 / 560 | Ga0110939_11968331 | 366..728 | - | [JN233704](http://www.ncbi.nlm.nih.gov/nuccore/JN233704) |
|  | *Col156* | 95.45 | 154 / 154 | Ga0110939_10068631 | 4600..4753 | - | [NC_009781](http://www.ncbi.nlm.nih.gov/nuccore/NC_009781) |
|  | *IncI1* | 100 | 93 / 142 | Ga0110939_11678231 | 534..626 | - | [AP005147](http://www.ncbi.nlm.nih.gov/nuccore/AP005147) |
|  | *IncN* | 99.81 | 514 / 514 | Ga0110939_10034741 | 5630..6143 | - | [AY046276](http://www.ncbi.nlm.nih.gov/nuccore/AY046276) |
|  | *IncFII(29)* | 99.61 | 259 / 259 | Ga0110939_10001761 | 6098..6356 | *bla*_TEM-1_ (Ga0110939_10001761) | [CP003035](http://www.ncbi.nlm.nih.gov/nuccore/CP003035) |
|  | *Col156* | 98.59 | 142 / 154 | Ga0110939_10032721 | 6974..7115 | - | [NC_009781](http://www.ncbi.nlm.nih.gov/nuccore/NC_009781) |
|  | *IncFIB(AP001918)* | 96.63 | 682 / 682 | Ga0110939_10010401 | 8390..9071 | - | [AP001918](http://www.ncbi.nlm.nih.gov/nuccore/AP001918) |
| H4 | *Col156* | 98.05 | 154 / 154 | Ga0110940_10287991 | 3652..3805 | - | [NC_009781](http://www.ncbi.nlm.nih.gov/nuccore/NC_009781) |
|  | *IncA/C2* | 100 | 406 / 417 | Ga0110940_10734081 | 404..809 | - | [JN157804](http://www.ncbi.nlm.nih.gov/nuccore/JN157804) |
| H5 | *IncA/C2* | 99.52 | 417 / 417 | Ga0110941_11202401 | 1022..1438 | - | [JN157804](http://www.ncbi.nlm.nih.gov/nuccore/JN157804) |
|  | *Col(BS512)* | 100 | 193 / 193 | Ga0110941_10329171 | 10..202 | - | [NC_010656](http://www.ncbi.nlm.nih.gov/nuccore/NC_010656) |
|  | *IncI1* | 96.27 | 134 / 142 | Ga0110941_10749601 | 120..252 | - | [AP005147](http://www.ncbi.nlm.nih.gov/nuccore/AP005147) |
|  | *IncHI2A* | 99.75 | 398 / 630 | Ga0110941_11059471 | 1..398 | - | [BX664015](http://www.ncbi.nlm.nih.gov/nuccore/BX664015) |
|  | *IncY* | 99.42 | 520 / 765 | Ga0110941_10734621 | 1..520 | - | [K02380](http://www.ncbi.nlm.nih.gov/nuccore/K02380) |
|  | *ColKP3* | 97.36 | 265 / 280 | Ga0110941_10033721 | 20..284 | - | [JN205800](http://www.ncbi.nlm.nih.gov/nuccore/JN205800) |
|  | *Col(BS512)* | 100 | 233 / 233 | Ga0110941_10329171 | 274..506 | - | [NC_010656](http://www.ncbi.nlm.nih.gov/nuccore/NC_010656) |
|  | *IncHI2* | 100 | 327 / 327 | Ga0110941_11334831 | 355..681 | - | [BX664015](http://www.ncbi.nlm.nih.gov/nuccore/BX664015) |
|  | *IncP(Beta)* | 99.14 | 583 / 582 | Ga0110941_10017801 | 397..978 | - | [U67194](http://www.ncbi.nlm.nih.gov/nuccore/U67194) |
|  | *IncQ2* | 98.89 | 450 / 450 | Ga0110941_10012391 | 4320..4769 | - | [FJ696404](http://www.ncbi.nlm.nih.gov/nuccore/FJ696404) |
|  | *IncU* | 100 | 565 / 565 | Ga0110941_10012431 | 528..1092 | - | [DQ401103](http://www.ncbi.nlm.nih.gov/nuccore/DQ401103) |
|  | *IncFIB(AP001918)* | 98.39 | 682 / 682 | Ga0110941_11096041 | 553..1234 | - | [AP001918](http://www.ncbi.nlm.nih.gov/nuccore/AP001918) |
|  | *IncFIA(HI1)* | 95.62 | 388 / 388 | Ga0110941_10567271 | 61..448 | - | [AF250878](http://www.ncbi.nlm.nih.gov/nuccore/AF250878) |
| WW | *IncP(6)* | 99.38 | 806 / 806 | Ga0110933_10146131 | 1046..1851 | - | [JF785550](http://www.ncbi.nlm.nih.gov/nuccore/JF785550) |
|  | *Col(IMGS31)* | 97.47 | 198 / 198 | Ga0110933_11556731 | 187..384 | - | [NC_011406](http://www.ncbi.nlm.nih.gov/nuccore/NC_011406) |
|  | *Col3M* | 97.58 | 124 / 157 | Ga0110933_12248201 | 1..124 | - | [JX514065](http://www.ncbi.nlm.nih.gov/nuccore/JX514065) |
|  | *IncQ2* | 95.87 | 387 / 450 | Ga0110933_10062091 | 2745..3131 | *qnrS2* (Ga0110933_10062091) | [FJ696404](http://www.ncbi.nlm.nih.gov/nuccore/FJ696404) |
| BB | *IncQ2* | 97.7 | 434 / 450 | Ga0110930_11900431 | 1..434 | - | [FJ696404](http://www.ncbi.nlm.nih.gov/nuccore/FJ696404) |

Table S7. No. of contigs assigned to identified taxa.

| Genera | H1 | H2 | H3 | H4 | H5 | WW | TW1 | TW2 | BH | BI | BB | MA | RA |
| --- | --- | --- | --- | --- | --- | --- | --- | --- | --- | --- | --- | --- | --- |
| *Empedobacter* | 0 | 0 | 0 | 0 | 33 | 0 | 0 | 0 | 0 | 0 | 0 | 0 | 0 |
| *Lysinibacillus* | 0 | 0 | 0 | 0 | 36 | 0 | 0 | 0 | 0 | 0 | 0 | 0 | 0 |
| *Acetobacter* | 0 | 0 | 0 | 0 | 53 | 0 | 0 | 0 | 0 | 0 | 0 | 0 | 0 |
| *Xanthobacter* | 0 | 0 | 0 | 0 | 113 | 0 | 0 | 0 | 0 | 0 | 0 | 0 | 0 |
| *Holdemania* | 0 | 0 | 0 | 18 | 0 | 0 | 0 | 0 | 0 | 0 | 0 | 0 | 0 |
| *Senegalimassilia* | 0 | 0 | 0 | 32 | 0 | 0 | 0 | 0 | 0 | 0 | 0 | 0 | 0 |
| *Myroides* | 0 | 0 | 0 | 44 | 0 | 0 | 0 | 0 | 0 | 0 | 0 | 0 | 0 |
| *Vagococcus* | 0 | 0 | 0 | 48 | 0 | 0 | 0 | 0 | 0 | 0 | 0 | 0 | 0 |
| *Anaerovibrio* | 0 | 0 | 0 | 71 | 0 | 0 | 0 | 0 | 0 | 0 | 0 | 0 | 0 |
| *Alcaligenes* | 0 | 0 | 0 | 75 | 0 | 0 | 0 | 0 | 0 | 0 | 0 | 0 | 0 |
| *Dolosicoccus* | 0 | 0 | 0 | 123 | 0 | 0 | 0 | 0 | 0 | 0 | 0 | 0 | 0 |
| *Facklamia* | 0 | 0 | 0 | 151 | 0 | 0 | 0 | 0 | 0 | 0 | 0 | 0 | 0 |
| *Gardnerella* | 0 | 0 | 29 | 0 | 0 | 0 | 0 | 0 | 0 | 0 | 0 | 0 | 0 |
| *Angelakisella* | 0 | 0 | 40 | 0 | 0 | 0 | 0 | 0 | 0 | 0 | 0 | 0 | 0 |
| *Candidatus Stoquefichus* | 0 | 0 | 48 | 0 | 0 | 0 | 0 | 0 | 0 | 0 | 0 | 0 | 0 |
| *Robinsoniella* | 0 | 0 | 48 | 0 | 0 | 0 | 0 | 0 | 0 | 0 | 0 | 0 | 0 |
| *Bariatricus* | 0 | 0 | 54 | 0 | 0 | 0 | 0 | 0 | 0 | 0 | 0 | 0 | 0 |
| *Peptococcus* | 0 | 0 | 64 | 0 | 0 | 0 | 0 | 0 | 0 | 0 | 0 | 0 | 0 |
| *Morganella* | 0 | 0 | 91 | 0 | 51 | 0 | 0 | 0 | 0 | 0 | 0 | 0 | 0 |
| *Weissella* | 0 | 0 | 113 | 0 | 0 | 0 | 0 | 0 | 0 | 0 | 0 | 0 | 0 |
| *Providencia* | 0 | 0 | 246 | 0 | 35 | 0 | 0 | 0 | 0 | 0 | 0 | 0 | 0 |
| *Shinella* | 0 | 0 | 391 | 0 | 0 | 0 | 0 | 0 | 0 | 0 | 0 | 0 | 0 |
| *Drancourtella* | 0 | 0 | 414 | 0 | 0 | 0 | 0 | 0 | 0 | 0 | 0 | 0 | 0 |
| *Clostridioides* | 0 | 0 | 557 | 71 | 0 | 0 | 0 | 0 | 0 | 0 | 0 | 0 | 0 |
| *Brachyspira* | 0 | 18 | 0 | 0 | 0 | 0 | 0 | 0 | 0 | 0 | 0 | 0 | 0 |
| *Olsenella* | 0 | 20 | 47 | 0 | 49 | 0 | 0 | 0 | 0 | 0 | 0 | 0 | 0 |
| *Allisonella* | 0 | 22 | 38 | 0 | 0 | 0 | 0 | 0 | 0 | 0 | 0 | 0 | 0 |
| *Fournierella* | 0 | 29 | 38 | 44 | 0 | 0 | 0 | 0 | 0 | 0 | 0 | 0 | 0 |
| *Parasutterella* | 0 | 31 | 0 | 30 | 0 | 0 | 0 | 0 | 0 | 0 | 0 | 0 | 0 |
| *Proteus* | 0 | 34 | 143 | 0 | 0 | 0 | 0 | 0 | 0 | 0 | 0 | 0 | 0 |
| *Propionispora* | 0 | 155 | 82 | 43 | 0 | 0 | 0 | 0 | 0 | 0 | 0 | 0 | 0 |
| *Photobacterium* | 27 | 0 | 0 | 0 | 0 | 0 | 0 | 0 | 0 | 0 | 0 | 0 | 0 |
| *Yersinia* | 28 | 0 | 0 | 0 | 0 | 0 | 0 | 0 | 0 | 0 | 0 | 0 | 0 |
| *Acholeplasma* | 32 | 0 | 0 | 0 | 0 | 0 | 0 | 0 | 0 | 0 | 0 | 0 | 0 |
| *Raoultella* | 33 | 0 | 0 | 0 | 0 | 0 | 0 | 0 | 0 | 0 | 0 | 0 | 0 |
| *Aquitalea* | 34 | 0 | 74 | 0 | 0 | 0 | 0 | 0 | 0 | 0 | 0 | 0 | 0 |
| *Microlunatus* | 34 | 0 | 81 | 0 | 0 | 0 | 0 | 0 | 0 | 0 | 0 | 0 | 0 |
| *Proteiniclasticum* | 37 | 0 | 0 | 0 | 0 | 0 | 0 | 0 | 0 | 0 | 0 | 0 | 0 |
| *Mycoplasma* | 39 | 0 | 0 | 58 | 0 | 0 | 0 | 0 | 0 | 0 | 0 | 0 | 0 |
| *Microvirgula* | 44 | 0 | 0 | 0 | 27 | 0 | 0 | 0 | 0 | 0 | 0 | 0 | 0 |
| *Eikenella* | 50 | 0 | 53 | 96 | 58 | 0 | 0 | 0 | 0 | 0 | 0 | 0 | 0 |
| *Serratia* | 52 | 48 | 0 | 0 | 1128 | 0 | 0 | 0 | 0 | 0 | 0 | 0 | 0 |
| *Bergeriella* | 57 | 0 | 0 | 0 | 0 | 0 | 0 | 0 | 0 | 0 | 0 | 0 | 0 |
| *Vitreoscilla* | 57 | 55 | 0 | 0 | 0 | 0 | 0 | 0 | 0 | 0 | 0 | 0 | 0 |
| *Kineosphaera* | 65 | 0 | 0 | 0 | 0 | 0 | 0 | 0 | 0 | 0 | 0 | 0 | 0 |
| *Propionibacterium* | 69 | 0 | 0 | 0 | 0 | 0 | 0 | 0 | 0 | 0 | 0 | 0 | 0 |
| *Salmonella* | 71 | 47 | 0 | 0 | 0 | 0 | 0 | 0 | 0 | 0 | 0 | 0 | 0 |
| *Simplicispira* | 78 | 0 | 0 | 0 | 104 | 0 | 0 | 0 | 0 | 0 | 0 | 0 | 0 |
| *Propionicicella* | 82 | 0 | 88 | 0 | 0 | 0 | 0 | 0 | 0 | 0 | 0 | 0 | 0 |
| *Labrys* | 92 | 0 | 229 | 0 | 217 | 0 | 0 | 0 | 0 | 0 | 0 | 0 | 0 |
| *Kocuria* | 112 | 0 | 0 | 0 | 0 | 0 | 0 | 0 | 0 | 0 | 0 | 0 | 0 |
| *Snodgrassella* | 115 | 62 | 0 | 0 | 0 | 0 | 0 | 0 | 0 | 0 | 0 | 0 | 0 |
| *Diaphorobacter* | 122 | 110 | 85 | 141 | 140 | 0 | 0 | 0 | 0 | 0 | 0 | 0 | 0 |
| *Rhizomicrobium* | 166 | 130 | 0 | 0 | 0 | 0 | 0 | 0 | 0 | 0 | 0 | 0 | 0 |
| *Succiniclasticum* | 249 | 39 | 0 | 0 | 0 | 0 | 0 | 0 | 0 | 0 | 0 | 0 | 0 |
| *Fibrobacter* | 490 | 81 | 0 | 0 | 0 | 0 | 0 | 0 | 0 | 0 | 0 | 0 | 0 |
| *Anaeromusa* | 606 | 165 | 0 | 0 | 0 | 0 | 0 | 0 | 0 | 0 | 0 | 0 | 0 |
| *Anaeroarcus* | 802 | 184 | 0 | 0 | 0 | 0 | 0 | 0 | 0 | 0 | 0 | 0 | 0 |
| *Kluyvera* | 1284 | 352 | 84 | 0 | 0 | 0 | 0 | 0 | 0 | 0 | 0 | 0 | 0 |
| *Gloeocapsa* | 0 | 0 | 0 | 0 | 0 | 0 | 0 | 0 | 0 | 0 | 0 | 0 | 41 |
| *Nodosilinea* | 0 | 0 | 0 | 0 | 0 | 0 | 0 | 0 | 0 | 0 | 0 | 0 | 45 |
| *Microcoleus* | 0 | 0 | 0 | 0 | 0 | 0 | 0 | 0 | 0 | 0 | 0 | 0 | 54 |
| *Phormidium* | 0 | 0 | 0 | 0 | 0 | 0 | 0 | 0 | 0 | 0 | 0 | 0 | 74 |
| *Oscillochloris* | 0 | 0 | 0 | 0 | 0 | 0 | 0 | 0 | 0 | 0 | 0 | 0 | 79 |
| *Rubripirellula* | 0 | 0 | 0 | 0 | 0 | 0 | 0 | 0 | 0 | 0 | 0 | 0 | 87 |
| *Oscillatoria* | 0 | 0 | 0 | 0 | 0 | 0 | 0 | 0 | 0 | 0 | 0 | 0 | 94 |
| *Pseudohongiella* | 0 | 0 | 0 | 0 | 0 | 0 | 0 | 0 | 0 | 0 | 0 | 0 | 109 |
| *Calothrix* | 0 | 0 | 0 | 0 | 0 | 0 | 0 | 0 | 0 | 0 | 0 | 0 | 119 |
| *Fischerella* | 0 | 0 | 0 | 0 | 0 | 0 | 0 | 0 | 0 | 0 | 0 | 0 | 119 |
| *Trichormus* | 0 | 0 | 0 | 0 | 0 | 0 | 0 | 0 | 0 | 0 | 0 | 0 | 148 |
| *Blastopirellula* | 0 | 0 | 0 | 0 | 0 | 0 | 0 | 0 | 0 | 0 | 0 | 0 | 180 |
| *Cyanobium* | 0 | 0 | 0 | 0 | 0 | 0 | 0 | 0 | 0 | 0 | 0 | 0 | 317 |
| *Terrimicrobium* | 0 | 0 | 0 | 0 | 0 | 0 | 0 | 0 | 0 | 0 | 0 | 22 | 0 |
| *Phycisphaera* | 0 | 0 | 0 | 0 | 0 | 0 | 0 | 0 | 0 | 0 | 0 | 40 | 179 |
| *Eisenibacter* | 0 | 0 | 0 | 0 | 0 | 0 | 0 | 0 | 0 | 0 | 0 | 60 | 0 |
| *Aurantimicrobium* | 0 | 0 | 0 | 0 | 0 | 0 | 0 | 0 | 0 | 0 | 0 | 60 | 66 |
| *Acaryochloris* | 0 | 0 | 0 | 0 | 0 | 0 | 0 | 0 | 0 | 0 | 0 | 90 | 0 |
| *Candidatus Symbiothrix* | 0 | 0 | 0 | 0 | 0 | 0 | 0 | 0 | 0 | 0 | 0 | 100 | 0 |
| *Nostoc* | 0 | 0 | 0 | 0 | 0 | 0 | 0 | 0 | 0 | 0 | 0 | 104 | 324 |
| *Phormidesmis* | 0 | 0 | 0 | 0 | 0 | 0 | 0 | 0 | 0 | 0 | 0 | 137 | 0 |
| *Prochlorococcus* | 0 | 0 | 0 | 0 | 0 | 0 | 0 | 0 | 0 | 0 | 0 | 145 | 36 |
| *Synechocystis* | 0 | 0 | 0 | 0 | 0 | 0 | 0 | 0 | 0 | 0 | 0 | 145 | 80 |
| *Lyngbya* | 0 | 0 | 0 | 0 | 0 | 0 | 0 | 0 | 0 | 0 | 0 | 150 | 66 |
| *Geminocystis* | 0 | 0 | 0 | 0 | 0 | 0 | 0 | 0 | 0 | 0 | 0 | 158 | 75 |
| *Moorea* | 0 | 0 | 0 | 0 | 0 | 0 | 0 | 0 | 0 | 0 | 0 | 160 | 0 |
| *Pseudanabaena* | 0 | 0 | 0 | 0 | 0 | 0 | 0 | 0 | 0 | 0 | 0 | 185 | 2105 |
| *Cyanothece* | 0 | 0 | 0 | 0 | 0 | 0 | 0 | 0 | 0 | 0 | 0 | 240 | 119 |
| *Arthrospira* | 0 | 0 | 0 | 0 | 0 | 0 | 0 | 0 | 0 | 0 | 0 | 350 | 42 |
| *Belnapia* | 0 | 0 | 0 | 0 | 0 | 0 | 0 | 0 | 0 | 0 | 0 | 618 | 153 |
| *Candidatus Pelagibacter* | 0 | 0 | 0 | 0 | 0 | 0 | 0 | 0 | 0 | 0 | 0 | 1085 | 320 |
| *Prochlorothrix* | 0 | 0 | 0 | 0 | 0 | 0 | 0 | 0 | 0 | 0 | 0 | 1371 | 2672 |
| *Elusimicrobium* | 27 | 0 | 0 | 0 | 0 | 20 | 0 | 0 | 0 | 0 | 0 | 0 | 0 |
| *Hydrogenimonas* | 0 | 0 | 0 | 0 | 0 | 21 | 0 | 0 | 0 | 0 | 0 | 0 | 0 |
| *Propionispira* | 24 | 24 | 0 | 0 | 0 | 23 | 0 | 0 | 0 | 0 | 0 | 0 | 0 |
| *Erysipelatoclostridium* | 0 | 23 | 605 | 38 | 0 | 25 | 0 | 0 | 0 | 0 | 0 | 0 | 0 |
| *Sunxiuqinia* | 0 | 0 | 0 | 0 | 0 | 30 | 0 | 0 | 0 | 0 | 0 | 0 | 0 |
| *Acetobacterium* | 0 | 0 | 0 | 0 | 0 | 31 | 0 | 0 | 0 | 0 | 0 | 0 | 0 |
| *Prevotellamassilia* | 0 | 62 | 24 | 40 | 30 | 32 | 0 | 0 | 0 | 0 | 0 | 0 | 0 |
| *Hungatella* | 35 | 0 | 75 | 69 | 40 | 32 | 0 | 0 | 0 | 0 | 0 | 0 | 0 |
| *Draconibacterium* | 0 | 0 | 0 | 0 | 0 | 33 | 0 | 0 | 0 | 0 | 0 | 0 | 0 |
| *Tannerella* | 0 | 21 | 40 | 0 | 0 | 34 | 0 | 0 | 0 | 0 | 0 | 0 | 0 |
| *Actinomyces* | 125 | 39 | 90 | 0 | 0 | 37 | 0 | 0 | 0 | 0 | 0 | 0 | 0 |
| *Eisenbergiella* | 0 | 38 | 81 | 56 | 35 | 38 | 0 | 0 | 0 | 0 | 0 | 0 | 0 |
| *Anaerotruncus* | 32 | 0 | 45 | 50 | 0 | 38 | 0 | 0 | 0 | 0 | 0 | 0 | 0 |
| *Helicobacter* | 0 | 0 | 0 | 0 | 0 | 39 | 0 | 0 | 0 | 0 | 0 | 0 | 0 |
| *Ilyobacter* | 0 | 0 | 0 | 0 | 0 | 41 | 0 | 0 | 0 | 0 | 0 | 0 | 0 |
| *Sebaldella* | 112 | 22 | 0 | 0 | 0 | 42 | 0 | 0 | 0 | 0 | 0 | 0 | 0 |
| *Barnesiella* | 0 | 45 | 72 | 118 | 0 | 43 | 0 | 0 | 0 | 0 | 0 | 0 | 0 |
| *Citrobacter* | 151 | 91 | 264 | 42 | 162 | 43 | 0 | 0 | 0 | 0 | 0 | 0 | 0 |
| *Romboutsia* | 0 | 100 | 0 | 136 | 0 | 45 | 0 | 0 | 0 | 0 | 0 | 0 | 0 |
| *Porphyromonas* | 0 | 28 | 0 | 0 | 30 | 47 | 0 | 0 | 0 | 0 | 0 | 0 | 0 |
| *Ruthenibacterium* | 41 | 54 | 226 | 68 | 0 | 47 | 0 | 0 | 0 | 0 | 0 | 0 | 0 |
| *Fusibacter* | 0 | 0 | 0 | 0 | 0 | 49 | 0 | 0 | 0 | 0 | 0 | 0 | 0 |
| *Prolixibacter* | 0 | 0 | 0 | 0 | 0 | 52 | 0 | 0 | 0 | 0 | 0 | 0 | 0 |
| *Pleomorphomonas* | 141 | 297 | 117 | 77 | 95 | 52 | 0 | 0 | 0 | 0 | 0 | 0 | 0 |
| *Leucobacter* | 429 | 171 | 64 | 0 | 0 | 57 | 0 | 0 | 0 | 0 | 0 | 0 | 0 |
| *Coprobacillus* | 212 | 183 | 333 | 158 | 30 | 67 | 0 | 0 | 0 | 0 | 0 | 0 | 0 |
| *Formivibrio* | 781 | 511 | 0 | 0 | 29 | 67 | 0 | 0 | 0 | 0 | 0 | 0 | 0 |
| *Flavonifractor* | 86 | 97 | 210 | 239 | 106 | 70 | 0 | 0 | 0 | 0 | 0 | 0 | 0 |
| *Paraprevotella* | 31 | 22 | 0 | 72 | 75 | 71 | 0 | 0 | 0 | 0 | 0 | 0 | 0 |
| *Butyricimonas* | 37 | 141 | 42 | 127 | 87 | 83 | 0 | 0 | 0 | 0 | 0 | 0 | 0 |
| *Eggerthella* | 73 | 59 | 69 | 93 | 35 | 86 | 0 | 0 | 0 | 0 | 0 | 0 | 0 |
| *Aminomonas* | 0 | 0 | 0 | 0 | 0 | 91 | 0 | 0 | 0 | 0 | 0 | 0 | 0 |
| *Cloacibacillus* | 0 | 114 | 0 | 0 | 0 | 91 | 0 | 0 | 0 | 0 | 0 | 0 | 0 |
| *Geofilum* | 0 | 0 | 0 | 0 | 0 | 97 | 0 | 0 | 0 | 0 | 0 | 0 | 0 |
| *Tessaracoccus* | 471 | 138 | 249 | 75 | 0 | 109 | 0 | 0 | 0 | 0 | 0 | 0 | 0 |
| *Desulfomicrobium* | 0 | 0 | 0 | 0 | 0 | 113 | 0 | 0 | 0 | 0 | 0 | 0 | 0 |
| *Butyricicoccus* | 65 | 91 | 203 | 208 | 64 | 116 | 0 | 0 | 0 | 0 | 0 | 0 | 0 |
| *Butyrivibrio* | 40 | 64 | 54 | 85 | 0 | 118 | 0 | 0 | 0 | 0 | 0 | 0 | 0 |
| *Tyzzerella* | 0 | 76 | 124 | 467 | 111 | 120 | 0 | 0 | 0 | 0 | 0 | 0 | 0 |
| *Dehalobacter* | 123 | 0 | 194 | 0 | 0 | 127 | 0 | 0 | 0 | 0 | 0 | 0 | 0 |
| *Enterococcus* | 531 | 243 | 1985 | 211 | 448 | 128 | 0 | 0 | 0 | 0 | 0 | 0 | 0 |
| *Sporomusa* | 246 | 0 | 0 | 0 | 0 | 129 | 0 | 0 | 0 | 0 | 0 | 0 | 0 |
| *Actinobacillus* | 0 | 0 | 0 | 0 | 0 | 135 | 0 | 0 | 0 | 0 | 0 | 0 | 0 |
| *Mitsuokella* | 57 | 635 | 21 | 100 | 274 | 136 | 0 | 0 | 0 | 0 | 0 | 0 | 0 |
| *Pelosinus* | 0 | 0 | 0 | 0 | 0 | 137 | 0 | 0 | 0 | 0 | 0 | 0 | 0 |
| *Dysgonomonas* | 194 | 233 | 57 | 87 | 39 | 145 | 0 | 0 | 0 | 0 | 0 | 0 | 0 |
| *Sulfurovum* | 49 | 46 | 23 | 29 | 44 | 159 | 0 | 0 | 0 | 0 | 0 | 0 | 0 |
| *Proteocatella* | 0 | 0 | 0 | 0 | 0 | 171 | 0 | 0 | 0 | 0 | 0 | 0 | 0 |
| *Bilophila* | 116 | 213 | 414 | 333 | 137 | 174 | 0 | 0 | 0 | 0 | 0 | 0 | 0 |
| *Anaerostipes* | 78 | 273 | 67 | 453 | 202 | 184 | 0 | 0 | 0 | 0 | 0 | 0 | 0 |
| *Lachnospira* | 69 | 307 | 0 | 123 | 45 | 203 | 0 | 0 | 0 | 0 | 0 | 0 | 0 |
| *Akkermansia* | 173 | 86 | 218 | 366 | 1444 | 217 | 0 | 0 | 0 | 0 | 0 | 0 | 0 |
| *Sulfurimonas* | 90 | 76 | 67 | 40 | 64 | 226 | 0 | 0 | 0 | 0 | 0 | 0 | 0 |
| *Acidaminococcus* | 471 | 482 | 1034 | 965 | 752 | 245 | 0 | 0 | 0 | 0 | 0 | 0 | 0 |
| *Lachnoclostridium* | 176 | 181 | 1982 | 427 | 665 | 255 | 0 | 0 | 0 | 0 | 0 | 0 | 0 |
| *Succinatimonas* | 694 | 47 | 61 | 223 | 163 | 367 | 0 | 0 | 0 | 0 | 0 | 0 | 0 |
| *Oscillibacter* | 193 | 445 | 1884 | 1101 | 229 | 372 | 0 | 0 | 0 | 0 | 0 | 0 | 0 |
| *Sutterella* | 335 | 742 | 868 | 1689 | 701 | 383 | 0 | 0 | 0 | 0 | 0 | 0 | 0 |
| *Laribacter* | 343 | 830 | 63 | 158 | 76 | 385 | 0 | 0 | 0 | 0 | 0 | 0 | 0 |
| *Dialister* | 325 | 939 | 76 | 679 | 486 | 465 | 0 | 0 | 0 | 0 | 0 | 0 | 0 |
| *Catenibacterium* | 121 | 912 | 598 | 987 | 548 | 476 | 0 | 0 | 0 | 0 | 0 | 0 | 0 |
| *Leptotrichia* | 245 | 222 | 145 | 233 | 0 | 478 | 0 | 0 | 0 | 0 | 0 | 0 | 0 |
| *Coprococcus* | 147 | 837 | 311 | 971 | 346 | 568 | 0 | 0 | 0 | 0 | 0 | 0 | 0 |
| *Holdemanella* | 167 | 457 | 218 | 712 | 611 | 584 | 0 | 0 | 0 | 0 | 0 | 0 | 0 |
| *Phascolarctobacterium* | 512 | 634 | 781 | 946 | 621 | 599 | 0 | 0 | 0 | 0 | 0 | 0 | 0 |
| *Haemophilus* | 83 | 103 | 0 | 78 | 0 | 949 | 0 | 0 | 0 | 0 | 0 | 0 | 0 |
| *Subdoligranulum* | 640 | 882 | 704 | 1583 | 915 | 965 | 0 | 0 | 0 | 0 | 0 | 0 | 0 |
| *Fusicatenibacter* | 731 | 1067 | 1154 | 1424 | 721 | 1151 | 0 | 0 | 0 | 0 | 0 | 0 | 0 |
| *Dorea* | 465 | 1710 | 1328 | 2899 | 729 | 1288 | 0 | 0 | 0 | 0 | 0 | 0 | 0 |
| *Collinsella* | 967 | 1395 | 1253 | 1759 | 1095 | 1368 | 0 | 0 | 0 | 0 | 0 | 0 | 0 |
| *Acetoanaerobium* | 121 | 66 | 0 | 0 | 0 | 1392 | 0 | 0 | 0 | 0 | 0 | 0 | 0 |
| *Blautia* | 1182 | 2593 | 6072 | 5768 | 2924 | 3322 | 0 | 0 | 0 | 0 | 0 | 0 | 0 |
| *Methylogaea* | 0 | 0 | 0 | 0 | 0 | 0 | 0 | 0 | 0 | 46 | 0 | 0 | 0 |
| *Hydrobacter* | 0 | 0 | 0 | 0 | 0 | 0 | 0 | 0 | 0 | 47 | 0 | 0 | 0 |
| *Methylocaldum* | 0 | 0 | 0 | 0 | 0 | 0 | 0 | 0 | 0 | 49 | 0 | 0 | 0 |
| *Methylovorus* | 0 | 0 | 0 | 0 | 0 | 0 | 0 | 0 | 0 | 59 | 0 | 0 | 0 |
| *Methylococcus* | 0 | 0 | 0 | 0 | 0 | 0 | 0 | 0 | 0 | 66 | 0 | 0 | 0 |
| *Methylomarinum* | 0 | 0 | 0 | 0 | 0 | 0 | 0 | 0 | 0 | 77 | 0 | 0 | 0 |
| *Aquincola* | 0 | 0 | 0 | 0 | 0 | 0 | 0 | 0 | 0 | 83 | 0 | 0 | 0 |
| *Rubellimicrobium* | 0 | 0 | 0 | 0 | 0 | 0 | 0 | 0 | 0 | 92 | 0 | 0 | 0 |
| *Brachymonas* | 506 | 902 | 81 | 145 | 490 | 330 | 0 | 0 | 0 | 93 | 0 | 0 | 0 |
| *Methylovulum* | 0 | 0 | 0 | 0 | 0 | 0 | 0 | 0 | 0 | 135 | 0 | 0 | 0 |
| *Limnothrix* | 0 | 0 | 0 | 0 | 0 | 0 | 0 | 0 | 0 | 146 | 0 | 0 | 1681 |
| *Beggiatoa* | 0 | 0 | 0 | 0 | 0 | 0 | 0 | 0 | 0 | 154 | 0 | 0 | 0 |
| *Planktothrix* | 0 | 0 | 0 | 0 | 0 | 0 | 0 | 0 | 0 | 214 | 0 | 0 | 72 |
| *Rubritalea* | 0 | 0 | 0 | 0 | 0 | 0 | 0 | 0 | 33 | 0 | 0 | 0 | 0 |
| *Asticcacaulis* | 0 | 0 | 0 | 0 | 0 | 0 | 0 | 0 | 33 | 0 | 0 | 54 | 0 |
| *Owenweeksia* | 0 | 0 | 0 | 0 | 0 | 0 | 0 | 0 | 42 | 0 | 0 | 0 | 0 |
| *Lentimicrobium* | 0 | 0 | 0 | 0 | 0 | 37 | 0 | 0 | 42 | 0 | 0 | 0 | 0 |
| *Sandarakinorhabdus* | 0 | 0 | 0 | 0 | 0 | 0 | 0 | 0 | 46 | 128 | 0 | 0 | 0 |
| *Beijerinckia* | 0 | 0 | 0 | 0 | 0 | 0 | 0 | 0 | 49 | 103 | 0 | 0 | 88 |
| *Mariniradius* | 0 | 0 | 0 | 0 | 0 | 0 | 0 | 0 | 51 | 0 | 0 | 0 | 0 |
| *Immundisolibacter* | 0 | 0 | 0 | 0 | 0 | 0 | 0 | 0 | 54 | 28 | 0 | 0 | 206 |
| *Arenimonas* | 0 | 0 | 0 | 0 | 0 | 0 | 0 | 0 | 58 | 0 | 0 | 0 | 0 |
| *Rhodoplanes* | 0 | 0 | 0 | 0 | 0 | 0 | 0 | 0 | 63 | 0 | 0 | 0 | 0 |
| *Rhodonellum* | 0 | 0 | 0 | 0 | 0 | 0 | 0 | 0 | 73 | 0 | 0 | 0 | 0 |
| *Candidatus Aquiluna* | 0 | 0 | 0 | 0 | 0 | 0 | 0 | 0 | 78 | 0 | 0 | 143 | 266 |
| *Methylobacillus* | 0 | 0 | 0 | 0 | 0 | 0 | 0 | 0 | 80 | 89 | 0 | 0 | 0 |
| *Xylophilus* | 0 | 0 | 0 | 0 | 0 | 0 | 0 | 0 | 83 | 100 | 0 | 0 | 0 |
| *Lishizhenia* | 0 | 0 | 0 | 0 | 0 | 0 | 0 | 0 | 83 | 189 | 0 | 121 | 86 |
| *Hirschia* | 0 | 0 | 0 | 0 | 0 | 0 | 0 | 0 | 94 | 0 | 0 | 0 | 0 |
| *Scytonema* | 0 | 0 | 0 | 0 | 0 | 0 | 0 | 0 | 94 | 0 | 0 | 106 | 0 |
| *Hyphomonas* | 0 | 0 | 0 | 0 | 0 | 0 | 0 | 0 | 94 | 0 | 0 | 188 | 0 |
| *Pseudacidovorax* | 0 | 0 | 0 | 0 | 0 | 0 | 0 | 0 | 112 | 98 | 0 | 0 | 139 |
| *Pseudorhodobacter* | 0 | 0 | 0 | 0 | 0 | 0 | 0 | 0 | 134 | 0 | 0 | 0 | 0 |
| *Ruegeria* | 0 | 0 | 0 | 0 | 0 | 0 | 0 | 0 | 168 | 98 | 0 | 0 | 0 |
| *Caenimonas* | 0 | 0 | 0 | 0 | 0 | 0 | 0 | 0 | 170 | 170 | 0 | 0 | 0 |
| *Planktothricoides* | 0 | 0 | 0 | 0 | 0 | 0 | 0 | 0 | 199 | 447 | 0 | 0 | 999 |
| *Altererythrobacter* | 0 | 0 | 0 | 0 | 0 | 0 | 0 | 0 | 207 | 378 | 0 | 0 | 0 |
| *Noviherbaspirillum* | 0 | 0 | 0 | 0 | 0 | 0 | 0 | 0 | 487 | 348 | 0 | 0 | 0 |
| *Arsukibacterium* | 0 | 0 | 0 | 0 | 0 | 0 | 0 | 0 | 0 | 0 | 24 | 0 | 0 |
| *Lactococcus* | 0 | 0 | 23 | 33 | 0 | 35 | 0 | 0 | 0 | 0 | 24 | 0 | 0 |
| *Azotobacter* | 0 | 0 | 0 | 0 | 0 | 0 | 0 | 0 | 0 | 0 | 29 | 0 | 0 |
| *Leuconostoc* | 0 | 0 | 339 | 0 | 0 | 0 | 0 | 0 | 0 | 0 | 32 | 0 | 0 |
| *Ferrovum* | 0 | 0 | 0 | 0 | 0 | 0 | 0 | 0 | 76 | 110 | 32 | 74 | 45 |
| *Halobacteriovorax* | 0 | 0 | 0 | 0 | 0 | 0 | 0 | 0 | 47 | 286 | 33 | 0 | 0 |
| *Chroococcidiopsis* | 0 | 0 | 0 | 0 | 0 | 0 | 0 | 0 | 163 | 0 | 35 | 0 | 0 |
| *Acidimicrobium* | 0 | 0 | 0 | 0 | 0 | 0 | 0 | 0 | 39 | 0 | 36 | 1856 | 3035 |
| *Hylemonella* | 0 | 0 | 0 | 0 | 0 | 0 | 0 | 0 | 272 | 93 | 47 | 0 | 0 |
| *Riemerella* | 41 | 41 | 0 | 34 | 43 | 63 | 0 | 0 | 0 | 0 | 51 | 0 | 0 |
| *Odoribacter* | 87 | 196 | 167 | 384 | 314 | 134 | 0 | 0 | 0 | 0 | 52 | 0 | 0 |
| *Filimonas* | 0 | 0 | 0 | 0 | 0 | 0 | 0 | 0 | 95 | 78 | 57 | 0 | 0 |
| *Haematospirillum* | 0 | 0 | 0 | 0 | 0 | 0 | 0 | 0 | 0 | 0 | 62 | 0 | 0 |
| *Saprospira* | 0 | 0 | 0 | 0 | 0 | 0 | 0 | 0 | 0 | 0 | 63 | 1299 | 0 |
| *Alteromonas* | 0 | 0 | 0 | 0 | 0 | 0 | 0 | 0 | 0 | 0 | 79 | 0 | 0 |
| *Faecalibacterium* | 3475 | 6257 | 4694 | 7101 | 4583 | 3758 | 0 | 0 | 0 | 0 | 82 | 0 | 0 |
| *Pseudoxanthomonas* | 0 | 0 | 96 | 0 | 0 | 169 | 0 | 0 | 58 | 0 | 90 | 0 | 0 |
| *Novispirillum* | 0 | 0 | 0 | 0 | 58 | 0 | 0 | 0 | 0 | 0 | 95 | 0 | 0 |
| *Sulfuricurvum* | 40 | 60 | 0 | 56 | 0 | 217 | 0 | 0 | 68 | 0 | 101 | 0 | 0 |
| *Selenomonas* | 71 | 103 | 20 | 69 | 40 | 217 | 0 | 0 | 0 | 0 | 129 | 0 | 0 |
| *Elizabethkingia* | 179 | 165 | 236 | 194 | 1831 | 162 | 0 | 0 | 0 | 0 | 138 | 0 | 0 |
| *Nevskia* | 0 | 0 | 0 | 0 | 0 | 0 | 0 | 0 | 0 | 0 | 140 | 0 | 0 |
| *Rhodoluna* | 0 | 0 | 0 | 0 | 0 | 0 | 0 | 0 | 925 | 0 | 142 | 1340 | 272 |
| *Tepidimonas* | 0 | 0 | 87 | 0 | 0 | 0 | 0 | 0 | 456 | 237 | 151 | 0 | 182 |
| *Roseomonas* | 0 | 0 | 0 | 0 | 0 | 0 | 0 | 0 | 150 | 170 | 163 | 217 | 204 |
| *Dolichospermum* | 0 | 0 | 0 | 0 | 0 | 0 | 0 | 0 | 0 | 0 | 169 | 0 | 0 |
| *Limnobacter* | 0 | 0 | 0 | 0 | 0 | 0 | 0 | 0 | 0 | 0 | 176 | 0 | 0 |
| *Mitsuaria* | 0 | 0 | 0 | 86 | 0 | 0 | 0 | 0 | 243 | 118 | 178 | 0 | 0 |
| *Vogesella* | 0 | 0 | 0 | 0 | 34 | 0 | 0 | 0 | 0 | 0 | 203 | 0 | 0 |
| *Roseburia* | 1717 | 3835 | 4465 | 3611 | 4336 | 3663 | 0 | 0 | 0 | 0 | 205 | 0 | 0 |
| *Erythrobacter* | 0 | 0 | 0 | 0 | 0 | 0 | 0 | 0 | 202 | 293 | 234 | 164 | 118 |
| *Blastomonas* | 0 | 0 | 0 | 0 | 0 | 0 | 0 | 0 | 49 | 90 | 236 | 0 | 0 |
| *Pseudarcicella* | 0 | 0 | 0 | 0 | 0 | 0 | 0 | 0 | 1025 | 287 | 237 | 0 | 228 |
| *Cylindrospermopsis* | 0 | 0 | 0 | 0 | 0 | 0 | 0 | 0 | 101 | 0 | 290 | 306 | 3160 |
| *Shewanella* | 34 | 32 | 0 | 0 | 49 | 49 | 0 | 0 | 0 | 48 | 293 | 0 | 0 |
| *Methylophilus* | 0 | 0 | 0 | 0 | 0 | 0 | 0 | 0 | 1313 | 1888 | 338 | 0 | 63 |
| *Candidatus Methylopumilus* | 0 | 0 | 0 | 0 | 0 | 0 | 0 | 0 | 1274 | 1905 | 376 | 1888 | 3595 |
| *Emticicia* | 0 | 0 | 0 | 0 | 0 | 0 | 0 | 0 | 646 | 902 | 440 | 189 | 315 |
| *Silanimonas* | 0 | 0 | 0 | 0 | 0 | 0 | 0 | 0 | 747 | 233 | 449 | 0 | 0 |
| *Aquaspirillum* | 217 | 128 | 59 | 151 | 1527 | 1028 | 0 | 0 | 0 | 0 | 503 | 0 | 0 |
| *Methylotenera* | 0 | 0 | 0 | 0 | 0 | 0 | 0 | 0 | 1586 | 2221 | 522 | 40 | 179 |
| *Alishewanella* | 0 | 0 | 0 | 0 | 0 | 0 | 0 | 0 | 239 | 84 | 534 | 0 | 0 |
| *Porphyrobacter* | 0 | 0 | 0 | 0 | 0 | 0 | 0 | 0 | 399 | 500 | 711 | 822 | 229 |
| *Gemmobacter* | 0 | 0 | 0 | 0 | 0 | 0 | 0 | 0 | 235 | 277 | 809 | 0 | 0 |
| *Cellvibrio* | 0 | 0 | 53 | 0 | 29 | 0 | 0 | 0 | 122 | 125 | 820 | 0 | 0 |
| *Anabaena* | 0 | 0 | 0 | 0 | 0 | 0 | 0 | 0 | 0 | 151 | 884 | 126 | 946 |
| *Flectobacillus* | 0 | 0 | 0 | 0 | 0 | 0 | 0 | 0 | 4902 | 2643 | 3984 | 155 | 434 |
| *Rheinheimera* | 0 | 0 | 58 | 0 | 76 | 0 | 0 | 0 | 1463 | 1247 | 11654 | 206 | 227 |
| *Candidatus Moduliflexus* | 0 | 0 | 0 | 0 | 0 | 0 | 0 | 23 | 0 | 0 | 0 | 0 | 0 |
| *Candidatus Magnetobacterium* | 0 | 0 | 0 | 0 | 0 | 0 | 0 | 24 | 0 | 0 | 0 | 0 | 0 |
| *Fusobacterium* | 44 | 180 | 122 | 335 | 168 | 296 | 0 | 35 | 0 | 0 | 0 | 0 | 0 |
| *Veillonella* | 89 | 39 | 133 | 55 | 110 | 231 | 0 | 37 | 0 | 0 | 0 | 0 | 0 |
| *Megasphaera* | 594 | 1660 | 1122 | 1739 | 949 | 546 | 0 | 37 | 0 | 0 | 0 | 0 | 0 |
| *Gloeobacter* | 0 | 0 | 0 | 0 | 0 | 0 | 0 | 41 | 0 | 0 | 0 | 0 | 0 |
| *Candidatus Izimaplasma* | 0 | 0 | 0 | 0 | 0 | 0 | 0 | 43 | 0 | 0 | 0 | 0 | 0 |
| *Nocardiopsis* | 0 | 0 | 0 | 0 | 0 | 0 | 0 | 45 | 0 | 0 | 0 | 0 | 0 |
| *Bartonella* | 0 | 0 | 0 | 0 | 0 | 0 | 0 | 47 | 0 | 0 | 0 | 0 | 0 |
| *Syntrophus* | 0 | 0 | 0 | 0 | 0 | 75 | 0 | 49 | 0 | 0 | 0 | 0 | 0 |
| *Isosphaera* | 0 | 0 | 0 | 0 | 0 | 0 | 0 | 50 | 0 | 0 | 0 | 0 | 43 |
| *Candidatus Scalindua* | 0 | 0 | 0 | 0 | 0 | 0 | 0 | 54 | 0 | 0 | 0 | 0 | 0 |
| *Phaeospirillum* | 0 | 0 | 0 | 0 | 0 | 0 | 0 | 55 | 0 | 0 | 0 | 0 | 0 |
| *Salinispora* | 0 | 0 | 0 | 0 | 0 | 0 | 0 | 57 | 0 | 0 | 0 | 0 | 0 |
| *Corynebacterium* | 33 | 0 | 0 | 0 | 0 | 0 | 0 | 57 | 0 | 0 | 0 | 0 | 0 |
| *Planctopirus* | 0 | 0 | 0 | 0 | 0 | 0 | 0 | 57 | 0 | 0 | 0 | 0 | 298 |
| *Nitratireductor* | 0 | 0 | 0 | 0 | 0 | 0 | 0 | 58 | 0 | 0 | 0 | 0 | 0 |
| *Thalassospira* | 0 | 0 | 0 | 0 | 0 | 0 | 0 | 59 | 0 | 0 | 0 | 0 | 0 |
| *Microcystis* | 0 | 0 | 0 | 0 | 0 | 0 | 0 | 62 | 51 | 0 | 120 | 341 | 695 |
| *Ensifer* | 0 | 0 | 357 | 0 | 0 | 0 | 0 | 64 | 0 | 0 | 0 | 0 | 0 |
| *Desulfatibacillum* | 0 | 0 | 0 | 0 | 0 | 0 | 0 | 65 | 0 | 0 | 0 | 0 | 0 |
| *Desulforegula* | 0 | 0 | 0 | 0 | 0 | 145 | 0 | 65 | 0 | 0 | 0 | 0 | 0 |
| *Dehalococcoides* | 0 | 0 | 0 | 0 | 0 | 0 | 0 | 66 | 0 | 0 | 0 | 0 | 0 |
| *Zoogloea* | 0 | 0 | 0 | 0 | 0 | 0 | 0 | 66 | 0 | 0 | 0 | 0 | 0 |
| *Methyloceanibacter* | 0 | 0 | 0 | 0 | 0 | 0 | 0 | 67 | 0 | 0 | 0 | 0 | 0 |
| *Levilinea* | 0 | 0 | 0 | 0 | 0 | 0 | 0 | 68 | 0 | 0 | 0 | 0 | 0 |
| *Methylosinus* | 0 | 0 | 0 | 0 | 0 | 0 | 0 | 71 | 0 | 0 | 0 | 0 | 0 |
| *Candidatus Magnetomorum* | 0 | 0 | 0 | 0 | 0 | 0 | 0 | 74 | 0 | 0 | 0 | 0 | 138 |
| *Methylomicrobium* | 0 | 0 | 0 | 0 | 0 | 0 | 0 | 79 | 109 | 405 | 0 | 0 | 0 |
| *Rugosibacter* | 0 | 0 | 0 | 0 | 0 | 0 | 0 | 80 | 0 | 0 | 0 | 0 | 0 |
| *Microvirga* | 0 | 0 | 0 | 0 | 0 | 0 | 0 | 80 | 38 | 0 | 0 | 107 | 0 |
| *Aureimonas* | 0 | 0 | 0 | 0 | 0 | 0 | 0 | 81 | 0 | 0 | 0 | 0 | 0 |
| *Nonomuraea* | 0 | 0 | 0 | 0 | 0 | 0 | 0 | 81 | 0 | 0 | 0 | 0 | 0 |
| *Capnocytophaga* | 0 | 0 | 0 | 32 | 0 | 35 | 0 | 81 | 0 | 0 | 0 | 0 | 0 |
| *Pelagibacterium* | 0 | 0 | 0 | 0 | 0 | 0 | 0 | 82 | 0 | 0 | 0 | 0 | 0 |
| *Solimonas* | 0 | 0 | 0 | 0 | 0 | 0 | 0 | 82 | 0 | 0 | 0 | 0 | 0 |
| *Ruminiclostridium* | 55 | 56 | 86 | 211 | 74 | 88 | 0 | 83 | 0 | 0 | 0 | 0 | 0 |
| *Caballeronia* | 0 | 0 | 0 | 0 | 0 | 0 | 0 | 90 | 0 | 0 | 0 | 0 | 0 |
| *Ochrobactrum* | 153 | 112 | 65 | 0 | 347 | 0 | 0 | 91 | 0 | 0 | 0 | 0 | 0 |
| *Methylocystis* | 0 | 0 | 0 | 0 | 0 | 0 | 0 | 91 | 0 | 60 | 0 | 0 | 99 |
| *Herbidospora* | 0 | 0 | 0 | 0 | 0 | 0 | 0 | 92 | 0 | 0 | 0 | 0 | 0 |
| *Planctomicrobium* | 0 | 0 | 0 | 0 | 0 | 0 | 0 | 92 | 0 | 0 | 0 | 0 | 355 |
| *Bellilinea* | 0 | 0 | 0 | 0 | 0 | 0 | 0 | 94 | 0 | 0 | 0 | 0 | 0 |
| *Prosthecomicrobium* | 0 | 0 | 0 | 0 | 50 | 0 | 0 | 94 | 0 | 0 | 0 | 0 | 0 |
| *Trichodesmium* | 0 | 0 | 0 | 0 | 0 | 0 | 0 | 97 | 0 | 0 | 0 | 0 | 0 |
| *Halomonas* | 0 | 0 | 0 | 0 | 0 | 0 | 0 | 100 | 0 | 0 | 40 | 0 | 0 |
| *Marichromatium* | 0 | 0 | 0 | 0 | 0 | 0 | 0 | 106 | 0 | 0 | 0 | 0 | 0 |
| *Parabacteroides* | 513 | 1472 | 3268 | 1856 | 2483 | 1847 | 0 | 108 | 0 | 0 | 105 | 0 | 0 |
| *Pandoraea* | 113 | 90 | 46 | 53 | 65 | 0 | 0 | 110 | 0 | 92 | 0 | 0 | 0 |
| *Caulobacter* | 0 | 47 | 40 | 0 | 0 | 0 | 0 | 110 | 161 | 106 | 140 | 203 | 103 |
| *Thiorhodococcus* | 0 | 0 | 0 | 0 | 0 | 0 | 0 | 112 | 0 | 0 | 0 | 0 | 0 |
| *Sinorhizobium* | 0 | 0 | 662 | 0 | 0 | 0 | 0 | 115 | 0 | 0 | 0 | 0 | 0 |
| *Chelatococcus* | 0 | 0 | 0 | 0 | 0 | 0 | 0 | 116 | 0 | 0 | 0 | 0 | 0 |
| *Megamonas* | 790 | 643 | 921 | 535 | 1092 | 1655 | 0 | 116 | 0 | 0 | 0 | 0 | 0 |
| *Lamprocystis* | 0 | 0 | 0 | 0 | 0 | 0 | 0 | 118 | 0 | 0 | 0 | 0 | 0 |
| *Nitrobacter* | 0 | 0 | 0 | 0 | 0 | 0 | 0 | 120 | 0 | 0 | 0 | 0 | 0 |
| *Labrenzia* | 0 | 0 | 0 | 0 | 0 | 0 | 0 | 121 | 0 | 0 | 0 | 0 | 0 |
| *Sulfitobacter* | 0 | 0 | 0 | 0 | 0 | 0 | 0 | 121 | 0 | 0 | 0 | 0 | 0 |
| *Limnohabitans* | 0 | 0 | 0 | 0 | 0 | 0 | 0 | 121 | 25739 | 21011 | 3366 | 10041 | 12358 |
| *Geodermatophilus* | 0 | 0 | 0 | 0 | 0 | 0 | 0 | 124 | 0 | 0 | 0 | 0 | 0 |
| *Thiomonas* | 0 | 0 | 0 | 0 | 0 | 0 | 0 | 129 | 148 | 124 | 85 | 207 | 0 |
| *Rhodobacter* | 0 | 105 | 0 | 0 | 0 | 0 | 0 | 130 | 1758 | 3224 | 1410 | 816 | 1285 |
| *Rhodopseudomonas* | 0 | 0 | 0 | 0 | 198 | 0 | 0 | 136 | 0 | 0 | 0 | 122 | 0 |
| *Neisseria* | 2354 | 1242 | 1979 | 2273 | 1601 | 509 | 0 | 136 | 0 | 0 | 0 | 0 | 0 |
| *Paucibacter* | 0 | 0 | 0 | 63 | 51 | 0 | 0 | 138 | 154 | 183 | 147 | 0 | 0 |
| *Rhodovulum* | 0 | 0 | 0 | 0 | 0 | 0 | 0 | 146 | 0 | 0 | 0 | 0 | 0 |
| *Giesbergeria* | 98 | 0 | 65 | 0 | 100 | 119 | 0 | 157 | 154 | 175 | 85 | 0 | 0 |
| *Acidiphilium* | 0 | 0 | 0 | 0 | 0 | 0 | 0 | 166 | 0 | 0 | 0 | 0 | 0 |
| *Azorhizobium* | 0 | 0 | 0 | 0 | 0 | 0 | 0 | 167 | 0 | 0 | 0 | 0 | 0 |
| *Rhodanobacter* | 0 | 0 | 0 | 0 | 0 | 0 | 0 | 170 | 0 | 0 | 0 | 0 | 0 |
| *Gluconacetobacter* | 0 | 0 | 0 | 0 | 0 | 0 | 0 | 183 | 0 | 0 | 0 | 0 | 0 |
| *Pseudogulbenkiania* | 0 | 0 | 0 | 0 | 0 | 0 | 0 | 222 | 0 | 0 | 0 | 0 | 0 |
| *Macellibacteroides* | 118 | 157 | 0 | 26 | 21 | 2592 | 0 | 230 | 0 | 0 | 219 | 0 | 0 |
| *Desulfotomaculum* | 0 | 0 | 0 | 0 | 0 | 0 | 0 | 231 | 0 | 0 | 0 | 0 | 0 |
| *Rhizobacter* | 0 | 0 | 0 | 0 | 0 | 0 | 0 | 249 | 166 | 189 | 89 | 0 | 0 |
| *Escherichia* | 527 | 1566 | 503 | 1485 | 2151 | 1844 | 0 | 282 | 0 | 0 | 112 | 0 | 0 |
| *Candidatus Cloacimonas* | 0 | 0 | 0 | 0 | 0 | 761 | 0 | 309 | 0 | 0 | 0 | 0 | 0 |
| *Chromobacterium* | 0 | 0 | 78 | 0 | 126 | 0 | 0 | 334 | 0 | 0 | 41 | 0 | 0 |
| *Thermus* | 0 | 0 | 0 | 0 | 0 | 0 | 24 | 58 | 0 | 0 | 0 | 0 | 0 |
| *Meiothermus* | 0 | 0 | 0 | 0 | 0 | 0 | 25 | 41 | 0 | 0 | 0 | 0 | 0 |
| *Acidithiobacillus* | 41 | 28 | 32 | 34 | 60 | 29 | 28 | 58 | 51 | 72 | 28 | 0 | 0 |
| *Campylobacter* | 53 | 54 | 29 | 32 | 32 | 140 | 30 | 55 | 0 | 0 | 0 | 0 | 0 |
| *Candidatus Entotheonella* | 0 | 0 | 0 | 0 | 0 | 0 | 35 | 40 | 28 | 0 | 0 | 0 | 28 |
| *Syntrophorhabdus* | 0 | 0 | 0 | 0 | 0 | 116 | 35 | 42 | 0 | 0 | 0 | 0 | 0 |
| *Nakamurella* | 0 | 0 | 30 | 0 | 0 | 0 | 38 | 61 | 0 | 0 | 0 | 0 | 0 |
| *Fimbriimonas* | 0 | 0 | 0 | 0 | 0 | 0 | 39 | 29 | 0 | 0 | 0 | 1044 | 0 |
| *Bryobacter* | 0 | 0 | 0 | 0 | 0 | 0 | 41 | 46 | 34 | 0 | 0 | 51 | 66 |
| *Brevundimonas* | 80 | 47 | 0 | 0 | 0 | 0 | 42 | 87 | 44 | 58 | 37 | 58 | 0 |
| *Ktedonobacter* | 0 | 0 | 0 | 0 | 0 | 0 | 43 | 47 | 0 | 0 | 0 | 0 | 0 |
| *Sulfurospirillum* | 1985 | 1605 | 45 | 59 | 36 | 2348 | 45 | 343 | 0 | 0 | 162 | 0 | 0 |
| *Occidentia* | 0 | 0 | 0 | 0 | 0 | 0 | 46 | 0 | 0 | 0 | 0 | 0 | 0 |
| *Chlorobium* | 0 | 0 | 0 | 0 | 0 | 0 | 46 | 61 | 52 | 67 | 0 | 66 | 56 |
| *Desulfobacca* | 0 | 0 | 0 | 0 | 0 | 0 | 48 | 85 | 0 | 0 | 0 | 0 | 0 |
| *Smithella* | 0 | 0 | 0 | 0 | 0 | 552 | 48 | 171 | 63 | 0 | 0 | 0 | 0 |
| *Hahella* | 0 | 0 | 0 | 0 | 0 | 0 | 49 | 55 | 0 | 0 | 0 | 0 | 0 |
| *Thermoflexibacter* | 0 | 0 | 0 | 0 | 0 | 0 | 50 | 0 | 0 | 0 | 0 | 0 | 0 |
| *Desulfobulbus* | 0 | 0 | 32 | 0 | 0 | 154 | 52 | 71 | 0 | 38 | 0 | 0 | 0 |
| *Nitrosovibrio* | 0 | 0 | 0 | 0 | 0 | 0 | 52 | 85 | 0 | 0 | 0 | 0 | 0 |
| *Crocinitomix* | 0 | 0 | 0 | 0 | 0 | 0 | 53 | 0 | 148 | 193 | 0 | 297 | 102 |
| *Haloferula* | 0 | 0 | 0 | 0 | 0 | 0 | 53 | 0 | 941 | 127 | 0 | 136 | 112 |
| *Endozoicomonas* | 0 | 0 | 0 | 0 | 0 | 0 | 53 | 57 | 0 | 0 | 0 | 0 | 0 |
| *Lactobacillus* | 209 | 802 | 2558 | 408 | 1912 | 717 | 53 | 72 | 0 | 0 | 187 | 0 | 0 |
| *Roseimaritima* | 0 | 0 | 0 | 0 | 0 | 0 | 54 | 0 | 0 | 0 | 0 | 0 | 276 |
| *Rubinisphaera* | 0 | 0 | 0 | 0 | 0 | 0 | 54 | 54 | 0 | 0 | 0 | 0 | 311 |
| *Pyrinomonas* | 0 | 0 | 0 | 0 | 0 | 0 | 54 | 74 | 31 | 0 | 0 | 91 | 91 |
| *Bacteriovorax* | 0 | 0 | 0 | 0 | 0 | 0 | 54 | 83 | 138 | 891 | 115 | 43 | 59 |
| *Plesiocystis* | 0 | 0 | 0 | 0 | 0 | 0 | 55 | 51 | 0 | 0 | 0 | 0 | 0 |
| *Enhygromyxa* | 0 | 0 | 0 | 0 | 0 | 0 | 55 | 67 | 0 | 0 | 0 | 0 | 0 |
| *Synechococcus* | 0 | 0 | 0 | 0 | 0 | 0 | 56 | 129 | 56 | 0 | 0 | 560 | 737 |
| *Moraxella* | 25 | 21 | 0 | 27 | 0 | 105 | 58 | 84 | 0 | 0 | 35 | 0 | 0 |
| *Phenylobacterium* | 0 | 0 | 38 | 0 | 0 | 59 | 58 | 135 | 787 | 990 | 103 | 1142 | 128 |
| *Microscilla* | 0 | 0 | 0 | 0 | 0 | 0 | 59 | 0 | 0 | 0 | 0 | 0 | 0 |
| *Pontibacter* | 0 | 0 | 0 | 0 | 0 | 0 | 59 | 0 | 40 | 0 | 0 | 80 | 0 |
| *Holospora* | 0 | 0 | 0 | 0 | 0 | 0 | 59 | 38 | 0 | 0 | 0 | 0 | 0 |
| *Azovibrio* | 110 | 75 | 666 | 72 | 48 | 56 | 61 | 66 | 0 | 0 | 46 | 0 | 0 |
| *Psychrobacter* | 0 | 0 | 0 | 0 | 0 | 0 | 62 | 0 | 0 | 0 | 52 | 0 | 0 |
| *Nitrolancea* | 0 | 0 | 0 | 0 | 0 | 0 | 63 | 70 | 0 | 0 | 0 | 0 | 0 |
| *Deinococcus* | 0 | 0 | 0 | 0 | 0 | 0 | 63 | 107 | 0 | 0 | 0 | 29 | 37 |
| *Kaistia* | 0 | 0 | 0 | 0 | 0 | 0 | 64 | 65 | 0 | 0 | 0 | 0 | 0 |
| *Alistipes* | 903 | 1659 | 1901 | 3269 | 1386 | 2019 | 64 | 89 | 0 | 0 | 190 | 0 | 0 |
| *Devosia* | 0 | 0 | 0 | 0 | 0 | 0 | 64 | 165 | 93 | 75 | 0 | 0 | 0 |
| *Gemmatirosa* | 0 | 0 | 0 | 0 | 0 | 0 | 65 | 0 | 31 | 0 | 0 | 0 | 0 |
| *Arachidicoccus* | 0 | 0 | 0 | 0 | 0 | 0 | 68 | 0 | 0 | 0 | 0 | 0 | 0 |
| *Bifidobacterium* | 6006 | 4738 | 3478 | 3657 | 3721 | 3708 | 70 | 92 | 0 | 0 | 0 | 0 | 0 |
| *Azonexus* | 346 | 196 | 63 | 44 | 112 | 93 | 70 | 118 | 34 | 41 | 68 | 0 | 0 |
| *Leadbetterella* | 0 | 0 | 0 | 0 | 0 | 0 | 71 | 0 | 173 | 103 | 104 | 178 | 173 |
| *Segetibacter* | 0 | 0 | 0 | 0 | 0 | 0 | 73 | 0 | 91 | 50 | 53 | 0 | 0 |
| *Sphingobacterium* | 28 | 32 | 26 | 22 | 30 | 34 | 74 | 67 | 67 | 38 | 74 | 0 | 0 |
| *Chloracidobacterium* | 0 | 0 | 0 | 0 | 0 | 0 | 75 | 51 | 0 | 0 | 0 | 35 | 37 |
| *Treponema* | 68 | 49 | 0 | 0 | 0 | 307 | 75 | 123 | 29 | 0 | 0 | 0 | 0 |
| *Magnetospirillum* | 213 | 255 | 91 | 128 | 53 | 61 | 75 | 169 | 75 | 153 | 76 | 244 | 102 |
| *Gimesia* | 0 | 0 | 0 | 0 | 0 | 0 | 78 | 65 | 65 | 0 | 0 | 0 | 755 |
| *Methylobacter* | 0 | 0 | 0 | 0 | 0 | 0 | 78 | 177 | 164 | 735 | 50 | 0 | 0 |
| *Tepidiphilus* | 0 | 186 | 291 | 46 | 41 | 0 | 79 | 78 | 0 | 0 | 0 | 0 | 0 |
| *Aeromonas* | 9048 | 5701 | 3051 | 238 | 6851 | 2901 | 79 | 309 | 51 | 67 | 2865 | 0 | 0 |
| *Stigmatella* | 0 | 0 | 0 | 0 | 0 | 0 | 81 | 167 | 0 | 0 | 0 | 0 | 0 |
| *Hyalangium* | 0 | 0 | 0 | 0 | 0 | 0 | 81 | 306 | 0 | 0 | 0 | 0 | 0 |
| *Parafilimonas* | 0 | 0 | 0 | 0 | 0 | 0 | 83 | 0 | 77 | 47 | 0 | 0 | 0 |
| *Sediminibacterium* | 0 | 0 | 0 | 0 | 0 | 0 | 83 | 0 | 1157 | 2042 | 472 | 186 | 355 |
| *Holophaga* | 0 | 87 | 0 | 0 | 0 | 0 | 83 | 48 | 0 | 0 | 0 | 0 | 0 |
| *Rufibacter* | 0 | 0 | 0 | 0 | 0 | 0 | 85 | 91 | 43 | 0 | 0 | 70 | 0 |
| *Derxia* | 0 | 0 | 0 | 0 | 0 | 0 | 85 | 222 | 0 | 0 | 0 | 0 | 0 |
| *Sporocytophaga* | 0 | 0 | 0 | 0 | 0 | 0 | 86 | 0 | 84 | 0 | 0 | 0 | 0 |
| *Phaeodactylibacter* | 0 | 0 | 0 | 0 | 0 | 0 | 86 | 75 | 0 | 0 | 24 | 278 | 99 |
| *Aromatoleum* | 0 | 0 | 0 | 0 | 31 | 0 | 86 | 89 | 0 | 0 | 36 | 0 | 0 |
| *Afipia* | 0 | 0 | 0 | 0 | 0 | 0 | 87 | 0 | 0 | 0 | 0 | 114 | 0 |
| *Bosea* | 163 | 82 | 290 | 103 | 483 | 0 | 87 | 161 | 56 | 0 | 0 | 155 | 0 |
| *Simkania* | 0 | 0 | 0 | 0 | 0 | 0 | 88 | 51 | 0 | 0 | 0 | 0 | 0 |
| *Gordonia* | 0 | 0 | 0 | 0 | 0 | 0 | 88 | 80 | 0 | 0 | 0 | 0 | 0 |
| *Thermoleophilum* | 0 | 0 | 0 | 0 | 0 | 29 | 89 | 0 | 0 | 0 | 0 | 0 | 0 |
| *Archangium* | 0 | 0 | 0 | 0 | 0 | 0 | 91 | 146 | 0 | 0 | 0 | 0 | 0 |
| *Terrimonas* | 0 | 0 | 0 | 0 | 0 | 0 | 92 | 0 | 0 | 0 | 0 | 0 | 0 |
| *Chthoniobacter* | 0 | 0 | 0 | 0 | 0 | 0 | 94 | 85 | 212 | 55 | 0 | 535 | 236 |
| *Aphanizomenon* | 0 | 0 | 0 | 0 | 0 | 0 | 94 | 262 | 86 | 0 | 581 | 1099 | 2175 |
| *Candidatus Paracaedibacter* | 0 | 0 | 0 | 0 | 0 | 0 | 95 | 63 | 0 | 0 | 0 | 0 | 0 |
| *Cytophaga* | 0 | 0 | 0 | 0 | 0 | 0 | 96 | 0 | 89 | 49 | 36 | 0 | 0 |
| *Roseiflexus* | 0 | 0 | 0 | 0 | 0 | 0 | 96 | 63 | 0 | 0 | 0 | 241 | 48 |
| *Caedibacter* | 0 | 0 | 0 | 0 | 0 | 0 | 97 | 63 | 0 | 0 | 0 | 91 | 66 |
| *Chloroflexus* | 0 | 0 | 0 | 0 | 0 | 0 | 98 | 99 | 0 | 0 | 0 | 57 | 150 |
| *Eubacterium* | 1094 | 3142 | 4145 | 3844 | 1128 | 2541 | 99 | 130 | 0 | 0 | 0 | 0 | 0 |
| *Leptolyngbya* | 0 | 0 | 0 | 0 | 0 | 0 | 99 | 159 | 110 | 0 | 59 | 1053 | 789 |
| *Alkanindiges* | 0 | 0 | 0 | 0 | 0 | 0 | 100 | 108 | 0 | 168 | 504 | 0 | 0 |
| *Alcanivorax* | 0 | 0 | 0 | 0 | 0 | 0 | 100 | 129 | 69 | 41 | 93 | 0 | 0 |
| *Amycolatopsis* | 0 | 0 | 0 | 0 | 0 | 0 | 100 | 252 | 0 | 0 | 0 | 96 | 90 |
| *Actinomadura* | 0 | 0 | 0 | 0 | 0 | 0 | 101 | 66 | 0 | 0 | 0 | 0 | 0 |
| *Pseudoalteromonas* | 27 | 0 | 0 | 0 | 0 | 0 | 101 | 75 | 0 | 34 | 197 | 82 | 0 |
| *Agrobacterium* | 0 | 0 | 137 | 0 | 115 | 0 | 101 | 145 | 0 | 0 | 0 | 0 | 0 |
| *Herpetosiphon* | 0 | 0 | 0 | 0 | 0 | 0 | 104 | 132 | 0 | 0 | 0 | 0 | 0 |
| *Opitutus* | 0 | 54 | 0 | 0 | 0 | 0 | 106 | 74 | 239 | 107 | 85 | 48 | 603 |
| *Candidatus Solibacter* | 0 | 0 | 0 | 0 | 0 | 0 | 107 | 72 | 38 | 0 | 0 | 128 | 70 |
| *Duganella* | 0 | 0 | 155 | 0 | 79 | 0 | 107 | 138 | 332 | 295 | 395 | 0 | 0 |
| *Chlamydia* | 94 | 162 | 66 | 157 | 105 | 128 | 109 | 116 | 0 | 0 | 0 | 0 | 0 |
| *Singulisphaera* | 0 | 0 | 0 | 0 | 0 | 0 | 110 | 64 | 0 | 0 | 0 | 0 | 98 |
| *Actinoplanes* | 0 | 0 | 0 | 0 | 0 | 0 | 110 | 133 | 0 | 0 | 0 | 0 | 0 |
| *Pseudonocardia* | 0 | 0 | 40 | 0 | 0 | 0 | 112 | 0 | 0 | 0 | 0 | 0 | 0 |
| *Cystobacter* | 0 | 0 | 0 | 0 | 0 | 0 | 112 | 285 | 0 | 0 | 0 | 0 | 0 |
| *Blastococcus* | 0 | 0 | 0 | 0 | 0 | 0 | 114 | 0 | 0 | 0 | 0 | 0 | 0 |
| *Aquimarina* | 0 | 0 | 0 | 0 | 0 | 0 | 114 | 81 | 81 | 0 | 46 | 0 | 164 |
| *Fluviicola* | 0 | 0 | 28 | 0 | 0 | 0 | 114 | 98 | 589 | 2282 | 114 | 1907 | 776 |
| *Collimonas* | 0 | 0 | 0 | 0 | 0 | 0 | 114 | 108 | 380 | 306 | 118 | 0 | 399 |
| *Tolumonas* | 640 | 698 | 64 | 36 | 681 | 1867 | 114 | 1013 | 0 | 0 | 193 | 0 | 0 |
| *Spirosoma* | 0 | 0 | 0 | 0 | 0 | 0 | 116 | 155 | 142 | 60 | 62 | 233 | 0 |
| *Cellulomonas* | 51 | 0 | 0 | 0 | 0 | 0 | 118 | 141 | 0 | 0 | 0 | 0 | 0 |
| *Geobacter* | 0 | 0 | 27 | 0 | 0 | 121 | 119 | 99 | 94 | 55 | 60 | 70 | 79 |
| *Sphingopyxis* | 0 | 0 | 144 | 86 | 0 | 0 | 120 | 92 | 265 | 490 | 201 | 79 | 608 |
| *Lysobacter* | 0 | 0 | 0 | 0 | 0 | 0 | 120 | 134 | 58 | 90 | 61 | 0 | 0 |
| *Nitrosospira* | 0 | 0 | 0 | 0 | 0 | 0 | 121 | 130 | 0 | 0 | 0 | 49 | 0 |
| *Dyadobacter* | 0 | 0 | 0 | 0 | 0 | 0 | 121 | 177 | 205 | 85 | 46 | 166 | 0 |
| *Mucilaginibacter* | 0 | 0 | 0 | 0 | 0 | 0 | 122 | 108 | 159 | 83 | 45 | 214 | 118 |
| *Azohydromonas* | 0 | 0 | 0 | 0 | 0 | 0 | 124 | 130 | 236 | 215 | 146 | 0 | 296 |
| *Marinobacterium* | 0 | 0 | 0 | 0 | 0 | 0 | 126 | 222 | 0 | 0 | 0 | 142 | 0 |
| *Intrasporangium* | 0 | 0 | 0 | 0 | 0 | 0 | 127 | 0 | 0 | 0 | 0 | 0 | 0 |
| *Rhodopirellula* | 0 | 0 | 0 | 0 | 0 | 0 | 129 | 106 | 121 | 0 | 0 | 197 | 540 |
| *Xanthomonas* | 111 | 122 | 82 | 48 | 72 | 0 | 129 | 130 | 0 | 0 | 67 | 0 | 0 |
| *Rhodococcus* | 0 | 0 | 0 | 0 | 0 | 0 | 129 | 157 | 0 | 0 | 0 | 61 | 39 |
| *Polynucleobacter* | 0 | 0 | 0 | 0 | 0 | 0 | 129 | 227 | 1786 | 1304 | 176 | 4171 | 2473 |
| *Methylomonas* | 54 | 47 | 36 | 0 | 0 | 0 | 131 | 152 | 3661 | 13296 | 268 | 207 | 167 |
| *Zavarzinella* | 0 | 0 | 0 | 0 | 0 | 0 | 131 | 210 | 0 | 0 | 0 | 24 | 83 |
| *Azospirillum* | 0 | 61 | 45 | 0 | 64 | 94 | 131 | 287 | 68 | 50 | 76 | 275 | 168 |
| *Roseateles* | 0 | 0 | 0 | 0 | 0 | 0 | 132 | 194 | 100 | 0 | 93 | 0 | 0 |
| *Candidatus Saccharimonas* | 0 | 0 | 0 | 0 | 0 | 140 | 133 | 169 | 0 | 0 | 0 | 0 | 0 |
| *Ramlibacter* | 0 | 0 | 0 | 0 | 0 | 0 | 134 | 0 | 821 | 832 | 61 | 272 | 426 |
| *Caldimonas* | 0 | 0 | 0 | 0 | 0 | 0 | 134 | 157 | 0 | 0 | 0 | 0 | 0 |
| *Flavisolibacter* | 0 | 0 | 0 | 0 | 0 | 0 | 136 | 114 | 246 | 162 | 88 | 176 | 161 |
| *Niabella* | 0 | 0 | 0 | 0 | 0 | 0 | 136 | 175 | 154 | 61 | 0 | 0 | 177 |
| *Nocardia* | 0 | 0 | 0 | 0 | 0 | 0 | 138 | 236 | 0 | 0 | 0 | 0 | 61 |
| *Leptonema* | 0 | 0 | 0 | 0 | 0 | 0 | 140 | 36 | 0 | 0 | 0 | 0 | 0 |
| *Steroidobacter* | 0 | 0 | 0 | 0 | 0 | 0 | 140 | 99 | 122 | 0 | 0 | 188 | 257 |
| *Azospira* | 517 | 369 | 103 | 78 | 130 | 157 | 140 | 127 | 0 | 0 | 99 | 0 | 0 |
| *Planctomyces* | 0 | 0 | 0 | 0 | 0 | 0 | 141 | 103 | 74 | 0 | 0 | 0 | 390 |
| *Hymenobacter* | 0 | 0 | 0 | 0 | 0 | 0 | 141 | 111 | 75 | 98 | 55 | 161 | 157 |
| *Micromonospora* | 0 | 0 | 0 | 0 | 0 | 0 | 145 | 122 | 0 | 0 | 0 | 98 | 0 |
| *Herminiimonas* | 0 | 75 | 0 | 0 | 0 | 0 | 145 | 319 | 166 | 168 | 0 | 0 | 0 |
| *Thioalkalivibrio* | 0 | 0 | 0 | 0 | 0 | 0 | 147 | 166 | 0 | 66 | 0 | 109 | 130 |
| *Gemmatimonas* | 0 | 0 | 0 | 0 | 0 | 0 | 148 | 133 | 64 | 165 | 0 | 58 | 272 |
| *Pseudorhodoferax* | 0 | 0 | 0 | 0 | 0 | 0 | 151 | 193 | 240 | 180 | 64 | 0 | 147 |
| *Sulfuritalea* | 0 | 0 | 0 | 0 | 0 | 0 | 156 | 203 | 0 | 0 | 0 | 73 | 0 |
| *Paraburkholderia* | 0 | 48 | 46 | 0 | 52 | 0 | 156 | 493 | 122 | 142 | 64 | 222 | 138 |
| *Lewinella* | 0 | 0 | 0 | 0 | 0 | 0 | 157 | 135 | 28 | 26 | 39 | 275 | 72 |
| *Sandaracinus* | 0 | 0 | 0 | 0 | 0 | 0 | 157 | 316 | 0 | 0 | 0 | 259 | 77 |
| *Oligella* | 0 | 0 | 0 | 560 | 70 | 0 | 158 | 119 | 0 | 0 | 0 | 0 | 0 |
| *Herbaspirillum* | 85 | 85 | 91 | 0 | 123 | 0 | 158 | 240 | 1069 | 908 | 214 | 358 | 771 |
| *Streptococcus* | 2161 | 1622 | 1612 | 2257 | 1089 | 2520 | 159 | 134 | 0 | 0 | 34 | 0 | 0 |
| *Schlesneria* | 0 | 0 | 0 | 0 | 0 | 0 | 160 | 92 | 0 | 0 | 0 | 0 | 948 |
| *Ruminococcus* | 1229 | 2391 | 2483 | 4386 | 2501 | 2361 | 160 | 527 | 0 | 0 | 53 | 0 | 0 |
| *Marinobacter* | 0 | 0 | 0 | 0 | 0 | 0 | 161 | 191 | 68 | 63 | 67 | 126 | 170 |
| *Phycicoccus* | 0 | 0 | 0 | 0 | 0 | 0 | 162 | 0 | 0 | 0 | 0 | 0 | 0 |
| *Thiocapsa* | 0 | 0 | 0 | 0 | 0 | 0 | 162 | 0 | 0 | 0 | 0 | 0 | 0 |
| *Thiocystis* | 0 | 0 | 0 | 0 | 0 | 0 | 162 | 112 | 0 | 0 | 0 | 0 | 0 |
| *Flexibacter* | 0 | 0 | 0 | 0 | 0 | 0 | 162 | 192 | 120 | 106 | 83 | 311 | 152 |
| *Vibrio* | 162 | 162 | 43 | 32 | 145 | 135 | 162 | 251 | 97 | 92 | 199 | 116 | 164 |
| *Frankia* | 0 | 0 | 0 | 0 | 0 | 0 | 170 | 151 | 0 | 0 | 0 | 0 | 56 |
| *Uliginosibacterium* | 207 | 153 | 0 | 0 | 44 | 51 | 173 | 208 | 0 | 0 | 0 | 0 | 0 |
| *Chitinimonas* | 0 | 0 | 0 | 0 | 0 | 0 | 177 | 279 | 0 | 0 | 77 | 0 | 0 |
| *Sphaerotilus* | 2791 | 993 | 507 | 331 | 1173 | 372 | 180 | 0 | 296 | 433 | 4852 | 207 | 0 |
| *Thiobacillus* | 39 | 0 | 0 | 0 | 0 | 0 | 180 | 157 | 95 | 73 | 66 | 0 | 0 |
| *Haliscomenobacter* | 0 | 0 | 0 | 0 | 0 | 0 | 181 | 100 | 96 | 63 | 78 | 1426 | 375 |
| *Enterobacter* | 8291 | 4673 | 2508 | 89 | 3062 | 670 | 181 | 193 | 0 | 0 | 216 | 0 | 0 |
| *Anaerolinea* | 0 | 0 | 0 | 0 | 0 | 0 | 182 | 129 | 0 | 0 | 0 | 0 | 0 |
| *Rhodoferax* | 0 | 0 | 0 | 0 | 61 | 0 | 185 | 279 | 197 | 165 | 109 | 0 | 0 |
| *Prevotella* | 4051 | 10525 | 2716 | 9279 | 5445 | 6798 | 193 | 591 | 54 | 0 | 706 | 271 | 234 |
| *Gemmata* | 0 | 0 | 0 | 0 | 0 | 0 | 197 | 534 | 44 | 0 | 0 | 45 | 137 |
| *Parachlamydia* | 0 | 0 | 0 | 0 | 0 | 0 | 198 | 126 | 0 | 0 | 0 | 0 | 0 |
| *Leptothrix* | 0 | 0 | 0 | 0 | 0 | 0 | 199 | 129 | 284 | 308 | 221 | 0 | 169 |
| *Aquabacterium* | 0 | 0 | 0 | 0 | 36 | 0 | 199 | 304 | 314 | 361 | 1902 | 0 | 0 |
| *Desulfovibrio* | 2563 | 2413 | 456 | 272 | 166 | 744 | 201 | 415 | 35 | 0 | 52 | 66 | 51 |
| *Bordetella* | 94 | 75 | 68 | 0 | 75 | 0 | 204 | 313 | 458 | 301 | 104 | 930 | 622 |
| *Flavihumibacter* | 0 | 0 | 0 | 0 | 0 | 0 | 205 | 90 | 410 | 219 | 158 | 421 | 347 |
| *Anaeromyxobacter* | 0 | 0 | 0 | 0 | 0 | 0 | 206 | 302 | 72 | 0 | 97 | 196 | 64 |
| *Stenotrophomonas* | 168 | 158 | 82 | 126 | 101 | 148 | 215 | 142 | 68 | 95 | 99 | 245 | 376 |
| *Arthrobacter* | 0 | 0 | 0 | 0 | 0 | 0 | 219 | 223 | 0 | 0 | 0 | 0 | 0 |
| *Thiothrix* | 0 | 0 | 0 | 0 | 0 | 110 | 220 | 189 | 0 | 0 | 50 | 0 | 0 |
| *Methylosarcina* | 0 | 0 | 0 | 0 | 0 | 0 | 223 | 144 | 68 | 278 | 0 | 0 | 0 |
| *Arcobacter* | 3674 | 3012 | 1245 | 1423 | 1626 | 6046 | 223 | 1190 | 68 | 0 | 1554 | 0 | 0 |
| *Algoriphagus* | 0 | 0 | 0 | 0 | 0 | 0 | 225 | 0 | 7474 | 2235 | 1020 | 382 | 419 |
| *Candidatus Microthrix* | 0 | 0 | 0 | 0 | 0 | 0 | 226 | 44 | 0 | 0 | 0 | 0 | 0 |
| *Pedosphaera* | 0 | 0 | 0 | 0 | 0 | 0 | 228 | 246 | 385 | 106 | 38 | 221 | 1444 |
| *Myxococcus* | 0 | 0 | 0 | 0 | 0 | 0 | 228 | 346 | 0 | 0 | 0 | 109 | 104 |
| *Ilumatobacter* | 0 | 0 | 0 | 0 | 0 | 0 | 230 | 60 | 0 | 0 | 0 | 45 | 320 |
| *Ardenticatena* | 0 | 0 | 0 | 0 | 0 | 0 | 232 | 124 | 0 | 0 | 0 | 0 | 33 |
| *Methylobacterium* | 55 | 0 | 65 | 43 | 104 | 0 | 235 | 539 | 45 | 79 | 63 | 241 | 150 |
| *Haliangium* | 0 | 0 | 0 | 0 | 0 | 0 | 237 | 538 | 0 | 0 | 0 | 0 | 72 |
| *Achromobacter* | 108 | 100 | 99 | 41 | 175 | 0 | 243 | 387 | 202 | 186 | 123 | 404 | 217 |
| *Dongia* | 0 | 0 | 0 | 0 | 0 | 0 | 244 | 78 | 68 | 0 | 0 | 0 | 0 |
| *Runella* | 0 | 0 | 0 | 0 | 0 | 0 | 248 | 111 | 373 | 262 | 284 | 211 | 249 |
| *Patulibacter* | 0 | 0 | 0 | 0 | 0 | 69 | 254 | 0 | 0 | 0 | 0 | 0 | 0 |
| *Rhizobium* | 624 | 451 | 340 | 0 | 278 | 0 | 254 | 647 | 263 | 301 | 279 | 487 | 316 |
| *Massilia* | 76 | 0 | 80 | 0 | 198 | 0 | 259 | 264 | 820 | 823 | 587 | 444 | 798 |
| *Paenibacillus* | 86 | 57 | 56 | 64 | 0 | 85 | 259 | 468 | 85 | 100 | 0 | 155 | 180 |
| *Paludibacter* | 345 | 43 | 0 | 31 | 0 | 1277 | 271 | 244 | 242 | 156 | 87 | 0 | 0 |
| *Klebsiella* | 8638 | 4412 | 3455 | 2709 | 3521 | 2578 | 271 | 326 | 0 | 206 | 282 | 0 | 0 |
| *Pelomonas* | 0 | 0 | 245 | 126 | 104 | 0 | 277 | 243 | 987 | 1380 | 3482 | 483 | 182 |
| *Bacteroides* | 14541 | 16458 | 20084 | 23139 | 20169 | 21896 | 278 | 1096 | 121 | 106 | 2968 | 206 | 129 |
| *Pedobacter* | 28 | 25 | 59 | 46 | 25 | 84 | 283 | 175 | 631 | 298 | 180 | 459 | 325 |
| *Microbacterium* | 405 | 119 | 1481 | 189 | 0 | 205 | 296 | 424 | 0 | 0 | 0 | 105 | 0 |
| *Ralstonia* | 117 | 90 | 68 | 66 | 98 | 0 | 299 | 513 | 378 | 309 | 169 | 185 | 222 |
| *Labilithrix* | 0 | 0 | 0 | 0 | 0 | 0 | 302 | 643 | 0 | 0 | 0 | 64 | 79 |
| *Curvibacter* | 117 | 85 | 98 | 107 | 115 | 0 | 303 | 286 | 4328 | 1752 | 491 | 1053 | 748 |
| *Sphingobium* | 0 | 75 | 58 | 40 | 0 | 0 | 315 | 349 | 265 | 426 | 197 | 57 | 218 |
| *Pirellula* | 0 | 0 | 0 | 0 | 0 | 0 | 321 | 351 | 107 | 0 | 0 | 131 | 1189 |
| *Chitinophaga* | 0 | 0 | 0 | 0 | 0 | 0 | 332 | 296 | 332 | 148 | 133 | 529 | 271 |
| *Legionella* | 0 | 0 | 0 | 0 | 0 | 0 | 332 | 297 | 61 | 59 | 0 | 146 | 143 |
| *Alicycliphilus* | 1120 | 596 | 740 | 676 | 634 | 288 | 336 | 236 | 229 | 175 | 133 | 0 | 0 |
| *Propionivibrio* | 251 | 626 | 86 | 53 | 101 | 400 | 341 | 388 | 48 | 0 | 63 | 0 | 0 |
| *Clostridium* | 1203 | 2230 | 2669 | 6891 | 2161 | 2234 | 341 | 603 | 210 | 225 | 350 | 176 | 127 |
| *Methyloversatilis* | 41 | 36 | 487 | 181 | 0 | 41 | 346 | 421 | 402 | 711 | 190 | 151 | 117 |
| *Lautropia* | 0 | 0 | 0 | 0 | 0 | 0 | 347 | 233 | 453 | 241 | 81 | 4134 | 1958 |
| *Verrucomicrobium* | 0 | 0 | 0 | 0 | 0 | 0 | 352 | 413 | 1206 | 469 | 43 | 1844 | 375 |
| *Hydrogenophaga* | 98 | 100 | 126 | 97 | 122 | 0 | 362 | 307 | 4264 | 2915 | 2158 | 458 | 800 |
| *Bacillus* | 103 | 85 | 69 | 116 | 33 | 133 | 364 | 500 | 129 | 103 | 85 | 196 | 204 |
| *Aureispira* | 0 | 0 | 0 | 0 | 0 | 0 | 365 | 230 | 0 | 0 | 141 | 706 | 432 |
| *Azoarcus* | 82 | 68 | 63 | 36 | 64 | 80 | 366 | 492 | 64 | 46 | 112 | 78 | 0 |
| *Novosphingobium* | 113 | 0 | 310 | 214 | 500 | 0 | 378 | 153 | 5475 | 5382 | 2823 | 245 | 1127 |
| *Kouleothrix* | 0 | 0 | 0 | 0 | 0 | 0 | 379 | 260 | 0 | 0 | 0 | 31 | 34 |
| *Methylibium* | 0 | 0 | 0 | 0 | 0 | 0 | 379 | 462 | 569 | 735 | 280 | 399 | 546 |
| *Sphingomonas* | 113 | 91 | 152 | 94 | 62 | 0 | 385 | 435 | 580 | 986 | 434 | 5663 | 1062 |
| *Delftia* | 437 | 375 | 253 | 379 | 522 | 119 | 387 | 393 | 112 | 134 | 122 | 0 | 0 |
| *Candidatus Competibacter* | 0 | 0 | 0 | 0 | 0 | 61 | 392 | 1157 | 0 | 0 | 0 | 0 | 0 |
| *Solirubrobacter* | 0 | 0 | 0 | 0 | 0 | 61 | 394 | 93 | 0 | 0 | 0 | 0 | 0 |
| *Chondromyces* | 0 | 0 | 0 | 0 | 0 | 0 | 401 | 417 | 0 | 0 | 0 | 63 | 0 |
| *Caldilinea* | 0 | 0 | 0 | 0 | 0 | 0 | 405 | 195 | 0 | 0 | 0 | 36 | 80 |
| *Cupriavidus* | 241 | 128 | 140 | 120 | 120 | 90 | 429 | 467 | 349 | 426 | 128 | 231 | 253 |
| *Janthinobacterium* | 72 | 70 | 0 | 0 | 110 | 0 | 431 | 656 | 309 | 559 | 225 | 502 | 718 |
| *Ideonella* | 0 | 0 | 0 | 0 | 0 | 0 | 455 | 360 | 492 | 593 | 330 | 236 | 242 |
| *Geothrix* | 0 | 35 | 0 | 0 | 0 | 0 | 458 | 58 | 0 | 0 | 0 | 0 | 0 |
| *Mesorhizobium* | 64 | 0 | 104 | 0 | 64 | 0 | 476 | 776 | 164 | 161 | 75 | 343 | 242 |
| *Polaromonas* | 152 | 110 | 90 | 87 | 140 | 0 | 505 | 579 | 832 | 573 | 214 | 557 | 522 |
| *Perlucidibaca* | 0 | 0 | 0 | 0 | 0 | 0 | 509 | 752 | 35 | 87 | 482 | 0 | 0 |
| *Hyphomicrobium* | 0 | 0 | 65 | 0 | 0 | 0 | 587 | 239 | 148 | 75 | 0 | 710 | 202 |
| *Conexibacter* | 0 | 0 | 0 | 0 | 0 | 106 | 602 | 88 | 0 | 0 | 0 | 0 | 0 |
| *Chryseobacterium* | 1109 | 1201 | 1044 | 1375 | 1154 | 1222 | 642 | 424 | 324 | 530 | 1410 | 563 | 355 |
| *Rubrivivax* | 0 | 0 | 0 | 0 | 0 | 0 | 645 | 628 | 231 | 403 | 147 | 207 | 315 |
| *Turneriella* | 0 | 0 | 0 | 0 | 0 | 0 | 648 | 219 | 0 | 0 | 0 | 0 | 0 |
| *Dechloromonas* | 1107 | 879 | 793 | 607 | 254 | 1705 | 687 | 1841 | 176 | 574 | 1618 | 0 | 69 |
| *Niastella* | 0 | 0 | 0 | 0 | 0 | 0 | 698 | 290 | 460 | 221 | 126 | 636 | 271 |
| *Marmoricola* | 0 | 0 | 0 | 0 | 0 | 0 | 717 | 0 | 0 | 0 | 0 | 0 | 0 |
| *Cloacibacterium* | 3440 | 2876 | 2354 | 2523 | 2393 | 2947 | 744 | 2194 | 146 | 1749 | 3931 | 0 | 0 |
| *Hassallia* | 0 | 0 | 0 | 0 | 0 | 0 | 752 | 90 | 122 | 0 | 0 | 0 | 101 |
| *Ottowia* | 383 | 556 | 683 | 593 | 263 | 182 | 766 | 236 | 368 | 330 | 64 | 0 | 139 |
| *Variovorax* | 250 | 185 | 188 | 175 | 201 | 119 | 774 | 830 | 1248 | 754 | 347 | 1016 | 783 |
| *Streptomyces* | 26 | 0 | 35 | 0 | 0 | 0 | 782 | 1211 | 155 | 81 | 40 | 319 | 292 |
| *Bdellovibrio* | 124 | 88 | 137 | 111 | 91 | 63 | 816 | 1059 | 375 | 652 | 632 | 130 | 220 |
| *Sorangium* | 0 | 0 | 0 | 0 | 0 | 0 | 835 | 917 | 0 | 0 | 0 | 149 | 134 |
| *Candidatus Contendobacter* | 0 | 0 | 0 | 0 | 0 | 140 | 886 | 2395 | 0 | 0 | 0 | 0 | 0 |
| *Tetrasphaera* | 0 | 0 | 0 | 0 | 0 | 0 | 926 | 0 | 0 | 0 | 0 | 0 | 0 |
| *Paracoccus* | 759 | 744 | 2424 | 739 | 107 | 528 | 963 | 243 | 227 | 162 | 444 | 0 | 0 |
| *Pseudomonas* | 3613 | 1475 | 3110 | 1906 | 4516 | 1083 | 1165 | 1624 | 2560 | 952 | 13107 | 437 | 549 |
| *Flavobacterium* | 946 | 1213 | 1397 | 1298 | 651 | 554 | 1177 | 529 | 4005 | 12119 | 9794 | 1871 | 1551 |
| *Nannocystis* | 0 | 0 | 0 | 0 | 0 | 0 | 1230 | 637 | 0 | 0 | 0 | 56 | 42 |
| *Comamonas* | 7755 | 5252 | 4766 | 1702 | 8881 | 1004 | 1406 | 694 | 875 | 1064 | 5466 | 471 | 687 |
| *Burkholderia* | 629 | 573 | 364 | 438 | 721 | 675 | 1478 | 2112 | 1059 | 1273 | 583 | 1279 | 1036 |
| *Acinetobacter* | 4969 | 4555 | 5337 | 3611 | 8237 | 4132 | 1519 | 1656 | 497 | 818 | 10795 | 122 | 98 |
| *Nocardioides* | 0 | 0 | 0 | 0 | 0 | 80 | 1521 | 343 | 0 | 0 | 0 | 94 | 0 |
| *Bradyrhizobium* | 103 | 87 | 137 | 66 | 70 | 163 | 1531 | 1812 | 475 | 291 | 218 | 897 | 621 |
| *Acidovorax* | 4914 | 4325 | 5089 | 5892 | 7067 | 5401 | 1599 | 1295 | 1926 | 1648 | 2632 | 706 | 678 |
| *Rickettsia* | 0 | 0 | 0 | 0 | 0 | 0 | 1829 | 926 | 82 | 134 | 0 | 1772 | 483 |
| *Leptospira* | 0 | 0 | 0 | 0 | 0 | 0 | 2001 | 362 | 38 | 60 | 51 | 534 | 75 |
| *Nitrosomonas* | 60 | 58 | 32 | 30 | 56 | 76 | 2502 | 760 | 115 | 158 | 87 | 75 | 133 |
| *Candidatus Accumulibacter* | 72 | 95 | 56 | 47 | 61 | 361 | 4649 | 8374 | 91 | 125 | 113 | 93 | 81 |
| *Nitrospira* | 0 | 0 | 32 | 0 | 0 | 75 | 5071 | 1433 | 61 | 0 | 112 | 0 | 32 |
| *Thauera* | 271 | 182 | 175 | 112 | 704 | 813 | 8052 | 5385 | 322 | 481 | 1352 | 107 | 96 |
| *Mycobacterium* | 160 | 42 | 594 | 104 | 65 | 1129 | 10215 | 9450 | 80 | 84 | 76 | 189 | 184 |

Table S8. ARGs identified.

| Sample ID | IMG gene ID | Antibiotic resistant gene/protein |
| --- | --- | --- |
| BB | Ga0110930_12140582 | AAC(3)-Id |
| BB | Ga0110930_10719292 | AAC(6')-Ib9 |
| BB | Ga0110930_11513951 | aadA11 |
| BB | Ga0110930_10896191 | abeM |
| BB | Ga0110930_10985231 | abeM |
| BB | Ga0110930_12320491 | acrD |
| BB | Ga0110930_10461672 | adeB |
| BB | Ga0110930_10567191 | adeB |
| BB | Ga0110930_10836761 | adeB |
| BB | Ga0110930_10923431 | adeB |
| BB | Ga0110930_11164441 | adeB |
| BB | Ga0110930_11925831 | adeB |
| BB | Ga0110930_10302871 | adeC-adeK-oprM |
| BB | Ga0110930_10461671 | adeC-adeK-oprM |
| BB | Ga0110930_10768752 | adeC-adeK-oprM |
| BB | Ga0110930_11600051 | adeC-adeK-oprM |
| BB | Ga0110930_10097301 | adeG |
| BB | Ga0110930_11587661 | adeG |
| BB | Ga0110930_11827651 | adeJ |
| BB | Ga0110930_10124282 | ANT3 |
| BB | Ga0110930_10164381 | ANT3 |
| BB | Ga0110930_10787071 | ANT3 |
| BB | Ga0110930_10008401 | APH3" |
| BB | Ga0110930_10037351 | APH3" |
| BB | Ga0110930_10051724 | APH3" |
| BB | Ga0110930_10150301 | APH3" |
| BB | Ga0110930_10336051 | APH3" |
| BB | Ga0110930_10351441 | APH3" |
| BB | Ga0110930_10384821 | APH3" |
| BB | Ga0110930_10539391 | APH3" |
| BB | Ga0110930_10541543 | APH3" |
| BB | Ga0110930_10785791 | APH3" |
| BB | Ga0110930_10823291 | APH3" |
| BB | Ga0110930_12339081 | APH3" |
| BB | Ga0110930_12380211 | APH3" |
| BB | Ga0110930_10872471 | APH3' |
| BB | Ga0110930_11532242 | APH6 |
| BB | Ga0110930_10196111 | arnA |
| BB | Ga0110930_10089401 | baeR |
| BB | Ga0110930_11187311 | baeR |
| BB | Ga0110930_11539422 | baeR |
| BB | Ga0110930_10089402 | baeS |
| BB | Ga0110930_10698621 | ceoB |
| BB | Ga0110930_10310793 | Cfr 23 Ribosomal RNA Methyltransferase |
| BB | Ga0110930_11369031 | Cfr 23 Ribosomal RNA Methyltransferase |
| BB | Ga0110930_11808341 | Cfr 23 Ribosomal RNA Methyltransferase |
| BB | Ga0110930_11808351 | Cfr 23 Ribosomal RNA Methyltransferase |
| BB | Ga0110930_11966192 | cfxA6 |
| BB | Ga0110930_11442212 | Chloramphenicol Acetyltransferase CAT |
| BB | Ga0110930_11499172 | CRP |
| BB | Ga0110930_11005371 | dfrA1 |
| BB | Ga0110930_12429971 | dfrA16 |
| BB | Ga0110930_11910141 | dfrA17 |
| BB | Ga0110930_11049831 | emrB |
| BB | Ga0110930_11096821 | emrB |
| BB | Ga0110930_10107051 | ermF |
| BB | Ga0110930_10810791 | ermF |
| BB | Ga0110930_11586891 | evgS |
| BB | Ga0110930_10015841 | Fluoroquinolone Resistant DNA Topoisomerase |
| BB | Ga0110930_10015851 | Fluoroquinolone Resistant DNA Topoisomerase |
| BB | Ga0110930_10015861 | Fluoroquinolone Resistant DNA Topoisomerase |
| BB | Ga0110930_10015881 | Fluoroquinolone Resistant DNA Topoisomerase |
| BB | Ga0110930_10015882 | Fluoroquinolone Resistant DNA Topoisomerase |
| BB | Ga0110930_10052962 | Fluoroquinolone Resistant DNA Topoisomerase |
| BB | Ga0110930_10078021 | Fluoroquinolone Resistant DNA Topoisomerase |
| BB | Ga0110930_10087551 | Fluoroquinolone Resistant DNA Topoisomerase |
| BB | Ga0110930_10096452 | Fluoroquinolone Resistant DNA Topoisomerase |
| BB | Ga0110930_10101841 | Fluoroquinolone Resistant DNA Topoisomerase |
| BB | Ga0110930_10102991 | Fluoroquinolone Resistant DNA Topoisomerase |
| BB | Ga0110930_10103005 | Fluoroquinolone Resistant DNA Topoisomerase |
| BB | Ga0110930_10128851 | Fluoroquinolone Resistant DNA Topoisomerase |
| BB | Ga0110930_10182181 | Fluoroquinolone Resistant DNA Topoisomerase |
| BB | Ga0110930_10182251 | Fluoroquinolone Resistant DNA Topoisomerase |
| BB | Ga0110930_10237031 | Fluoroquinolone Resistant DNA Topoisomerase |
| BB | Ga0110930_10237041 | Fluoroquinolone Resistant DNA Topoisomerase |
| BB | Ga0110930_10339161 | Fluoroquinolone Resistant DNA Topoisomerase |
| BB | Ga0110930_10364271 | Fluoroquinolone Resistant DNA Topoisomerase |
| BB | Ga0110930_10382751 | Fluoroquinolone Resistant DNA Topoisomerase |
| BB | Ga0110930_10421251 | Fluoroquinolone Resistant DNA Topoisomerase |
| BB | Ga0110930_10439161 | Fluoroquinolone Resistant DNA Topoisomerase |
| BB | Ga0110930_10439652 | Fluoroquinolone Resistant DNA Topoisomerase |
| BB | Ga0110930_10453071 | Fluoroquinolone Resistant DNA Topoisomerase |
| BB | Ga0110930_10492601 | Fluoroquinolone Resistant DNA Topoisomerase |
| BB | Ga0110930_10502771 | Fluoroquinolone Resistant DNA Topoisomerase |
| BB | Ga0110930_10538651 | Fluoroquinolone Resistant DNA Topoisomerase |
| BB | Ga0110930_10596902 | Fluoroquinolone Resistant DNA Topoisomerase |
| BB | Ga0110930_10643881 | Fluoroquinolone Resistant DNA Topoisomerase |
| BB | Ga0110930_10643891 | Fluoroquinolone Resistant DNA Topoisomerase |
| BB | Ga0110930_10691501 | Fluoroquinolone Resistant DNA Topoisomerase |
| BB | Ga0110930_10708951 | Fluoroquinolone Resistant DNA Topoisomerase |
| BB | Ga0110930_10725911 | Fluoroquinolone Resistant DNA Topoisomerase |
| BB | Ga0110930_10878231 | Fluoroquinolone Resistant DNA Topoisomerase |
| BB | Ga0110930_10894821 | Fluoroquinolone Resistant DNA Topoisomerase |
| BB | Ga0110930_10922821 | Fluoroquinolone Resistant DNA Topoisomerase |
| BB | Ga0110930_11143091 | Fluoroquinolone Resistant DNA Topoisomerase |
| BB | Ga0110930_11173142 | Fluoroquinolone Resistant DNA Topoisomerase |
| BB | Ga0110930_11181862 | Fluoroquinolone Resistant DNA Topoisomerase |
| BB | Ga0110930_11267411 | Fluoroquinolone Resistant DNA Topoisomerase |
| BB | Ga0110930_11343151 | Fluoroquinolone Resistant DNA Topoisomerase |
| BB | Ga0110930_11385911 | Fluoroquinolone Resistant DNA Topoisomerase |
| BB | Ga0110930_11879291 | Fluoroquinolone Resistant DNA Topoisomerase |
| BB | Ga0110930_10379952 | macB |
| BB | Ga0110930_10442091 | macB |
| BB | Ga0110930_10442101 | macB |
| BB | Ga0110930_10442111 | macB |
| BB | Ga0110930_10542221 | macB |
| BB | Ga0110930_10721021 | macB |
| BB | Ga0110930_12020221 | mdfA |
| BB | Ga0110930_10006821 | mdtB |
| BB | Ga0110930_10254921 | mdtB |
| BB | Ga0110930_10670611 | mdtB |
| BB | Ga0110930_11095211 | mdtH |
| BB | Ga0110930_10032951 | mexB |
| BB | Ga0110930_10069831 | mexB |
| BB | Ga0110930_10244261 | mexB |
| BB | Ga0110930_10617401 | mexB |
| BB | Ga0110930_10792251 | mexB |
| BB | Ga0110930_10498571 | mexC |
| BB | Ga0110930_10024431 | mexE |
| BB | Ga0110930_10209151 | mexE |
| BB | Ga0110930_11275271 | mexE |
| BB | Ga0110930_10043861 | mexF |
| BB | Ga0110930_10246761 | mexF |
| BB | Ga0110930_10249391 | mexF |
| BB | Ga0110930_10619511 | mexF |
| BB | Ga0110930_12410101 | mexF |
| BB | Ga0110930_10000366 | mexK |
| BB | Ga0110930_10065261 | mexK |
| BB | Ga0110930_10129931 | mexK |
| BB | Ga0110930_10345761 | mexK |
| BB | Ga0110930_10571291 | mexK |
| BB | Ga0110930_10641712 | mexK |
| BB | Ga0110930_10974291 | mexK |
| BB | Ga0110930_10604691 | mexT |
| BB | Ga0110930_10799471 | mexT |
| BB | Ga0110930_10850152 | mexT |
| BB | Ga0110930_10906361 | mexT |
| BB | Ga0110930_11193821 | mexT |
| BB | Ga0110930_11433931 | mexT |
| BB | Ga0110930_12187491 | mexT |
| BB | Ga0110930_12117741 | *bla*_MOX-8_ |
| BB | Ga0110930_10085171 | msbA |
| BB | Ga0110930_10085181 | msbA |
| BB | Ga0110930_10238771 | msbA |
| BB | Ga0110930_10238781 | msbA |
| BB | Ga0110930_10238791 | msbA |
| BB | Ga0110930_10300311 | msbA |
| BB | Ga0110930_10300321 | msbA |
| BB | Ga0110930_10478461 | msbA |
| BB | Ga0110930_11069351 | msbA |
| BB | Ga0110930_11259771 | msbA |
| BB | Ga0110930_11558831 | msbA |
| BB | Ga0110930_12146901 | msbA |
| BB | Ga0110930_12146911 | msbA |
| BB | Ga0110930_10882911 | msrE |
| BB | Ga0110930_11049191 | msrE |
| BB | Ga0110930_10072001 | mtrD |
| BB | Ga0110930_10815891 | oprN |
| BB | Ga0110930_11165341 | oprN |
| BB | Ga0110930_10628711 | *bla*_OXA-209_ |
| BB | Ga0110930_10124281 | *bla*_OXA-35_ |
| BB | Ga0110930_11866211 | *bla*_PER-1_ |
| BB | Ga0110930_11883792 | phoP |
| BB | Ga0110930_10635011 | PmrE |
| BB | Ga0110930_11220261 | PmrE |
| BB | Ga0110930_11269071 | PmrE |
| BB | Ga0110930_11724581 | PmrE |
| BB | Ga0110930_10832961 | QnrS2 |
| BB | Ga0110930_11235031 | QnrS6 |
| BB | Ga0110930_10010701 | RND Antibiotic Efflux Pump |
| BB | Ga0110930_10010711 | RND Antibiotic Efflux Pump |
| BB | Ga0110930_10051191 | RND Antibiotic Efflux Pump |
| BB | Ga0110930_10069862 | RND Antibiotic Efflux Pump |
| BB | Ga0110930_10080281 | RND Antibiotic Efflux Pump |
| BB | Ga0110930_10080291 | RND Antibiotic Efflux Pump |
| BB | Ga0110930_10150431 | RND Antibiotic Efflux Pump |
| BB | Ga0110930_10150441 | RND Antibiotic Efflux Pump |
| BB | Ga0110930_10163681 | RND Antibiotic Efflux Pump |
| BB | Ga0110930_10235461 | RND Antibiotic Efflux Pump |
| BB | Ga0110930_10249191 | RND Antibiotic Efflux Pump |
| BB | Ga0110930_10254951 | RND Antibiotic Efflux Pump |
| BB | Ga0110930_10264771 | RND Antibiotic Efflux Pump |
| BB | Ga0110930_10295041 | RND Antibiotic Efflux Pump |
| BB | Ga0110930_10295042 | RND Antibiotic Efflux Pump |
| BB | Ga0110930_10362821 | RND Antibiotic Efflux Pump |
| BB | Ga0110930_10379141 | RND Antibiotic Efflux Pump |
| BB | Ga0110930_10392671 | RND Antibiotic Efflux Pump |
| BB | Ga0110930_10545172 | RND Antibiotic Efflux Pump |
| BB | Ga0110930_10545173 | RND Antibiotic Efflux Pump |
| BB | Ga0110930_10598141 | RND Antibiotic Efflux Pump |
| BB | Ga0110930_10639051 | RND Antibiotic Efflux Pump |
| BB | Ga0110930_10699781 | RND Antibiotic Efflux Pump |
| BB | Ga0110930_10710411 | RND Antibiotic Efflux Pump |
| BB | Ga0110930_10738561 | RND Antibiotic Efflux Pump |
| BB | Ga0110930_10835391 | RND Antibiotic Efflux Pump |
| BB | Ga0110930_11110471 | RND Antibiotic Efflux Pump |
| BB | Ga0110930_11343211 | RND Antibiotic Efflux Pump |
| BB | Ga0110930_11401251 | RND Antibiotic Efflux Pump |
| BB | Ga0110930_11535592 | RND Antibiotic Efflux Pump |
| BB | Ga0110930_11722751 | RND Antibiotic Efflux Pump |
| BB | Ga0110930_11835931 | RND Antibiotic Efflux Pump |
| BB | Ga0110930_11840641 | RND Antibiotic Efflux Pump |
| BB | Ga0110930_11910051 | RND Antibiotic Efflux Pump |
| BB | Ga0110930_12149911 | RND Antibiotic Efflux Pump |
| BB | Ga0110930_12183001 | RND Antibiotic Efflux Pump |
| BB | Ga0110930_12381381 | RND Antibiotic Efflux Pump |
| BB | Ga0110930_12413601 | RND Antibiotic Efflux Pump |
| BB | Ga0110930_12447581 | RND Antibiotic Efflux Pump |
| BB | Ga0110930_10033363 | sul1 |
| BB | Ga0110930_10396652 | sul2 |
| BB | Ga0110930_11083371 | sul2 |
| BB | Ga0110930_11083381 | sul2 |
| BB | Ga0110930_10179852 | Tetracycline Resistance MFS Efflux Pump |
| BB | Ga0110930_11332201 | Tetracycline Resistance MFS Efflux Pump |
| BB | Ga0110930_11851691 | Tetracycline Resistance MFS Efflux Pump |
| BB | Ga0110930_12078051 | Tetracycline Resistance MFS Efflux Pump |
| BB | Ga0110930_10295081 | Tetracycline Resistance Ribosomal Protection Protein |
| BB | Ga0110930_10833831 | Tetracycline Resistance Ribosomal Protection Protein |
| BB | Ga0110930_11345041 | Tetracycline Resistance Ribosomal Protection Protein |
| BB | Ga0110930_11544472 | Tetracycline Resistance Ribosomal Protection Protein |
| BB | Ga0110930_11778061 | tetX |
| BH | Ga0110927_10693393 | aadA |
| BH | Ga0110927_10088001 | acrB |
| BH | Ga0110927_10130261 | APH3" |
| BH | Ga0110927_10193691 | APH3" |
| BH | Ga0110927_10270491 | APH3" |
| BH | Ga0110927_10551351 | APH3" |
| BH | Ga0110927_10737362 | APH3" |
| BH | Ga0110927_10737372 | APH3" |
| BH | Ga0110927_11492802 | APH3" |
| BH | Ga0110927_12424761 | APH3" |
| BH | Ga0110927_11172351 | baeR |
| BH | Ga0110927_11507691 | ereA |
| BH | Ga0110927_10003211 | Fluoroquinolone Resistant DNA Topoisomerase |
| BH | Ga0110927_10071841 | Fluoroquinolone Resistant DNA Topoisomerase |
| BH | Ga0110927_10168971 | Fluoroquinolone Resistant DNA Topoisomerase |
| BH | Ga0110927_10375951 | Fluoroquinolone Resistant DNA Topoisomerase |
| BH | Ga0110927_10383402 | Fluoroquinolone Resistant DNA Topoisomerase |
| BH | Ga0110927_10543341 | Fluoroquinolone Resistant DNA Topoisomerase |
| BH | Ga0110927_10587811 | Fluoroquinolone Resistant DNA Topoisomerase |
| BH | Ga0110927_10627611 | Fluoroquinolone Resistant DNA Topoisomerase |
| BH | Ga0110927_10699922 | Fluoroquinolone Resistant DNA Topoisomerase |
| BH | Ga0110927_10700121 | Fluoroquinolone Resistant DNA Topoisomerase |
| BH | Ga0110927_10487431 | macB |
| BH | Ga0110927_10833511 | mdtB |
| BH | Ga0110927_11140901 | mdtB |
| BH | Ga0110927_11281092 | mdtB |
| BH | Ga0110927_11961371 | mdtB |
| BH | Ga0110927_10402431 | mdtF |
| BH | Ga0110927_10837511 | mexF |
| BH | Ga0110927_10945431 | mexF |
| BH | Ga0110927_10025013 | mexK |
| BH | Ga0110927_10110451 | mexK |
| BH | Ga0110927_10110461 | mexK |
| BH | Ga0110927_10447301 | mexK |
| BH | Ga0110927_11024411 | mexK |
| BH | Ga0110927_11921872 | mexK |
| BH | Ga0110927_10351981 | oprN |
| BH | Ga0110927_11350081 | *bla*_OXA_ |
| BH | Ga0110927_11883891 | *bla*_OXA-198_ |
| BH | Ga0110927_11517201 | *bla*_OXA-209_ |
| BH | Ga0110927_11050431 | PmrE |
| BH | Ga0110927_10009011 | RND Antibiotic Efflux Pump |
| BH | Ga0110927_10080571 | RND Antibiotic Efflux Pump |
| BH | Ga0110927_10083791 | RND Antibiotic Efflux Pump |
| BH | Ga0110927_10083831 | RND Antibiotic Efflux Pump |
| BH | Ga0110927_10228351 | RND Antibiotic Efflux Pump |
| BH | Ga0110927_10244731 | RND Antibiotic Efflux Pump |
| BH | Ga0110927_10260951 | RND Antibiotic Efflux Pump |
| BH | Ga0110927_10286681 | RND Antibiotic Efflux Pump |
| BH | Ga0110927_10299211 | RND Antibiotic Efflux Pump |
| BH | Ga0110927_10342771 | RND Antibiotic Efflux Pump |
| BH | Ga0110927_10403171 | RND Antibiotic Efflux Pump |
| BH | Ga0110927_10543781 | RND Antibiotic Efflux Pump |
| BH | Ga0110927_10551941 | RND Antibiotic Efflux Pump |
| BH | Ga0110927_10552121 | RND Antibiotic Efflux Pump |
| BH | Ga0110927_10644861 | RND Antibiotic Efflux Pump |
| BH | Ga0110927_10688161 | RND Antibiotic Efflux Pump |
| BH | Ga0110927_10728311 | RND Antibiotic Efflux Pump |
| BH | Ga0110927_10744621 | RND Antibiotic Efflux Pump |
| BH | Ga0110927_10837171 | RND Antibiotic Efflux Pump |
| BH | Ga0110927_10843731 | RND Antibiotic Efflux Pump |
| BH | Ga0110927_10870011 | RND Antibiotic Efflux Pump |
| BH | Ga0110927_10912311 | RND Antibiotic Efflux Pump |
| BH | Ga0110927_10956681 | RND Antibiotic Efflux Pump |
| BH | Ga0110927_10968401 | RND Antibiotic Efflux Pump |
| BH | Ga0110927_10968402 | RND Antibiotic Efflux Pump |
| BH | Ga0110927_10992421 | RND Antibiotic Efflux Pump |
| BH | Ga0110927_11227781 | RND Antibiotic Efflux Pump |
| BH | Ga0110927_11263281 | RND Antibiotic Efflux Pump |
| BH | Ga0110927_11356311 | RND Antibiotic Efflux Pump |
| BH | Ga0110927_11847651 | RND Antibiotic Efflux Pump |
| BH | Ga0110927_11127351 | Beta-lactamase Class D |
| BH | Ga0110927_10693391 | sul1 |
| BH | Ga0110927_11757771 | tetX |
| BI | Ga0110932_11828151 | aadA11 |
| BI | Ga0110932_11111231 | ANT3 |
| BI | Ga0110932_11189861 | ANT3 |
| BI | Ga0110932_11494342 | APH3" |
| BI | Ga0110932_10346411 | APH3" |
| BI | Ga0110932_10528081 | APH3" |
| BI | Ga0110932_10030301 | APH3" |
| BI | Ga0110932_10026232 | APH3" |
| BI | Ga0110932_10874121 | APH3" |
| BI | Ga0110932_11363381 | APH3" |
| BI | Ga0110932_10252921 | APH3" |
| BI | Ga0110932_11346132 | APH3" |
| BI | Ga0110932_10126671 | APH3" |
| BI | Ga0110932_10586541 | APH3" |
| BI | Ga0110932_10570832 | APH3" |
| BI | Ga0110932_10163901 | APH3" |
| BI | Ga0110932_10173861 | APH6 |
| BI | Ga0110932_11084292 | bacA |
| BI | Ga0110932_10403451 | dfrE |
| BI | Ga0110932_10358491 | Fluoroquinolone Resistant DNA Topoisomerase |
| BI | Ga0110932_11019671 | Fluoroquinolone Resistant DNA Topoisomerase |
| BI | Ga0110932_11355651 | Fluoroquinolone Resistant DNA Topoisomerase |
| BI | Ga0110932_12036281 | Fluoroquinolone Resistant DNA Topoisomerase |
| BI | Ga0110932_10138231 | Fluoroquinolone Resistant DNA Topoisomerase |
| BI | Ga0110932_10050572 | Fluoroquinolone Resistant DNA Topoisomerase |
| BI | Ga0110932_10800751 | Fluoroquinolone Resistant DNA Topoisomerase |
| BI | Ga0110932_10557871 | Fluoroquinolone Resistant DNA Topoisomerase |
| BI | Ga0110932_10557881 | Fluoroquinolone Resistant DNA Topoisomerase |
| BI | Ga0110932_11366251 | Fluoroquinolone Resistant DNA Topoisomerase |
| BI | Ga0110932_10144771 | Fluoroquinolone Resistant DNA Topoisomerase |
| BI | Ga0110932_10144841 | Fluoroquinolone Resistant DNA Topoisomerase |
| BI | Ga0110932_11968521 | Fluoroquinolone Resistant DNA Topoisomerase |
| BI | Ga0110932_11521732 | Fluoroquinolone Resistant DNA Topoisomerase |
| BI | Ga0110932_11087461 | Fluoroquinolone Resistant DNA Topoisomerase |
| BI | Ga0110932_12020472 | fosC2 |
| BI | Ga0110932_10491001 | mdtB |
| BI | Ga0110932_10094102 | mexF |
| BI | Ga0110932_10233261 | mexK |
| BI | Ga0110932_10063271 | mexK |
| BI | Ga0110932_10118561 | mexK |
| BI | Ga0110932_11371971 | mexK |
| BI | Ga0110932_10118631 | mexK |
| BI | Ga0110932_10112051 | mexY |
| BI | Ga0110932_11207961 | *bla*_OXA-198_ |
| BI | Ga0110932_10806811 | PmrE |
| BI | Ga0110932_11687101 | PmrE |
| BI | Ga0110932_10587202 | RND Antibiotic Efflux Pump |
| BI | Ga0110932_10943651 | RND Antibiotic Efflux Pump |
| BI | Ga0110932_11284101 | RND Antibiotic Efflux Pump |
| BI | Ga0110932_10009081 | RND Antibiotic Efflux Pump |
| BI | Ga0110932_11526891 | RND Antibiotic Efflux Pump |
| BI | Ga0110932_10541381 | RND Antibiotic Efflux Pump |
| BI | Ga0110932_11763962 | RND Antibiotic Efflux Pump |
| BI | Ga0110932_10347301 | RND Antibiotic Efflux Pump |
| BI | Ga0110932_11083651 | RND Antibiotic Efflux Pump |
| BI | Ga0110932_10436061 | RND Antibiotic Efflux Pump |
| BI | Ga0110932_10614241 | RND Antibiotic Efflux Pump |
| BI | Ga0110932_10091451 | sul1 |
| BI | Ga0110932_11391251 | sul2 |
| BI | Ga0110932_11511841 | Tetracycline Resistance MFS Efflux Pump |
| H1 | Ga0110937_10083231 | AAC(6')-Ib-cr |
| H1 | Ga0110937_10029281 | AAC(6')-Ie-APH(2'')-Ia |
| H1 | Ga0110937_10262311 | AAC(6')-Ie-APH(2'')-Ia |
| H1 | Ga0110937_10242801 | AAC(6')-Ie-APH(2'')-Ia |
| H1 | Ga0110937_11266481 | AAC(6')-Ie-APH(2'')-Ia |
| H1 | Ga0110937_10050351 | AAC3 |
| H1 | Ga0110937_10246674 | AAC3 |
| H1 | Ga0110937_10170851 | AAC3 |
| H1 | Ga0110937_10125081 | AAC3-Ia |
| H1 | Ga0110937_10010361 | AAC6-Ib |
| H1 | Ga0110937_10001461 | aadA |
| H1 | Ga0110937_10008932 | aadA11 |
| H1 | Ga0110937_11213301 | aadA16 |
| H1 | Ga0110937_10001471 | aadA22 |
| H1 | Ga0110937_10010833 | aadA23 |
| H1 | Ga0110937_10013231 | aadA23 |
| H1 | Ga0110937_10313041 | aadA4 |
| H1 | Ga0110937_10021501 | ABC Antibiotic Efflux Pump |
| H1 | Ga0110937_10013381 | acrA |
| H1 | Ga0110937_10332931 | acrA |
| H1 | Ga0110937_10203641 | acrA |
| H1 | Ga0110937_11747031 | acrA |
| H1 | Ga0110937_10959971 | acrA |
| H1 | Ga0110937_10917071 | acrA |
| H1 | Ga0110937_10009364 | acrB |
| H1 | Ga0110937_10952651 | acrB |
| H1 | Ga0110937_10266481 | acrB |
| H1 | Ga0110937_10079711 | acrB |
| H1 | Ga0110937_10361151 | acrB |
| H1 | Ga0110937_10627631 | acrB |
| H1 | Ga0110937_10009381 | acrB |
| H1 | Ga0110937_10509181 | acrB |
| H1 | Ga0110937_10114211 | acrB |
| H1 | Ga0110937_10141731 | acrB |
| H1 | Ga0110937_10245761 | acrB |
| H1 | Ga0110937_10639781 | acrB |
| H1 | Ga0110937_11988841 | acrB |
| H1 | Ga0110937_10009371 | acrB |
| H1 | Ga0110937_11832451 | acrB |
| H1 | Ga0110937_10082305 | acrD |
| H1 | Ga0110937_10082304 | acrD |
| H1 | Ga0110937_10236182 | acrD |
| H1 | Ga0110937_10117691 | acrD |
| H1 | Ga0110937_10491741 | acrD |
| H1 | Ga0110937_10798761 | acrD |
| H1 | Ga0110937_10602132 | acrD |
| H1 | Ga0110937_10203631 | acrD |
| H1 | Ga0110937_11917361 | acrD |
| H1 | Ga0110937_10142431 | acrD |
| H1 | Ga0110937_10723751 | acrE |
| H1 | Ga0110937_10539231 | acrE |
| H1 | Ga0110937_10472171 | acrF |
| H1 | Ga0110937_10032091 | acrF |
| H1 | Ga0110937_10478641 | acrF |
| H1 | Ga0110937_10399261 | acrF |
| H1 | Ga0110937_12019651 | acrF |
| H1 | Ga0110937_12327851 | acrF |
| H1 | Ga0110937_10043171 | acrF |
| H1 | Ga0110937_11547971 | acrF |
| H1 | Ga0110937_10350651 | acrF |
| H1 | Ga0110937_10881531 | acrF |
| H1 | Ga0110937_11398431 | *bla*_ACT-28_ |
| H1 | Ga0110937_10773293 | *bla*_ACT-29_ |
| H1 | Ga0110937_10440441 | adeA-adeI |
| H1 | Ga0110937_10324441 | adeB |
| H1 | Ga0110937_12105261 | adeB |
| H1 | Ga0110937_11241531 | adeB |
| H1 | Ga0110937_10433811 | adeB |
| H1 | Ga0110937_12124971 | adeB |
| H1 | Ga0110937_11045211 | adeB |
| H1 | Ga0110937_10423681 | adeB |
| H1 | Ga0110937_11238711 | adeB |
| H1 | Ga0110937_10527821 | adeB |
| H1 | Ga0110937_11633881 | adeB |
| H1 | Ga0110937_11061392 | adeC-adeK-oprM |
| H1 | Ga0110937_11859862 | adeC-adeK-oprM |
| H1 | Ga0110937_11307811 | adeC-adeK-oprM |
| H1 | Ga0110937_11985791 | adeC-adeK-oprM |
| H1 | Ga0110937_10702841 | adeC-adeK-oprM |
| H1 | Ga0110937_11443581 | adeG |
| H1 | Ga0110937_12057191 | adeG |
| H1 | Ga0110937_11476091 | adeG |
| H1 | Ga0110937_10838041 | adeG |
| H1 | Ga0110937_10802541 | adeG |
| H1 | Ga0110937_10706872 | adeG |
| H1 | Ga0110937_11532661 | adeH |
| H1 | Ga0110937_11177141 | adeJ |
| H1 | Ga0110937_10083232 | ANT(2'')-Ia |
| H1 | Ga0110937_10406821 | ANT(6)-Ia |
| H1 | Ga0110937_10013221 | ANT3 |
| H1 | Ga0110937_10008661 | ANT3 |
| H1 | Ga0110937_11394611 | ANT3 |
| H1 | Ga0110937_11206221 | ANT3 |
| H1 | Ga0110937_11102832 | ANT6 |
| H1 | Ga0110937_10848971 | ANT6 |
| H1 | Ga0110937_10066783 | APH(6)-Id |
| H1 | Ga0110937_10141883 | APH3 |
| H1 | Ga0110937_10001567 | APH3" |
| H1 | Ga0110937_10017301 | APH3" |
| H1 | Ga0110937_10019998 | APH3" |
| H1 | Ga0110937_10066782 | APH3" |
| H1 | Ga0110937_10058411 | APH3" |
| H1 | Ga0110937_11510842 | APH3" |
| H1 | Ga0110937_11309211 | APH3" |
| H1 | Ga0110937_10662151 | APH3" |
| H1 | Ga0110937_10621562 | APH3" |
| H1 | Ga0110937_10142501 | APH3" |
| H1 | Ga0110937_10142511 | APH3" |
| H1 | Ga0110937_10265922 | APH3' |
| H1 | Ga0110937_10193061 | APH3' |
| H1 | Ga0110937_10637761 | APH3' |
| H1 | Ga0110937_10066801 | APH6 |
| H1 | Ga0110937_10066791 | APH6 |
| H1 | Ga0110937_10002904 | armA |
| H1 | Ga0110937_10116371 | armA |
| H1 | Ga0110937_10067425 | armA |
| H1 | Ga0110937_11018121 | arnA |
| H1 | Ga0110937_11281191 | arnA |
| H1 | Ga0110937_10213951 | arnA |
| H1 | Ga0110937_11163331 | arnA |
| H1 | Ga0110937_10213921 | arnA |
| H1 | Ga0110937_10213931 | arnA |
| H1 | Ga0110937_11399021 | arnA |
| H1 | Ga0110937_10027021 | arr-3 |
| H1 | Ga0110937_10499702 | bacA |
| H1 | Ga0110937_10081202 | bacA |
| H1 | Ga0110937_10791091 | bacA |
| H1 | Ga0110937_10364191 | baeR |
| H1 | Ga0110937_10396291 | baeR |
| H1 | Ga0110937_10780431 | baeR |
| H1 | Ga0110937_10780371 | baeR |
| H1 | Ga0110937_11757401 | baeR |
| H1 | Ga0110937_11842691 | baeR |
| H1 | Ga0110937_10364091 | baeR |
| H1 | Ga0110937_10869791 | baeR |
| H1 | Ga0110937_11195801 | baeR |
| H1 | Ga0110937_11779731 | baeR |
| H1 | Ga0110937_12110861 | baeS |
| H1 | Ga0110937_10863621 | baeS |
| H1 | Ga0110937_10599831 | baeS |
| H1 | Ga0110937_11610492 | baeS |
| H1 | Ga0110937_10212931 | Beta-lactamase Class A |
| H1 | Ga0110937_10099702 | Beta-lactamase Class A |
| H1 | Ga0110937_11695261 | Beta-lactamase Class A |
| H1 | Ga0110937_10768941 | Beta-lactamase Class A |
| H1 | Ga0110937_11797961 | Beta-lactamase Class A |
| H1 | Ga0110937_10926071 | BlaB_AERHY |
| H1 | Ga0110937_12221811 | *bla*_CMY-112_ |
| H1 | Ga0110937_10034422 | *bla*_CTX-M-15_ |
| H1 | Ga0110937_10141903 | *bla*_CTX-M-18_ |
| H1 | Ga0110937_10773294 | *bla*_DHA-6_ |
| H1 | Ga0110937_10209692 | *bla*_IMP-1_ |
| H1 | Ga0110937_100002322 | *bla*_KPC-2_ |
| H1 | Ga0110937_10122753 | *bla*_MOX-2_ |
| H1 | Ga0110937_11226771 | *bla*_NDM-1_ |
| H1 | Ga0110937_10653232 | *bla*_NDM-2_ |
| H1 | Ga0110937_10008732 | *bla*_OXA-10_ |
| H1 | Ga0110937_10740691 | *bla*_OXA-10_ |
| H1 | Ga0110937_10340681 | *bla*_OXA-129_ |
| H1 | Ga0110937_10004592 | *bla*_OXA-2_ |
| H1 | Ga0110937_10483141 | *bla*_OXA-21_ |
| H1 | Ga0110937_10393051 | *bla*_OXA-21_ |
| H1 | Ga0110937_10004591 | *bla*_OXA-21_ |
| H1 | Ga0110937_11310751 | *bla*_PER-3_ |
| H1 | Ga0110937_10010351 | *bla*_PSE-1_ |
| H1 | Ga0110937_10102746 | *bla*_SHV-36_ |
| H1 | Ga0110937_10027231 | *bla*_TEM-157_ |
| H1 | Ga0110937_10463432 | *bla*_VIM-2_ |
| H1 | Ga0110937_10653231 | bleomycin resistance protein (BRP) |
| H1 | Ga0110937_10002902 | cat |
| H1 | Ga0110937_11590761 | CAT1_CLOPF |
| H1 | Ga0110937_10726711 | CAT1_CLOPF |
| H1 | Ga0110937_10486641 | ceoB |
| H1 | Ga0110937_10465952 | ceoB |
| H1 | Ga0110937_10788431 | Cfr 23 Ribosomal RNA Methyltransferase |
| H1 | Ga0110937_10788441 | Cfr 23 Ribosomal RNA Methyltransferase |
| H1 | Ga0110937_10099711 | cfxA6 |
| H1 | Ga0110937_12016651 | cfxA6 |
| H1 | Ga0110937_10027022 | Chloramphenicol Acetyltransferase CAT |
| H1 | Ga0110937_10066781 | Chloramphenicol Acetyltransferase CAT |
| H1 | Ga0110937_10097481 | Chloramphenicol Acetyltransferase CAT |
| H1 | Ga0110937_10643531 | Chloramphenicol Acetyltransferase CAT |
| H1 | Ga0110937_12177561 | Chloramphenicol Acetyltransferase CAT |
| H1 | Ga0110937_11910801 | Chloramphenicol Acetyltransferase CAT |
| H1 | Ga0110937_10085932 | Chloramphenicol Acetyltransferase CAT |
| H1 | Ga0110937_11544271 | Chloramphenicol Acetyltransferase CAT |
| H1 | Ga0110937_10008731 | Chloramphenicol Efflux Pump |
| H1 | Ga0110937_10332121 | Chloramphenicol Efflux Pump |
| H1 | Ga0110937_10000037 | cpxA |
| H1 | Ga0110937_10068045 | cpxA |
| H1 | Ga0110937_10132663 | cpxA |
| H1 | Ga0110937_12255311 | cpxA |
| H1 | Ga0110937_12399661 | cpxA |
| H1 | Ga0110937_12157921 | cpxA |
| H1 | Ga0110937_10437011 | cpxA |
| H1 | Ga0110937_10731241 | cpxA |
| H1 | Ga0110937_10068044 | cpxR |
| H1 | Ga0110937_10132662 | cpxR |
| H1 | Ga0110937_10197211 | cpxR |
| H1 | Ga0110937_10003338 | CRP |
| H1 | Ga0110937_10042185 | CRP |
| H1 | Ga0110937_10589812 | CRP |
| H1 | Ga0110937_10296861 | CRP |
| H1 | Ga0110937_10296751 | CRP |
| H1 | Ga0110937_10281061 | CRP |
| H1 | Ga0110937_10197532 | CRP |
| H1 | Ga0110937_10128071 | CRP |
| H1 | Ga0110937_10762851 | CRP |
| H1 | Ga0110937_10197541 | CRP |
| H1 | Ga0110937_10894791 | CRP |
| H1 | Ga0110937_11507281 | CRP |
| H1 | Ga0110937_10438761 | dfrA1 |
| H1 | Ga0110937_10010832 | dfrA14 |
| H1 | Ga0110937_10013232 | dfrA15 |
| H1 | Ga0110937_11511601 | dfrB1 |
| H1 | Ga0110937_11008413 | dfrF |
| H1 | Ga0110937_12162601 | dfrF |
| H1 | Ga0110937_10856752 | emrA |
| H1 | Ga0110937_11228732 | emrA |
| H1 | Ga0110937_10856743 | emrA |
| H1 | Ga0110937_10472362 | emrA |
| H1 | Ga0110937_10472371 | emrA |
| H1 | Ga0110937_10833911 | emrA |
| H1 | Ga0110937_10472351 | emrA |
| H1 | Ga0110937_11211471 | emrA |
| H1 | Ga0110937_11211461 | emrA |
| H1 | Ga0110937_10158704 | emrB |
| H1 | Ga0110937_10526631 | emrB |
| H1 | Ga0110937_10472361 | emrB |
| H1 | Ga0110937_10941101 | emrB |
| H1 | Ga0110937_10195831 | emrB |
| H1 | Ga0110937_10702491 | emrB |
| H1 | Ga0110937_10550621 | emrB |
| H1 | Ga0110937_11177501 | emrB |
| H1 | Ga0110937_10671751 | emrB |
| H1 | Ga0110937_11111211 | emrB |
| H1 | Ga0110937_11164001 | emrB |
| H1 | Ga0110937_10941111 | emrB |
| H1 | Ga0110937_11635951 | emrB |
| H1 | Ga0110937_12316261 | emrB |
| H1 | Ga0110937_10038891 | emrD |
| H1 | Ga0110937_10031862 | emrD |
| H1 | Ga0110937_11394921 | emrE |
| H1 | Ga0110937_10174161 | emrK |
| H1 | Ga0110937_10121871 | emrK |
| H1 | Ga0110937_10112453 | emrR |
| H1 | Ga0110937_10856742 | emrR |
| H1 | Ga0110937_11228731 | emrR |
| H1 | Ga0110937_10856751 | emrR |
| H1 | Ga0110937_10309931 | ereA2 |
| H1 | Ga0110937_11249561 | ereB |
| H1 | Ga0110937_10154282 | Erm 23S ribosomal RNA Methyltransferase |
| H1 | Ga0110937_10073361 | ermB |
| H1 | Ga0110937_10073351 | ermB |
| H1 | Ga0110937_10841141 | ermF |
| H1 | Ga0110937_10134211 | ermF |
| H1 | Ga0110937_10585681 | ermX |
| H1 | Ga0110937_10190474 | Fluoroquinolone Resistant DNA Topoisomerase |
| H1 | Ga0110937_10425601 | Fluoroquinolone Resistant DNA Topoisomerase |
| H1 | Ga0110937_10193771 | Fluoroquinolone Resistant DNA Topoisomerase |
| H1 | Ga0110937_10147381 | Fluoroquinolone Resistant DNA Topoisomerase |
| H1 | Ga0110937_10396471 | Fluoroquinolone Resistant DNA Topoisomerase |
| H1 | Ga0110937_10768012 | Fluoroquinolone Resistant DNA Topoisomerase |
| H1 | Ga0110937_11158081 | Fluoroquinolone Resistant DNA Topoisomerase |
| H1 | Ga0110937_10012201 | Fluoroquinolone Resistant DNA Topoisomerase |
| H1 | Ga0110937_11345181 | Fluoroquinolone Resistant DNA Topoisomerase |
| H1 | Ga0110937_10746881 | Fluoroquinolone Resistant DNA Topoisomerase |
| H1 | Ga0110937_10273892 | Fluoroquinolone Resistant DNA Topoisomerase |
| H1 | Ga0110937_10130433 | Fluoroquinolone Resistant DNA Topoisomerase |
| H1 | Ga0110937_10273902 | Fluoroquinolone Resistant DNA Topoisomerase |
| H1 | Ga0110937_10142301 | Fluoroquinolone Resistant DNA Topoisomerase |
| H1 | Ga0110937_10587061 | Fluoroquinolone Resistant DNA Topoisomerase |
| H1 | Ga0110937_12249641 | Fluoroquinolone Resistant DNA Topoisomerase |
| H1 | Ga0110937_11227191 | Fluoroquinolone Resistant DNA Topoisomerase |
| H1 | Ga0110937_10915111 | Fluoroquinolone Resistant DNA Topoisomerase |
| H1 | Ga0110937_10236281 | Fluoroquinolone Resistant DNA Topoisomerase |
| H1 | Ga0110937_10012171 | Fluoroquinolone Resistant DNA Topoisomerase |
| H1 | Ga0110937_10356551 | Fluoroquinolone Resistant DNA Topoisomerase |
| H1 | Ga0110937_12233991 | Fluoroquinolone Resistant DNA Topoisomerase |
| H1 | Ga0110937_10012191 | Fluoroquinolone Resistant DNA Topoisomerase |
| H1 | Ga0110937_10333041 | Fluoroquinolone Resistant DNA Topoisomerase |
| H1 | Ga0110937_10688301 | Fluoroquinolone Resistant DNA Topoisomerase |
| H1 | Ga0110937_10273911 | Fluoroquinolone Resistant DNA Topoisomerase |
| H1 | Ga0110937_10289991 | Fluoroquinolone Resistant DNA Topoisomerase |
| H1 | Ga0110937_11369001 | Fluoroquinolone Resistant DNA Topoisomerase |
| H1 | Ga0110937_11183121 | Fluoroquinolone Resistant DNA Topoisomerase |
| H1 | Ga0110937_11215731 | Fluoroquinolone Resistant DNA Topoisomerase |
| H1 | Ga0110937_10332731 | Fluoroquinolone Resistant DNA Topoisomerase |
| H1 | Ga0110937_10394771 | Fluoroquinolone Resistant DNA Topoisomerase |
| H1 | Ga0110937_12187341 | Fluoroquinolone Resistant DNA Topoisomerase |
| H1 | Ga0110937_10438831 | Fluoroquinolone Resistant DNA Topoisomerase |
| H1 | Ga0110937_10590431 | Fluoroquinolone Resistant DNA Topoisomerase |
| H1 | Ga0110937_11183131 | Fluoroquinolone Resistant DNA Topoisomerase |
| H1 | Ga0110937_10221341 | Fluoroquinolone Resistant DNA Topoisomerase |
| H1 | Ga0110937_10424641 | Fluoroquinolone Resistant DNA Topoisomerase |
| H1 | Ga0110937_10438811 | Fluoroquinolone Resistant DNA Topoisomerase |
| H1 | Ga0110937_10438821 | Fluoroquinolone Resistant DNA Topoisomerase |
| H1 | Ga0110937_10537041 | Fluoroquinolone Resistant DNA Topoisomerase |
| H1 | Ga0110937_10915301 | Fluoroquinolone Resistant DNA Topoisomerase |
| H1 | Ga0110937_10949681 | Fluoroquinolone Resistant DNA Topoisomerase |
| H1 | Ga0110937_11019321 | Fluoroquinolone Resistant DNA Topoisomerase |
| H1 | Ga0110937_11646511 | Fluoroquinolone Resistant DNA Topoisomerase |
| H1 | Ga0110937_11665891 | Fluoroquinolone Resistant DNA Topoisomerase |
| H1 | Ga0110937_11769311 | Fluoroquinolone Resistant DNA Topoisomerase |
| H1 | Ga0110937_11987051 | Fluoroquinolone Resistant DNA Topoisomerase |
| H1 | Ga0110937_12442331 | Fluoroquinolone Resistant DNA Topoisomerase |
| H1 | Ga0110937_11980402 | fosA |
| H1 | Ga0110937_12371972 | fosA |
| H1 | Ga0110937_10326485 | fosA2 |
| H1 | Ga0110937_10047959 | fosA5 |
| H1 | Ga0110937_12032491 | gadX |
| H1 | Ga0110937_10007971 | *bla*_GES-1_ |
| H1 | Ga0110937_10794143 | H-NS |
| H1 | Ga0110937_10425392 | H-NS |
| H1 | Ga0110937_10343202 | H-NS |
| H1 | Ga0110937_11084521 | *bla*_LCR-1_ |
| H1 | Ga0110937_10727421 | lnuB |
| H1 | Ga0110937_11062661 | lnuC |
| H1 | Ga0110937_10095821 | lnuC |
| H1 | Ga0110937_10727422 | lsaE |
| H1 | Ga0110937_10586292 | lsaE |
| H1 | Ga0110937_10790813 | macA |
| H1 | Ga0110937_11143911 | macA |
| H1 | Ga0110937_10209172 | macA |
| H1 | Ga0110937_11907571 | macA |
| H1 | Ga0110937_10273141 | macA |
| H1 | Ga0110937_11611711 | macA |
| H1 | Ga0110937_12279211 | macB |
| H1 | Ga0110937_11143912 | macB |
| H1 | Ga0110937_10706041 | macB |
| H1 | Ga0110937_10437991 | macB |
| H1 | Ga0110937_10790811 | macB |
| H1 | Ga0110937_10790535 | marA |
| H1 | Ga0110937_11065293 | marA |
| H1 | Ga0110937_12024562 | marA |
| H1 | Ga0110937_11483281 | marA |
| H1 | Ga0110937_10331932 | mdfA |
| H1 | Ga0110937_10275372 | mdfA |
| H1 | Ga0110937_10140631 | mdfA |
| H1 | Ga0110937_10140621 | mdfA |
| H1 | Ga0110937_11261001 | mdfA |
| H1 | Ga0110937_10044871 | mdfA |
| H1 | Ga0110937_10044861 | mdfA |
| H1 | Ga0110937_10400841 | mdsB |
| H1 | Ga0110937_11946121 | mdsB |
| H1 | Ga0110937_10439161 | mdsB |
| H1 | Ga0110937_10751051 | mdtA |
| H1 | Ga0110937_10025081 | mdtA |
| H1 | Ga0110937_10025101 | mdtA |
| H1 | Ga0110937_10759971 | mdtA |
| H1 | Ga0110937_10025098 | mdtB |
| H1 | Ga0110937_10222441 | mdtB |
| H1 | Ga0110937_10025097 | mdtB |
| H1 | Ga0110937_10452581 | mdtB |
| H1 | Ga0110937_10832351 | mdtB |
| H1 | Ga0110937_10128001 | mdtB |
| H1 | Ga0110937_11576081 | mdtB |
| H1 | Ga0110937_10909891 | mdtB |
| H1 | Ga0110937_10363061 | mdtB |
| H1 | Ga0110937_11212661 | mdtB |
| H1 | Ga0110937_10901111 | mdtB |
| H1 | Ga0110937_12010921 | mdtB |
| H1 | Ga0110937_10721301 | mdtB |
| H1 | Ga0110937_10362941 | mdtB |
| H1 | Ga0110937_10128011 | mdtB |
| H1 | Ga0110937_10734691 | mdtB |
| H1 | Ga0110937_10025099 | mdtC |
| H1 | Ga0110937_100250910 | mdtC |
| H1 | Ga0110937_10298231 | mdtC |
| H1 | Ga0110937_10993231 | mdtC |
| H1 | Ga0110937_10222442 | mdtC |
| H1 | Ga0110937_10174632 | mdtC |
| H1 | Ga0110937_10861261 | mdtC |
| H1 | Ga0110937_11067711 | mdtC |
| H1 | Ga0110937_10523261 | mdtC |
| H1 | Ga0110937_11067701 | mdtC |
| H1 | Ga0110937_10015401 | mdtC |
| H1 | Ga0110937_10262721 | mdtC |
| H1 | Ga0110937_11304251 | mdtC |
| H1 | Ga0110937_10174631 | mdtD |
| H1 | Ga0110937_10229311 | mdtG |
| H1 | Ga0110937_10229132 | mdtG |
| H1 | Ga0110937_12007911 | mdtG |
| H1 | Ga0110937_10097141 | mdtH |
| H1 | Ga0110937_10413753 | mdtH |
| H1 | Ga0110937_10752872 | mdtH |
| H1 | Ga0110937_10413761 | mdtH |
| H1 | Ga0110937_12166821 | mdtH |
| H1 | Ga0110937_10761901 | mdtK |
| H1 | Ga0110937_10662811 | mdtK |
| H1 | Ga0110937_10737551 | mdtK |
| H1 | Ga0110937_10114611 | mdtL |
| H1 | Ga0110937_10015906 | mdtL |
| H1 | Ga0110937_10276271 | mdtL |
| H1 | Ga0110937_10340541 | mdtL |
| H1 | Ga0110937_11927951 | mdtL |
| H1 | Ga0110937_11034241 | mdtM |
| H1 | Ga0110937_10178352 | mdtN |
| H1 | Ga0110937_11598631 | mdtP |
| H1 | Ga0110937_11868691 | mdtP |
| H1 | Ga0110937_10434511 | mefA |
| H1 | Ga0110937_10110111 | MexA |
| H1 | Ga0110937_10694061 | mexB |
| H1 | Ga0110937_10009391 | mexB |
| H1 | Ga0110937_10104181 | mexB |
| H1 | Ga0110937_11619171 | mexC |
| H1 | Ga0110937_10445462 | mexD |
| H1 | Ga0110937_11822171 | mexD |
| H1 | Ga0110937_10339921 | mexD |
| H1 | Ga0110937_10464861 | mexE |
| H1 | Ga0110937_11048932 | mexF |
| H1 | Ga0110937_11780411 | mexF |
| H1 | Ga0110937_11369101 | mexF |
| H1 | Ga0110937_10888961 | mexF |
| H1 | Ga0110937_10466141 | mexF |
| H1 | Ga0110937_11753171 | mexH |
| H1 | Ga0110937_11332862 | mexJ |
| H1 | Ga0110937_10241002 | mexK |
| H1 | Ga0110937_10145432 | mexK |
| H1 | Ga0110937_12232291 | mexK |
| H1 | Ga0110937_11332861 | mexK |
| H1 | Ga0110937_10400321 | mexK |
| H1 | Ga0110937_11641271 | mexK |
| H1 | Ga0110937_12433911 | mexL |
| H1 | Ga0110937_10553881 | mexL |
| H1 | Ga0110937_10733991 | mexN |
| H1 | Ga0110937_10950852 | mexN |
| H1 | Ga0110937_10860801 | mexP |
| H1 | Ga0110937_10306711 | mexQ |
| H1 | Ga0110937_11685091 | mexQ |
| H1 | Ga0110937_10959021 | mexS |
| H1 | Ga0110937_12042341 | mexT |
| H1 | Ga0110937_12243251 | mexT |
| H1 | Ga0110937_11741521 | mexV |
| H1 | Ga0110937_12152551 | mexY |
| H1 | Ga0110937_10478841 | mexY |
| H1 | Ga0110937_10031861 | MFS Antibiotic Efflux Pump |
| H1 | Ga0110937_10604191 | MFS Antibiotic Efflux Pump |
| H1 | Ga0110937_10956331 | MFS Antibiotic Efflux Pump |
| H1 | Ga0110937_10017283 | mphA |
| H1 | Ga0110937_10232721 | msbA |
| H1 | Ga0110937_10645011 | msbA |
| H1 | Ga0110937_11883951 | msbA |
| H1 | Ga0110937_10021463 | msrE |
| H1 | Ga0110937_11000741 | mtrD |
| H1 | Ga0110937_10410901 | opmD |
| H1 | Ga0110937_10308571 | opmE |
| H1 | Ga0110937_10238331 | opmH |
| H1 | Ga0110937_11284041 | opmH |
| H1 | Ga0110937_11628001 | opmH |
| H1 | Ga0110937_10000851 | *bla*_OXA-1_ |
| H1 | Ga0110937_10013222 | *bla*_OXA-1_ |
| H1 | Ga0110937_10276341 | *bla*_OXA-12_ |
| H1 | Ga0110937_10874151 | *bla*_OXA-12_ |
| H1 | Ga0110937_10393701 | *bla*_OXA-226_ |
| H1 | Ga0110937_10134212 | *bla*_OXA-347_ |
| H1 | Ga0110937_10153901 | *bla*_OXA-420_ |
| H1 | Ga0110937_11160501 | *bla*_OXA-1_ |
| H1 | Ga0110937_10638401 | phoP |
| H1 | Ga0110937_10917152 | phoP |
| H1 | Ga0110937_10917153 | phoQ |
| H1 | Ga0110937_10192341 | phoQ |
| H1 | Ga0110937_10665941 | phoQ |
| H1 | Ga0110937_10192351 | phoQ |
| H1 | Ga0110937_10304041 | phoQ |
| H1 | Ga0110937_11206082 | PmrA |
| H1 | Ga0110937_10362892 | PmrA |
| H1 | Ga0110937_11149392 | PmrA |
| H1 | Ga0110937_10624142 | PmrA |
| H1 | Ga0110937_10701921 | PmrA |
| H1 | Ga0110937_10701922 | PmrA |
| H1 | Ga0110937_10918072 | PmrE |
| H1 | Ga0110937_11314832 | PmrE |
| H1 | Ga0110937_10709072 | PmrE |
| H1 | Ga0110937_11567511 | PmrE |
| H1 | Ga0110937_10157941 | PmrE |
| H1 | Ga0110937_10404031 | PmrE |
| H1 | Ga0110937_10741561 | PmrE |
| H1 | Ga0110937_10057962 | PmrF |
| H1 | Ga0110937_11079351 | PmrF |
| H1 | Ga0110937_10025402 | QnrB6 |
| H1 | Ga0110937_10058123 | QnrS2 |
| H1 | Ga0110937_10040141 | QnrVC4 |
| H1 | Ga0110937_100163213 | Quinolone Resistance Protein Qnr |
| H1 | Ga0110937_10013122 | ramA |
| H1 | Ga0110937_10685351 | ramA |
| H1 | Ga0110937_10919471 | ramA |
| H1 | Ga0110937_12172811 | ramA |
| H1 | Ga0110937_10246622 | RND Antibiotic Efflux Pump |
| H1 | Ga0110937_10318802 | RND Antibiotic Efflux Pump |
| H1 | Ga0110937_10262711 | RND Antibiotic Efflux Pump |
| H1 | Ga0110937_11989652 | RND Antibiotic Efflux Pump |
| H1 | Ga0110937_11407551 | RND Antibiotic Efflux Pump |
| H1 | Ga0110937_10507451 | RND Antibiotic Efflux Pump |
| H1 | Ga0110937_12338111 | RND Antibiotic Efflux Pump |
| H1 | Ga0110937_10821991 | RND Antibiotic Efflux Pump |
| H1 | Ga0110937_10538461 | RND Antibiotic Efflux Pump |
| H1 | Ga0110937_12294921 | RND Antibiotic Efflux Pump |
| H1 | Ga0110937_11167041 | RND Antibiotic Efflux Pump |
| H1 | Ga0110937_10255251 | RND Antibiotic Efflux Pump |
| H1 | Ga0110937_10821641 | RND Antibiotic Efflux Pump |
| H1 | Ga0110937_10113532 | RND Antibiotic Efflux Pump |
| H1 | Ga0110937_10533371 | RND Antibiotic Efflux Pump |
| H1 | Ga0110937_11315461 | RND Antibiotic Efflux Pump |
| H1 | Ga0110937_11332791 | RND Antibiotic Efflux Pump |
| H1 | Ga0110937_10102471 | robA |
| H1 | Ga0110937_10615061 | robA |
| H1 | Ga0110937_10093842 | robA |
| H1 | Ga0110937_10703711 | robA |
| H1 | Ga0110937_11997872 | robA |
| H1 | Ga0110937_11416941 | robA |
| H1 | Ga0110937_11223241 | romA |
| H1 | Ga0110937_11482731 | romA |
| H1 | Ga0110937_10920031 | romA |
| H1 | Ga0110937_10183492 | rosB |
| H1 | Ga0110937_10095123 | rosB |
| H1 | Ga0110937_10095101 | rosB |
| H1 | Ga0110937_10095111 | rosB |
| H1 | Ga0110937_11102831 | sat-4 |
| H1 | Ga0110937_10265921 | sat-4 |
| H1 | Ga0110937_10102751 | *bla*_SHV-123_ |
| H1 | Ga0110937_10444053 | smeE |
| H1 | Ga0110937_10084052 | soxR |
| H1 | Ga0110937_10499601 | soxR |
| H1 | Ga0110937_10222253 | soxR |
| H1 | Ga0110937_10222261 | soxR |
| H1 | Ga0110937_11267671 | soxR |
| H1 | Ga0110937_10014252 | sul1 |
| H1 | Ga0110937_100002219 | sul2 |
| H1 | Ga0110937_10045171 | *bla*_TEM-211_ |
| H1 | Ga0110937_11052181 | tet40 |
| H1 | Ga0110937_10901891 | TetD |
| H1 | Ga0110937_10977661 | tetO |
| H1 | Ga0110937_10163881 | tetO |
| H1 | Ga0110937_10004701 | tetO |
| H1 | Ga0110937_10206745 | Tetracycline Resistance MFS Efflux Pump |
| H1 | Ga0110937_10037653 | Tetracycline Resistance MFS Efflux Pump |
| H1 | Ga0110937_10358312 | Tetracycline Resistance MFS Efflux Pump |
| H1 | Ga0110937_10245232 | Tetracycline Resistance MFS Efflux Pump |
| H1 | Ga0110937_10259992 | Tetracycline Resistance MFS Efflux Pump |
| H1 | Ga0110937_11158711 | Tetracycline Resistance MFS Efflux Pump |
| H1 | Ga0110937_10150181 | Tetracycline Resistance MFS Efflux Pump |
| H1 | Ga0110937_10149061 | Tetracycline Resistance MFS Efflux Pump |
| H1 | Ga0110937_10720881 | Tetracycline Resistance MFS Efflux Pump |
| H1 | Ga0110937_10176881 | Tetracycline Resistance Ribosomal Protection Protein |
| H1 | Ga0110937_10160002 | Tetracycline Resistance Ribosomal Protection Protein |
| H1 | Ga0110937_10967631 | Tetracycline Resistance Ribosomal Protection Protein |
| H1 | Ga0110937_10149071 | Tetracycline Resistance Ribosomal Protection Protein |
| H1 | Ga0110937_11922301 | Tetracycline Resistance Ribosomal Protection Protein |
| H1 | Ga0110937_10438171 | Tetracycline Resistance Ribosomal Protection Protein |
| H1 | Ga0110937_10492961 | Tetracycline Resistance Ribosomal Protection Protein |
| H1 | Ga0110937_11985951 | Tetracycline Resistance Ribosomal Protection Protein |
| H1 | Ga0110937_10905211 | Tetracycline Resistance Ribosomal Protection Protein |
| H1 | Ga0110937_10822691 | Tetracycline Resistance Ribosomal Protection Protein |
| H1 | Ga0110937_11008391 | Tetracycline Resistance Ribosomal Protection Protein |
| H1 | Ga0110937_11459731 | Tetracycline Resistance Ribosomal Protection Protein |
| H1 | Ga0110937_10134218 | tetX |
| H1 | Ga0110937_10453821 | tetY |
| H1 | Ga0110937_10015986 | tolC |
| H1 | Ga0110937_10370663 | tolC |
| H1 | Ga0110937_11567431 | tolC |
| H1 | Ga0110937_10370411 | tolC |
| H1 | Ga0110937_10171101 | tolC |
| H1 | Ga0110937_11914241 | TriA |
| H1 | Ga0110937_10500731 | TriB |
| H1 | Ga0110937_10500732 | TriC |
| H1 | Ga0110937_12330031 | TriC |
| H1 | Ga0110937_11998661 | vanB |
| H1 | Ga0110937_11481882 | vanB |
| H1 | Ga0110937_12138392 | vanG |
| H1 | Ga0110937_11998662 | vanH |
| H1 | Ga0110937_10643621 | vanR |
| H1 | Ga0110937_12168792 | vanR |
| H1 | Ga0110937_11865181 | vanS |
| H1 | Ga0110937_10643622 | vanS |
| H1 | Ga0110937_11249911 | vanS |
| H1 | Ga0110937_12029661 | vanT |
| H1 | Ga0110937_11268891 | vanX |
| H1 | Ga0110937_11481881 | vanX |
| H1 | Ga0110937_12138391 | vanXYG |
| H1 | Ga0110937_11212931 | vanY |
| H1 | Ga0110937_11790132 | vanYA |
| H1 | Ga0110937_10083233 | *bla*_VEB-1a_ |
| H2 | Ga0110938_10020411 | AAC(6')-Ib9 |
| H2 | Ga0110938_10613842 | AAC(6')-Ie-APH(2'')-Ia |
| H2 | Ga0110938_10666351 | AAC(6')-Ie-APH(2'')-Ia |
| H2 | Ga0110938_12270881 | AAC(6')-Ip |
| H2 | Ga0110938_10017232 | AAC3 |
| H2 | Ga0110938_10046371 | AAC6-Ib |
| H2 | Ga0110938_11949541 | AAC6-II |
| H2 | Ga0110938_11201682 | aadA15 |
| H2 | Ga0110938_10506962 | AAC3-Ia |
| H2 | Ga0110938_10085291 | ABC Antibiotic Efflux Pump |
| H2 | Ga0110938_10602891 | abeM |
| H2 | Ga0110938_12933341 | abeM |
| H2 | Ga0110938_10194054 | acrA |
| H2 | Ga0110938_11955251 | acrA |
| H2 | Ga0110938_12719601 | acrA |
| H2 | Ga0110938_10194055 | acrB |
| H2 | Ga0110938_11709031 | acrB |
| H2 | Ga0110938_10351481 | acrB |
| H2 | Ga0110938_11558361 | acrB |
| H2 | Ga0110938_12566711 | acrB |
| H2 | Ga0110938_11290541 | acrB |
| H2 | Ga0110938_10909621 | acrB |
| H2 | Ga0110938_10131611 | acrB |
| H2 | Ga0110938_10351491 | acrB |
| H2 | Ga0110938_11238361 | acrB |
| H2 | Ga0110938_13131891 | acrB |
| H2 | Ga0110938_12858011 | acrD |
| H2 | Ga0110938_10637511 | acrD |
| H2 | Ga0110938_10131601 | acrD |
| H2 | Ga0110938_11387151 | acrD |
| H2 | Ga0110938_10297331 | acrE |
| H2 | Ga0110938_10215541 | acrE |
| H2 | Ga0110938_11880211 | acrE |
| H2 | Ga0110938_10215542 | acrF |
| H2 | Ga0110938_12915581 | acrF |
| H2 | Ga0110938_10721942 | acrF |
| H2 | Ga0110938_11181101 | acrF |
| H2 | Ga0110938_11823211 | acrF |
| H2 | Ga0110938_10812201 | acrF |
| H2 | Ga0110938_11181111 | acrF |
| H2 | Ga0110938_10542514 | acrS |
| H2 | Ga0110938_11239221 | *bla*_ACT-29_ |
| H2 | Ga0110938_10926452 | adeA-adeI |
| H2 | Ga0110938_10344111 | adeA-adeI |
| H2 | Ga0110938_10928732 | adeB |
| H2 | Ga0110938_11569972 | adeB |
| H2 | Ga0110938_12400551 | adeB |
| H2 | Ga0110938_12918501 | adeB |
| H2 | Ga0110938_11153611 | adeB |
| H2 | Ga0110938_10721181 | adeB |
| H2 | Ga0110938_10860101 | adeB |
| H2 | Ga0110938_10409321 | adeB |
| H2 | Ga0110938_10590012 | adeC-adeK-oprM |
| H2 | Ga0110938_11129981 | adeC-adeK-oprM |
| H2 | Ga0110938_12275201 | adeC-adeK-oprM |
| H2 | Ga0110938_12366761 | adeC-adeK-oprM |
| H2 | Ga0110938_11129971 | adeC-adeK-oprM |
| H2 | Ga0110938_11005281 | adeJ |
| H2 | Ga0110938_11804821 | adeN |
| H2 | Ga0110938_12817701 | adeR |
| H2 | Ga0110938_12300871 | adeR |
| H2 | Ga0110938_10271933 | ampC |
| H2 | Ga0110938_10278931 | ampC |
| H2 | Ga0110938_11146982 | ampC |
| H2 | Ga0110938_10028161 | ANT(2'')-Ia |
| H2 | Ga0110938_10423381 | ANT(6)-Ia |
| H2 | Ga0110938_10028151 | ANT2 |
| H2 | Ga0110938_10080881 | ANT3 |
| H2 | Ga0110938_10138991 | ANT3 |
| H2 | Ga0110938_11485891 | ANT3 |
| H2 | Ga0110938_11485881 | ANT3 |
| H2 | Ga0110938_10839051 | ANT6 |
| H2 | Ga0110938_10720963 | ANT6 |
| H2 | Ga0110938_10974621 | ANT6 |
| H2 | Ga0110938_10863491 | ANT6 |
| H2 | Ga0110938_12523191 | APH(2'')-IIa |
| H2 | Ga0110938_10004482 | APH3 |
| H2 | Ga0110938_10116041 | APH3 |
| H2 | Ga0110938_10005216 | APH3" |
| H2 | Ga0110938_10281748 | APH3" |
| H2 | Ga0110938_100863712 | APH3" |
| H2 | Ga0110938_10047862 | APH3" |
| H2 | Ga0110938_10191212 | APH3" |
| H2 | Ga0110938_10805861 | APH3" |
| H2 | Ga0110938_10255301 | APH3" |
| H2 | Ga0110938_11825291 | APH3" |
| H2 | Ga0110938_10104691 | APH3" |
| H2 | Ga0110938_12395682 | APH3" |
| H2 | Ga0110938_11076151 | APH3" |
| H2 | Ga0110938_10604841 | APH3" |
| H2 | Ga0110938_11862841 | APH3" |
| H2 | Ga0110938_10720961 | APH3' |
| H2 | Ga0110938_10622421 | APH3' |
| H2 | Ga0110938_100863711 | APH6 |
| H2 | Ga0110938_10020071 | armA |
| H2 | Ga0110938_12191331 | armA |
| H2 | Ga0110938_10179452 | arnA |
| H2 | Ga0110938_11192861 | arnA |
| H2 | Ga0110938_12299241 | arnA |
| H2 | Ga0110938_10231541 | arnA |
| H2 | Ga0110938_11319021 | arnA |
| H2 | Ga0110938_10243141 | arr-3 |
| H2 | Ga0110938_11254591 | bacA |
| H2 | Ga0110938_10376442 | bacA |
| H2 | Ga0110938_11601552 | bacA |
| H2 | Ga0110938_11439031 | bacA |
| H2 | Ga0110938_11104321 | bacA |
| H2 | Ga0110938_11454981 | bacA |
| H2 | Ga0110938_11082691 | bacA |
| H2 | Ga0110938_10101041 | bacA |
| H2 | Ga0110938_12574591 | baeR |
| H2 | Ga0110938_11703451 | baeR |
| H2 | Ga0110938_11265311 | baeR |
| H2 | Ga0110938_10697741 | baeR |
| H2 | Ga0110938_11862131 | baeS |
| H2 | Ga0110938_11589521 | baeS |
| H2 | Ga0110938_12518661 | baeS |
| H2 | Ga0110938_10015321 | Beta-lactamase Class A |
| H2 | Ga0110938_10274591 | Beta-lactamase Class A |
| H2 | Ga0110938_10110402 | BlaB_AERHY |
| H2 | Ga0110938_11925641 | BLAC_RHOCA |
| H2 | Ga0110938_10214751 | BLAC_RHOCA |
| H2 | Ga0110938_10050681 | *bla*_CTX-M-15_ |
| H2 | Ga0110938_10059341 | *bla*_CTX-M-18_ |
| H2 | Ga0110938_13099461 | *bla*_DHA-7_ |
| H2 | Ga0110938_10199882 | *bla*_IMP-4_ |
| H2 | Ga0110938_100066613 | *bla*_KPC-2_ |
| H2 | Ga0110938_12137891 | *bla*_OXA-129_ |
| H2 | Ga0110938_10796541 | *bla*_OXA-129_ |
| H2 | Ga0110938_10184901 | *bla*_OXA-2_ |
| H2 | Ga0110938_10184911 | *bla*_OXA-21_ |
| H2 | Ga0110938_10363931 | *bla*_PER-3_ |
| H2 | Ga0110938_10055851 | *bla*_PSE-1_ |
| H2 | Ga0110938_11721882 | *bla*_SHV-36_ |
| H2 | Ga0110938_10266521 | *bla*_VIM-11_ |
| H2 | Ga0110938_12091691 | *bla*_VIM-2_ |
| H2 | Ga0110938_10736381 | bleomycin resistance protein (BRP) |
| H2 | Ga0110938_10687121 | cat |
| H2 | Ga0110938_11910691 | Cfr 23 Ribosomal RNA Methyltransferase |
| H2 | Ga0110938_10661821 | Cfr 23 Ribosomal RNA Methyltransferase |
| H2 | Ga0110938_10010793 | cfxA6 |
| H2 | Ga0110938_10047861 | Chloramphenicol Acetyltransferase CAT |
| H2 | Ga0110938_10054281 | Chloramphenicol Acetyltransferase CAT |
| H2 | Ga0110938_10411652 | Chloramphenicol Acetyltransferase CAT |
| H2 | Ga0110938_11753751 | Chloramphenicol Acetyltransferase CAT |
| H2 | Ga0110938_11062151 | Chloramphenicol Acetyltransferase CAT |
| H2 | Ga0110938_12547532 | Chloramphenicol Acetyltransferase CAT |
| H2 | Ga0110938_10781951 | Chloramphenicol Acetyltransferase CAT |
| H2 | Ga0110938_10196191 | Chloramphenicol Efflux Pump |
| H2 | Ga0110938_11493841 | Chloramphenicol Efflux Pump |
| H2 | Ga0110938_10943771 | Chloramphenicol Efflux Pump |
| H2 | Ga0110938_11990631 | Chloramphenicol Efflux Pump |
| H2 | Ga0110938_11026691 | Chloramphenicol Efflux Pump |
| H2 | Ga0110938_10454584 | cpxA |
| H2 | Ga0110938_10126491 | cpxA |
| H2 | Ga0110938_10281611 | cpxA |
| H2 | Ga0110938_12529761 | cpxA |
| H2 | Ga0110938_10835981 | cpxA |
| H2 | Ga0110938_10909811 | cpxA |
| H2 | Ga0110938_10397431 | cpxA |
| H2 | Ga0110938_10454583 | cpxR |
| H2 | Ga0110938_10126492 | cpxR |
| H2 | Ga0110938_10099691 | CRP |
| H2 | Ga0110938_10235201 | CRP |
| H2 | Ga0110938_10043613 | CRP |
| H2 | Ga0110938_10482591 | CRP |
| H2 | Ga0110938_10942731 | CRP |
| H2 | Ga0110938_11268391 | CRP |
| H2 | Ga0110938_11273191 | CRP |
| H2 | Ga0110938_10567841 | dfrA12 |
| H2 | Ga0110938_10016132 | dfrA14 |
| H2 | Ga0110938_11111441 | dfrA15 |
| H2 | Ga0110938_11929051 | dfrA16 |
| H2 | Ga0110938_10086021 | dfrF |
| H2 | Ga0110938_12112211 | emrA |
| H2 | Ga0110938_11707921 | emrA |
| H2 | Ga0110938_12112221 | emrA |
| H2 | Ga0110938_11203061 | emrA |
| H2 | Ga0110938_11325921 | emrB |
| H2 | Ga0110938_10811292 | emrB |
| H2 | Ga0110938_11064641 | emrB |
| H2 | Ga0110938_10842391 | emrB |
| H2 | Ga0110938_11971171 | emrB |
| H2 | Ga0110938_11064651 | emrB |
| H2 | Ga0110938_10974491 | emrB |
| H2 | Ga0110938_11553851 | emrD |
| H2 | Ga0110938_10383141 | emrK |
| H2 | Ga0110938_10586192 | emrR |
| H2 | Ga0110938_12034831 | emrR |
| H2 | Ga0110938_12314091 | emrR |
| H2 | Ga0110938_11886211 | emrY |
| H2 | Ga0110938_10603521 | ereA |
| H2 | Ga0110938_10293361 | ereA3 |
| H2 | Ga0110938_10603512 | ereA4 |
| H2 | Ga0110938_10603531 | ereA5 |
| H2 | Ga0110938_10508862 | ereB |
| H2 | Ga0110938_12325061 | Erm 23S ribosomal RNA Methyltransferase |
| H2 | Ga0110938_10306496 | ermB |
| H2 | Ga0110938_10204172 | ermF |
| H2 | Ga0110938_12097801 | ermX |
| H2 | Ga0110938_10198511 | evgA |
| H2 | Ga0110938_10863441 | evgS |
| H2 | Ga0110938_11930711 | evgS |
| H2 | Ga0110938_10303891 | evgS |
| H2 | Ga0110938_100229211 | Fluoroquinolone Resistant DNA Topoisomerase |
| H2 | Ga0110938_10447532 | Fluoroquinolone Resistant DNA Topoisomerase |
| H2 | Ga0110938_10130771 | Fluoroquinolone Resistant DNA Topoisomerase |
| H2 | Ga0110938_10708142 | Fluoroquinolone Resistant DNA Topoisomerase |
| H2 | Ga0110938_11237071 | Fluoroquinolone Resistant DNA Topoisomerase |
| H2 | Ga0110938_10884031 | Fluoroquinolone Resistant DNA Topoisomerase |
| H2 | Ga0110938_12060971 | Fluoroquinolone Resistant DNA Topoisomerase |
| H2 | Ga0110938_10067072 | Fluoroquinolone Resistant DNA Topoisomerase |
| H2 | Ga0110938_10837951 | Fluoroquinolone Resistant DNA Topoisomerase |
| H2 | Ga0110938_10883421 | Fluoroquinolone Resistant DNA Topoisomerase |
| H2 | Ga0110938_12218111 | Fluoroquinolone Resistant DNA Topoisomerase |
| H2 | Ga0110938_12881421 | Fluoroquinolone Resistant DNA Topoisomerase |
| H2 | Ga0110938_11964571 | Fluoroquinolone Resistant DNA Topoisomerase |
| H2 | Ga0110938_10936761 | Fluoroquinolone Resistant DNA Topoisomerase |
| H2 | Ga0110938_10882472 | Fluoroquinolone Resistant DNA Topoisomerase |
| H2 | Ga0110938_10067081 | Fluoroquinolone Resistant DNA Topoisomerase |
| H2 | Ga0110938_11003141 | Fluoroquinolone Resistant DNA Topoisomerase |
| H2 | Ga0110938_11270951 | Fluoroquinolone Resistant DNA Topoisomerase |
| H2 | Ga0110938_10108951 | Fluoroquinolone Resistant DNA Topoisomerase |
| H2 | Ga0110938_10231291 | Fluoroquinolone Resistant DNA Topoisomerase |
| H2 | Ga0110938_12598052 | Fluoroquinolone Resistant DNA Topoisomerase |
| H2 | Ga0110938_10161102 | Fluoroquinolone Resistant DNA Topoisomerase |
| H2 | Ga0110938_10702691 | Fluoroquinolone Resistant DNA Topoisomerase |
| H2 | Ga0110938_10002881 | Fluoroquinolone Resistant DNA Topoisomerase |
| H2 | Ga0110938_11715751 | Fluoroquinolone Resistant DNA Topoisomerase |
| H2 | Ga0110938_12671311 | Fluoroquinolone Resistant DNA Topoisomerase |
| H2 | Ga0110938_12397561 | Fluoroquinolone Resistant DNA Topoisomerase |
| H2 | Ga0110938_10582581 | Fluoroquinolone Resistant DNA Topoisomerase |
| H2 | Ga0110938_11741061 | Fluoroquinolone Resistant DNA Topoisomerase |
| H2 | Ga0110938_10650661 | Fluoroquinolone Resistant DNA Topoisomerase |
| H2 | Ga0110938_11213351 | Fluoroquinolone Resistant DNA Topoisomerase |
| H2 | Ga0110938_10702681 | Fluoroquinolone Resistant DNA Topoisomerase |
| H2 | Ga0110938_11649251 | Fluoroquinolone Resistant DNA Topoisomerase |
| H2 | Ga0110938_11858011 | Fluoroquinolone Resistant DNA Topoisomerase |
| H2 | Ga0110938_10983231 | Fluoroquinolone Resistant DNA Topoisomerase |
| H2 | Ga0110938_11650361 | Fluoroquinolone Resistant DNA Topoisomerase |
| H2 | Ga0110938_12406641 | Fluoroquinolone Resistant DNA Topoisomerase |
| H2 | Ga0110938_10143261 | Fluoroquinolone Resistant DNA Topoisomerase |
| H2 | Ga0110938_10920261 | Fluoroquinolone Resistant DNA Topoisomerase |
| H2 | Ga0110938_12415361 | Fluoroquinolone Resistant DNA Topoisomerase |
| H2 | Ga0110938_10702671 | Fluoroquinolone Resistant DNA Topoisomerase |
| H2 | Ga0110938_12509111 | Fluoroquinolone Resistant DNA Topoisomerase |
| H2 | Ga0110938_10055821 | Fluoroquinolone Resistant DNA Topoisomerase |
| H2 | Ga0110938_10161081 | Fluoroquinolone Resistant DNA Topoisomerase |
| H2 | Ga0110938_10276161 | Fluoroquinolone Resistant DNA Topoisomerase |
| H2 | Ga0110938_10390661 | Fluoroquinolone Resistant DNA Topoisomerase |
| H2 | Ga0110938_10606761 | Fluoroquinolone Resistant DNA Topoisomerase |
| H2 | Ga0110938_10618051 | Fluoroquinolone Resistant DNA Topoisomerase |
| H2 | Ga0110938_10618061 | Fluoroquinolone Resistant DNA Topoisomerase |
| H2 | Ga0110938_10713631 | Fluoroquinolone Resistant DNA Topoisomerase |
| H2 | Ga0110938_11222931 | Fluoroquinolone Resistant DNA Topoisomerase |
| H2 | Ga0110938_11468961 | Fluoroquinolone Resistant DNA Topoisomerase |
| H2 | Ga0110938_11597561 | Fluoroquinolone Resistant DNA Topoisomerase |
| H2 | Ga0110938_11649241 | Fluoroquinolone Resistant DNA Topoisomerase |
| H2 | Ga0110938_11721071 | Fluoroquinolone Resistant DNA Topoisomerase |
| H2 | Ga0110938_11586182 | fosA2 |
| H2 | Ga0110938_11143072 | gadX |
| H2 | Ga0110938_10604851 | gadX |
| H2 | Ga0110938_10028951 | *bla*_GES-1_ |
| H2 | Ga0110938_11946251 | golS |
| H2 | Ga0110938_11712971 | H-NS |
| H2 | Ga0110938_10443821 | *bla*_IMP-42_ |
| H2 | Ga0110938_10443811 | *bla*_IMP-42_ |
| H2 | Ga0110938_11780382 | *bla*_IMP-6_ |
| H2 | Ga0110938_10642051 | linB |
| H2 | Ga0110938_12694501 | linB |
| H2 | Ga0110938_10005824 | lnuC |
| H2 | Ga0110938_10521822 | lnuD |
| H2 | Ga0110938_11024171 | macA |
| H2 | Ga0110938_10258441 | macA |
| H2 | Ga0110938_11465432 | macB |
| H2 | Ga0110938_11246811 | macB |
| H2 | Ga0110938_12620812 | macB |
| H2 | Ga0110938_13030481 | macB |
| H2 | Ga0110938_12625111 | macB |
| H2 | Ga0110938_12810611 | macB |
| H2 | Ga0110938_12754451 | marA |
| H2 | Ga0110938_11516891 | mdfA |
| H2 | Ga0110938_12156151 | mdfA |
| H2 | Ga0110938_12266371 | mdfA |
| H2 | Ga0110938_10478962 | mdsB |
| H2 | Ga0110938_12934551 | mdsB |
| H2 | Ga0110938_10750501 | mdtA |
| H2 | Ga0110938_11397531 | mdtA |
| H2 | Ga0110938_11439241 | mdtA |
| H2 | Ga0110938_10125131 | mdtB |
| H2 | Ga0110938_10282461 | mdtB |
| H2 | Ga0110938_10675151 | mdtB |
| H2 | Ga0110938_10750011 | mdtB |
| H2 | Ga0110938_11817641 | mdtB |
| H2 | Ga0110938_10125122 | mdtB |
| H2 | Ga0110938_12034721 | mdtB |
| H2 | Ga0110938_12041581 | mdtB |
| H2 | Ga0110938_10110221 | mdtC |
| H2 | Ga0110938_11461582 | mdtC |
| H2 | Ga0110938_10998391 | mdtC |
| H2 | Ga0110938_10125121 | mdtC |
| H2 | Ga0110938_11077501 | mdtC |
| H2 | Ga0110938_10127464 | mdtC |
| H2 | Ga0110938_11065001 | mdtC |
| H2 | Ga0110938_11170441 | mdtC |
| H2 | Ga0110938_12194161 | mdtC |
| H2 | Ga0110938_10127463 | mdtD |
| H2 | Ga0110938_10127462 | mdtD |
| H2 | Ga0110938_12684071 | mdtD |
| H2 | Ga0110938_11203181 | mdtE |
| H2 | Ga0110938_12541351 | mdtE |
| H2 | Ga0110938_11556861 | mdtF |
| H2 | Ga0110938_10185311 | mdtF |
| H2 | Ga0110938_11717921 | mdtF |
| H2 | Ga0110938_11019281 | mdtF |
| H2 | Ga0110938_11576501 | mdtG |
| H2 | Ga0110938_12142761 | mdtG |
| H2 | Ga0110938_10927551 | mdtH |
| H2 | Ga0110938_10221072 | mdtH |
| H2 | Ga0110938_12687581 | mdtK |
| H2 | Ga0110938_12687571 | mdtK |
| H2 | Ga0110938_12408172 | mdtK |
| H2 | Ga0110938_11884111 | mdtK |
| H2 | Ga0110938_10040572 | mdtK |
| H2 | Ga0110938_12219721 | mdtK |
| H2 | Ga0110938_11715211 | mdtK |
| H2 | Ga0110938_12606562 | mdtK |
| H2 | Ga0110938_11064681 | mdtL |
| H2 | Ga0110938_11223092 | mdtL |
| H2 | Ga0110938_11845381 | mdtM |
| H2 | Ga0110938_11587721 | mdtN |
| H2 | Ga0110938_10657401 | mdtO |
| H2 | Ga0110938_12527991 | mdtO |
| H2 | Ga0110938_10328721 | mdtP |
| H2 | Ga0110938_11587701 | mdtP |
| H2 | Ga0110938_10442321 | mefA |
| H2 | Ga0110938_11790621 | mexC |
| H2 | Ga0110938_10106061 | mexD |
| H2 | Ga0110938_10268741 | mexD |
| H2 | Ga0110938_12447511 | mexD |
| H2 | Ga0110938_11733711 | mexF |
| H2 | Ga0110938_10397101 | mexL |
| H2 | Ga0110938_10636972 | mexQ |
| H2 | Ga0110938_12669261 | mexT |
| H2 | Ga0110938_12294182 | mexT |
| H2 | Ga0110938_12366331 | mexT |
| H2 | Ga0110938_10249121 | mexY |
| H2 | Ga0110938_10923061 | MFS Antibiotic Efflux Pump |
| H2 | Ga0110938_10712701 | MFS Antibiotic Efflux Pump |
| H2 | Ga0110938_10038012 | mphA |
| H2 | Ga0110938_12260342 | msbA |
| H2 | Ga0110938_10734871 | msbA |
| H2 | Ga0110938_11663331 | msbA |
| H2 | Ga0110938_12592691 | msbA |
| H2 | Ga0110938_10039081 | msrE |
| H2 | Ga0110938_12446082 | mtrD |
| H2 | Ga0110938_10736382 | *bla*_NDM-2_ |
| H2 | Ga0110938_10284301 | oprN |
| H2 | Ga0110938_10020431 | *bla*_OXA-1_ |
| H2 | Ga0110938_10020444 | *bla*_OXA-1_ |
| H2 | Ga0110938_10006001 | *bla*_OXA-10_ |
| H2 | Ga0110938_10084272 | *bla*_OXA-12_ |
| H2 | Ga0110938_10084121 | *bla*_OXA-12_ |
| H2 | Ga0110938_12891081 | *bla*_OXA-334_ |
| H2 | Ga0110938_10204171 | *bla*_OXA-347_ |
| H2 | Ga0110938_10056971 | *bla*_OXA-58_ |
| H2 | Ga0110938_10104561 | phoP |
| H2 | Ga0110938_11638511 | phoP |
| H2 | Ga0110938_10604331 | phoQ |
| H2 | Ga0110938_12700742 | phoQ |
| H2 | Ga0110938_11289741 | phoQ |
| H2 | Ga0110938_11289751 | phoQ |
| H2 | Ga0110938_10945782 | PmrA |
| H2 | Ga0110938_11796992 | PmrA |
| H2 | Ga0110938_12356462 | PmrA |
| H2 | Ga0110938_12274741 | PmrB |
| H2 | Ga0110938_10799982 | PmrB |
| H2 | Ga0110938_12356461 | PmrC |
| H2 | Ga0110938_11175122 | PmrC |
| H2 | Ga0110938_11093461 | PmrC |
| H2 | Ga0110938_11796991 | PmrC |
| H2 | Ga0110938_10194161 | PmrE |
| H2 | Ga0110938_10194091 | PmrE |
| H2 | Ga0110938_11741651 | PmrE |
| H2 | Ga0110938_11335162 | PmrF |
| H2 | Ga0110938_11180461 | PmrF |
| H2 | Ga0110938_11180591 | PmrF |
| H2 | Ga0110938_10930572 | QnrB6 |
| H2 | Ga0110938_10187781 | QnrS2 |
| H2 | Ga0110938_10565071 | QnrS7 |
| H2 | Ga0110938_10754491 | QnrS8 |
| H2 | Ga0110938_10126074 | QnrVC4 |
| H2 | Ga0110938_11384952 | ramA |
| H2 | Ga0110938_10898691 | RND Antibiotic Efflux Pump |
| H2 | Ga0110938_11721482 | RND Antibiotic Efflux Pump |
| H2 | Ga0110938_10698971 | RND Antibiotic Efflux Pump |
| H2 | Ga0110938_11266391 | RND Antibiotic Efflux Pump |
| H2 | Ga0110938_10940971 | RND Antibiotic Efflux Pump |
| H2 | Ga0110938_10842162 | RND Antibiotic Efflux Pump |
| H2 | Ga0110938_10238601 | RND Antibiotic Efflux Pump |
| H2 | Ga0110938_12496591 | RND Antibiotic Efflux Pump |
| H2 | Ga0110938_10460632 | RND Antibiotic Efflux Pump |
| H2 | Ga0110938_10518541 | RND Antibiotic Efflux Pump |
| H2 | Ga0110938_12579331 | RND Antibiotic Efflux Pump |
| H2 | Ga0110938_12031511 | RND Antibiotic Efflux Pump |
| H2 | Ga0110938_10157901 | RND Antibiotic Efflux Pump |
| H2 | Ga0110938_10212381 | RND Antibiotic Efflux Pump |
| H2 | Ga0110938_10442521 | RND Antibiotic Efflux Pump |
| H2 | Ga0110938_10759531 | RND Antibiotic Efflux Pump |
| H2 | Ga0110938_10912691 | RND Antibiotic Efflux Pump |
| H2 | Ga0110938_11414171 | RND Antibiotic Efflux Pump |
| H2 | Ga0110938_10380091 | robA |
| H2 | Ga0110938_10488153 | robA |
| H2 | Ga0110938_10610411 | robA |
| H2 | Ga0110938_11000842 | romA |
| H2 | Ga0110938_10797111 | romA |
| H2 | Ga0110938_11384951 | romA |
| H2 | Ga0110938_12608522 | rosA |
| H2 | Ga0110938_10205451 | rosB |
| H2 | Ga0110938_13101111 | rosB |
| H2 | Ga0110938_12499341 | rosB |
| H2 | Ga0110938_12163381 | rosB |
| H2 | Ga0110938_10720962 | sat-4 |
| H2 | Ga0110938_11540761 | *bla*_SHV-123_ |
| H2 | Ga0110938_10386291 | soxR |
| H2 | Ga0110938_11146851 | soxR |
| H2 | Ga0110938_10004441 | sul1 |
| H2 | Ga0110938_100372421 | sul2 |
| H2 | Ga0110938_10167571 | *bla*_TEM_ |
| H2 | Ga0110938_10309301 | tet40 |
| H2 | Ga0110938_10011549 | tetO |
| H2 | Ga0110938_10042996 | Tetracycline Resistance MFS Efflux Pump |
| H2 | Ga0110938_10203652 | Tetracycline Resistance MFS Efflux Pump |
| H2 | Ga0110938_11445541 | Tetracycline Resistance MFS Efflux Pump |
| H2 | Ga0110938_10134972 | Tetracycline Resistance MFS Efflux Pump |
| H2 | Ga0110938_11390431 | Tetracycline Resistance MFS Efflux Pump |
| H2 | Ga0110938_12963321 | Tetracycline Resistance MFS Efflux Pump |
| H2 | Ga0110938_10083622 | Tetracycline Resistance MFS Efflux Pump |
| H2 | Ga0110938_11656001 | Tetracycline Resistance MFS Efflux Pump |
| H2 | Ga0110938_12207831 | Tetracycline Resistance MFS Efflux Pump |
| H2 | Ga0110938_13089301 | Tetracycline Resistance MFS Efflux Pump |
| H2 | Ga0110938_10061528 | Tetracycline Resistance Ribosomal Protection Protein |
| H2 | Ga0110938_10484694 | Tetracycline Resistance Ribosomal Protection Protein |
| H2 | Ga0110938_10105261 | Tetracycline Resistance Ribosomal Protection Protein |
| H2 | Ga0110938_10127012 | Tetracycline Resistance Ribosomal Protection Protein |
| H2 | Ga0110938_10584841 | Tetracycline Resistance Ribosomal Protection Protein |
| H2 | Ga0110938_10727401 | Tetracycline Resistance Ribosomal Protection Protein |
| H2 | Ga0110938_11002031 | Tetracycline Resistance Ribosomal Protection Protein |
| H2 | Ga0110938_12131341 | Tetracycline Resistance Ribosomal Protection Protein |
| H2 | Ga0110938_10909941 | Tetracycline Resistance Ribosomal Protection Protein |
| H2 | Ga0110938_10397771 | Tetracycline Resistance Ribosomal Protection Protein |
| H2 | Ga0110938_10655771 | Tetracycline Resistance Ribosomal Protection Protein |
| H2 | Ga0110938_12587861 | Tetracycline Resistance Ribosomal Protection Protein |
| H2 | Ga0110938_13014481 | Tetracycline Resistance Ribosomal Protection Protein |
| H2 | Ga0110938_11618601 | Tetracycline Resistance Ribosomal Protection Protein |
| H2 | Ga0110938_11002021 | Tetracycline Resistance Ribosomal Protection Protein |
| H2 | Ga0110938_11002041 | Tetracycline Resistance Ribosomal Protection Protein |
| H2 | Ga0110938_11110731 | Tetracycline Resistance Ribosomal Protection Protein |
| H2 | Ga0110938_13002001 | Tetracycline Resistance Ribosomal Protection Protein |
| H2 | Ga0110938_10065811 | tetX |
| H2 | Ga0110938_12852471 | tolC |
| H2 | Ga0110938_10624081 | tolC |
| H2 | Ga0110938_10195831 | tolC |
| H2 | Ga0110938_12422891 | tolC |
| H2 | Ga0110938_11898011 | tolC |
| H2 | Ga0110938_12267461 | tolC |
| H2 | Ga0110938_12171631 | vanH |
| H2 | Ga0110938_12204561 | vanH |
| H2 | Ga0110938_12919052 | vanR |
| H2 | Ga0110938_11051711 | vanR |
| H2 | Ga0110938_11051712 | vanS |
| H2 | Ga0110938_12609631 | vanS |
| H2 | Ga0110938_11310331 | *bla*_VEB-3_ |
| H3 | Ga0110939_12921312 | AAC(2')-Ib |
| H3 | Ga0110939_10044571 | AAC(6')-Ie-APH(2'')-Ia |
| H3 | Ga0110939_10255571 | AAC(6')-Ie-APH(2'')-Ia |
| H3 | Ga0110939_10732871 | AAC(6')-Ie-APH(2'')-Ia |
| H3 | Ga0110939_10732881 | AAC(6')-Ie-APH(2'')-Ia |
| H3 | Ga0110939_12503691 | AAC3-Ia |
| H3 | Ga0110939_10043342 | aac3/AAC(3)-Iia |
| H3 | Ga0110939_10009191 | AAC6-Ib |
| H3 | Ga0110939_11115731 | AAC6-II |
| H3 | Ga0110939_13086421 | AAC6-II |
| H3 | Ga0110939_10332681 | aad(6) |
| H3 | Ga0110939_10020671 | aadA16 |
| H3 | Ga0110939_12192081 | aadA8 |
| H3 | Ga0110939_10228122 | ABC Antibiotic Efflux Pump |
| H3 | Ga0110939_10749752 | ABC Antibiotic Efflux Pump |
| H3 | Ga0110939_10069663 | abeM |
| H3 | Ga0110939_10738951 | abeM |
| H3 | Ga0110939_12595052 | abeM |
| H3 | Ga0110939_100003540 | acrA |
| H3 | Ga0110939_10403061 | acrA |
| H3 | Ga0110939_10403071 | acrA |
| H3 | Ga0110939_11766051 | acrA |
| H3 | Ga0110939_12199681 | acrA |
| H3 | Ga0110939_100003541 | acrB |
| H3 | Ga0110939_10840711 | acrB |
| H3 | Ga0110939_11196261 | acrB |
| H3 | Ga0110939_100009294 | acrD |
| H3 | Ga0110939_10707991 | acrD |
| H3 | Ga0110939_10863321 | acrD |
| H3 | Ga0110939_11113371 | acrD |
| H3 | Ga0110939_12174471 | acrD |
| H3 | Ga0110939_12558291 | acrD |
| H3 | Ga0110939_1000235148 | acrE |
| H3 | Ga0110939_12021081 | acrE |
| H3 | Ga0110939_1000235149 | acrF |
| H3 | Ga0110939_10240761 | acrF |
| H3 | Ga0110939_10240771 | acrF |
| H3 | Ga0110939_11461211 | acrF |
| H3 | Ga0110939_11625041 | acrF |
| H3 | Ga0110939_13087511 | acrF |
| H3 | Ga0110939_1000235147 | acrS |
| H3 | Ga0110939_11642841 | adeA-adeI |
| H3 | Ga0110939_12555071 | adeA-adeI |
| H3 | Ga0110939_10120063 | adeB |
| H3 | Ga0110939_10376471 | adeB |
| H3 | Ga0110939_11033341 | adeB |
| H3 | Ga0110939_11231011 | adeB |
| H3 | Ga0110939_11307961 | adeB |
| H3 | Ga0110939_11310681 | adeB |
| H3 | Ga0110939_11361851 | adeB |
| H3 | Ga0110939_11408481 | adeB |
| H3 | Ga0110939_12001491 | adeB |
| H3 | Ga0110939_12709201 | adeB |
| H3 | Ga0110939_12729931 | adeB |
| H3 | Ga0110939_12982291 | adeB |
| H3 | Ga0110939_12984941 | adeB |
| H3 | Ga0110939_13112372 | adeB |
| H3 | Ga0110939_11682862 | adeC |
| H3 | Ga0110939_13264041 | adeC |
| H3 | Ga0110939_10457231 | adeC-adeK-oprM |
| H3 | Ga0110939_11129281 | adeC-adeK-oprM |
| H3 | Ga0110939_13151001 | adeC-adeK-oprM |
| H3 | Ga0110939_12148051 | adeF |
| H3 | Ga0110939_12148052 | adeF |
| H3 | Ga0110939_12363951 | adeG |
| H3 | Ga0110939_13083911 | adeJ |
| H3 | Ga0110939_10414262 | adeN |
| H3 | Ga0110939_11060581 | adeR |
| H3 | Ga0110939_10953011 | ampC |
| H3 | Ga0110939_10383491 | ANT(2'')-Ia |
| H3 | Ga0110939_10189063 | ANT(6)-Ia |
| H3 | Ga0110939_12606111 | ANT(9)-Ia |
| H3 | Ga0110939_10093411 | ANT3 |
| H3 | Ga0110939_10112001 | ANT3 |
| H3 | Ga0110939_10285571 | ANT3 |
| H3 | Ga0110939_10132477 | ANT6 |
| H3 | Ga0110939_10141741 | ANT6 |
| H3 | Ga0110939_10687671 | ANT6 |
| H3 | Ga0110939_13288561 | APH(2'')-IIa |
| H3 | Ga0110939_10148994 | APH3 |
| H3 | Ga0110939_10392341 | APH3 |
| H3 | Ga0110939_11037301 | APH3 |
| H3 | Ga0110939_11056061 | APH3 |
| H3 | Ga0110939_1000008147 | APH3" |
| H3 | Ga0110939_10060551 | APH3" |
| H3 | Ga0110939_10092653 | APH3" |
| H3 | Ga0110939_10168143 | APH3" |
| H3 | Ga0110939_10304001 | APH3" |
| H3 | Ga0110939_10325112 | APH3" |
| H3 | Ga0110939_10377451 | APH3" |
| H3 | Ga0110939_10665962 | APH3" |
| H3 | Ga0110939_10975272 | APH3" |
| H3 | Ga0110939_11052921 | APH3" |
| H3 | Ga0110939_11200361 | APH3" |
| H3 | Ga0110939_11299612 | APH3" |
| H3 | Ga0110939_11678241 | APH3" |
| H3 | Ga0110939_11940911 | APH3" |
| H3 | Ga0110939_10141743 | APH3' |
| H3 | Ga0110939_10176702 | APH3' |
| H3 | Ga0110939_10392342 | APH3' |
| H3 | Ga0110939_10092652 | APH6 |
| H3 | Ga0110939_10011471 | armA |
| H3 | Ga0110939_10011472 | armA |
| H3 | Ga0110939_10181661 | armA |
| H3 | Ga0110939_100116960 | arnA |
| H3 | Ga0110939_11723212 | arnA |
| H3 | Ga0110939_10173762 | arr-2 |
| H3 | Ga0110939_10020673 | arr-3 |
| H3 | Ga0110939_10020681 | arr-3 |
| H3 | Ga0110939_100014552 | bacA |
| H3 | Ga0110939_10168451 | bacA |
| H3 | Ga0110939_11119332 | bacA |
| H3 | Ga0110939_11390071 | bacA |
| H3 | Ga0110939_100024283 | baeR |
| H3 | Ga0110939_10679331 | baeR |
| H3 | Ga0110939_10680181 | baeR |
| H3 | Ga0110939_11442711 | baeR |
| H3 | Ga0110939_11723801 | baeR |
| H3 | Ga0110939_12492562 | baeR |
| H3 | Ga0110939_12571191 | baeR |
| H3 | Ga0110939_12947233 | baeR |
| H3 | Ga0110939_100024282 | baeS |
| H3 | Ga0110939_10422691 | baeS |
| H3 | Ga0110939_10801401 | baeS |
| H3 | Ga0110939_10801411 | baeS |
| H3 | Ga0110939_12200131 | baeS |
| H3 | Ga0110939_12238181 | baeS |
| H3 | Ga0110939_12478471 | baeS |
| H3 | Ga0110939_12481331 | baeS |
| H3 | Ga0110939_12492561 | baeS |
| H3 | Ga0110939_13265231 | baeS |
| H3 | Ga0110939_10004641 | Beta-lactamase Class A |
| H3 | Ga0110939_100089318 | Beta-lactamase Class A |
| H3 | Ga0110939_10497541 | Beta-lactamase Class A |
| H3 | Ga0110939_11513623 | Beta-lactamase Class A |
| H3 | Ga0110939_12636491 | Beta-lactamase Class A |
| H3 | Ga0110939_10084301 | *bla*_CTX-M3_ |
| H3 | Ga0110939_12192082 | *bla*_CARB-7_ |
| H3 | Ga0110939_11797311 | *bla*_DHA-7_ |
| H3 | Ga0110939_10028071 | *bla*_IMP-1_ |
| H3 | Ga0110939_10933941 | *bla*_NDM-2_ |
| H3 | Ga0110939_10643791 | *bla*_SHV-12_ |
| H3 | Ga0110939_10542241 | *bla*_SHV-28_ |
| H3 | Ga0110939_11244331 | *bla*_VIM-18_ |
| H3 | Ga0110939_10774021 | *bla*_VIM-26_ |
| H3 | Ga0110939_10774032 | *bla*_VIM-30_ |
| H3 | Ga0110939_10000441 | bleomycin resistance protein (BRP) |
| H3 | Ga0110939_10933952 | bleomycin resistance protein (BRP) |
| H3 | Ga0110939_10580442 | cat |
| H3 | Ga0110939_10913011 | catS |
| H3 | Ga0110939_10886651 | cblA |
| H3 | Ga0110939_12270912 | cblA |
| H3 | Ga0110939_10103351 | ceoB |
| H3 | Ga0110939_11291912 | ceoB |
| H3 | Ga0110939_10199471 | Cfr 23 Ribosomal RNA Methyltransferase |
| H3 | Ga0110939_11042651 | Cfr 23 Ribosomal RNA Methyltransferase |
| H3 | Ga0110939_11778781 | Cfr 23 Ribosomal RNA Methyltransferase |
| H3 | Ga0110939_12137962 | Cfr 23 Ribosomal RNA Methyltransferase |
| H3 | Ga0110939_12744861 | Cfr 23 Ribosomal RNA Methyltransferase |
| H3 | Ga0110939_12865851 | Cfr 23 Ribosomal RNA Methyltransferase |
| H3 | Ga0110939_10050473 | cfxA6 |
| H3 | Ga0110939_11290671 | cfxA6 |
| H3 | Ga0110939_10014432 | Chloramphenicol Acetyltransferase CAT |
| H3 | Ga0110939_10014441 | Chloramphenicol Acetyltransferase CAT |
| H3 | Ga0110939_10024341 | Chloramphenicol Acetyltransferase CAT |
| H3 | Ga0110939_10024351 | Chloramphenicol Acetyltransferase CAT |
| H3 | Ga0110939_10486681 | Chloramphenicol Acetyltransferase CAT |
| H3 | Ga0110939_11826811 | Chloramphenicol Acetyltransferase CAT |
| H3 | Ga0110939_11826821 | Chloramphenicol Acetyltransferase CAT |
| H3 | Ga0110939_12205712 | Chloramphenicol Acetyltransferase CAT |
| H3 | Ga0110939_12752671 | Chloramphenicol Acetyltransferase CAT |
| H3 | Ga0110939_12926991 | Chloramphenicol Acetyltransferase CAT |
| H3 | Ga0110939_13081801 | Chloramphenicol Acetyltransferase CAT |
| H3 | Ga0110939_10149631 | Chloramphenicol Efflux Pump |
| H3 | Ga0110939_10173761 | Chloramphenicol Efflux Pump |
| H3 | Ga0110939_10456911 | Chloramphenicol Efflux Pump |
| H3 | Ga0110939_10456922 | Chloramphenicol Efflux Pump |
| H3 | Ga0110939_10457081 | Chloramphenicol Efflux Pump |
| H3 | Ga0110939_10457091 | Chloramphenicol Efflux Pump |
| H3 | Ga0110939_11599611 | Chloramphenicol Efflux Pump |
| H3 | Ga0110939_11762931 | Chloramphenicol Efflux Pump |
| H3 | Ga0110939_11762941 | Chloramphenicol Efflux Pump |
| H3 | Ga0110939_12003401 | Chloramphenicol Efflux Pump |
| H3 | Ga0110939_12620481 | Chloramphenicol Efflux Pump |
| H3 | Ga0110939_12890591 | Chloramphenicol Efflux Pump |
| H3 | Ga0110939_100007835 | ClassC-AmpC |
| H3 | Ga0110939_10252181 | ClassC-AmpC |
| H3 | Ga0110939_11484141 | ClassC-AmpC |
| H3 | Ga0110939_11402691 | cmlB1 |
| H3 | Ga0110939_10953002 | *bla*_CMY-78_ |
| H3 | Ga0110939_1001090101 | cpxA |
| H3 | Ga0110939_10294071 | cpxA |
| H3 | Ga0110939_11671131 | cpxA |
| H3 | Ga0110939_12336591 | cpxA |
| H3 | Ga0110939_12612171 | cpxA |
| H3 | Ga0110939_1001090100 | cpxR |
| H3 | Ga0110939_10525951 | cpxR |
| H3 | Ga0110939_1000008131 | CRP |
| H3 | Ga0110939_10167191 | CRP |
| H3 | Ga0110939_10170822 | CRP |
| H3 | Ga0110939_10739001 | CRP |
| H3 | Ga0110939_12823381 | CRP |
| H3 | Ga0110939_11092961 | *bla*_CTX-M-147_ |
| H3 | Ga0110939_12323781 | dfrA12 |
| H3 | Ga0110939_11462051 | dfrA14 |
| H3 | Ga0110939_10093412 | dfrA17 |
| H3 | Ga0110939_11968821 | dfrB1 |
| H3 | Ga0110939_10312001 | dfrF |
| H3 | Ga0110939_10371751 | dfrG |
| H3 | Ga0110939_12043941 | dfrG |
| H3 | Ga0110939_10000839 | emrA |
| H3 | Ga0110939_10140482 | emrA |
| H3 | Ga0110939_10183931 | emrA |
| H3 | Ga0110939_10000838 | emrB |
| H3 | Ga0110939_10949731 | emrB |
| H3 | Ga0110939_11072931 | emrB |
| H3 | Ga0110939_11139321 | emrB |
| H3 | Ga0110939_12022131 | emrB |
| H3 | Ga0110939_12562751 | emrB |
| H3 | Ga0110939_12666191 | emrB |
| H3 | Ga0110939_10961901 | emrD |
| H3 | Ga0110939_11952391 | emrD |
| H3 | Ga0110939_100005536 | emrE |
| H3 | Ga0110939_100008310 | emrR |
| H3 | Ga0110939_10140481 | emrR |
| H3 | Ga0110939_11684311 | emrR |
| H3 | Ga0110939_10000922 | emrY |
| H3 | Ga0110939_10096732 | ereA |
| H3 | Ga0110939_10168751 | ereA6 |
| H3 | Ga0110939_10486671 | ereB |
| H3 | Ga0110939_10529541 | ereB |
| H3 | Ga0110939_11245301 | ereB |
| H3 | Ga0110939_10044573 | Erm 23S ribosomal RNA Methyltransferase |
| H3 | Ga0110939_12416842 | Erm 23S ribosomal RNA Methyltransferase |
| H3 | Ga0110939_11015591 | ermB |
| H3 | Ga0110939_100139313 | ermF |
| H3 | Ga0110939_10000924 | evgA |
| H3 | Ga0110939_10000925 | evgS |
| H3 | Ga0110939_100014598 | Fluoroquinolone Resistant DNA Topoisomerase |
| H3 | Ga0110939_100116936 | Fluoroquinolone Resistant DNA Topoisomerase |
| H3 | Ga0110939_10024271 | Fluoroquinolone Resistant DNA Topoisomerase |
| H3 | Ga0110939_10111651 | Fluoroquinolone Resistant DNA Topoisomerase |
| H3 | Ga0110939_10140724 | Fluoroquinolone Resistant DNA Topoisomerase |
| H3 | Ga0110939_10152612 | Fluoroquinolone Resistant DNA Topoisomerase |
| H3 | Ga0110939_10157102 | Fluoroquinolone Resistant DNA Topoisomerase |
| H3 | Ga0110939_10157955 | Fluoroquinolone Resistant DNA Topoisomerase |
| H3 | Ga0110939_10177901 | Fluoroquinolone Resistant DNA Topoisomerase |
| H3 | Ga0110939_10179441 | Fluoroquinolone Resistant DNA Topoisomerase |
| H3 | Ga0110939_10179451 | Fluoroquinolone Resistant DNA Topoisomerase |
| H3 | Ga0110939_10213421 | Fluoroquinolone Resistant DNA Topoisomerase |
| H3 | Ga0110939_10214211 | Fluoroquinolone Resistant DNA Topoisomerase |
| H3 | Ga0110939_10216911 | Fluoroquinolone Resistant DNA Topoisomerase |
| H3 | Ga0110939_10275861 | Fluoroquinolone Resistant DNA Topoisomerase |
| H3 | Ga0110939_10289471 | Fluoroquinolone Resistant DNA Topoisomerase |
| H3 | Ga0110939_10330561 | Fluoroquinolone Resistant DNA Topoisomerase |
| H3 | Ga0110939_10349811 | Fluoroquinolone Resistant DNA Topoisomerase |
| H3 | Ga0110939_10387471 | Fluoroquinolone Resistant DNA Topoisomerase |
| H3 | Ga0110939_10438031 | Fluoroquinolone Resistant DNA Topoisomerase |
| H3 | Ga0110939_10466191 | Fluoroquinolone Resistant DNA Topoisomerase |
| H3 | Ga0110939_10559731 | Fluoroquinolone Resistant DNA Topoisomerase |
| H3 | Ga0110939_10559732 | Fluoroquinolone Resistant DNA Topoisomerase |
| H3 | Ga0110939_10577831 | Fluoroquinolone Resistant DNA Topoisomerase |
| H3 | Ga0110939_10599921 | Fluoroquinolone Resistant DNA Topoisomerase |
| H3 | Ga0110939_10659341 | Fluoroquinolone Resistant DNA Topoisomerase |
| H3 | Ga0110939_10673391 | Fluoroquinolone Resistant DNA Topoisomerase |
| H3 | Ga0110939_10673401 | Fluoroquinolone Resistant DNA Topoisomerase |
| H3 | Ga0110939_10676581 | Fluoroquinolone Resistant DNA Topoisomerase |
| H3 | Ga0110939_10734371 | Fluoroquinolone Resistant DNA Topoisomerase |
| H3 | Ga0110939_10734381 | Fluoroquinolone Resistant DNA Topoisomerase |
| H3 | Ga0110939_10947171 | Fluoroquinolone Resistant DNA Topoisomerase |
| H3 | Ga0110939_10962531 | Fluoroquinolone Resistant DNA Topoisomerase |
| H3 | Ga0110939_10968871 | Fluoroquinolone Resistant DNA Topoisomerase |
| H3 | Ga0110939_11072992 | Fluoroquinolone Resistant DNA Topoisomerase |
| H3 | Ga0110939_11133001 | Fluoroquinolone Resistant DNA Topoisomerase |
| H3 | Ga0110939_11208431 | Fluoroquinolone Resistant DNA Topoisomerase |
| H3 | Ga0110939_11306611 | Fluoroquinolone Resistant DNA Topoisomerase |
| H3 | Ga0110939_11359121 | Fluoroquinolone Resistant DNA Topoisomerase |
| H3 | Ga0110939_11438381 | Fluoroquinolone Resistant DNA Topoisomerase |
| H3 | Ga0110939_11449241 | Fluoroquinolone Resistant DNA Topoisomerase |
| H3 | Ga0110939_11800801 | Fluoroquinolone Resistant DNA Topoisomerase |
| H3 | Ga0110939_11917852 | Fluoroquinolone Resistant DNA Topoisomerase |
| H3 | Ga0110939_12009871 | Fluoroquinolone Resistant DNA Topoisomerase |
| H3 | Ga0110939_12298251 | Fluoroquinolone Resistant DNA Topoisomerase |
| H3 | Ga0110939_12353541 | Fluoroquinolone Resistant DNA Topoisomerase |
| H3 | Ga0110939_12392401 | Fluoroquinolone Resistant DNA Topoisomerase |
| H3 | Ga0110939_12441821 | Fluoroquinolone Resistant DNA Topoisomerase |
| H3 | Ga0110939_12555181 | Fluoroquinolone Resistant DNA Topoisomerase |
| H3 | Ga0110939_12704491 | Fluoroquinolone Resistant DNA Topoisomerase |
| H3 | Ga0110939_12740031 | Fluoroquinolone Resistant DNA Topoisomerase |
| H3 | Ga0110939_12747361 | Fluoroquinolone Resistant DNA Topoisomerase |
| H3 | Ga0110939_12775021 | Fluoroquinolone Resistant DNA Topoisomerase |
| H3 | Ga0110939_12802721 | Fluoroquinolone Resistant DNA Topoisomerase |
| H3 | Ga0110939_13048081 | Fluoroquinolone Resistant DNA Topoisomerase |
| H3 | Ga0110939_13293091 | Fluoroquinolone Resistant DNA Topoisomerase |
| H3 | Ga0110939_100066435 | gadX |
| H3 | Ga0110939_10009481 | *bla*_GES-1_ |
| H3 | Ga0110939_100120826 | H-NS |
| H3 | Ga0110939_11020031 | H-NS |
| H3 | Ga0110939_11823412 | H-NS |
| H3 | Ga0110939_11907312 | H-NS |
| H3 | Ga0110939_13013571 | *bla*_LCR-1_ |
| H3 | Ga0110939_11298682 | lmrD |
| H3 | Ga0110939_10198308 | lnuC |
| H3 | Ga0110939_10215001 | lnuC |
| H3 | Ga0110939_13088831 | lsaA |
| H3 | Ga0110939_12292551 | lsaC |
| H3 | Ga0110939_100070929 | macA |
| H3 | Ga0110939_11288411 | macA |
| H3 | Ga0110939_11677291 | macA |
| H3 | Ga0110939_12430101 | macA |
| H3 | Ga0110939_10372061 | macB |
| H3 | Ga0110939_11216301 | macB |
| H3 | Ga0110939_11792001 | macB |
| H3 | Ga0110939_11975341 | macB |
| H3 | Ga0110939_12014241 | macB |
| H3 | Ga0110939_12661281 | macB |
| H3 | Ga0110939_12792371 | macB |
| H3 | Ga0110939_100297611 | marA |
| H3 | Ga0110939_10809804 | marA |
| H3 | Ga0110939_100070965 | mdfA |
| H3 | Ga0110939_10249701 | mdfA |
| H3 | Ga0110939_11750152 | mdsB |
| H3 | Ga0110939_100024278 | mdtA |
| H3 | Ga0110939_10716312 | mdtA |
| H3 | Ga0110939_10716321 | mdtA |
| H3 | Ga0110939_11261251 | mdtA |
| H3 | Ga0110939_12082401 | mdtA |
| H3 | Ga0110939_100024279 | mdtB |
| H3 | Ga0110939_10132802 | mdtB |
| H3 | Ga0110939_10387662 | mdtB |
| H3 | Ga0110939_10489021 | mdtB |
| H3 | Ga0110939_10869451 | mdtB |
| H3 | Ga0110939_10880821 | mdtB |
| H3 | Ga0110939_11218481 | mdtB |
| H3 | Ga0110939_11377491 | mdtB |
| H3 | Ga0110939_11438152 | mdtB |
| H3 | Ga0110939_12170961 | mdtB |
| H3 | Ga0110939_100024280 | mdtC |
| H3 | Ga0110939_10232851 | mdtC |
| H3 | Ga0110939_10668271 | mdtC |
| H3 | Ga0110939_10793311 | mdtC |
| H3 | Ga0110939_11438151 | mdtC |
| H3 | Ga0110939_100024281 | mdtD |
| H3 | Ga0110939_10232852 | mdtD |
| H3 | Ga0110939_100066432 | mdtE |
| H3 | Ga0110939_100066433 | mdtF |
| H3 | Ga0110939_11704051 | mdtF |
| H3 | Ga0110939_10006273 | mdtG |
| H3 | Ga0110939_10345861 | mdtG |
| H3 | Ga0110939_10584102 | mdtG |
| H3 | Ga0110939_10837271 | mdtG |
| H3 | Ga0110939_10849001 | mdtG |
| H3 | Ga0110939_100062715 | mdtH |
| H3 | Ga0110939_12990211 | mdtH |
| H3 | Ga0110939_1000245192 | mdtK |
| H3 | Ga0110939_10475481 | mdtK |
| H3 | Ga0110939_1000079210 | mdtL |
| H3 | Ga0110939_12460491 | mdtL |
| H3 | Ga0110939_100051227 | mdtM |
| H3 | Ga0110939_10428861 | mdtM |
| H3 | Ga0110939_11570121 | mdtM |
| H3 | Ga0110939_1000076103 | mdtN |
| H3 | Ga0110939_1000076102 | mdtO |
| H3 | Ga0110939_1000076101 | mdtP |
| H3 | Ga0110939_10007181 | mdtP |
| H3 | Ga0110939_13001301 | mdtP |
| H3 | Ga0110939_10228121 | mefA |
| H3 | Ga0110939_10079326 | mexB |
| H3 | Ga0110939_10184871 | mexB |
| H3 | Ga0110939_10756231 | mexB |
| H3 | Ga0110939_10267686 | mexD |
| H3 | Ga0110939_10283401 | mexD |
| H3 | Ga0110939_10318051 | mexD |
| H3 | Ga0110939_11632721 | mexD |
| H3 | Ga0110939_11798842 | mexE |
| H3 | Ga0110939_11798841 | mexE |
| H3 | Ga0110939_10536091 | mexF |
| H3 | Ga0110939_11406751 | mexF |
| H3 | Ga0110939_11527961 | mexF |
| H3 | Ga0110939_10148641 | mexK |
| H3 | Ga0110939_10281402 | mexK |
| H3 | Ga0110939_10532621 | mexK |
| H3 | Ga0110939_11477881 | mexK |
| H3 | Ga0110939_10148643 | mexL |
| H3 | Ga0110939_11217292 | mexN |
| H3 | Ga0110939_11500421 | mexQ |
| H3 | Ga0110939_10922772 | mexT |
| H3 | Ga0110939_12881371 | mexT |
| H3 | Ga0110939_1000079177 | MFS Antibiotic Efflux Pump |
| H3 | Ga0110939_10000923 | MFS Antibiotic Efflux Pump |
| H3 | Ga0110939_10753011 | MFS Antibiotic Efflux Pump |
| H3 | Ga0110939_10047193 | mphA |
| H3 | Ga0110939_10606161 | msbA |
| H3 | Ga0110939_10796411 | msbA |
| H3 | Ga0110939_10883621 | msbA |
| H3 | Ga0110939_11311522 | msbA |
| H3 | Ga0110939_12507601 | msbA |
| H3 | Ga0110939_13114651 | msbA |
| H3 | Ga0110939_10379931 | msrC |
| H3 | Ga0110939_10119491 | msrE |
| H3 | Ga0110939_11973342 | mtrD |
| H3 | Ga0110939_10933951 | *bla*_NDM-3_ |
| H3 | Ga0110939_10153891 | oprN |
| H3 | Ga0110939_12942391 | *bla*_OXA_ |
| H3 | Ga0110939_10004311 | *bla*_OXA-1_ |
| H3 | Ga0110939_10162981 | *bla*_OXA-10_ |
| H3 | Ga0110939_10793112 | *bla*_OXA-12_ |
| H3 | Ga0110939_10882221 | *bla*_OXA-129_ |
| H3 | Ga0110939_10162971 | *bla*_OXA-13_ |
| H3 | Ga0110939_11379651 | *bla*_OXA-167_ |
| H3 | Ga0110939_10332983 | *bla*_OXA-2_ |
| H3 | Ga0110939_11537451 | *bla*_OXA-211_ |
| H3 | Ga0110939_12516471 | *bla*_OXA-226_ |
| H3 | Ga0110939_100139311 | *bla*_OXA-347_ |
| H3 | Ga0110939_12549431 | *bla*_OXA-5_ |
| H3 | Ga0110939_10472641 | *bla*_OXA-58_ |
| H3 | Ga0110939_100062779 | phoP |
| H3 | Ga0110939_10779671 | phoP |
| H3 | Ga0110939_12090531 | phoP |
| H3 | Ga0110939_100062778 | phoQ |
| H3 | Ga0110939_12078381 | phoQ |
| H3 | Ga0110939_12444741 | phoQ |
| H3 | Ga0110939_1000076134 | PmrA |
| H3 | Ga0110939_13287291 | PmrA |
| H3 | Ga0110939_1000076133 | PmrB |
| H3 | Ga0110939_11442271 | PmrB |
| H3 | Ga0110939_1000076135 | PmrC |
| H3 | Ga0110939_12678201 | PmrC |
| H3 | Ga0110939_100024233 | PmrE |
| H3 | Ga0110939_10982511 | PmrE |
| H3 | Ga0110939_12026981 | PmrE |
| H3 | Ga0110939_100116959 | PmrF |
| H3 | Ga0110939_11368541 | PmrF |
| H3 | Ga0110939_12237211 | PmrF |
| H3 | Ga0110939_12322981 | qepA |
| H3 | Ga0110939_10046362 | QnrB6 |
| H3 | Ga0110939_11230681 | QnrS2 |
| H3 | Ga0110939_10518671 | QnrVC4 |
| H3 | Ga0110939_11230671 | Quinolone Resistance Protein Qnr |
| H3 | Ga0110939_11658271 | ramA |
| H3 | Ga0110939_12179071 | ramA |
| H3 | Ga0110939_12417272 | ramA |
| H3 | Ga0110939_10110481 | RND Antibiotic Efflux Pump |
| H3 | Ga0110939_10135451 | RND Antibiotic Efflux Pump |
| H3 | Ga0110939_10138022 | RND Antibiotic Efflux Pump |
| H3 | Ga0110939_10139011 | RND Antibiotic Efflux Pump |
| H3 | Ga0110939_10161901 | RND Antibiotic Efflux Pump |
| H3 | Ga0110939_10161911 | RND Antibiotic Efflux Pump |
| H3 | Ga0110939_10239283 | RND Antibiotic Efflux Pump |
| H3 | Ga0110939_10276421 | RND Antibiotic Efflux Pump |
| H3 | Ga0110939_10295971 | RND Antibiotic Efflux Pump |
| H3 | Ga0110939_10394661 | RND Antibiotic Efflux Pump |
| H3 | Ga0110939_10444631 | RND Antibiotic Efflux Pump |
| H3 | Ga0110939_10460931 | RND Antibiotic Efflux Pump |
| H3 | Ga0110939_10522062 | RND Antibiotic Efflux Pump |
| H3 | Ga0110939_10536081 | RND Antibiotic Efflux Pump |
| H3 | Ga0110939_10554901 | RND Antibiotic Efflux Pump |
| H3 | Ga0110939_10707552 | RND Antibiotic Efflux Pump |
| H3 | Ga0110939_10734651 | RND Antibiotic Efflux Pump |
| H3 | Ga0110939_10828081 | RND Antibiotic Efflux Pump |
| H3 | Ga0110939_10901691 | RND Antibiotic Efflux Pump |
| H3 | Ga0110939_10901701 | RND Antibiotic Efflux Pump |
| H3 | Ga0110939_11006221 | RND Antibiotic Efflux Pump |
| H3 | Ga0110939_11087611 | RND Antibiotic Efflux Pump |
| H3 | Ga0110939_11113372 | RND Antibiotic Efflux Pump |
| H3 | Ga0110939_11129121 | RND Antibiotic Efflux Pump |
| H3 | Ga0110939_11338041 | RND Antibiotic Efflux Pump |
| H3 | Ga0110939_11459821 | RND Antibiotic Efflux Pump |
| H3 | Ga0110939_11541011 | RND Antibiotic Efflux Pump |
| H3 | Ga0110939_11627581 | RND Antibiotic Efflux Pump |
| H3 | Ga0110939_11631201 | RND Antibiotic Efflux Pump |
| H3 | Ga0110939_11642031 | RND Antibiotic Efflux Pump |
| H3 | Ga0110939_11737421 | RND Antibiotic Efflux Pump |
| H3 | Ga0110939_12084691 | RND Antibiotic Efflux Pump |
| H3 | Ga0110939_12102781 | RND Antibiotic Efflux Pump |
| H3 | Ga0110939_13037881 | RND Antibiotic Efflux Pump |
| H3 | Ga0110939_100031631 | robA |
| H3 | Ga0110939_10863631 | robA |
| H3 | Ga0110939_11929112 | robA |
| H3 | Ga0110939_11658272 | romA |
| H3 | Ga0110939_12754701 | romA |
| H3 | Ga0110939_11747652 | sat-1 |
| H3 | Ga0110939_10141742 | sat-4 |
| H3 | Ga0110939_100007675 | soxR |
| H3 | Ga0110939_11243942 | soxR |
| H3 | Ga0110939_11468242 | soxR |
| H3 | Ga0110939_12496401 | soxR |
| H3 | Ga0110939_12579192 | soxR |
| H3 | Ga0110939_10024361 | sul1 |
| H3 | Ga0110939_10208982 | sul2 |
| H3 | Ga0110939_10001761 | *bla*_TEM_ |
| H3 | Ga0110939_10012111 | tet40 |
| H3 | Ga0110939_10012121 | tet40 |
| H3 | Ga0110939_12937411 | TetH-TetJ |
| H3 | Ga0110939_10022326 | tetO |
| H3 | Ga0110939_10005081 | Tetracycline Resistance MFS Efflux Pump |
| H3 | Ga0110939_10017613 | Tetracycline Resistance MFS Efflux Pump |
| H3 | Ga0110939_10025051 | Tetracycline Resistance MFS Efflux Pump |
| H3 | Ga0110939_10176451 | Tetracycline Resistance MFS Efflux Pump |
| H3 | Ga0110939_10176461 | Tetracycline Resistance MFS Efflux Pump |
| H3 | Ga0110939_10176471 | Tetracycline Resistance MFS Efflux Pump |
| H3 | Ga0110939_10406361 | Tetracycline Resistance MFS Efflux Pump |
| H3 | Ga0110939_11169011 | Tetracycline Resistance MFS Efflux Pump |
| H3 | Ga0110939_11169022 | Tetracycline Resistance MFS Efflux Pump |
| H3 | Ga0110939_12319711 | Tetracycline Resistance MFS Efflux Pump |
| H3 | Ga0110939_12882211 | Tetracycline Resistance MFS Efflux Pump |
| H3 | Ga0110939_10011743 | Tetracycline Resistance Ribosomal Protection Protein |
| H3 | Ga0110939_10014581 | Tetracycline Resistance Ribosomal Protection Protein |
| H3 | Ga0110939_10062093 | Tetracycline Resistance Ribosomal Protection Protein |
| H3 | Ga0110939_10062102 | Tetracycline Resistance Ribosomal Protection Protein |
| H3 | Ga0110939_10092071 | Tetracycline Resistance Ribosomal Protection Protein |
| H3 | Ga0110939_10092081 | Tetracycline Resistance Ribosomal Protection Protein |
| H3 | Ga0110939_10106541 | Tetracycline Resistance Ribosomal Protection Protein |
| H3 | Ga0110939_10165051 | Tetracycline Resistance Ribosomal Protection Protein |
| H3 | Ga0110939_10272011 | Tetracycline Resistance Ribosomal Protection Protein |
| H3 | Ga0110939_10455541 | Tetracycline Resistance Ribosomal Protection Protein |
| H3 | Ga0110939_12070261 | Tetracycline Resistance Ribosomal Protection Protein |
| H3 | Ga0110939_10218963 | tetX |
| H3 | Ga0110939_11821451 | tetX |
| H3 | Ga0110939_100014575 | tolC |
| H3 | Ga0110939_11396721 | tolC |
| H3 | Ga0110939_11401851 | tolC |
| H3 | Ga0110939_11401861 | tolC |
| H3 | Ga0110939_12242991 | tolC |
| H3 | Ga0110939_10321384 | vanA |
| H3 | Ga0110939_10930002 | vanA |
| H3 | Ga0110939_10802462 | vanB |
| H3 | Ga0110939_10232942 | vanG |
| H3 | Ga0110939_10338661 | vanH |
| H3 | Ga0110939_10409511 | vanH |
| H3 | Ga0110939_10930001 | vanH |
| H3 | Ga0110939_10409513 | vanR |
| H3 | Ga0110939_10769814 | vanR |
| H3 | Ga0110939_11303622 | vanR |
| H3 | Ga0110939_11303631 | vanR |
| H3 | Ga0110939_11948122 | vanR |
| H3 | Ga0110939_12677912 | vanR |
| H3 | Ga0110939_13285511 | vanR |
| H3 | Ga0110939_10790291 | vanRO |
| H3 | Ga0110939_10409512 | vanS |
| H3 | Ga0110939_10232944 | vanT |
| H3 | Ga0110939_10584851 | vanT |
| H3 | Ga0110939_11351381 | vanU |
| H3 | Ga0110939_10232941 | vanW |
| H3 | Ga0110939_10321383 | vanX |
| H3 | Ga0110939_10802461 | vanX |
| H3 | Ga0110939_10232943 | vanXYG |
| H3 | Ga0110939_10321382 | vanY |
| H3 | Ga0110939_10321381 | vanZ |
| H3 | Ga0110939_12824821 | vatB |
| H3 | Ga0110939_12287451 | *bla*_VEB-1a_ |
| H4 | Ga0110940_10115381 | AAC(6')-Ib11 |
| H4 | Ga0110940_10152312 | AAC(6')-Ib11 |
| H4 | Ga0110940_10080731 | AAC(6')-Ib9 |
| H4 | Ga0110940_10844731 | AAC(6')-Ib9 |
| H4 | Ga0110940_10195671 | AAC(6')-Ie-APH(2'')-Ia |
| H4 | Ga0110940_10501411 | AAC(6')-Ie-APH(2'')-Ia |
| H4 | Ga0110940_10082423 | AAC(6')-Ie-APH(2'')-Ia |
| H4 | Ga0110940_10218153 | AAC(6')-Ie-APH(2'')-Ia |
| H4 | Ga0110940_12890751 | AAC(6')-IIc |
| H4 | Ga0110940_10318011 | AAC(6')-IIc |
| H4 | Ga0110940_11415091 | AAC3 |
| H4 | Ga0110940_12356301 | AAC3 |
| H4 | Ga0110940_11415211 | AAC3-Ia |
| H4 | Ga0110940_10185427 | AAC3-Ia |
| H4 | Ga0110940_11549161 | aadA6/aadA10 |
| H4 | Ga0110940_11913131 | aadA6/aadA10 |
| H4 | Ga0110940_10423141 | ABC Antibiotic Efflux Pump |
| H4 | Ga0110940_10635041 | ABC Antibiotic Efflux Pump |
| H4 | Ga0110940_10635051 | ABC Antibiotic Efflux Pump |
| H4 | Ga0110940_10659161 | ABC Antibiotic Efflux Pump |
| H4 | Ga0110940_10659171 | ABC Antibiotic Efflux Pump |
| H4 | Ga0110940_10659181 | ABC Antibiotic Efflux Pump |
| H4 | Ga0110940_11344311 | ABC Antibiotic Efflux Pump |
| H4 | Ga0110940_11355381 | ABC Antibiotic Efflux Pump |
| H4 | Ga0110940_11357171 | ABC Antibiotic Efflux Pump |
| H4 | Ga0110940_10462941 | abeM |
| H4 | Ga0110940_11435201 | abeM |
| H4 | Ga0110940_11862591 | abeM |
| H4 | Ga0110940_12414681 | acrA |
| H4 | Ga0110940_10981421 | acrB |
| H4 | Ga0110940_12143811 | acrB |
| H4 | Ga0110940_12481621 | acrB |
| H4 | Ga0110940_10386801 | acrD |
| H4 | Ga0110940_11199421 | acrD |
| H4 | Ga0110940_13010411 | acrD |
| H4 | Ga0110940_10348431 | acrF |
| H4 | Ga0110940_10433881 | acrF |
| H4 | Ga0110940_11127701 | acrF |
| H4 | Ga0110940_11178001 | acrF |
| H4 | Ga0110940_11541481 | acrF |
| H4 | Ga0110940_13025431 | acrF |
| H4 | Ga0110940_11390821 | acrS |
| H4 | Ga0110940_12628251 | acrS |
| H4 | Ga0110940_10200031 | adeB |
| H4 | Ga0110940_10347951 | adeB |
| H4 | Ga0110940_10390631 | adeB |
| H4 | Ga0110940_10825961 | adeB |
| H4 | Ga0110940_10825971 | adeB |
| H4 | Ga0110940_10834801 | adeB |
| H4 | Ga0110940_11587381 | adeB |
| H4 | Ga0110940_11616961 | adeB |
| H4 | Ga0110940_11749471 | adeB |
| H4 | Ga0110940_11241681 | adeC-adeK-oprM |
| H4 | Ga0110940_12211601 | adeC-adeK-oprM |
| H4 | Ga0110940_10229661 | adeG |
| H4 | Ga0110940_10229681 | adeG |
| H4 | Ga0110940_12049691 | adeG |
| H4 | Ga0110940_12272231 | adeG |
| H4 | Ga0110940_11241682 | adeK |
| H4 | Ga0110940_11334951 | adeK |
| H4 | Ga0110940_10749272 | ampC |
| H4 | Ga0110940_10749281 | ampC |
| H4 | Ga0110940_10306791 | ANT(2'')-Ia |
| H4 | Ga0110940_10024522 | ANT(2'')-Ia |
| H4 | Ga0110940_10218152 | ANT(6)-Ia |
| H4 | Ga0110940_10044771 | APH3" |
| H4 | Ga0110940_10069531 | APH3" |
| H4 | Ga0110940_10245041 | APH3" |
| H4 | Ga0110940_10275353 | APH3" |
| H4 | Ga0110940_10295312 | APH3" |
| H4 | Ga0110940_10295332 | APH3" |
| H4 | Ga0110940_10295352 | APH3" |
| H4 | Ga0110940_10490181 | APH3" |
| H4 | Ga0110940_10581522 | APH3" |
| H4 | Ga0110940_10581531 | APH3" |
| H4 | Ga0110940_10792221 | APH3" |
| H4 | Ga0110940_10993921 | APH3" |
| H4 | Ga0110940_11287972 | APH3" |
| H4 | Ga0110940_11724711 | APH3" |
| H4 | Ga0110940_12262911 | APH3" |
| H4 | Ga0110940_12925461 | APH3" |
| H4 | Ga0110940_13093161 | APH3" |
| H4 | Ga0110940_10218155 | APH3' |
| H4 | Ga0110940_10324281 | APH3' |
| H4 | Ga0110940_10024521 | APH6 |
| H4 | Ga0110940_12686001 | armA |
| H4 | Ga0110940_11142842 | arnA |
| H4 | Ga0110940_11768441 | arnA |
| H4 | Ga0110940_10838331 | bacA |
| H4 | Ga0110940_12386101 | bacA |
| H4 | Ga0110940_10471561 | baeR |
| H4 | Ga0110940_11393391 | baeR |
| H4 | Ga0110940_12093961 | baeR |
| H4 | Ga0110940_10329291 | baeS |
| H4 | Ga0110940_10027142 | Beta-lactamase Class A |
| H4 | Ga0110940_10433411 | Beta-lactamase Class A |
| H4 | Ga0110940_11219421 | Beta-lactamase Class A |
| H4 | Ga0110940_12592831 | Beta-lactamase Class A |
| H4 | Ga0110940_12836121 | Beta-lactamase Class A |
| H4 | Ga0110940_10070631 | *bla*_IMP-1_ |
| H4 | Ga0110940_12774821 | *bla*_TEM-163_ |
| H4 | Ga0110940_11608161 | *bla*_VIM-12_ |
| H4 | Ga0110940_12575572 | bleomycin resistance protein (BRP) |
| H4 | Ga0110940_11369651 | cat |
| H4 | Ga0110940_11150921 | catB2 |
| H4 | Ga0110940_11204851 | cblA |
| H4 | Ga0110940_11982511 | cblA |
| H4 | Ga0110940_10554402 | Cfr 23 Ribosomal RNA Methyltransferase |
| H4 | Ga0110940_10751192 | Cfr 23 Ribosomal RNA Methyltransferase |
| H4 | Ga0110940_11145271 | Cfr 23 Ribosomal RNA Methyltransferase |
| H4 | Ga0110940_10027151 | cfxA6 |
| H4 | Ga0110940_10064251 | Chloramphenicol Acetyltransferase CAT |
| H4 | Ga0110940_10421751 | Chloramphenicol Acetyltransferase CAT |
| H4 | Ga0110940_10978941 | Chloramphenicol Acetyltransferase CAT |
| H4 | Ga0110940_11315341 | Chloramphenicol Acetyltransferase CAT |
| H4 | Ga0110940_11428162 | Chloramphenicol Acetyltransferase CAT |
| H4 | Ga0110940_11841641 | Chloramphenicol Acetyltransferase CAT |
| H4 | Ga0110940_11862432 | Chloramphenicol Acetyltransferase CAT |
| H4 | Ga0110940_12129511 | Chloramphenicol Acetyltransferase CAT |
| H4 | Ga0110940_10708571 | Chloramphenicol Efflux Pump |
| H4 | Ga0110940_11334392 | Chloramphenicol Efflux Pump |
| H4 | Ga0110940_11619571 | Chloramphenicol Efflux Pump |
| H4 | Ga0110940_11638021 | Chloramphenicol Efflux Pump |
| H4 | Ga0110940_12418891 | Chloramphenicol Efflux Pump |
| H4 | Ga0110940_12524981 | Chloramphenicol Efflux Pump |
| H4 | Ga0110940_12666971 | cmlB1 |
| H4 | Ga0110940_11091471 | cpxA |
| H4 | Ga0110940_11405781 | cpxA |
| H4 | Ga0110940_11405791 | cpxA |
| H4 | Ga0110940_11091472 | cpxR |
| H4 | Ga0110940_10649621 | CRP |
| H4 | Ga0110940_11727431 | CRP |
| H4 | Ga0110940_12219581 | CRP |
| H4 | Ga0110940_12755311 | CRP |
| H4 | Ga0110940_12538951 | dfrA1 |
| H4 | Ga0110940_12210541 | dfrA14 |
| H4 | Ga0110940_10601961 | dfrE |
| H4 | Ga0110940_10055241 | dfrF |
| H4 | Ga0110940_12819821 | dfrG |
| H4 | Ga0110940_11401721 | emrA |
| H4 | Ga0110940_12000741 | emrA |
| H4 | Ga0110940_10613531 | emrB |
| H4 | Ga0110940_10613542 | emrB |
| H4 | Ga0110940_10937811 | emrB |
| H4 | Ga0110940_11057001 | emrB |
| H4 | Ga0110940_11057011 | emrB |
| H4 | Ga0110940_11070494 | emrB |
| H4 | Ga0110940_11310652 | emrD |
| H4 | Ga0110940_12945011 | emrD |
| H4 | Ga0110940_10948791 | emrK |
| H4 | Ga0110940_12406951 | emrK |
| H4 | Ga0110940_11210931 | emrR |
| H4 | Ga0110940_10120071 | emrY |
| H4 | Ga0110940_12406952 | emrY |
| H4 | Ga0110940_10137241 | ereA |
| H4 | Ga0110940_10876001 | ereA |
| H4 | Ga0110940_10137251 | ereA7 |
| H4 | Ga0110940_11546142 | ereB |
| H4 | Ga0110940_11833411 | ereB |
| H4 | Ga0110940_12248481 | ereB |
| H4 | Ga0110940_10145481 | Erm 23S ribosomal RNA Methyltransferase |
| H4 | Ga0110940_10661711 | Erm 23S ribosomal RNA Methyltransferase |
| H4 | Ga0110940_11075831 | Erm 23S ribosomal RNA Methyltransferase |
| H4 | Ga0110940_10069403 | ermB |
| H4 | Ga0110940_10661701 | ermF |
| H4 | Ga0110940_10840541 | ermF |
| H4 | Ga0110940_10385171 | ermG |
| H4 | Ga0110940_10988751 | *bla*_ESP-1_ |
| H4 | Ga0110940_10687241 | evgS |
| H4 | Ga0110940_12054141 | evgS |
| H4 | Ga0110940_12583261 | evgS |
| H4 | Ga0110940_10122581 | Fluoroquinolone Resistant DNA Topoisomerase |
| H4 | Ga0110940_10277901 | Fluoroquinolone Resistant DNA Topoisomerase |
| H4 | Ga0110940_10423791 | Fluoroquinolone Resistant DNA Topoisomerase |
| H4 | Ga0110940_10497701 | Fluoroquinolone Resistant DNA Topoisomerase |
| H4 | Ga0110940_10542671 | Fluoroquinolone Resistant DNA Topoisomerase |
| H4 | Ga0110940_10575721 | Fluoroquinolone Resistant DNA Topoisomerase |
| H4 | Ga0110940_10594671 | Fluoroquinolone Resistant DNA Topoisomerase |
| H4 | Ga0110940_10600112 | Fluoroquinolone Resistant DNA Topoisomerase |
| H4 | Ga0110940_10781372 | Fluoroquinolone Resistant DNA Topoisomerase |
| H4 | Ga0110940_10781592 | Fluoroquinolone Resistant DNA Topoisomerase |
| H4 | Ga0110940_10801121 | Fluoroquinolone Resistant DNA Topoisomerase |
| H4 | Ga0110940_10801131 | Fluoroquinolone Resistant DNA Topoisomerase |
| H4 | Ga0110940_10823521 | Fluoroquinolone Resistant DNA Topoisomerase |
| H4 | Ga0110940_10835051 | Fluoroquinolone Resistant DNA Topoisomerase |
| H4 | Ga0110940_10902131 | Fluoroquinolone Resistant DNA Topoisomerase |
| H4 | Ga0110940_11173551 | Fluoroquinolone Resistant DNA Topoisomerase |
| H4 | Ga0110940_11237721 | Fluoroquinolone Resistant DNA Topoisomerase |
| H4 | Ga0110940_11306341 | Fluoroquinolone Resistant DNA Topoisomerase |
| H4 | Ga0110940_11306351 | Fluoroquinolone Resistant DNA Topoisomerase |
| H4 | Ga0110940_11358571 | Fluoroquinolone Resistant DNA Topoisomerase |
| H4 | Ga0110940_11374891 | Fluoroquinolone Resistant DNA Topoisomerase |
| H4 | Ga0110940_11402311 | Fluoroquinolone Resistant DNA Topoisomerase |
| H4 | Ga0110940_11437121 | Fluoroquinolone Resistant DNA Topoisomerase |
| H4 | Ga0110940_11493171 | Fluoroquinolone Resistant DNA Topoisomerase |
| H4 | Ga0110940_11493181 | Fluoroquinolone Resistant DNA Topoisomerase |
| H4 | Ga0110940_11495271 | Fluoroquinolone Resistant DNA Topoisomerase |
| H4 | Ga0110940_11876721 | Fluoroquinolone Resistant DNA Topoisomerase |
| H4 | Ga0110940_11884802 | Fluoroquinolone Resistant DNA Topoisomerase |
| H4 | Ga0110940_11884811 | Fluoroquinolone Resistant DNA Topoisomerase |
| H4 | Ga0110940_11968851 | Fluoroquinolone Resistant DNA Topoisomerase |
| H4 | Ga0110940_11994191 | Fluoroquinolone Resistant DNA Topoisomerase |
| H4 | Ga0110940_12141171 | Fluoroquinolone Resistant DNA Topoisomerase |
| H4 | Ga0110940_12216911 | Fluoroquinolone Resistant DNA Topoisomerase |
| H4 | Ga0110940_12395061 | Fluoroquinolone Resistant DNA Topoisomerase |
| H4 | Ga0110940_12435391 | Fluoroquinolone Resistant DNA Topoisomerase |
| H4 | Ga0110940_12440361 | Fluoroquinolone Resistant DNA Topoisomerase |
| H4 | Ga0110940_12482141 | Fluoroquinolone Resistant DNA Topoisomerase |
| H4 | Ga0110940_12726571 | Fluoroquinolone Resistant DNA Topoisomerase |
| H4 | Ga0110940_11296031 | gadX |
| H4 | Ga0110940_10093131 | *bla*_GES-1_ |
| H4 | Ga0110940_11637482 | *bla*_GES-21_ |
| H4 | Ga0110940_12558522 | H-NS |
| H4 | Ga0110940_12951052 | *bla*_LCR-1_ |
| H4 | Ga0110940_100325228 | lnuC |
| H4 | Ga0110940_11128861 | macA |
| H4 | Ga0110940_11132541 | macA |
| H4 | Ga0110940_11194851 | macA |
| H4 | Ga0110940_11194852 | macA |
| H4 | Ga0110940_10407392 | macB |
| H4 | Ga0110940_11330911 | macB |
| H4 | Ga0110940_11785902 | macB |
| H4 | Ga0110940_12142001 | macB |
| H4 | Ga0110940_12715141 | macB |
| H4 | Ga0110940_13059811 | mdsA |
| H4 | Ga0110940_13041961 | mdsB |
| H4 | Ga0110940_11504451 | mdtA |
| H4 | Ga0110940_10176571 | mdtB |
| H4 | Ga0110940_11369781 | mdtB |
| H4 | Ga0110940_11889272 | mdtB |
| H4 | Ga0110940_12040461 | mdtB |
| H4 | Ga0110940_12069071 | mdtB |
| H4 | Ga0110940_10891271 | mdtC |
| H4 | Ga0110940_11120211 | mdtC |
| H4 | Ga0110940_12040462 | mdtC |
| H4 | Ga0110940_10891272 | mdtD |
| H4 | Ga0110940_12315451 | mdtD |
| H4 | Ga0110940_10228441 | mdtF |
| H4 | Ga0110940_11980191 | mdtF |
| H4 | Ga0110940_12034832 | mdtG |
| H4 | Ga0110940_12327681 | mdtH |
| H4 | Ga0110940_12494771 | mdtH |
| H4 | Ga0110940_12600091 | mdtK |
| H4 | Ga0110940_13069641 | mdtK |
| H4 | Ga0110940_11416211 | mdtL |
| H4 | Ga0110940_12378991 | mdtL |
| H4 | Ga0110940_11626191 | mdtN |
| H4 | Ga0110940_12151281 | mdtO |
| H4 | Ga0110940_12227121 | mdtO |
| H4 | Ga0110940_10430421 | mdtP |
| H4 | Ga0110940_10441022 | mefA |
| H4 | Ga0110940_10635042 | mefA |
| H4 | Ga0110940_10964321 | mefA |
| H4 | Ga0110940_10736141 | mexB |
| H4 | Ga0110940_11843061 | mexB |
| H4 | Ga0110940_10177471 | mexD |
| H4 | Ga0110940_10996001 | mexD |
| H4 | Ga0110940_11057351 | mexD |
| H4 | Ga0110940_12734311 | mexE |
| H4 | Ga0110940_11122581 | mexK |
| H4 | Ga0110940_11943302 | mexK |
| H4 | Ga0110940_11207102 | mexT |
| H4 | Ga0110940_10046041 | MFS Antibiotic Efflux Pump |
| H4 | Ga0110940_12821451 | MFS Antibiotic Efflux Pump |
| H4 | Ga0110940_10559272 | mphA |
| H4 | Ga0110940_10470201 | msbA |
| H4 | Ga0110940_11095031 | msbA |
| H4 | Ga0110940_11095041 | msbA |
| H4 | Ga0110940_10081981 | msrE |
| H4 | Ga0110940_12575571 | *bla*_NDM-3_ |
| H4 | Ga0110940_11535081 | oprN |
| H4 | Ga0110940_11612352 | oprN |
| H4 | Ga0110940_10828871 | *bla*_OXA-1_ |
| H4 | Ga0110940_10262301 | *bla*_OXA-10_ |
| H4 | Ga0110940_10743071 | *bla*_OXA-2_ |
| H4 | Ga0110940_10083361 | *bla*_OXA-225_ |
| H4 | Ga0110940_10543181 | *bla*_OXA-347_ |
| H4 | Ga0110940_10466561 | *bla*_OXA-5_ |
| H4 | Ga0110940_12631741 | *bla*_OXA-5_ |
| H4 | Ga0110940_10214261 | *bla*_OXA-58_ |
| H4 | Ga0110940_11293001 | PBP2b |
| H4 | Ga0110940_12820111 | PBP2b |
| H4 | Ga0110940_12668661 | phoP |
| H4 | Ga0110940_11557461 | phoQ |
| H4 | Ga0110940_11735541 | PmrA |
| H4 | Ga0110940_11166151 | PmrC |
| H4 | Ga0110940_11255551 | PmrE |
| H4 | Ga0110940_11409411 | PmrE |
| H4 | Ga0110940_12179761 | PmrE |
| H4 | Ga0110940_10291631 | PmrF |
| H4 | Ga0110940_11311861 | pp-flo |
| H4 | Ga0110940_11514821 | Quinolone Resistance Protein Qnr |
| H4 | Ga0110940_10070191 | RND Antibiotic Efflux Pump |
| H4 | Ga0110940_10077442 | RND Antibiotic Efflux Pump |
| H4 | Ga0110940_10120251 | RND Antibiotic Efflux Pump |
| H4 | Ga0110940_10185121 | RND Antibiotic Efflux Pump |
| H4 | Ga0110940_10306602 | RND Antibiotic Efflux Pump |
| H4 | Ga0110940_10360271 | RND Antibiotic Efflux Pump |
| H4 | Ga0110940_10360391 | RND Antibiotic Efflux Pump |
| H4 | Ga0110940_10452741 | RND Antibiotic Efflux Pump |
| H4 | Ga0110940_10455951 | RND Antibiotic Efflux Pump |
| H4 | Ga0110940_10657011 | RND Antibiotic Efflux Pump |
| H4 | Ga0110940_10788402 | RND Antibiotic Efflux Pump |
| H4 | Ga0110940_11033301 | RND Antibiotic Efflux Pump |
| H4 | Ga0110940_11242431 | RND Antibiotic Efflux Pump |
| H4 | Ga0110940_11298402 | RND Antibiotic Efflux Pump |
| H4 | Ga0110940_11575482 | RND Antibiotic Efflux Pump |
| H4 | Ga0110940_11614841 | RND Antibiotic Efflux Pump |
| H4 | Ga0110940_11647601 | RND Antibiotic Efflux Pump |
| H4 | Ga0110940_11728391 | RND Antibiotic Efflux Pump |
| H4 | Ga0110940_11802661 | RND Antibiotic Efflux Pump |
| H4 | Ga0110940_11844111 | RND Antibiotic Efflux Pump |
| H4 | Ga0110940_12168831 | RND Antibiotic Efflux Pump |
| H4 | Ga0110940_12207212 | RND Antibiotic Efflux Pump |
| H4 | Ga0110940_12232332 | RND Antibiotic Efflux Pump |
| H4 | Ga0110940_12232341 | RND Antibiotic Efflux Pump |
| H4 | Ga0110940_12288921 | RND Antibiotic Efflux Pump |
| H4 | Ga0110940_12292481 | RND Antibiotic Efflux Pump |
| H4 | Ga0110940_12398901 | RND Antibiotic Efflux Pump |
| H4 | Ga0110940_12716191 | RND Antibiotic Efflux Pump |
| H4 | Ga0110940_12963921 | RND Antibiotic Efflux Pump |
| H4 | Ga0110940_10940661 | rosB |
| H4 | Ga0110940_11154961 | rosB |
| H4 | Ga0110940_12264841 | rosB |
| H4 | Ga0110940_10218154 | sat-4 |
| H4 | Ga0110940_12076971 | *bla*_SHV-123_ |
| H4 | Ga0110940_11376312 | soxR |
| H4 | Ga0110940_10097341 | sul1 |
| H4 | Ga0110940_10096031 | sul2 |
| H4 | Ga0110940_10140941 | tet40 |
| H4 | Ga0110940_10092802 | tetO |
| H4 | Ga0110940_10053372 | Tetracycline Resistance MFS Efflux Pump |
| H4 | Ga0110940_10185422 | Tetracycline Resistance MFS Efflux Pump |
| H4 | Ga0110940_10452041 | Tetracycline Resistance MFS Efflux Pump |
| H4 | Ga0110940_11794681 | Tetracycline Resistance MFS Efflux Pump |
| H4 | Ga0110940_10039192 | Tetracycline Resistance Ribosomal Protection Protein |
| H4 | Ga0110940_10051742 | Tetracycline Resistance Ribosomal Protection Protein |
| H4 | Ga0110940_10081611 | Tetracycline Resistance Ribosomal Protection Protein |
| H4 | Ga0110940_10092431 | Tetracycline Resistance Ribosomal Protection Protein |
| H4 | Ga0110940_10092791 | Tetracycline Resistance Ribosomal Protection Protein |
| H4 | Ga0110940_10132311 | Tetracycline Resistance Ribosomal Protection Protein |
| H4 | Ga0110940_10139321 | Tetracycline Resistance Ribosomal Protection Protein |
| H4 | Ga0110940_10175361 | Tetracycline Resistance Ribosomal Protection Protein |
| H4 | Ga0110940_10184861 | Tetracycline Resistance Ribosomal Protection Protein |
| H4 | Ga0110940_10501901 | Tetracycline Resistance Ribosomal Protection Protein |
| H4 | Ga0110940_10553781 | Tetracycline Resistance Ribosomal Protection Protein |
| H4 | Ga0110940_10826231 | Tetracycline Resistance Ribosomal Protection Protein |
| H4 | Ga0110940_11322261 | Tetracycline Resistance Ribosomal Protection Protein |
| H4 | Ga0110940_11349652 | Tetracycline Resistance Ribosomal Protection Protein |
| H4 | Ga0110940_11365651 | Tetracycline Resistance Ribosomal Protection Protein |
| H4 | Ga0110940_11365661 | Tetracycline Resistance Ribosomal Protection Protein |
| H4 | Ga0110940_11365671 | Tetracycline Resistance Ribosomal Protection Protein |
| H4 | Ga0110940_11394491 | Tetracycline Resistance Ribosomal Protection Protein |
| H4 | Ga0110940_11964231 | Tetracycline Resistance Ribosomal Protection Protein |
| H4 | Ga0110940_12565671 | Tetracycline Resistance Ribosomal Protection Protein |
| H4 | Ga0110940_12613561 | Tetracycline Resistance Ribosomal Protection Protein |
| H4 | Ga0110940_12772641 | Tetracycline Resistance Ribosomal Protection Protein |
| H4 | Ga0110940_13069471 | Tetracycline Resistance Ribosomal Protection Protein |
| H4 | Ga0110940_10444031 | tetX |
| H4 | Ga0110940_10225581 | tolC |
| H4 | Ga0110940_12223141 | tolC |
| H4 | Ga0110940_10846832 | vanG |
| H4 | Ga0110940_11759671 | vanR |
| H4 | Ga0110940_11780301 | vanR |
| H4 | Ga0110940_10247742 | vanT |
| H4 | Ga0110940_10516492 | vanT |
| H4 | Ga0110940_10770212 | vanU |
| H4 | Ga0110940_10430881 | vanW |
| H4 | Ga0110940_11821241 | vanW |
| H4 | Ga0110940_12189811 | vanW |
| H4 | Ga0110940_10516491 | vanXYG |
| H4 | Ga0110940_10846833 | vanXYG |
| H4 | Ga0110940_11353581 | vanY |
| H4 | Ga0110940_10306802 | *bla*_VEB-1b_ |
| H5 | Ga0110941_10039991 | AAC(6')-Ib9 |
| H5 | Ga0110941_11259701 | AAC(6')-Ic |
| H5 | Ga0110941_10187731 | AAC(6')-Ie-APH(2'')-Ia |
| H5 | Ga0110941_10004111 | AAC3 |
| H5 | Ga0110941_10697991 | AAC6-Ia |
| H5 | Ga0110941_11096641 | AAC6-II |
| H5 | Ga0110941_10072791 | aadA16 |
| H5 | Ga0110941_11764342 | aadA6/aadA10 |
| H5 | Ga0110941_10952991 | aadA7 |
| H5 | Ga0110941_10743121 | ABC Antibiotic Efflux Pump |
| H5 | Ga0110941_10743131 | ABC Antibiotic Efflux Pump |
| H5 | Ga0110941_10880361 | ABC Antibiotic Efflux Pump |
| H5 | Ga0110941_11296911 | ABC Antibiotic Efflux Pump |
| H5 | Ga0110941_11604861 | ABC Antibiotic Efflux Pump |
| H5 | Ga0110941_10459584 | abeM |
| H5 | Ga0110941_10835722 | abeM |
| H5 | Ga0110941_10835731 | abeM |
| H5 | Ga0110941_10835741 | abeM |
| H5 | Ga0110941_11011651 | abeM |
| H5 | Ga0110941_11709381 | abeS |
| H5 | Ga0110941_10379771 | acrA |
| H5 | Ga0110941_10961551 | acrA |
| H5 | Ga0110941_11339682 | acrA |
| H5 | Ga0110941_11483581 | acrA |
| H5 | Ga0110941_10197771 | acrB |
| H5 | Ga0110941_10310781 | acrB |
| H5 | Ga0110941_11591061 | acrB |
| H5 | Ga0110941_11893141 | acrB |
| H5 | Ga0110941_11643551 | acrD |
| H5 | Ga0110941_11873831 | acrD |
| H5 | Ga0110941_11150621 | acrE |
| H5 | Ga0110941_10564151 | acrF |
| H5 | Ga0110941_11256031 | acrF |
| H5 | Ga0110941_11552971 | acrF |
| H5 | Ga0110941_11716151 | acrF |
| H5 | Ga0110941_12038091 | acrF |
| H5 | Ga0110941_12117731 | acrS |
| H5 | Ga0110941_12160461 | *bla*_ACT-27_ |
| H5 | Ga0110941_10745861 | *bla*_ACT-31_ |
| H5 | Ga0110941_11536371 | adeA |
| H5 | Ga0110941_10223301 | adeA-adeI |
| H5 | Ga0110941_11293711 | adeA-adeI |
| H5 | Ga0110941_11442711 | adeA-adeI |
| H5 | Ga0110941_11536361 | adeA-adeI |
| H5 | Ga0110941_10283802 | adeB |
| H5 | Ga0110941_10515522 | adeB |
| H5 | Ga0110941_11071881 | adeB |
| H5 | Ga0110941_11346581 | adeB |
| H5 | Ga0110941_11364701 | adeB |
| H5 | Ga0110941_11579361 | adeB |
| H5 | Ga0110941_11695491 | adeB |
| H5 | Ga0110941_11706901 | adeB |
| H5 | Ga0110941_11851161 | adeB |
| H5 | Ga0110941_11866231 | adeB |
| H5 | Ga0110941_11910111 | adeB |
| H5 | Ga0110941_12311021 | adeB |
| H5 | Ga0110941_10583242 | adeC |
| H5 | Ga0110941_10137565 | adeC-adeK-oprM |
| H5 | Ga0110941_10289511 | adeC-adeK-oprM |
| H5 | Ga0110941_10367321 | adeC-adeK-oprM |
| H5 | Ga0110941_10448761 | adeC-adeK-oprM |
| H5 | Ga0110941_10528921 | adeC-adeK-oprM |
| H5 | Ga0110941_11032941 | adeC-adeK-oprM |
| H5 | Ga0110941_11422661 | adeC-adeK-oprM |
| H5 | Ga0110941_11782491 | adeC-adeK-oprM |
| H5 | Ga0110941_11941991 | adeC-adeK-oprM |
| H5 | Ga0110941_12221601 | adeC-adeK-oprM |
| H5 | Ga0110941_10023721 | adeG |
| H5 | Ga0110941_10165161 | adeG |
| H5 | Ga0110941_11873231 | adeG |
| H5 | Ga0110941_12434661 | adeG |
| H5 | Ga0110941_12482271 | adeG |
| H5 | Ga0110941_12209501 | adeJ |
| H5 | Ga0110941_100003112 | ampC |
| H5 | Ga0110941_10178301 | ampC |
| H5 | Ga0110941_10873001 | ampC |
| H5 | Ga0110941_11147311 | ampC |
| H5 | Ga0110941_11422652 | ampC |
| H5 | Ga0110941_10023731 | amrB |
| H5 | Ga0110941_11717811 | amrB |
| H5 | Ga0110941_10277041 | ANT(2'')-Ia |
| H5 | Ga0110941_11618632 | ANT(6)-Ia |
| H5 | Ga0110941_10014191 | ANT3 |
| H5 | Ga0110941_10043151 | ANT3 |
| H5 | Ga0110941_10102961 | ANT3 |
| H5 | Ga0110941_11291742 | ANT3 |
| H5 | Ga0110941_10032242 | ANT6 |
| H5 | Ga0110941_10058472 | APH3 |
| H5 | Ga0110941_11127211 | APH3 |
| H5 | Ga0110941_10002442 | APH3" |
| H5 | Ga0110941_10020616 | APH3" |
| H5 | Ga0110941_10046241 | APH3" |
| H5 | Ga0110941_10046513 | APH3" |
| H5 | Ga0110941_10170151 | APH3" |
| H5 | Ga0110941_10170161 | APH3" |
| H5 | Ga0110941_10301512 | APH3" |
| H5 | Ga0110941_10308863 | APH3" |
| H5 | Ga0110941_10332491 | APH3" |
| H5 | Ga0110941_11045511 | APH3" |
| H5 | Ga0110941_11045521 | APH3" |
| H5 | Ga0110941_11180811 | APH3" |
| H5 | Ga0110941_11607671 | APH3" |
| H5 | Ga0110941_11627141 | APH3" |
| H5 | Ga0110941_11082301 | APH3' |
| H5 | Ga0110941_11151381 | APH3' |
| H5 | Ga0110941_10002441 | APH6 |
| H5 | Ga0110941_11279931 | armA |
| H5 | Ga0110941_11279932 | armA |
| H5 | Ga0110941_11527481 | armA |
| H5 | Ga0110941_11234331 | arnA |
| H5 | Ga0110941_11643361 | arnA |
| H5 | Ga0110941_11927801 | arnA |
| H5 | Ga0110941_11937881 | arnA |
| H5 | Ga0110941_12412441 | arnA |
| H5 | Ga0110941_10218431 | arr-3 |
| H5 | Ga0110941_10302693 | bacA |
| H5 | Ga0110941_11298061 | bacA |
| H5 | Ga0110941_10010601 | baeR |
| H5 | Ga0110941_10054261 | baeR |
| H5 | Ga0110941_11000571 | baeR |
| H5 | Ga0110941_11152961 | baeR |
| H5 | Ga0110941_11339931 | baeR |
| H5 | Ga0110941_10393381 | baeS |
| H5 | Ga0110941_10636241 | baeS |
| H5 | Ga0110941_11051941 | baeS |
| H5 | Ga0110941_11275371 | baeS |
| H5 | Ga0110941_12256082 | baeS |
| H5 | Ga0110941_12492691 | baeS |
| H5 | Ga0110941_10473941 | *bla*_AER-1_ |
| H5 | Ga0110941_11308731 | *bla*_CARB-7_ |
| H5 | Ga0110941_10931361 | *bla*_DHA-7_ |
| H5 | Ga0110941_11518171 | *bla*_DHA-7_ |
| H5 | Ga0110941_10093731 | *bla*_IMP-1_ |
| H5 | Ga0110941_11075181 | *bla*_OXA_ |
| H5 | Ga0110941_11322534 | *bla*_OXA-141_ |
| H5 | Ga0110941_10015853 | *bla*_OXA-2_ |
| H5 | Ga0110941_10095812 | *bla*_PER-3_ |
| H5 | Ga0110941_11441831 | *bla*_PSE-1_ |
| H5 | Ga0110941_10901642 | *bla*_SHV-17_ |
| H5 | Ga0110941_10030191 | *bla*_TEM_ |
| H5 | Ga0110941_12022731 | *bla*_VIM-3_ |
| H5 | Ga0110941_10007652 | cat |
| H5 | Ga0110941_10101861 | CAT1_CLOPF |
| H5 | Ga0110941_10688681 | cblA |
| H5 | Ga0110941_10062621 | ceoB |
| H5 | Ga0110941_10208541 | ceoB |
| H5 | Ga0110941_10738832 | ceoB |
| H5 | Ga0110941_10815951 | ceoB |
| H5 | Ga0110941_11226531 | ceoB |
| H5 | Ga0110941_12274751 | ceoB |
| H5 | Ga0110941_10044372 | Cfr 23 Ribosomal RNA Methyltransferase |
| H5 | Ga0110941_10903211 | Cfr 23 Ribosomal RNA Methyltransferase |
| H5 | Ga0110941_10011252 | cfxA |
| H5 | Ga0110941_10271931 | cfxA6 |
| H5 | Ga0110941_10269861 | Chloramphenicol Acetyltransferase CAT |
| H5 | Ga0110941_10440292 | Chloramphenicol Acetyltransferase CAT |
| H5 | Ga0110941_10930721 | Chloramphenicol Acetyltransferase CAT |
| H5 | Ga0110941_11245071 | Chloramphenicol Acetyltransferase CAT |
| H5 | Ga0110941_11681262 | Chloramphenicol Acetyltransferase CAT |
| H5 | Ga0110941_11851522 | Chloramphenicol Acetyltransferase CAT |
| H5 | Ga0110941_12028672 | Chloramphenicol Acetyltransferase CAT |
| H5 | Ga0110941_12390041 | Chloramphenicol Acetyltransferase CAT |
| H5 | Ga0110941_10218441 | Chloramphenicol Efflux Pump |
| H5 | Ga0110941_10430421 | Chloramphenicol Efflux Pump |
| H5 | Ga0110941_10430431 | Chloramphenicol Efflux Pump |
| H5 | Ga0110941_11037322 | Chloramphenicol Efflux Pump |
| H5 | Ga0110941_12131701 | Chloramphenicol Efflux Pump |
| H5 | Ga0110941_11098231 | cmlB1 |
| H5 | Ga0110941_12222871 | cpxA |
| H5 | Ga0110941_10207751 | cpxR |
| H5 | Ga0110941_10485201 | cpxR |
| H5 | Ga0110941_10690751 | cpxR |
| H5 | Ga0110941_10845261 | cpxR |
| H5 | Ga0110941_100011712 | CRP |
| H5 | Ga0110941_10016511 | CRP |
| H5 | Ga0110941_10208141 | CRP |
| H5 | Ga0110941_10452652 | CRP |
| H5 | Ga0110941_10265521 | dfrA1 |
| H5 | Ga0110941_11190991 | dfrA14 |
| H5 | Ga0110941_12085071 | dfrA21 |
| H5 | Ga0110941_10206731 | dfrF |
| H5 | Ga0110941_10967321 | emrA |
| H5 | Ga0110941_11731561 | emrA |
| H5 | Ga0110941_12240211 | emrA |
| H5 | Ga0110941_10609621 | emrB |
| H5 | Ga0110941_10855111 | emrB |
| H5 | Ga0110941_10995851 | emrB |
| H5 | Ga0110941_11232201 | emrB |
| H5 | Ga0110941_11363381 | emrB |
| H5 | Ga0110941_11731562 | emrB |
| H5 | Ga0110941_12159091 | emrB |
| H5 | Ga0110941_11613002 | emrD |
| H5 | Ga0110941_12375221 | emrD |
| H5 | Ga0110941_10259571 | emrE |
| H5 | Ga0110941_12144922 | emrK |
| H5 | Ga0110941_10967322 | emrR |
| H5 | Ga0110941_12061211 | emrR |
| H5 | Ga0110941_10974771 | emrY |
| H5 | Ga0110941_12144921 | emrY |
| H5 | Ga0110941_10054081 | ereA |
| H5 | Ga0110941_10054092 | ereA8 |
| H5 | Ga0110941_10520081 | ereB |
| H5 | Ga0110941_12301011 | ereB |
| H5 | Ga0110941_11378841 | Erm 23S ribosomal RNA Methyltransferase |
| H5 | Ga0110941_10090751 | ermB |
| H5 | Ga0110941_10179711 | ermF |
| H5 | Ga0110941_12380201 | ermG |
| H5 | Ga0110941_12106431 | ermX |
| H5 | Ga0110941_11081951 | evgA |
| H5 | Ga0110941_11092422 | evgA |
| H5 | Ga0110941_10164721 | evgS |
| H5 | Ga0110941_10284471 | evgS |
| H5 | Ga0110941_10284481 | evgS |
| H5 | Ga0110941_11081952 | evgS |
| H5 | Ga0110941_11296941 | evgS |
| H5 | Ga0110941_10061165 | Fluoroquinolone Resistant DNA Topoisomerase |
| H5 | Ga0110941_10072321 | Fluoroquinolone Resistant DNA Topoisomerase |
| H5 | Ga0110941_100903310 | Fluoroquinolone Resistant DNA Topoisomerase |
| H5 | Ga0110941_10151312 | Fluoroquinolone Resistant DNA Topoisomerase |
| H5 | Ga0110941_10211161 | Fluoroquinolone Resistant DNA Topoisomerase |
| H5 | Ga0110941_10313301 | Fluoroquinolone Resistant DNA Topoisomerase |
| H5 | Ga0110941_10363851 | Fluoroquinolone Resistant DNA Topoisomerase |
| H5 | Ga0110941_10458661 | Fluoroquinolone Resistant DNA Topoisomerase |
| H5 | Ga0110941_10478031 | Fluoroquinolone Resistant DNA Topoisomerase |
| H5 | Ga0110941_10493451 | Fluoroquinolone Resistant DNA Topoisomerase |
| H5 | Ga0110941_10548381 | Fluoroquinolone Resistant DNA Topoisomerase |
| H5 | Ga0110941_10640151 | Fluoroquinolone Resistant DNA Topoisomerase |
| H5 | Ga0110941_10699551 | Fluoroquinolone Resistant DNA Topoisomerase |
| H5 | Ga0110941_10699691 | Fluoroquinolone Resistant DNA Topoisomerase |
| H5 | Ga0110941_10712671 | Fluoroquinolone Resistant DNA Topoisomerase |
| H5 | Ga0110941_10729291 | Fluoroquinolone Resistant DNA Topoisomerase |
| H5 | Ga0110941_10906561 | Fluoroquinolone Resistant DNA Topoisomerase |
| H5 | Ga0110941_10956891 | Fluoroquinolone Resistant DNA Topoisomerase |
| H5 | Ga0110941_10980811 | Fluoroquinolone Resistant DNA Topoisomerase |
| H5 | Ga0110941_11007341 | Fluoroquinolone Resistant DNA Topoisomerase |
| H5 | Ga0110941_11046111 | Fluoroquinolone Resistant DNA Topoisomerase |
| H5 | Ga0110941_11094661 | Fluoroquinolone Resistant DNA Topoisomerase |
| H5 | Ga0110941_11181981 | Fluoroquinolone Resistant DNA Topoisomerase |
| H5 | Ga0110941_11291441 | Fluoroquinolone Resistant DNA Topoisomerase |
| H5 | Ga0110941_11505961 | Fluoroquinolone Resistant DNA Topoisomerase |
| H5 | Ga0110941_11505962 | Fluoroquinolone Resistant DNA Topoisomerase |
| H5 | Ga0110941_11517241 | Fluoroquinolone Resistant DNA Topoisomerase |
| H5 | Ga0110941_11629131 | Fluoroquinolone Resistant DNA Topoisomerase |
| H5 | Ga0110941_11654161 | Fluoroquinolone Resistant DNA Topoisomerase |
| H5 | Ga0110941_11699851 | Fluoroquinolone Resistant DNA Topoisomerase |
| H5 | Ga0110941_11766821 | Fluoroquinolone Resistant DNA Topoisomerase |
| H5 | Ga0110941_11766831 | Fluoroquinolone Resistant DNA Topoisomerase |
| H5 | Ga0110941_11779951 | Fluoroquinolone Resistant DNA Topoisomerase |
| H5 | Ga0110941_11806851 | Fluoroquinolone Resistant DNA Topoisomerase |
| H5 | Ga0110941_11858341 | Fluoroquinolone Resistant DNA Topoisomerase |
| H5 | Ga0110941_11895472 | Fluoroquinolone Resistant DNA Topoisomerase |
| H5 | Ga0110941_11909191 | Fluoroquinolone Resistant DNA Topoisomerase |
| H5 | Ga0110941_11974371 | Fluoroquinolone Resistant DNA Topoisomerase |
| H5 | Ga0110941_12051942 | Fluoroquinolone Resistant DNA Topoisomerase |
| H5 | Ga0110941_12097091 | Fluoroquinolone Resistant DNA Topoisomerase |
| H5 | Ga0110941_12103641 | Fluoroquinolone Resistant DNA Topoisomerase |
| H5 | Ga0110941_12163232 | Fluoroquinolone Resistant DNA Topoisomerase |
| H5 | Ga0110941_12193071 | Fluoroquinolone Resistant DNA Topoisomerase |
| H5 | Ga0110941_12224121 | Fluoroquinolone Resistant DNA Topoisomerase |
| H5 | Ga0110941_12378261 | Fluoroquinolone Resistant DNA Topoisomerase |
| H5 | Ga0110941_12389481 | Fluoroquinolone Resistant DNA Topoisomerase |
| H5 | Ga0110941_12435091 | Fluoroquinolone Resistant DNA Topoisomerase |
| H5 | Ga0110941_12529701 | Fluoroquinolone Resistant DNA Topoisomerase |
| H5 | Ga0110941_11542471 | fosA4 |
| H5 | Ga0110941_11098052 | gadX |
| H5 | Ga0110941_11337991 | gadX |
| H5 | Ga0110941_10162331 | *bla*_GES_ |
| H5 | Ga0110941_11634812 | *bla*_GES-21_ |
| H5 | Ga0110941_10555383 | golS |
| H5 | Ga0110941_11780661 | H-NS |
| H5 | Ga0110941_10080171 | *bla*_LCR-1_ |
| H5 | Ga0110941_11760681 | *bla*_LCR-1_ |
| H5 | Ga0110941_10093641 | lnuC |
| H5 | Ga0110941_10264443 | lnuC |
| H5 | Ga0110941_10264451 | lnuC |
| H5 | Ga0110941_12455451 | lsaE |
| H5 | Ga0110941_10472322 | macA |
| H5 | Ga0110941_10473691 | macA |
| H5 | Ga0110941_10138081 | macB |
| H5 | Ga0110941_10804981 | macB |
| H5 | Ga0110941_11134152 | macB |
| H5 | Ga0110941_11866331 | marA |
| H5 | Ga0110941_10755431 | mdfA |
| H5 | Ga0110941_12106411 | mdfA |
| H5 | Ga0110941_11467241 | mdsB |
| H5 | Ga0110941_11874111 | mdsB |
| H5 | Ga0110941_10312931 | mdtA |
| H5 | Ga0110941_10852611 | mdtA |
| H5 | Ga0110941_11824551 | mdtA |
| H5 | Ga0110941_10312932 | mdtB |
| H5 | Ga0110941_10707801 | mdtB |
| H5 | Ga0110941_11473411 | mdtB |
| H5 | Ga0110941_11829171 | mdtB |
| H5 | Ga0110941_12352161 | mdtB |
| H5 | Ga0110941_10331851 | mdtC |
| H5 | Ga0110941_11685221 | mdtC |
| H5 | Ga0110941_11691191 | mdtC |
| H5 | Ga0110941_12459111 | mdtC |
| H5 | Ga0110941_10331861 | mdtD |
| H5 | Ga0110941_10881801 | mdtF |
| H5 | Ga0110941_10764601 | mdtG |
| H5 | Ga0110941_11807721 | mdtH |
| H5 | Ga0110941_11884151 | mdtH |
| H5 | Ga0110941_12378001 | mdtH |
| H5 | Ga0110941_10449151 | mdtK |
| H5 | Ga0110941_10522681 | mdtK |
| H5 | Ga0110941_11678851 | mdtK |
| H5 | Ga0110941_11863021 | mdtK |
| H5 | Ga0110941_10397831 | mdtL |
| H5 | Ga0110941_12227932 | mdtM |
| H5 | Ga0110941_12428111 | mdtM |
| H5 | Ga0110941_10061331 | mdtN |
| H5 | Ga0110941_10499521 | mdtN |
| H5 | Ga0110941_10691321 | mdtO |
| H5 | Ga0110941_11298121 | mdtP |
| H5 | Ga0110941_11451551 | mdtP |
| H5 | Ga0110941_10716761 | mefA |
| H5 | Ga0110941_12130992 | mefB |
| H5 | Ga0110941_11157722 | mexB |
| H5 | Ga0110941_10871161 | mexD |
| H5 | Ga0110941_10477221 | mexK |
| H5 | Ga0110941_10635701 | mexK |
| H5 | Ga0110941_11991042 | mexK |
| H5 | Ga0110941_10320793 | mexT |
| H5 | Ga0110941_10758231 | mexT |
| H5 | Ga0110941_11620761 | mexT |
| H5 | Ga0110941_11891641 | mexT |
| H5 | Ga0110941_12544381 | mexT |
| H5 | Ga0110941_10889981 | MFS Antibiotic Efflux Pump |
| H5 | Ga0110941_11131361 | MFS Antibiotic Efflux Pump |
| H5 | Ga0110941_12259591 | MFS Antibiotic Efflux Pump |
| H5 | Ga0110941_10067791 | mphA |
| H5 | Ga0110941_10833311 | msbA |
| H5 | Ga0110941_11447222 | msbA |
| H5 | Ga0110941_10003231 | msrE |
| H5 | Ga0110941_10428231 | *bla*_OXA-1_ |
| H5 | Ga0110941_10087801 | *bla*_OXA-10_ |
| H5 | Ga0110941_10006806 | *bla*_OXA-12_ |
| H5 | Ga0110941_10076592 | *bla*_OXA-12_ |
| H5 | Ga0110941_10076791 | *bla*_OXA-12_ |
| H5 | Ga0110941_10143761 | *bla*_OXA-334_ |
| H5 | Ga0110941_10227172 | *bla*_OXA-347_ |
| H5 | Ga0110941_10650891 | *bla*_OXA-58_ |
| H5 | Ga0110941_10438881 | phoP |
| H5 | Ga0110941_10438951 | phoP |
| H5 | Ga0110941_10653631 | phoP |
| H5 | Ga0110941_11550782 | phoP |
| H5 | Ga0110941_10438882 | phoQ |
| H5 | Ga0110941_10438952 | phoQ |
| H5 | Ga0110941_11760771 | phoQ |
| H5 | Ga0110941_10945031 | PmrA |
| H5 | Ga0110941_11005222 | PmrB |
| H5 | Ga0110941_11183751 | PmrB |
| H5 | Ga0110941_11241751 | PmrB |
| H5 | Ga0110941_10766992 | PmrC |
| H5 | Ga0110941_10797121 | PmrC |
| H5 | Ga0110941_10846851 | PmrC |
| H5 | Ga0110941_10945021 | PmrC |
| H5 | Ga0110941_10497061 | PmrE |
| H5 | Ga0110941_10651141 | PmrE |
| H5 | Ga0110941_11418871 | PmrE |
| H5 | Ga0110941_10540651 | PmrF |
| H5 | Ga0110941_11608371 | PmrF |
| H5 | Ga0110941_11927802 | PmrF |
| H5 | Ga0110941_10155313 | QnrB4 |
| H5 | Ga0110941_10008512 | QnrS2 |
| H5 | Ga0110941_12077551 | QnrS8 |
| H5 | Ga0110941_11013871 | QnrVC4 |
| H5 | Ga0110941_12264761 | QnrVC7 |
| H5 | Ga0110941_11873451 | Quinolone Resistance Protein Qnr |
| H5 | Ga0110941_10184012 | ramA |
| H5 | Ga0110941_10035011 | RND Antibiotic Efflux Pump |
| H5 | Ga0110941_10053391 | RND Antibiotic Efflux Pump |
| H5 | Ga0110941_10059322 | RND Antibiotic Efflux Pump |
| H5 | Ga0110941_10059341 | RND Antibiotic Efflux Pump |
| H5 | Ga0110941_10072601 | RND Antibiotic Efflux Pump |
| H5 | Ga0110941_10113552 | RND Antibiotic Efflux Pump |
| H5 | Ga0110941_10172661 | RND Antibiotic Efflux Pump |
| H5 | Ga0110941_10204121 | RND Antibiotic Efflux Pump |
| H5 | Ga0110941_10207221 | RND Antibiotic Efflux Pump |
| H5 | Ga0110941_10237721 | RND Antibiotic Efflux Pump |
| H5 | Ga0110941_10244431 | RND Antibiotic Efflux Pump |
| H5 | Ga0110941_10284071 | RND Antibiotic Efflux Pump |
| H5 | Ga0110941_10335231 | RND Antibiotic Efflux Pump |
| H5 | Ga0110941_10412941 | RND Antibiotic Efflux Pump |
| H5 | Ga0110941_10418282 | RND Antibiotic Efflux Pump |
| H5 | Ga0110941_10425721 | RND Antibiotic Efflux Pump |
| H5 | Ga0110941_10499271 | RND Antibiotic Efflux Pump |
| H5 | Ga0110941_10732551 | RND Antibiotic Efflux Pump |
| H5 | Ga0110941_10791681 | RND Antibiotic Efflux Pump |
| H5 | Ga0110941_10823771 | RND Antibiotic Efflux Pump |
| H5 | Ga0110941_10861601 | RND Antibiotic Efflux Pump |
| H5 | Ga0110941_10896801 | RND Antibiotic Efflux Pump |
| H5 | Ga0110941_11006431 | RND Antibiotic Efflux Pump |
| H5 | Ga0110941_11055691 | RND Antibiotic Efflux Pump |
| H5 | Ga0110941_11092181 | RND Antibiotic Efflux Pump |
| H5 | Ga0110941_11125601 | RND Antibiotic Efflux Pump |
| H5 | Ga0110941_11305071 | RND Antibiotic Efflux Pump |
| H5 | Ga0110941_11337791 | RND Antibiotic Efflux Pump |
| H5 | Ga0110941_11378911 | RND Antibiotic Efflux Pump |
| H5 | Ga0110941_11401681 | RND Antibiotic Efflux Pump |
| H5 | Ga0110941_11475741 | RND Antibiotic Efflux Pump |
| H5 | Ga0110941_11660331 | RND Antibiotic Efflux Pump |
| H5 | Ga0110941_11673231 | RND Antibiotic Efflux Pump |
| H5 | Ga0110941_11695281 | RND Antibiotic Efflux Pump |
| H5 | Ga0110941_11808061 | RND Antibiotic Efflux Pump |
| H5 | Ga0110941_11834441 | RND Antibiotic Efflux Pump |
| H5 | Ga0110941_11855681 | RND Antibiotic Efflux Pump |
| H5 | Ga0110941_10669541 | robA |
| H5 | Ga0110941_10989381 | robA |
| H5 | Ga0110941_11272451 | robA |
| H5 | Ga0110941_11587251 | romA |
| H5 | Ga0110941_10927871 | rosA |
| H5 | Ga0110941_11554651 | rosA |
| H5 | Ga0110941_11837902 | rosA |
| H5 | Ga0110941_10028871 | rosB |
| H5 | Ga0110941_10763721 | rosB |
| H5 | Ga0110941_12188321 | sat-4 |
| H5 | Ga0110941_11560271 | smeE |
| H5 | Ga0110941_10975852 | soxR |
| H5 | Ga0110941_10975862 | soxR |
| H5 | Ga0110941_11231401 | soxR |
| H5 | Ga0110941_11924871 | soxR |
| H5 | Ga0110941_12266572 | soxR |
| H5 | Ga0110941_10007662 | sul1 |
| H5 | Ga0110941_10173051 | sul2 |
| H5 | Ga0110941_11115381 | sul3 |
| H5 | Ga0110941_10593861 | tet40 |
| H5 | Ga0110941_11255191 | tet40 |
| H5 | Ga0110941_11589351 | tet40 |
| H5 | Ga0110941_11954782 | tetA(P) |
| H5 | Ga0110941_10129267 | tetO |
| H5 | Ga0110941_10018929 | Tetracycline Resistance MFS Efflux Pump |
| H5 | Ga0110941_10026263 | Tetracycline Resistance MFS Efflux Pump |
| H5 | Ga0110941_10096011 | Tetracycline Resistance MFS Efflux Pump |
| H5 | Ga0110941_10281794 | Tetracycline Resistance MFS Efflux Pump |
| H5 | Ga0110941_10790672 | Tetracycline Resistance MFS Efflux Pump |
| H5 | Ga0110941_12336792 | Tetracycline Resistance MFS Efflux Pump |
| H5 | Ga0110941_12367801 | Tetracycline Resistance MFS Efflux Pump |
| H5 | Ga0110941_10009921 | Tetracycline Resistance Ribosomal Protection Protein |
| H5 | Ga0110941_10039442 | Tetracycline Resistance Ribosomal Protection Protein |
| H5 | Ga0110941_10249932 | Tetracycline Resistance Ribosomal Protection Protein |
| H5 | Ga0110941_10504602 | Tetracycline Resistance Ribosomal Protection Protein |
| H5 | Ga0110941_10626361 | Tetracycline Resistance Ribosomal Protection Protein |
| H5 | Ga0110941_10880961 | Tetracycline Resistance Ribosomal Protection Protein |
| H5 | Ga0110941_11381241 | Tetracycline Resistance Ribosomal Protection Protein |
| H5 | Ga0110941_11543751 | Tetracycline Resistance Ribosomal Protection Protein |
| H5 | Ga0110941_11808291 | Tetracycline Resistance Ribosomal Protection Protein |
| H5 | Ga0110941_12384191 | Tetracycline Resistance Ribosomal Protection Protein |
| H5 | Ga0110941_10306231 | tetX |
| H5 | Ga0110941_10169802 | tolC |
| H5 | Ga0110941_10730231 | tolC |
| H5 | Ga0110941_11727851 | tolC |
| H5 | Ga0110941_12002971 | tolC |
| H5 | Ga0110941_12364071 | tolC |
| H5 | Ga0110941_11638741 | TriC |
| H5 | Ga0110941_11124341 | vanW |
| H5 | Ga0110941_11149641 | *bla*_VEB-3_ |
| H5 | Ga0110941_10217591 | Beta-lactamase Class B |
| H5 | Ga0110941_10504232 | Beta-lactamase Class B |
| MA | Ga0110929_10014085 | APH3" |
| MA | Ga0110929_10031403 | APH3" |
| MA | Ga0110929_10000131 | APH3" |
| MA | Ga0110929_10240243 | APH3" |
| MA | Ga0110929_10154222 | APH3" |
| MA | Ga0110929_10017791 | APH3" |
| MA | Ga0110929_10000171 | APH3" |
| MA | Ga0110929_10517152 | APH3" |
| MA | Ga0110929_10584751 | APH3" |
| MA | Ga0110929_11150391 | APH3" |
| MA | Ga0110929_10258902 | APH3" |
| MA | Ga0110929_10032501 | Fluoroquinolone Resistant DNA Topoisomerase |
| MA | Ga0110929_10053991 | Fluoroquinolone Resistant DNA Topoisomerase |
| MA | Ga0110929_10079484 | Fluoroquinolone Resistant DNA Topoisomerase |
| MA | Ga0110929_10125771 | Fluoroquinolone Resistant DNA Topoisomerase |
| MA | Ga0110929_10161351 | Fluoroquinolone Resistant DNA Topoisomerase |
| MA | Ga0110929_10259872 | Fluoroquinolone Resistant DNA Topoisomerase |
| MA | Ga0110929_10071406 | Fluoroquinolone Resistant DNA Topoisomerase |
| MA | Ga0110929_10580191 | Fluoroquinolone Resistant DNA Topoisomerase |
| MA | Ga0110929_10139701 | Fluoroquinolone Resistant DNA Topoisomerase |
| MA | Ga0110929_10138251 | Fluoroquinolone Resistant DNA Topoisomerase |
| MA | Ga0110929_10449491 | Fluoroquinolone Resistant DNA Topoisomerase |
| MA | Ga0110929_10099381 | Fluoroquinolone Resistant DNA Topoisomerase |
| MA | Ga0110929_10244881 | Fluoroquinolone Resistant DNA Topoisomerase |
| MA | Ga0110929_10505991 | Fluoroquinolone Resistant DNA Topoisomerase |
| MA | Ga0110929_10408081 | Fluoroquinolone Resistant DNA Topoisomerase |
| MA | Ga0110929_10958331 | Fluoroquinolone Resistant DNA Topoisomerase |
| MA | Ga0110929_10088901 | Fluoroquinolone Resistant DNA Topoisomerase |
| MA | Ga0110929_10139691 | Fluoroquinolone Resistant DNA Topoisomerase |
| MA | Ga0110929_10244871 | Fluoroquinolone Resistant DNA Topoisomerase |
| MA | Ga0110929_10775131 | Fluoroquinolone Resistant DNA Topoisomerase |
| MA | Ga0110929_10132371 | Fluoroquinolone Resistant DNA Topoisomerase |
| MA | Ga0110929_10658851 | Fluoroquinolone Resistant DNA Topoisomerase |
| MA | Ga0110929_10712761 | Fluoroquinolone Resistant DNA Topoisomerase |
| MA | Ga0110929_10854121 | Fluoroquinolone Resistant DNA Topoisomerase |
| MA | Ga0110929_11757001 | Fluoroquinolone Resistant DNA Topoisomerase |
| MA | Ga0110929_12166192 | rifampin phosphotransferase |
| MA | Ga0110929_11883791 | RND Antibiotic Efflux Pump |
| MA | Ga0110929_11080471 | RND Antibiotic Efflux Pump |
| MA | Ga0110929_10540941 | RND Antibiotic Efflux Pump |
| MA | Ga0110929_11636291 | RND Antibiotic Efflux Pump |
| MA | Ga0110929_11444561 | RND Antibiotic Efflux Pump |
| MA | Ga0110929_11724222 | RND Antibiotic Efflux Pump |
| MA | Ga0110929_11477922 | RND Antibiotic Efflux Pump |
| MA | Ga0110929_11040971 | RND Antibiotic Efflux Pump |
| RA | Ga0110928_11312272 | aadA15 |
| RA | Ga0110928_11794951 | APH3" |
| RA | Ga0110928_10296272 | APH3" |
| RA | Ga0110928_11576051 | APH3" |
| RA | Ga0110928_10091042 | APH3" |
| RA | Ga0110928_10186231 | APH3" |
| RA | Ga0110928_10129351 | APH3" |
| RA | Ga0110928_10906731 | APH3" |
| RA | Ga0110928_10035822 | APH3" |
| RA | Ga0110928_10391481 | APH3" |
| RA | Ga0110928_10395041 | APH3" |
| RA | Ga0110928_10536921 | APH3" |
| RA | Ga0110928_11040292 | APH3" |
| RA | Ga0110928_10085971 | Fluoroquinolone Resistant DNA Topoisomerase |
| RA | Ga0110928_10123091 | Fluoroquinolone Resistant DNA Topoisomerase |
| RA | Ga0110928_11310941 | Fluoroquinolone Resistant DNA Topoisomerase |
| RA | Ga0110928_10321472 | Fluoroquinolone Resistant DNA Topoisomerase |
| RA | Ga0110928_11690721 | Fluoroquinolone Resistant DNA Topoisomerase |
| RA | Ga0110928_10247111 | Fluoroquinolone Resistant DNA Topoisomerase |
| RA | Ga0110928_10960791 | Fluoroquinolone Resistant DNA Topoisomerase |
| RA | Ga0110928_10859691 | Fluoroquinolone Resistant DNA Topoisomerase |
| RA | Ga0110928_12004131 | Fluoroquinolone Resistant DNA Topoisomerase |
| RA | Ga0110928_10778601 | mdtB |
| RA | Ga0110928_11182921 | mexK |
| RA | Ga0110928_11520842 | PmrE |
| RA | Ga0110928_11648921 | RND Antibiotic Efflux Pump |
| RA | Ga0110928_10651481 | RND Antibiotic Efflux Pump |
| RA | Ga0110928_11226861 | RND Antibiotic Efflux Pump |
| RA | Ga0110928_10494771 | RND Antibiotic Efflux Pump |
| RA | Ga0110928_10485651 | RND Antibiotic Efflux Pump |
| RA | Ga0110928_10485661 | RND Antibiotic Efflux Pump |
| RA | Ga0110928_10462111 | RND Antibiotic Efflux Pump |
| RA | Ga0110928_10555711 | RND Antibiotic Efflux Pump |
| RA | Ga0110928_10796912 | RND Antibiotic Efflux Pump |
| RA | Ga0110928_10969382 | RND Antibiotic Efflux Pump |
| RA | Ga0110928_11049101 | RND Antibiotic Efflux Pump |
| RA | Ga0110928_11210601 | RND Antibiotic Efflux Pump |
| TW1 | Ga0110934_11151951 | ABC Antibiotic Efflux Pump |
| TW1 | Ga0110934_10281301 | ANT3 |
| TW1 | Ga0110934_10762691 | APH(6)-Id |
| TW1 | Ga0110934_10880911 | APH3" |
| TW1 | Ga0110934_10323801 | APH3" |
| TW1 | Ga0110934_11880461 | APH3" |
| TW1 | Ga0110934_11503791 | APH3" |
| TW1 | Ga0110934_12283932 | APH3" |
| TW1 | Ga0110934_10291452 | APH3" |
| TW1 | Ga0110934_10820241 | APH3" |
| TW1 | Ga0110934_10939711 | Beta-lactamase Class D |
| TW1 | Ga0110934_11397441 | Beta-lactamase Class D |
| TW1 | Ga0110934_12400401 | Beta-lactamase Class D |
| TW1 | Ga0110934_10878671 | ereA |
| TW1 | Ga0110934_11510501 | ermF |
| TW1 | Ga0110934_11317251 | Fluoroquinolone Resistant DNA Topoisomerase |
| TW1 | Ga0110934_10214741 | Fluoroquinolone Resistant DNA Topoisomerase |
| TW1 | Ga0110934_10921421 | Fluoroquinolone Resistant DNA Topoisomerase |
| TW1 | Ga0110934_11538051 | Fluoroquinolone Resistant DNA Topoisomerase |
| TW1 | Ga0110934_10022721 | Fluoroquinolone Resistant DNA Topoisomerase |
| TW1 | Ga0110934_10207141 | Fluoroquinolone Resistant DNA Topoisomerase |
| TW1 | Ga0110934_12246261 | Fluoroquinolone Resistant DNA Topoisomerase |
| TW1 | Ga0110934_10186462 | Fluoroquinolone Resistant DNA Topoisomerase |
| TW1 | Ga0110934_10317191 | Fluoroquinolone Resistant DNA Topoisomerase |
| TW1 | Ga0110934_10385411 | Fluoroquinolone Resistant DNA Topoisomerase |
| TW1 | Ga0110934_11358582 | Fluoroquinolone Resistant DNA Topoisomerase |
| TW1 | Ga0110934_10196402 | Fluoroquinolone Resistant DNA Topoisomerase |
| TW1 | Ga0110934_11501911 | Fluoroquinolone Resistant DNA Topoisomerase |
| TW1 | Ga0110934_11837931 | Fluoroquinolone Resistant DNA Topoisomerase |
| TW1 | Ga0110934_10000821 | Fluoroquinolone Resistant DNA Topoisomerase |
| TW1 | Ga0110934_10730111 | *bla*_GES-19_ |
| TW1 | Ga0110934_10911302 | macB |
| TW1 | Ga0110934_10059431 | macB |
| TW1 | Ga0110934_10911291 | macB |
| TW1 | Ga0110934_11600041 | macB |
| TW1 | Ga0110934_11382261 | mdtB |
| TW1 | Ga0110934_10473441 | mexK |
| TW1 | Ga0110934_12125352 | mexT |
| TW1 | Ga0110934_11437681 | msrE |
| TW1 | Ga0110934_11322011 | msrE |
| TW1 | Ga0110934_12173181 | msrE |
| TW1 | Ga0110934_10217493 | mtrA |
| TW1 | Ga0110934_10935431 | *bla*_OXA-347_ |
| TW1 | Ga0110934_11032881 | PmrE |
| TW1 | Ga0110934_10553922 | RbpA |
| TW1 | Ga0110934_10223022 | RND Antibiotic Efflux Pump |
| TW1 | Ga0110934_11354681 | RND Antibiotic Efflux Pump |
| TW1 | Ga0110934_11353711 | RND Antibiotic Efflux Pump |
| TW1 | Ga0110934_10741291 | RND Antibiotic Efflux Pump |
| TW1 | Ga0110934_10473431 | RND Antibiotic Efflux Pump |
| TW1 | Ga0110934_10639102 | sul1 |
| TW1 | Ga0110934_12244531 | sul2 |
| TW1 | Ga0110934_11255181 | Tetracycline Resistance Ribosomal Protection Protein |
| TW1 | Ga0110934_10134611 | tetX |
| TW1 | Ga0110934_10017463 | vanX |
| TW2 | Ga0110935_12029551 | aadA13 |
| TW2 | Ga0110935_12193721 | ABC Antibiotic Efflux Pump |
| TW2 | Ga0110935_11081291 | adeJ |
| TW2 | Ga0110935_10285661 | ANT3 |
| TW2 | Ga0110935_12086141 | APH(3'')-Ib |
| TW2 | Ga0110935_12086142 | APH(6)-Id |
| TW2 | Ga0110935_10036001 | APH3" |
| TW2 | Ga0110935_10208121 | APH3" |
| TW2 | Ga0110935_10907172 | APH3" |
| TW2 | Ga0110935_11471582 | APH3" |
| TW2 | Ga0110935_10023681 | APH3" |
| TW2 | Ga0110935_11134631 | APH6 |
| TW2 | Ga0110935_11983311 | ereA |
| TW2 | Ga0110935_12401362 | ermB |
| TW2 | Ga0110935_11272881 | ermB |
| TW2 | Ga0110935_10701391 | Fluoroquinolone Resistant DNA Topoisomerase |
| TW2 | Ga0110935_10390841 | Fluoroquinolone Resistant DNA Topoisomerase |
| TW2 | Ga0110935_10041032 | Fluoroquinolone Resistant DNA Topoisomerase |
| TW2 | Ga0110935_10041051 | Fluoroquinolone Resistant DNA Topoisomerase |
| TW2 | Ga0110935_10078831 | Fluoroquinolone Resistant DNA Topoisomerase |
| TW2 | Ga0110935_10096313 | Fluoroquinolone Resistant DNA Topoisomerase |
| TW2 | Ga0110935_10451861 | Fluoroquinolone Resistant DNA Topoisomerase |
| TW2 | Ga0110935_10562741 | Fluoroquinolone Resistant DNA Topoisomerase |
| TW2 | Ga0110935_10788721 | Fluoroquinolone Resistant DNA Topoisomerase |
| TW2 | Ga0110935_11139741 | Fluoroquinolone Resistant DNA Topoisomerase |
| TW2 | Ga0110935_11436361 | Fluoroquinolone Resistant DNA Topoisomerase |
| TW2 | Ga0110935_11436362 | Fluoroquinolone Resistant DNA Topoisomerase |
| TW2 | Ga0110935_11503372 | Fluoroquinolone Resistant DNA Topoisomerase |
| TW2 | Ga0110935_11676681 | Fluoroquinolone Resistant DNA Topoisomerase |
| TW2 | Ga0110935_10999991 | Fluoroquinolone Resistant DNA Topoisomerase |
| TW2 | Ga0110935_11025851 | macB |
| TW2 | Ga0110935_11132902 | mdtB |
| TW2 | Ga0110935_11558131 | mdtB |
| TW2 | Ga0110935_10154282 | mexT |
| TW2 | Ga0110935_10821611 | mexT |
| TW2 | Ga0110935_100642010 | mtrA |
| TW2 | Ga0110935_11093822 | *bla*_OXA-198_ |
| TW2 | Ga0110935_11030121 | QnrS2 |
| TW2 | Ga0110935_10325034 | RbpA |
| TW2 | Ga0110935_10695351 | RND Antibiotic Efflux Pump |
| TW2 | Ga0110935_12207261 | RND Antibiotic Efflux Pump |
| TW2 | Ga0110935_10777431 | RND Antibiotic Efflux Pump |
| TW2 | Ga0110935_10167231 | RND Antibiotic Efflux Pump |
| TW2 | Ga0110935_10291721 | RND Antibiotic Efflux Pump |
| TW2 | Ga0110935_10341892 | RND Antibiotic Efflux Pump |
| TW2 | Ga0110935_10441141 | RND Antibiotic Efflux Pump |
| TW2 | Ga0110935_12102781 | RND Antibiotic Efflux Pump |
| TW2 | Ga0110935_10440872 | RND Antibiotic Efflux Pump |
| TW2 | Ga0110935_10372501 | sul1 |
| TW2 | Ga0110935_11684182 | sul2 |
| TW2 | Ga0110935_10610661 | sul3 |
| TW2 | Ga0110935_12263501 | tetO |
| TW2 | Ga0110935_10300302 | Tetracycline Resistance MFS Efflux Pump |
| TW2 | Ga0110935_10545261 | Tetracycline Resistance MFS Efflux Pump |
| TW2 | Ga0110935_11307681 | Tetracycline Resistance Ribosomal Protection Protein |
| TW2 | Ga0110935_12258312 | Tetracycline Resistance Ribosomal Protection Protein |
| TW2 | Ga0110935_11138641 | Tetracycline Resistance Ribosomal Protection Protein |
| TW2 | Ga0110935_11527361 | Tetracycline Resistance Ribosomal Protection Protein |
| TW2 | Ga0110935_11649851 | Tetracycline Resistance Ribosomal Protection Protein |
| TW2 | Ga0110935_10277381 | tetX |
| TW2 | Ga0110935_10240771 | vanX |
| TW2 | Ga0110935_11957541 | vatB |
| TW2 | Ga0110935_11410622 | *bla*_VEB-1a_ |
| WW | Ga0110933_10328112 | AAC(6')-Ib9 |
| WW | Ga0110933_12064521 | AAC(6')-Ie-APH(2'')-Ia |
| WW | Ga0110933_12160452 | AAC(6')-Ie-APH(2'')-Ia |
| WW | Ga0110933_12160461 | AAC(6')-Ie-APH(2'')-Ia |
| WW | Ga0110933_12415401 | AAC(6')-Ie-APH(2'')-Ia |
| WW | Ga0110933_11359101 | AAC98494.1 |
| WW | Ga0110933_11982121 | aadA11 |
| WW | Ga0110933_11292831 | aadA6/aadA10 |
| WW | Ga0110933_10106401 | ABC Antibiotic Efflux Pump |
| WW | Ga0110933_10124821 | ABC Antibiotic Efflux Pump |
| WW | Ga0110933_10732141 | ABC Antibiotic Efflux Pump |
| WW | Ga0110933_10807391 | ABC Antibiotic Efflux Pump |
| WW | Ga0110933_11190631 | abeM |
| WW | Ga0110933_11205571 | acrA |
| WW | Ga0110933_11958551 | acrA |
| WW | Ga0110933_11989461 | acrA |
| WW | Ga0110933_10055331 | acrB |
| WW | Ga0110933_10141461 | acrB |
| WW | Ga0110933_11317281 | acrB |
| WW | Ga0110933_11584151 | acrB |
| WW | Ga0110933_11655592 | acrB |
| WW | Ga0110933_11728901 | acrB |
| WW | Ga0110933_11911861 | acrB |
| WW | Ga0110933_12000801 | acrB |
| WW | Ga0110933_12370711 | acrB |
| WW | Ga0110933_10179351 | acrD |
| WW | Ga0110933_10842701 | acrD |
| WW | Ga0110933_12752031 | acrD |
| WW | Ga0110933_11320071 | acrE |
| WW | Ga0110933_13222631 | acrE |
| WW | Ga0110933_10338581 | acrF |
| WW | Ga0110933_10454121 | acrF |
| WW | Ga0110933_10486021 | acrF |
| WW | Ga0110933_10905121 | acrF |
| WW | Ga0110933_11117481 | acrF |
| WW | Ga0110933_12350481 | acrS |
| WW | Ga0110933_10427441 | adeB |
| WW | Ga0110933_11177031 | adeB |
| WW | Ga0110933_11781091 | adeB |
| WW | Ga0110933_10488201 | adeC-adeK-oprM |
| WW | Ga0110933_11929671 | adeC-adeK-oprM |
| WW | Ga0110933_12282751 | adeC-adeK-oprM |
| WW | Ga0110933_11404461 | adeJ |
| WW | Ga0110933_11496261 | adeJ |
| WW | Ga0110933_12381931 | ANT(2'')-Ia |
| WW | Ga0110933_11464441 | ANT(9)-Ia |
| WW | Ga0110933_11178631 | ANT2 |
| WW | Ga0110933_10079301 | ANT3 |
| WW | Ga0110933_10319081 | ANT3 |
| WW | Ga0110933_10891001 | ANT3 |
| WW | Ga0110933_10276541 | ANT6 |
| WW | Ga0110933_10276551 | ANT6 |
| WW | Ga0110933_10453571 | ANT6 |
| WW | Ga0110933_11947051 | ANT6 |
| WW | Ga0110933_10015555 | APH3" |
| WW | Ga0110933_10333231 | APH3" |
| WW | Ga0110933_10424131 | APH3" |
| WW | Ga0110933_10514413 | APH3" |
| WW | Ga0110933_10818861 | APH3" |
| WW | Ga0110933_10891102 | APH3" |
| WW | Ga0110933_11408821 | APH3" |
| WW | Ga0110933_11465482 | APH3" |
| WW | Ga0110933_11662753 | APH3" |
| WW | Ga0110933_11807901 | APH3" |
| WW | Ga0110933_12252711 | APH3" |
| WW | Ga0110933_12977441 | APH3" |
| WW | Ga0110933_11473081 | APH3' |
| WW | Ga0110933_11473091 | APH3' |
| WW | Ga0110933_10514412 | APH6 |
| WW | Ga0110933_10329041 | arnA |
| WW | Ga0110933_11424701 | arnA |
| WW | Ga0110933_11795661 | arnA |
| WW | Ga0110933_12042642 | arnA |
| WW | Ga0110933_12347101 | arnA |
| WW | Ga0110933_11339501 | bacA |
| WW | Ga0110933_11937251 | bacA |
| WW | Ga0110933_10239522 | baeR |
| WW | Ga0110933_10239531 | baeR |
| WW | Ga0110933_10698251 | baeR |
| WW | Ga0110933_11492941 | baeR |
| WW | Ga0110933_10239521 | baeS |
| WW | Ga0110933_10696401 | Cfr 23 Ribosomal RNA Methyltransferase |
| WW | Ga0110933_10954311 | Cfr 23 Ribosomal RNA Methyltransferase |
| WW | Ga0110933_12396671 | Cfr 23 Ribosomal RNA Methyltransferase |
| WW | Ga0110933_12616061 | Cfr 23 Ribosomal RNA Methyltransferase |
| WW | Ga0110933_10012061 | cfxA6 |
| WW | Ga0110933_10454871 | Chloramphenicol Acetyltransferase CAT |
| WW | Ga0110933_10585521 | Chloramphenicol Acetyltransferase CAT |
| WW | Ga0110933_12188261 | Chloramphenicol Acetyltransferase CAT |
| WW | Ga0110933_13271391 | Chloramphenicol Acetyltransferase CAT |
| WW | Ga0110933_13286421 | Chloramphenicol Acetyltransferase CAT |
| WW | Ga0110933_10079303 | Chloramphenicol Efflux Pump |
| WW | Ga0110933_10134813 | Beta-lactamase Class A |
| WW | Ga0110933_11531931 | Beta-lactamase Class A |
| WW | Ga0110933_11784701 | Beta-lactamase Class A |
| WW | Ga0110933_13074371 | Beta-lactamase Class A |
| WW | Ga0110933_10041582 | ClassC-AmpC |
| WW | Ga0110933_11683051 | ClassC-AmpC |
| WW | Ga0110933_10079302 | Beta-lactamase Class D |
| WW | Ga0110933_11055491 | Beta-lactamase Class D |
| WW | Ga0110933_11906931 | Beta-lactamase Class D |
| WW | Ga0110933_11982351 | Beta-lactamase Class D |
| WW | Ga0110933_12575431 | Beta-lactamase Class D |
| WW | Ga0110933_10379581 | cpxA |
| WW | Ga0110933_10549311 | cpxA |
| WW | Ga0110933_11882791 | cpxA |
| WW | Ga0110933_12806131 | cpxA |
| WW | Ga0110933_11971951 | cpxR |
| WW | Ga0110933_10541352 | CRP |
| WW | Ga0110933_10973401 | CRP |
| WW | Ga0110933_11383111 | CRP |
| WW | Ga0110933_12320272 | CRP |
| WW | Ga0110933_12729062 | CRP |
| WW | Ga0110933_11672181 | dfrA14 |
| WW | Ga0110933_11618762 | dfrA3 |
| WW | Ga0110933_10544021 | dfrF |
| WW | Ga0110933_10407712 | emrA |
| WW | Ga0110933_11568681 | emrA |
| WW | Ga0110933_10697181 | emrB |
| WW | Ga0110933_11789292 | emrB |
| WW | Ga0110933_12166491 | emrB |
| WW | Ga0110933_12544511 | emrB |
| WW | Ga0110933_13308841 | emrB |
| WW | Ga0110933_11214501 | emrK |
| WW | Ga0110933_11743081 | emrK |
| WW | Ga0110933_10407711 | emrR |
| WW | Ga0110933_10531271 | emrR |
| WW | Ga0110933_11146571 | emrR |
| WW | Ga0110933_10152191 | ereA |
| WW | Ga0110933_10793731 | ereB |
| WW | Ga0110933_11097451 | ereB |
| WW | Ga0110933_11375021 | ermA |
| WW | Ga0110933_10225461 | ermB |
| WW | Ga0110933_10255571 | ermB |
| WW | Ga0110933_10004232 | ermF |
| WW | Ga0110933_10732143 | ermG |
| WW | Ga0110933_10079152 | evgA |
| WW | Ga0110933_10079151 | evgS |
| WW | Ga0110933_10614581 | evgS |
| WW | Ga0110933_12476711 | evgS |
| WW | Ga0110933_13210281 | evgS |
| WW | Ga0110933_13440551 | evgS |
| WW | Ga0110933_10095681 | Fluoroquinolone Resistant DNA Topoisomerase |
| WW | Ga0110933_10150311 | Fluoroquinolone Resistant DNA Topoisomerase |
| WW | Ga0110933_10254941 | Fluoroquinolone Resistant DNA Topoisomerase |
| WW | Ga0110933_10275881 | Fluoroquinolone Resistant DNA Topoisomerase |
| WW | Ga0110933_10281651 | Fluoroquinolone Resistant DNA Topoisomerase |
| WW | Ga0110933_10281671 | Fluoroquinolone Resistant DNA Topoisomerase |
| WW | Ga0110933_10297291 | Fluoroquinolone Resistant DNA Topoisomerase |
| WW | Ga0110933_10333961 | Fluoroquinolone Resistant DNA Topoisomerase |
| WW | Ga0110933_10342851 | Fluoroquinolone Resistant DNA Topoisomerase |
| WW | Ga0110933_10537131 | Fluoroquinolone Resistant DNA Topoisomerase |
| WW | Ga0110933_10655661 | Fluoroquinolone Resistant DNA Topoisomerase |
| WW | Ga0110933_10695521 | Fluoroquinolone Resistant DNA Topoisomerase |
| WW | Ga0110933_10758601 | Fluoroquinolone Resistant DNA Topoisomerase |
| WW | Ga0110933_10770681 | Fluoroquinolone Resistant DNA Topoisomerase |
| WW | Ga0110933_10908571 | Fluoroquinolone Resistant DNA Topoisomerase |
| WW | Ga0110933_10983951 | Fluoroquinolone Resistant DNA Topoisomerase |
| WW | Ga0110933_11014391 | Fluoroquinolone Resistant DNA Topoisomerase |
| WW | Ga0110933_11014441 | Fluoroquinolone Resistant DNA Topoisomerase |
| WW | Ga0110933_11115571 | Fluoroquinolone Resistant DNA Topoisomerase |
| WW | Ga0110933_11227151 | Fluoroquinolone Resistant DNA Topoisomerase |
| WW | Ga0110933_11380421 | Fluoroquinolone Resistant DNA Topoisomerase |
| WW | Ga0110933_11395241 | Fluoroquinolone Resistant DNA Topoisomerase |
| WW | Ga0110933_11412792 | Fluoroquinolone Resistant DNA Topoisomerase |
| WW | Ga0110933_11448711 | Fluoroquinolone Resistant DNA Topoisomerase |
| WW | Ga0110933_11479581 | Fluoroquinolone Resistant DNA Topoisomerase |
| WW | Ga0110933_11491521 | Fluoroquinolone Resistant DNA Topoisomerase |
| WW | Ga0110933_11741271 | Fluoroquinolone Resistant DNA Topoisomerase |
| WW | Ga0110933_11827742 | Fluoroquinolone Resistant DNA Topoisomerase |
| WW | Ga0110933_11917961 | Fluoroquinolone Resistant DNA Topoisomerase |
| WW | Ga0110933_11989731 | Fluoroquinolone Resistant DNA Topoisomerase |
| WW | Ga0110933_11989732 | Fluoroquinolone Resistant DNA Topoisomerase |
| WW | Ga0110933_12081331 | Fluoroquinolone Resistant DNA Topoisomerase |
| WW | Ga0110933_12108811 | Fluoroquinolone Resistant DNA Topoisomerase |
| WW | Ga0110933_12124681 | Fluoroquinolone Resistant DNA Topoisomerase |
| WW | Ga0110933_12167421 | Fluoroquinolone Resistant DNA Topoisomerase |
| WW | Ga0110933_12176761 | Fluoroquinolone Resistant DNA Topoisomerase |
| WW | Ga0110933_12199202 | Fluoroquinolone Resistant DNA Topoisomerase |
| WW | Ga0110933_12218831 | Fluoroquinolone Resistant DNA Topoisomerase |
| WW | Ga0110933_12439521 | Fluoroquinolone Resistant DNA Topoisomerase |
| WW | Ga0110933_12545901 | Fluoroquinolone Resistant DNA Topoisomerase |
| WW | Ga0110933_12844931 | Fluoroquinolone Resistant DNA Topoisomerase |
| WW | Ga0110933_12971441 | Fluoroquinolone Resistant DNA Topoisomerase |
| WW | Ga0110933_13004862 | Fluoroquinolone Resistant DNA Topoisomerase |
| WW | Ga0110933_13167811 | Fluoroquinolone Resistant DNA Topoisomerase |
| WW | Ga0110933_10283771 | fosA4 |
| WW | Ga0110933_11882881 | fosA5 |
| WW | Ga0110933_11075801 | gadX |
| WW | Ga0110933_10207601 | *bla*_GES_ |
| WW | Ga0110933_11127522 | H-NS |
| WW | Ga0110933_12350121 | *bla*_LCR-1_ |
| WW | Ga0110933_11066441 | lmrD |
| WW | Ga0110933_10241603 | lnuC |
| WW | Ga0110933_10178592 | lnuD |
| WW | Ga0110933_11130761 | lnuD |
| WW | Ga0110933_11032831 | macA |
| WW | Ga0110933_12136291 | macA |
| WW | Ga0110933_13053281 | macA |
| WW | Ga0110933_13350701 | macA |
| WW | Ga0110933_10560911 | macB |
| WW | Ga0110933_11287061 | macB |
| WW | Ga0110933_11499581 | macB |
| WW | Ga0110933_12012762 | macB |
| WW | Ga0110933_12012951 | macB |
| WW | Ga0110933_12259991 | macB |
| WW | Ga0110933_12716331 | macB |
| WW | Ga0110933_11365172 | mdfA |
| WW | Ga0110933_12594161 | mdtA |
| WW | Ga0110933_11350251 | mdtB |
| WW | Ga0110933_12142551 | mdtB |
| WW | Ga0110933_13211641 | mdtB |
| WW | Ga0110933_10295791 | mdtC |
| WW | Ga0110933_11651691 | mdtC |
| WW | Ga0110933_12073671 | mdtC |
| WW | Ga0110933_12717111 | mdtC |
| WW | Ga0110933_10139801 | mdtE |
| WW | Ga0110933_10163381 | mdtE |
| WW | Ga0110933_11492631 | mdtF |
| WW | Ga0110933_12451031 | mdtF |
| WW | Ga0110933_12675641 | mdtF |
| WW | Ga0110933_13107471 | mdtF |
| WW | Ga0110933_10014411 | mdtH |
| WW | Ga0110933_10002511 | mdtK |
| WW | Ga0110933_10735621 | mdtK |
| WW | Ga0110933_10595801 | mdtL |
| WW | Ga0110933_10595811 | mdtL |
| WW | Ga0110933_10679942 | mdtM |
| WW | Ga0110933_11587861 | mdtM |
| WW | Ga0110933_12205761 | mdtM |
| WW | Ga0110933_10172682 | mdtN |
| WW | Ga0110933_10717551 | mdtO |
| WW | Ga0110933_12454882 | mdtO |
| WW | Ga0110933_12454881 | mdtP |
| WW | Ga0110933_13045761 | mdtP |
| WW | Ga0110933_10124822 | mefA |
| WW | Ga0110933_10497041 | mefB |
| WW | Ga0110933_11635422 | mexB |
| WW | Ga0110933_11419601 | mexF |
| WW | Ga0110933_12034792 | mexF |
| WW | Ga0110933_13127491 | mexF |
| WW | Ga0110933_12065901 | mexT |
| WW | Ga0110933_12135391 | mexT |
| WW | Ga0110933_10990461 | MFS Antibiotic Efflux Pump |
| WW | Ga0110933_12977721 | mphA |
| WW | Ga0110933_10563601 | msbA |
| WW | Ga0110933_10148081 | msrE |
| WW | Ga0110933_10579472 | *bla*_OXA-333_ |
| WW | Ga0110933_10004231 | *bla*_OXA-347_ |
| WW | Ga0110933_12492081 | PBP1a |
| WW | Ga0110933_10236361 | PBP2b |
| WW | Ga0110933_10158041 | phoP |
| WW | Ga0110933_10788272 | phoP |
| WW | Ga0110933_10158051 | phoQ |
| WW | Ga0110933_10432901 | PmrB |
| WW | Ga0110933_10027731 | PmrC |
| WW | Ga0110933_11586591 | PmrC |
| WW | Ga0110933_13007581 | PmrC |
| WW | Ga0110933_10679281 | PmrE |
| WW | Ga0110933_11370331 | PmrE |
| WW | Ga0110933_11627521 | PmrE |
| WW | Ga0110933_11960491 | PmrE |
| WW | Ga0110933_12176702 | PmrE |
| WW | Ga0110933_12211431 | PmrE |
| WW | Ga0110933_12468541 | PmrE |
| WW | Ga0110933_12751571 | PmrE |
| WW | Ga0110933_12999831 | PmrE |
| WW | Ga0110933_10191131 | PmrF |
| WW | Ga0110933_12248201 | QnrD1 |
| WW | Ga0110933_10062091 | QnrS2 |
| WW | Ga0110933_11420121 | QnrVC1 |
| WW | Ga0110933_10328113 | QnrVC4 |
| WW | Ga0110933_12232221 | QnrVC6 |
| WW | Ga0110933_10541761 | ramA |
| WW | Ga0110933_12993571 | ramA |
| WW | Ga0110933_10084181 | RND Antibiotic Efflux Pump |
| WW | Ga0110933_10173281 | RND Antibiotic Efflux Pump |
| WW | Ga0110933_10191201 | RND Antibiotic Efflux Pump |
| WW | Ga0110933_10317662 | RND Antibiotic Efflux Pump |
| WW | Ga0110933_10341111 | RND Antibiotic Efflux Pump |
| WW | Ga0110933_10363972 | RND Antibiotic Efflux Pump |
| WW | Ga0110933_10630251 | RND Antibiotic Efflux Pump |
| WW | Ga0110933_11197941 | RND Antibiotic Efflux Pump |
| WW | Ga0110933_11238141 | RND Antibiotic Efflux Pump |
| WW | Ga0110933_11375571 | RND Antibiotic Efflux Pump |
| WW | Ga0110933_11838171 | RND Antibiotic Efflux Pump |
| WW | Ga0110933_11851411 | RND Antibiotic Efflux Pump |
| WW | Ga0110933_11945161 | RND Antibiotic Efflux Pump |
| WW | Ga0110933_12034791 | RND Antibiotic Efflux Pump |
| WW | Ga0110933_12246571 | RND Antibiotic Efflux Pump |
| WW | Ga0110933_12376511 | RND Antibiotic Efflux Pump |
| WW | Ga0110933_12560071 | RND Antibiotic Efflux Pump |
| WW | Ga0110933_12644771 | RND Antibiotic Efflux Pump |
| WW | Ga0110933_12644781 | RND Antibiotic Efflux Pump |
| WW | Ga0110933_12729351 | RND Antibiotic Efflux Pump |
| WW | Ga0110933_12744831 | RND Antibiotic Efflux Pump |
| WW | Ga0110933_13170062 | RND Antibiotic Efflux Pump |
| WW | Ga0110933_13376121 | RND Antibiotic Efflux Pump |
| WW | Ga0110933_10336091 | robA |
| WW | Ga0110933_12469951 | robA |
| WW | Ga0110933_10541763 | romA |
| WW | Ga0110933_12026911 | romA |
| WW | Ga0110933_12993572 | romA |
| WW | Ga0110933_10177451 | rosB |
| WW | Ga0110933_11140121 | rosB |
| WW | Ga0110933_10722362 | sdiA |
| WW | Ga0110933_11255361 | sdiA |
| WW | Ga0110933_11706481 | soxR |
| WW | Ga0110933_12341142 | soxR |
| WW | Ga0110933_10319082 | sul1 |
| WW | Ga0110933_10670531 | sul2 |
| WW | Ga0110933_12543201 | *bla*_TEM_ |
| WW | Ga0110933_12893892 | *bla*_TEM_ |
| WW | Ga0110933_10338101 | tet40 |
| WW | Ga0110933_11433331 | tetA(P) |
| WW | Ga0110933_13141751 | tetA(P) |
| WW | Ga0110933_10120672 | tetO |
| WW | Ga0110933_10018694 | Tetracycline Resistance MFS Efflux Pump |
| WW | Ga0110933_10594301 | Tetracycline Resistance MFS Efflux Pump |
| WW | Ga0110933_10913452 | Tetracycline Resistance MFS Efflux Pump |
| WW | Ga0110933_11837081 | Tetracycline Resistance MFS Efflux Pump |
| WW | Ga0110933_12504691 | Tetracycline Resistance MFS Efflux Pump |
| WW | Ga0110933_12557731 | Tetracycline Resistance MFS Efflux Pump |
| WW | Ga0110933_12677261 | Tetracycline Resistance MFS Efflux Pump |
| WW | Ga0110933_13151001 | Tetracycline Resistance MFS Efflux Pump |
| WW | Ga0110933_100012411 | Tetracycline Resistance Ribosomal Protection Protein |
| WW | Ga0110933_10116371 | Tetracycline Resistance Ribosomal Protection Protein |
| WW | Ga0110933_10133061 | Tetracycline Resistance Ribosomal Protection Protein |
| WW | Ga0110933_10170121 | Tetracycline Resistance Ribosomal Protection Protein |
| WW | Ga0110933_10705581 | Tetracycline Resistance Ribosomal Protection Protein |
| WW | Ga0110933_11424401 | Tetracycline Resistance Ribosomal Protection Protein |
| WW | Ga0110933_11872051 | Tetracycline Resistance Ribosomal Protection Protein |
| WW | Ga0110933_12637801 | Tetracycline Resistance Ribosomal Protection Protein |
| WW | Ga0110933_13066791 | Tetracycline Resistance Ribosomal Protection Protein |
| WW | Ga0110933_10173211 | tetX |
| WW | Ga0110933_11239921 | TLA-2 |
| WW | Ga0110933_10406941 | tolC |
| WW | Ga0110933_13096341 | tolC |
| WW | Ga0110933_13278002 | vanRD |
| WW | Ga0110933_11130762 | vatB |
| WW | Ga0110933_10656111 | *bla*_VEB-1a_ |


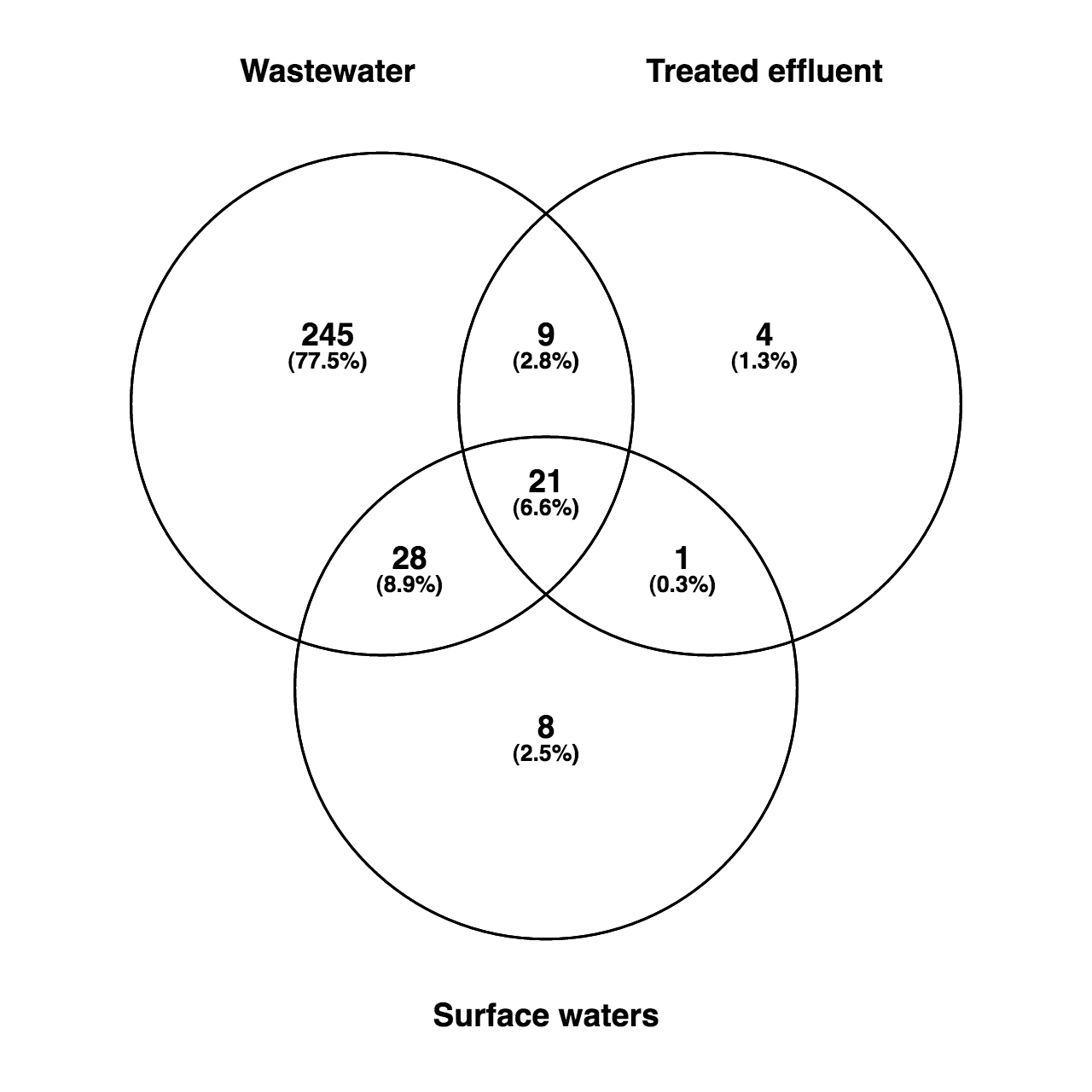


Figure S1. The share resistome between wastewater, treated effluents and surface waters.


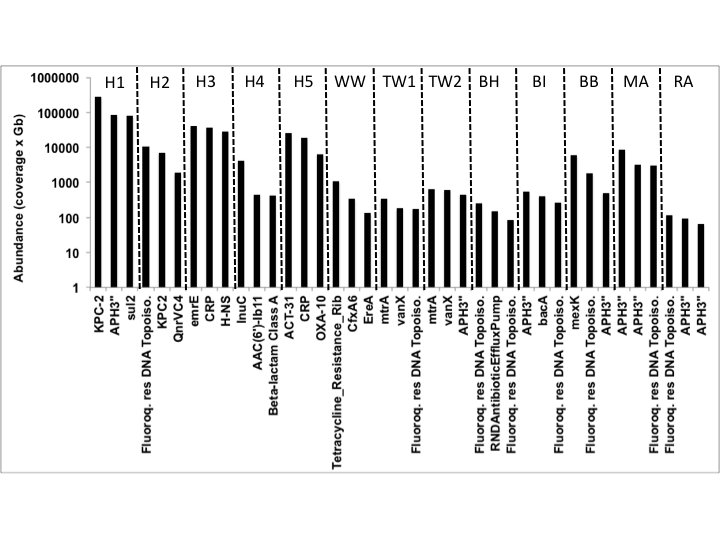


Figure S2. Top 3 most abundant ARGs


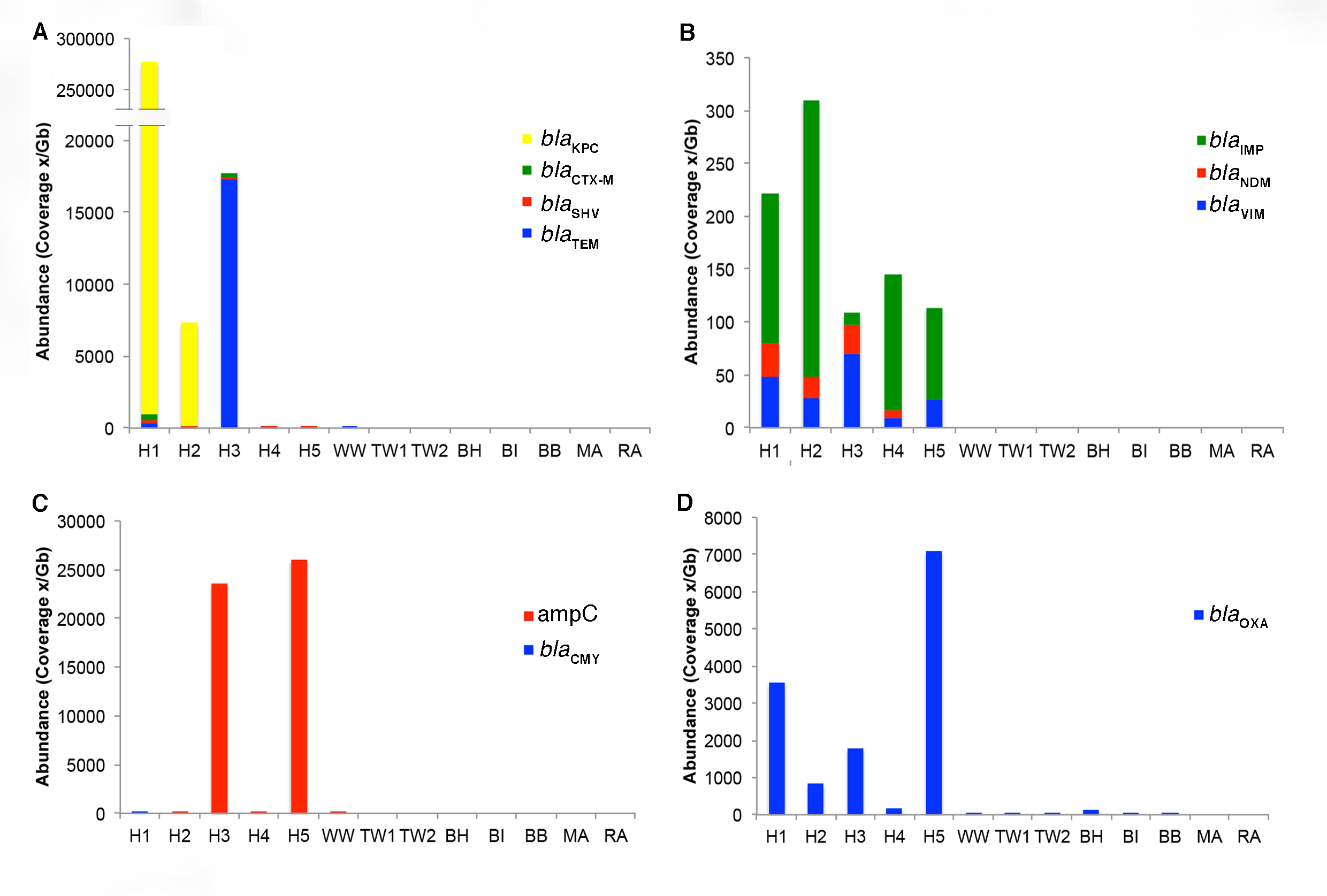


Figure S3. Abundance of clinically important beta lactamase (A) class A, (B) class B, (C) class C and (D) class D resistant genes.
